# Isolation of an elusive phosphatetrahedrane

**DOI:** 10.1126/sciadv.aaz3168

**Published:** 2020-03-25

**Authors:** Martin-Louis Y. Riu, Rebecca L. Jones, Wesley J. Transue, Peter Müller, Christopher C. Cummins

**Affiliations:** Department of Chemistry, Massachusetts Institute of Technology, Cambridge MA, USA.

## Abstract

This exploratory synthesis investigation was undertaken to determine the viability of replacing a single carbon vertex with another p-block element in a highly strained tetrahedrane molecule. Phosphorus was selected for this purpose because the stable molecular form of elemental phosphorus is tetrahedral. Our synthetic strategy was to generate an unsaturated phosphorus center bonded to a substituted cyclopropenyl group, a situation that could lead to closure to provide the desired phosphatetrahedrane framework. This was accomplished by dehydrofluorination of the in situ generated fluorophosphine H(F)P(C*^t^*Bu)_3_. Tri-*tert*-butyl phosphatetrahedrane, P(C*^t^*Bu)_3_, was then isolated in 19% yield as a low-melting, volatile, colorless solid and characterized spectroscopically and by a single-crystal x-ray diffraction study, confirming the tetrahedral nature of the molecule’s PC_3_ core. The molecule exhibits unexpected thermal stability.

## Teaser

Extreme strain eased with P-incorporation.

## INTRODUCTION

**Fig. 1:**
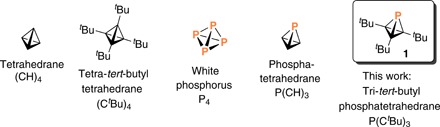
Chart of compounds relevant to the present study.

Molecules possessed of unusually acute bond angles at carbon are considered to be strained (*1*), high-energy species, for which tetrahedrane (Fig. 1)—the hydrocarbon whose carbon atoms describe the vertices of a regular tetrahedron—presents a limiting case. Strained cages such as tetrahedranes are interesting structural components for the design of novel high–energy density materials (*2*). While the parent tetrahedrane molecule has remained elusive, it is still considered to be a viable target (*3*). Ultimately, the successful isolation of molecules containing the tetrahedrane core of four carbon atoms has relied on the judicious choice of substituents to encage that reactive core, surrounding it with a protective barrier as in the case of tetra-*tert*-butyl tetrahedrane (*4*). A complementary approach is the inclusion of other elements into the tetrahedral core (*5*). Phosphorus has been referred to as “the carbon copy” as it approximates the electronegativity of carbon and carbon’s ability to form multiple bonds; these properties form the basis of phospha-organic chemistry (*6*). In the context of highly strained organic systems, we determine whether it is possible to replace a single core carbon atom of a tetrahedrane with phosphorus to yield a stable molecular entity.

The notion to create a phosphatetrahedrane is logical, given the tetrahedral nature of the P_4_ molecule, the only stable molecular form of elemental phosphorus (*7*). Many isolable compounds are known for which very small bond angles at trivalent phosphorus vertices obtain, suggesting that the impact of strain on stability associated with small bond angles at phosphorus is much less severe than is the case for carbon. Parent phosphatetrahedrane P(CH)_3_ has been contemplated by theorists who found eight structural isomers residing at lower energy than the tetrahedron (*8*). In other theoretical work, it has been predicted that phosphatetrahedrane molecules will behave as carbon bases upon gas-phase protonation (*9*).

**Fig. 2:**
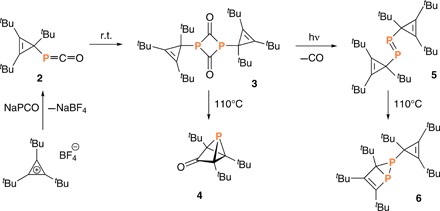
Synthesis of phosphaketene 2, diphosphene 5, and phosphorus analogs of tricyclopentanone 4 and housene 6 (*14*). r.t., room temperature.

Given that substitution with bulky groups has been the key to the stabilization of (CR)_4_ tetrahedranes (*4*, *10*–*13*), we selected P(C*^t^*Bu)_3_ (**1**, Fig. 1) as our target molecule. To approach such a target, a strategy analogous to that used in preparing some of the (CR)_4_ compounds would be to first synthesize a compound having the general formula (LG)P(cyclopropenyl), where LG is a neutral leaving group and the cyclopropenyl group carries three bulky substituents. In their elegant work on synthesis of phosphorus analogs of cyclopentadienone, tricyclopentanone, and housene, Slootweg and co-workers (*14*) generated such a compound with the formula (OC)P(C*^t^*Bu)_3_ (**2**, Fig. 2), with CO as the potential neutral leaving group and *tert*-butyl substituents on the cyclopropenyl ring. Phosphaketene **2** was found to be unstable with respect to dimerization, and the dimer **3** (Fig. 2) could be induced to extrude CO photochemically resulting in formation of diphosphene **5**. Heating phosphaketene dimer **3** resulted in the remarkable ketone **4**, whose structure exhibits two C─P─C bond angles registering less than 50°! Underscoring the chemical richness of this system, heating diphosphene **5** led to **6**, a diphosphorus analog of housene. It is notable that none of these reactions led to production of **1**, which the authors referred to as the “elusive phosphatetrahedrane”; compounds **5** and **6** have chemical formulas, making them formal dimers of **1**, while compounds **2** and **4** differ from **1** by only a CO molecule. A molecule related to **1** was reported while the present manuscript was in revision: di-*tert*-butyl diphosphatetrahedrane P_2_(C*^t^*Bu)_2_, similarly referred to as “elusive,” was obtained by catalytic dimerization of the phosphaalkyne *^t^*BuCP (*15*).

## RESULTS AND DISCUSSION

**Fig. 3:**
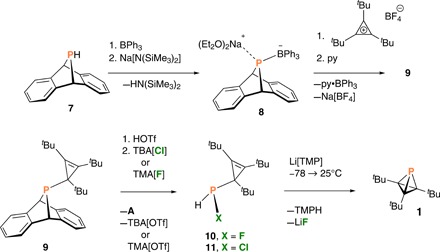
Synthesis of tri-*tert*-butyl phosphatetrahedrane 1. TBA, tetra-*n*-butyl ammonium; TMA, tetramethylammonium; TMP, tetramethylpiperidide; TMPH, tetramethylpiperidine.

**Fig. 4:**
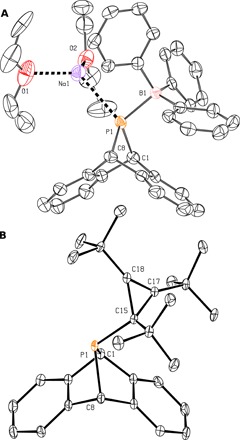
Molecular structures of key intermediates obtained from single-crystal x-ray diffraction experiments. (**A**) Drawing of Na[**8**] with thermal ellipsoids shown at the 50% probability level. Hydrogen atoms have been omitted. (**B**) Drawing of compound **9** with thermal ellipsoids shown at the 50% probability level. Hydrogen atoms have been omitted.

On the basis of our experience with phosphinidene transfer reactivity (*16*), we chose to prepare compound **A**P(C*^t^*Bu)_3_ (**9**; **A** = anthracene or C_14_H_10_), analogous to **2** but with anthracene in place of CO as a neutral leaving group. Because the secondary phosphine HP**A** (**7**) (*17*) exhibited no reaction with the tri-*tert*-butyl cyclopropenium ion, used as its tetrafluoroborate salt (*18*), we turned to the conjugate base of **7**. Deprotonation of HP**A** was accomplished in the presence of triphenylborane using sodium hexamethyldisilazide as the base, resulting in formation of [Na(OEt_2_)_2_][Ph_3_BP**A**] (Na[**8**]), which could be collected by filtration after precipitation from the crude reaction mixture in ca. 83% yield (Fig. 3). The borane-stabilized salt Na[**8**] has been characterized by x-ray crystallography as its bis diethyl etherate (Fig. 4A).

The borane-stabilized [P**A**]^−^ anion **8** combines smoothly with the tri-*tert*-butyl cyclopropenium ion to provide the desired cyclopropenyl phosphine **9** upon elimination of sodium tetrafluoroborate and dissociation of triphenylborane. Compound **9** was characterized in a single-crystal x-ray diffraction study revealing the molecular structure depicted in Fig. 4B.

Cyclopropenyl phosphine **9** proved to be thermally stable to at least its melting point of 130°C, so photochemical experiments were undertaken to induce anthracene elimination. Brief periods of irradiation (254 nm, 25°C, 10 min, hexanes) led to production of a species having a ^31^P nuclear magnetic resonance (NMR) signal at δ − 487.98 parts per million (ppm) tentatively identified as the desired phosphatetrahedrane **1** as one component of a complex mixture and under low conversion of **9**; extended periods of irradiation led to loss of the intriguing high-field NMR signal and an increase in the complexity of the reaction mixture.

Since the cyclopropenyl phosphinidene that would be produced upon anthracene loss from **9** is expected to strongly favor a triplet ground state (*19*), consistent with the observed thermal stability of **9**, we next opted to pursue an alternative strategy inspired by the long-studied reactivity of carbenoids (*20*). Carbenoids are carbenes stabilized in their singlet state, for example, by association with MX (M = e.g. Li and Na; X = halogen) and valued for their ability to effect reactions such as cyclopropanation, germane to the present target.

Gudat and co-workers (*21*) recently pointed out that “The stability of mixed H/X-substituted phosphines is greatly enhanced by introducing a bulky substituent”. Given the sterically bulky nature of the tri-*tert*-butyl cyclopropenyl substituent, accordingly, a cyclopropenyl halophosphine of formula HXP(C*^t^*Bu)_3_ seemed reasonable as a potential precursor to a phosphinidenoid (*22*) that could close to phosphatetrahedrane **1** upon deprotonation and ensuing MX elimination.

We have shown that halide addition to phosphonium compounds based on the P**A** framework can induce elimination of anthracene (*23*), pointing to a novel way to generate HXP(C*^t^*Bu)_3_ cyclopropenyl halophosphines from compound **9**. Treatment of **9** first with triflic acid to affect P-protonation, and second with [TBA]Cl or [TMA]F to form a P─X bond with anthracene elimination, led to the desired compounds HXP(C*^t^*Bu)_3_ (X = F, **10**; X = Cl, **11**; Fig. 3) as assessed by ^1^H NMR [**10**: δ 7.28 (P-H), ^1^*J*_PH_ = 187 Hz, ^2^*J*_FH_ = 45 Hz] and ^31^P NMR (**10**: δ 182.05, ^1^*J*_PH_ = 187 Hz, ^1^*J*_PF_ = 801 Hz) spectroscopy.

**Fig. 5:**
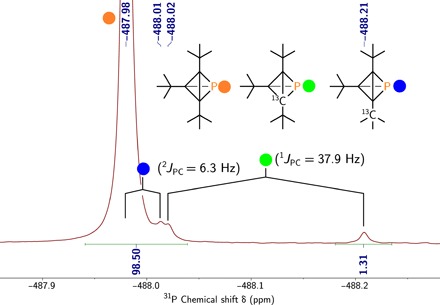
^31^P NMR spectrum (202 MHz, benzene-*d*_6_, 25°C) of compound 1. Main peak at −487.98 ppm with ^13^C satellites centered at −488.11 and −487.99 ppm for one- and two-bond couplings, respectively.

While chlorophosphine **11** initially appeared to undergo smooth dehydrohalogenation upon treatment with Na[N(SiMe_3_)_2_], with production of phosphatetrahedrane **1** as a major product as assayed by ^31^P NMR spectroscopy, this method proved irreproducible. Dehydrohalogenation of in situ–generated fluorophosphine **10** upon treatment with lithium tetramethylpiperidide (Fig. 3) proved to be an efficient, reproducible route to the target molecule, tri-*tert*-butyl phosphatetrahedrane **1**. The optimized protocol delivers compound **1** as the major product according to ^31^P NMR spectroscopy. Moreover, integration of the natural abundance ^13^C satellites associated with the high-field ^31^P NMR signal (Fig. 5, δ −487.98) is consistent with three equivalent carbon atoms bonded to the single phosphorus atom of the threefold symmetric phosphatetrahedrane structure assigned to **1**. Note also that the one-bond ^13^C satellites (^1^*J*_PC_ = 37.9 Hz) are isotope shifted to higher field than the main ^31^P signal by ca. 0.13 ppm, on par with some of the largest reported one-bond ^31^P^13^C isotope shifts (*24*). The two-bond ^2^*J*_PC_ splitting of 6.3 Hz indicated in Fig. 5 is derived from the ^13^C NMR spectrum, and the left branch of the doublet is inferred for the ^31^P NMR spectrum due to overlap with the main peak. Tri-*tert*-butyl phosphatetrahedrane **1** is characterized by ^13^C NMR signals at δ (ppm) 31.02 (methyl groups), 27.62 (tertiary *tert*-butyl carbons), and 25.22 (core carbon atoms) and a single ^1^H NMR resonance of chemical shift of 1.17 ppm.

Crude samples of phosphatetrahedrane **1** were initially obtained in the form of a pale yellow oil. This crude material was purified by simple distillation under vacuum at 23°C, affording **1** as a colorless oil. Further purification was achieved by passing a pentane solution of **1** through a silica plug to deliver, upon solvent removal under vacuum, colorless solid samples of phosphatetrahedrane **1**. The title compound is a waxy, low-melting solid (m.p. ca. 31°C) obtained in 19% yield from cyclopropenyl phosphine **9** according to the sequence of Fig. 3. We attribute the low isolated yield of **1** to losses due to volatility incurred during isolation and purification, as the chemistry of phosphatetrahedrane formation takes place with good efficiency according to spectroscopic monitoring before workup.

**Fig. 6:**
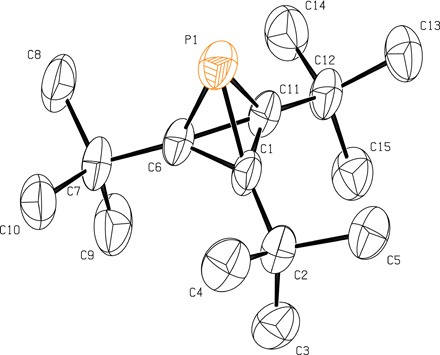
Structural drawing of tri-*tert*-butyl phosphatetrahedrane 1 from a single-crystal x-ray diffraction experiment. Thermal ellipsoids are shown at the 50% probability level, and hydrogen atoms have been omitted.

To grow crystals of phosphatetrahedrane **1** of suitable quality for an x-ray diffraction study, we turned to sublimation to leverage the volatile nature of this compound. While the obtained crystals of this low-melting solid were of low quality and diffracted the x-ray radiation poorly, the data obtained were fortunately sufficient for a structure determination (Fig. 6). The molecule crystallized in the space group *P*2_1_/*n* with the whole molecule in the asymmetric unit, such that the threefold molecular symmetry indicated by the solution ^1^H,^13^C, and ^31^P NMR spectroscopic characterization of compound **1**, is not reflected in the crystal symmetry. The observed structure from the x-ray determination is consistent with predictions from quantum chemical calculations, with the observed C─P─C bond angles being 47.1(4)° on average.

An initial thermal stability test showed phosphatetrahedrane **1** to be unchanged after heating at 75°C for 45 min as a solution in benzene-*d*_6_. This thermal stability is interesting, given that heating tetra-*tert*-butyl tetrahedrane to 130°C led to quantitative conversion to the isomeric cyclobutadiene (*4*). It has been predicted that the parent phosphacyclobutadiene is more stable than phosphatetrahedrane but only by 2.8 kcal/mol (*8*).

In the case of tri-*tert*-butyl phosphatetrahedrane **1** versus its phosphacyclobutadiene counterpart, the order of isomer stability is reversed according to our calculations using the G3(MP2, CCSD(T)) methodology (*25*), indicating that the heat of formation at 298 K is ca. 10.0 kcal/mol higher for the phosphacyclobutadiene form. Further heating of **1** (toluene-*d*_8_, 130°C, 3 hours, flame-sealed NMR tube) induced its partial conversion (ca. 60%) to the known phosphorus analog of housene (**6**, Fig. 2) that is a dimer of P(C*^t^*Bu)_3_ (*14*), as assessed by ^31^P NMR spectroscopy.

In addition to its considerable thermal stability, we find **1** to be at least briefly air stable, surviving exposure for half an hour at room temperature as a benzene-*d*_6_ solution. On the contrary, phosphatetrahedrane **1** is not stable to 254-nm ultraviolet irradiation being consumed to the extent of ca. 75% after 5 min (25°C), with formation of a number of presently unknown products according to the ^31^P NMR data. Among the new products formed was diphosphene **5** identified by its diagnostic low-field ^31^P NMR signal (δ 588.86), as reported by Slootweg and co-workers (*14*).

Preliminary reactivity studies demonstrate that phosphatetrahedrane **1** is highly susceptible to dimerization in the presence of a Lewis acid. For example, treatment of **1** with tungsten pentacarbonyl tetrahydrofuran provides uncomplexed housene **6** in ca. 85% yield, as assessed by ^31^P{^1^H} NMR spectroscopy. Moreover, treatment of the phosphatetrahedrane with a substoichiometric amount of triphenylborane generates a new ^31^P NMR signal at −47.60 ppm, which we tentatively assign to a [2+2] dimer of tri-*tert*-butyl phosphacyclobutadiene, and housene **6** in a 4:1 ratio. Further characterization of this dimer is in progress.

**Fig. 7:**
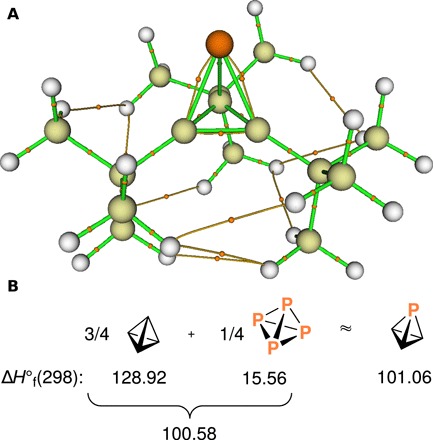
Analysis of bonding in compound 1 using quantum chemical calculations. (**A**) Molecular graph of P(C*^t^*Bu)_3_ (**1**) showing paths linking pairs of bonded atoms, bond critical points as small orange spheres, the phosphorus atom as a large orange sphere, carbon atoms as beige spheres, and hydrogen atoms as white spheres. (**B**) Standard heats of formation in kcal/mol at 298.15 K for tetrahedrane, P_4_, and phosphatetrahedrane from G3(MP2, CCSD(T)) calculations (*25*) performed using GAMESS (*37*). The phosphatetrahedrane Δ*H*∘f value can be approximated as the sum of three-quarters the value for tetrahedrane and one-quarter the value for the P_4_ molecule.

Quantum chemical calculations were used to illuminate the bonding in compound **1**. Topological analysis of the computed electron density (*26*) at the B3LYP-D3/6-31G** level of density functional theory revealed the molecular graph shown in Fig. 7A. A salient feature is that the bond paths connecting the phosphorus atom with the three core carbon atoms deviate substantially from the shortest path straight lines connecting the atoms, consistent with the very high atomic p-orbital character of the hybrids used by P in forming these bonds, as assessed by natural bond orbital analysis (*27*). The second feature of interest is the network of nine hydrogen-hydrogen bonds (*28*) serving as a glue to bind together the three *tert*-butyl groups. This is the physical basis of the so-called corset effect originally invoked to explain the stability of tetra-*tert*-butyl tetrahedrane (*4*). Compound **1** provides another nice illustration that three bulky substituents are sufficient to produce an isolable tetrahedrane (*29*).

The question of phosphatetrahedrane intrinsic stability vis-à-vis tetrahedrane can be probed with thermochemical considerations. Figure 7B illustrates our finding that the computed heat of formation of phosphatetrahedrane is quite well approximated by the sum of the appropriately weighted heats of formation of tetrahedrane and P_4_. This signifies that any change in the energy of the (CH)_3_ group on moving from tetrahedrane to the phosphatetrahedrane environment must be offset by a nearly equal and opposite change in the energy of the P atom on moving from P_4_ to phosphatetrahedrane.

Recently, atomic energies from Kohn-Sham calculations were shown to be useful reactivity descriptors within a model interacting quantum atoms (IQA) approach that does not suffer from the application of a biased scaling procedure (*30*). Using this model IQA approach for wave functions computed at the M06-2X/6-31G** level of theory led to the finding of (CH)_3_ group stabilization in phosphatetrahedrane relative to its energy in tetrahedrane by −65.7 kcal/mol and P-atom destabilization in phosphatetrahedrane relative to P_4_ by the nearly equal and opposite amount of +67.3 kcal/mol. This finding is in line with our chemical intuition that replacement of a single CH vertex by P in tetrahedrane should result in a core that is overall less strained. The respective stabilization and destabilization of the (CH)_3_ and P fragments within phosphatetrahedrane are connected to the development of partial positive charge at P (natural charge, +0.54) (*27*) and corresponding partial negative charge increase at C (from −0.26 to −0.45 for each C atom on going from tetrahedrane to phosphatetrahedrane). A practical consequence of this observation is that substituents able to delocalize negative charge ought to give rise to more intrinsically stable phosphatetrahedrane derivatives.

Natural bond orbital analysis (*27*) indicates that there is no notable change in the lone pair composition from P_4_ to phosphatetrahedrane (ca. 80% s in character), and likewise, the natural atomic orbital contributions to the bonds in these molecules differ almost not at all. The central bonds of the tetrahedral cores of the molecules shown in Fig. 7B are very high in p-orbital content, more so for P than for C. The C atom directs an external hybrid orbital that is 40% in s character, which, as discussed by Wiberg *et al.* (*31*), is the origin of the high strain energy for tetrahedrane (C is stabilized relative to the carbon in the standard methine group, but H is destabilized by an even greater amount).

## CONCLUSION

For tri-*tert*-butyl phosphatetrahedrane, the present work provides proof of existence of a molecule with the smallest sum of bond angles (141°; cf. 180° for P_4_!) presently conceivable for a trivalent phosphorus atom. The successful synthesis of phosphatetrahedrane **1** relied on the development of novel phosphinidenoid reaction chemistry, which remains to be elucidated in mechanistic detail and which may be applicable to other strained synthetic targets as a P─C bond-forming methodology.

## MATERIALS AND METHODS

### General methods

Except as otherwise noted, all manipulations were performed in a Vacuum Atmospheres model MO-40 M glovebox under an inert atmosphere of purified N_2_. All solvents were obtained anhydrous and oxygen free by bubble degassing (Ar) and purification through columns of alumina using a solvent purification system (Pure Process Technology, Nashua, NH) (*32*) and storage over 4.0-Å molecular sieves (*33*). Deuterated solvents were purchased from Cambridge Isotope Labs, then degassed, and stored over molecular sieves for at least 48 hours before use. Celite (EM Science), 4.0-Å molecular sieves, silica, acidic alumina, and charcoal were dried by heating above 200°C under dynamic vacuum (50 mtorr) for at least 48 hours before use. All glassware was dried in an oven for at least 2 hours at temperatures greater than 150°C.

Tri-*tert*-butyl cyclopropenyl tetrafluoroborate (*18*) and HP**A** (**7**, **A** = 9,10-dihydroanthracene-9,10-diyl) (*17*) were prepared according to literature procedures. Triphenylborane (Strem Chemicals), triphenylphosphine (Sigma-Aldrich), sodium bis(trimethylsilyl)amide (Sigma-Aldrich), lithium 2,2,6,6-tetramethylpiperidide (Sigma-Aldrich), trifluoromethanesulfonic acid (Strem Chemicals), and tungsten hexacarbonyl (Strem Chemicals) were used as received. Lithium 2,2,6,6-tetramethylpiperidide was also prepared according to a literature procedure (*34*). Pyridine (Sigma-Aldrich) was distilled under air, degassed three times by the freeze-pump-thaw method, and stored over 4-Å molecular sieves for 48 hours before use. Tetramethylammonium fluoride (Sigma-Aldrich) was dried at 100°C under reduced pressure (50 mtorr) for 48 hours before use. Tetrabutylammonium chloride (Sigma-Aldrich) was dried at 60°C under reduced pressure (50 mtorr) for 48 hours and crystallized from acetonitrile/pentane before use.

NMR spectra were obtained on a Jeol ECZ-500 instrument equipped with an Oxford Instruments superconducting magnet, on a Bruker Avance 400 instrument equipped with a Magnex Scientific or with a SpectroSpin superconducting magnet, or on a Bruker Avance 500 instrument equipped with a Magnex Scientific or with a SpectroSpin superconducting magnet. ^1^H and ^13^C NMR spectra were referenced internally to residual solvent signals (*35*). ^31^P NMR spectra were externally referenced to 85% H_3_PO_4_ (0 ppm). ^11^B NMR spectra were externally referenced to BF_3_-OEt_2_ (0 ppm). ^19^F NMR spectra were externally referenced to CFCl_3_ (0 ppm). Elemental combustion analyses were performed by Midwest Micro Laboratories (Indianapolis, IN, USA).

High-resolution mass spectral (HRMS) data were collected using a Jeol AccuTOF 4G LC-Plus mass spectrometer equipped with an Ion-Sense direct analysis in real time (DART) source. Data were calibrated to a sample of PEG-600 and were collected in positive-ion mode. Samples were prepared in tetrahydrofuran (THF) (10 μM concentration) and were briefly exposed to air (<5 s) before being placed in front of the DART source.

Photochemical reactions were performed using a Rayonet photochemical reactor RPR-200 (Southern New England Ultra Violet Company) loaded with 16 RPR-2537A lamps, each emitting ca. 35 W at 253.7 nm. Raman spectra were collected using a Renishaw Invia Reflex Micro Raman.

### Synthesis of [Na(OEt_2_)_2_][Ph_3_BPA] (Na[8])

#### Aluminum foil was used to limit exposure to ambient light during this experiment

A 250-ml flask charged with a solution of HP**A** (**7**, 0.850 g, 4.04 mmol, 1.00 equiv) and triphenylborane (1.10 g, 4.55 mmol, 1.13 equiv) in diethyl ether (60 ml) was frozen in the liquid nitrogen–cooled coldwell of the glovebox. Separately, a solution of sodium bis(trimethylsilyl)amide (0.834 g, 4.55 mmol, 1.13 equiv) in diethyl ether (10 ml) was prepared and frozen in the coldwell of the glovebox. Upon thawing, the sodium bis(trimethylsilyl)amide solution was added rapidly to the thawing solution of **7**. The solution became white and heterogeneous as it warmed with rapid stirring. After 20 min, the colorless precipitate was collected by vacuum filtration and washed with Et_2_O (2 × 10 ml). This afforded colorless powder of Na[**8**] (2.09 g, 3.34 mmol, 83%). The number of Et_2_O molecules in the formula has been determined by x-ray crystallography and by integration of ^1^H NMR signals. Compound Na[**8**] melts from 50 to 55°C. This sensitive material has not passed elemental analysis, being reproducibly low in carbon. Attempts to purify this compound by crystallization in minimal Et_2_O consistently results in the consumption of Na[**8**] and the formation of anthracene together with unidentified products. Elemental analysis found (calcd) for C_40_H_45_BNaO_2_P from two separate batch preparations: C, 75.16 (77.17); H, 6.61 (7.29); N, <0.02 (0.00) and C, 75.59 (77.17); H, 7.05 (7.29); N, <0.02(0.00). ^1^H NMR (400 MHz, THF-*d*_8_, 25°C) δ 7.21 (d, *J* = 7.3 Hz, 6H), 6.98 to 6.91 (m, 2H), 6.85 (t, *J* = 7.3 Hz, 6H), 6.72 (t, *J* = 7.1 Hz, 3H), 6.66 to 6.62 (m, 2H), 6.62 to 6.58 (m, 2H), 6.24 to 6.20 (m, 2H), 3.86 (d, ^2^*J*_PH_ = 13.2 Hz, 2H), 3.39 (q, *J* = 7.0 Hz, 4H), 1.12 (t, *J* = 7.0 Hz, 6H) ppm. ^11^B{^1^H} NMR (128 MHz, THF-*d*_8_, 25°C) δ −6.47 (br s) ppm. ^13^C{^1^H} NMR (101 MHz, THF-*d*_8_, 25°C) δ 154.76 (d, *J* = 12.3 Hz), 151.34, 136.25 (d, *J* = 8.6 Hz), 126.44, 123.67, 123.28, 122.69, 121.93, 120.51 (d, *J* = 6.4 Hz), 66.49, 54.66 (d, ^1^*J*_PC_ = 17.3 Hz), 15.85 ppm. ^31^P{^1^H} NMR (162 MHz, THF-*d*_8_, 25°C) δ 272.13 (br s) ppm.

### Synthesis of (*^t^*BuC)_3_PA (9)

#### Aluminum foil was used to limit exposure of the reaction mixture to ambient light during this experiment

A 100-ml flask charged with a solution of Na[**8**] (1.00 g, 1.61 mmol, 1.00 equiv) in THF (10 mL) and a Teflon-coated magnetic stir bar was frozen in the liquid nitrogen–cooled coldwell of the glovebox. Separately, a solution of tri-*tert*-butyl cyclopropenyl tetrafluoroborate (*18*) (0.473 g, 1.61 mmol, 1.00 equiv) in THF (20 ml) was prepared and frozen in the coldwell of the glovebox. Upon thawing, the solution of tri-*tert*-butyl cyclopropenyl tetrafluoroborate was rapidly added to the thawing solution of Na[**8**]. The solution became cloudy as it warmed with rapid stirring. After 1 hour, the solution was filtered through a coarse sintered frit (15 ml) containing a 3 cm plug of Celite. All volatile materials were removed in vacuo, and the resulting white solids were taken up in hexanes (16 ml). Pyridine (ca. 12 drops) was added to the solution, causing precipitation of the triphenylborane adduct of pyridine as a colorless solid (*36*). The reaction mixture was filtered through a coarse sintered frit (15 ml) containing a 5 cm plug of charcoal, and the plug was washed with hexanes (15 ml). All volatile materials were removed in vacuo from the combined filtrates yielding colorless solids. Crystallization from minimal pentane at −35°C provided colorless crystals of **9** (576 mg, 1.38 mmol, 86%).Compound **9** melts from 127 to 130°C. While **9** was not observed by DART HRMS, anthracene ([M + H]^+^ calcd for C_14_H_10_, 179.0846; found, 179.0861) and [*^t^*Bu_3_C_3_]^+^ ([M]^+^ calcd for C_15_H_27_, 207.2113; found, 207.2129) were observed. This material has not passed elemental analysis, being reproducibly low in carbon. Elemental analysis found (calcd) for C_29_H_37_P from two separate batch preparations: C, 81.84 (83.61); H, 9.44 (8.70); N, <0.02 (0.00) and 80.59(83.61); H, 8.95 (8.70); N, <0.02 (0.00). ^1^H NMR (400 MHz, chloroform-*d*, 25°C) δ 7.27 (ddd, *J* = 5.1, 3.2, 1.3 Hz, 2H), 7.22 (dd, *J* = 5.3, 3.1 Hz, 2H), 6.99 (dd, *J* = 5.4, 3.1 Hz, 2H), 6.88 (dd, *J* = 5.3, 3.1 Hz, 2H), 4.21 (d, ^2^*J*_PH_ = 13.4 Hz, 2H), 1.12 (s, 18H), 0.92 (s, 9H) ppm. ^13^C{^1^H} NMR (101 MHz, chloroform-*d*, 25°C) δ 148.91 (d, *J* = 1.9 Hz), 147.40 (d, *J* = 20.4 Hz), 127.72 (d, *J* = 4.4 Hz), 125.52, 124.82, 123.93, 122.28 (d, *J* = 3.0 Hz), 54.44 (d, ^1^*J*_PC_ = 23.4 Hz), 45.82 (d, *J* = 59.4 Hz), 37.55 (d, *J* = 19.5 Hz), 31.61, 31.25 (d, J = 2.3 Hz), 30.40 (d, *J* = 4.9 Hz) ppm. ^31^P{^1^H} NMR (162 MHz, chloroform-*d*, 25°C) δ 199.4 (t, ^2^*J*_PH_ = 13.4 Hz) ppm.

### Synthesis of (*^t^*BuC)_3_P (1)

A solution of trifluoromethanesulfonic acid (0.108 g, 0.719 mmol, 1.00 equiv) was added to a 20-ml scintillation vial charged with a solution of (*^t^*BuC)_3_P**A** (**9**, 0.300 g, 0.719 mmol, 1.00 equiv) in THF (2 ml) and a Teflon-coated magnetic stir bar. After 20 min, a slurry of tetramethylammonium fluoride (0.067 g, 0.719 mmol, 1.00 equiv) in THF (1 ml) was added dropwise. After the addition, the colorless heterogeneous solution was stirred for 30 min. All volatile materials were then removed in vacuo from the solution, resulting in a colorless residue. This material was slurried in pentane (5 ml), and the solution was filtered through a coarse sintered frit (15 ml) containing a 3 cm plug of charcoal. The plug was washed with pentane (10 ml). All volatile materials were then removed in vacuo from the combined filtrates, resulting in a colorless oil. This oil was taken up in THF (2 ml) to give a solution of (*^t^*BuC)_3_P(F)H (**10**) that was frozen in the liquid nitrogen–cooled coldwell of the glovebox. Separately, a solution of lithium tetramethylpiperidide (0.106 g, 0.719 mmol, 1.00 equiv) in THF (2 ml) was prepared and frozen in the liquid nitrogen–cooled coldwell of the glovebox. Upon thawing, the lithium tetramethylpiperidide solution was added dropwise to the thawing solution of **10**. After 20 min, all volatile materials were removed in vacuo, yielding colorless solids. The solids were taken up in pentane (2 ml), and the solution was filtered through a coarse sintered frit (15 ml) containing a 3 cm plug of acidic alumina. The plug was subsequently washed with pentane (1 ml). Under reduced pressure, all volatile materials were removed from the combined filtrates, yielding a pale yellow oil (87 mg). This oil was transferred to a Teflon-sealed trap-to-trap distillation apparatus, which was removed from the glovebox, connected to a Schlenk line, and placed under static vacuum (50 mtorr). One trap was kept at 23°C by using an oil bath, while the other trap was cooled to −78°C by using a mixture of dry ice and acetone. After 2 hours, colorless oil was collected in the −78°C trap, while a yellow gel was formed in the 23°C trap.

The apparatus was removed from the two baths, backfilled with nitrogen, and brought into the glovebox. The colorless oil was taken up in pentane (0.5 ml), and the solution was filtered through a glass fiber filter paper plugged pipette containing a 3 cm plug of silica. The plug was subsequently washed with pentane (1.5 ml). All volatile materials were removed from the combined filtrates under reduced pressure, yielding colorless solids (33 mg, 0.138 mmol, 19%). Compound **1** melts from 31 to 34°C. DART HRMS [(quadrupole orthogonal acceleration–time-of-flight (Q-TOF)] *m*/*z*: [M + H]^+^ calcd for C_15_H_28_P, 239.1929; found, 239.1931. Elemental analysis found (calcd) for C_15_H_27_P: C, 73.95(75.59); H, 11.32 (11.42); N, <0.02(0.00). ^1^H NMR (400 MHz, benzene-*d*_6_, 25°C) δ 1.17 (s, 27H) ppm. ^13^C{^1^H} NMR (101 MHz, benzene-*d*_6_, 25°C) δ 31.02, 27.62 (d, ^2^*J*_PC_ = 6.3 Hz), 25.22 (d, ^1^*J*_PC_ = 37.9 Hz) ppm. ^31^P{^1^H} NMR (162 MHz, benzene-*d*_6_, 25°C) δ −487.98 ppm.

## Supplementary Material

aaz3168_SM.pdf

## SUPPLEMENTARY MATERIALS

Supplementary material for this article is available at http://advances.sciencemag.org/cgi/content/full/6/13/eaaz3168/DC1 Section S1. Synthetic details and characterization of products Section S2. X-ray diffraction studies Section S3. Computational studies Fig. S1. Labeling scheme for Na[**8**]. Fig. S2. ^1^H NMR (400 MHz, THF-*d*_8_, 25°C) spectrum of Na[**8**]. Fig. S3. ^11^B{^1^H} NMR (128 MHz, THF-*d*_8_, 25°C) spectrum of Na[**8**]. Fig. S4. ^13^C{^1^H} NMR (101 MHz, THF-*d*_8_, 25°C) spectrum of Na[**8**]. Fig. S5. ^31^P{^1^H} NMR (162 MHz, THF-*d*_8_, 25°C) spectrum of Na[**8**]. Fig. S6. Labeling scheme for **9**. Fig. S7. ^1^H NMR (400 MHz, chloroform-*d*, 25°C) spectrum of **9**. Fig. S8. ^13^C{^1^H} NMR (101 MHz, chloroform-*d*, 25°C) spectrum of **9**. Fig. S9. ^31^P{^1^H} NMR (162 MHz, chloroform-*d*, 25°C) spectrum of **9**. Fig. S10. ^31^P{^1^H} NMR (162 MHz, toluene-*d*_8_, 25°C) spectrum after heating **9** to 110°C in toluene-*d*_8_ for 22 hours. Fig. S11. ^1^H NMR (162 MHz, benzene-*d*_6_, 25°C) spectrum after melting **9** at 130°C. Fig. S12. ^31^{^1^H} NMR (162 MHz, benzene-*d*_6_, 25°C) spectrum after melting **9** at 130°C. Fig. S13. ^31^P{^1^H} NMR (162 MHz, hexanes, 25°C) spectrum of **9** in hexanes after being exposed to 254-nm light for 10 min. Fig. S14. ^31^P{^1^H} NMR (162 MHz, hexanes, 25°C) spectrum of **9** in hexanes after being exposed to 254-nm light for 45 min. Fig. S15. Trap-to-trap distillation of **1**. Fig. S16. Labeling scheme for **1**. Fig. S17. ^1^H NMR (400 MHz, benzene-*d*_6_, 25°C) spectrum of **1**. Fig. S18. ^13^C{^1^H} NMR (101 MHz, benzene-*d*_6_, 25°C) spectrum of **1** and traces of pentane. Fig. S19. ^31^P{1H} NMR (162 MHz, benzene-*d*_6_, 25°C) spectrum of **1**. Fig. S20. ^1^H, ^13^C-HSQC NMR (400 MHz, benzene-*d*_6_, 25°C) spectrum of **1**. Fig. S21. ^1^H, ^13^C-HMBC NMR (400 MHz, benzene-*d*_6_, 25°C) spectrum of **1**. Fig. S22. Comparison of ^13^C{^1^H} NMR (125 MHz, benzene-*d*_6_, 25°C) and ^13^C{^1^H,^31^P} NMR (125 MHz, benzene-*d*_6_, 25°C) spectra of **1**. Fig. S23. Comparison of ^31^P{^1^H} NMR (202 MHz, benzene-*d*_6_, 25°C) and ^31^P{^1^H,^13^C} NMR (202 MHz, benzene-*d*_6_, 25°C) NMR spectra of **1**. Fig. S24. ^13^C, ^31^P-HSQC NMR (202 MHz, benzene-*d*_6_, 25°C) correlation experiment selective for one bond couplings in compound **1**. Fig. S25. ^13^C, ^31^P-HSQC NMR (202 MHz, benzene-*d*_6_, 25°C) correlation experiment selective for two bond couplings in compound **1**. Fig. S26. ^1^H NMR (400 MHz, benzene-*d*_6_, 25°C) spectrum of **1** before distillation. Fig. S27. ^31^P{^1^H} NMR (162 MHz, benzene-*d*_6_, 25°C) spectrum of crude **1** before distillation. Fig. S28. DART HRMS (Q-TOF) data corresponding to [C_15_H_28_P]^+^ and [C_15_H_27_]^+^. Fig. S29. Labeling scheme for natural abundance ^13^C satellites observed in ^31^P{^1^H} NMR spectra. Fig. S30. ^31^P{^1^H} NMR (162 MHz, benzene-*d*_6_, 25°C) spectrum of **1**. Fig. S31. ^31^P{^1^H} NMR (202 MHz, benzene-*d*_6_, 25°C) spectrum of **1**. Fig. S32. Experimental (black) and calculated (red) Raman spectrum of **1**. Fig. S33. Visualization of the totally symmetric breathing mode (a_1_), according to pseudo-C_3*v*_ symmetry, of **1**. Fig. S34. Labeling scheme for [(*^t^*BuC)_3_P(H)**A**][OTf]. Fig. S35. ^1^H NMR (500 MHz, THF-*d*_8_, 25°C) spectrum of [(*^t^*BuC)_3_P(H)**A**][OTf]. Fig. S36. ^19^F NMR (471 MHz, THF-*d*_8_, 25°C) spectrum of [(*^t^*BuC)_3_P(H)**A**][OTf]. Fig. S37. ^31^P{^1^H} NMR (202 MHz, THF-*d*_8_, 25°C) spectrum of [(*^t^*BuC)_3_P(H)**A**][OTf]. Fig. S38. ^31^P NMR (202 MHz, THF-*d*_8_, 25°C) spectrum of [(*^t^*BuC)_3_P(H)**A**][OTf]. Fig. S39. DART HRMS (Q-TOF) data corresponding to [C_15_H_27_]^+^ and [C_14_H_11_]^+^. Fig. S40. Initial ^31^P{^1^H} NMR (162 MHz, THF, 25°C) spectrum of [(*^t^*BuC)_3_P(H)**A**][OTf]. Fig. S41. ^31^P{^1^H} NMR (162 MHz, THF, 25°C) spectrum of [(*^t^*BuC)_3_P(H)**A**][OTf] after 16 hours. Fig. S42. Labeling scheme for **10**. Fig. S43. ^1^H NMR (400 MHz, benzene-*d*_6_, 25°C) spectrum of **10**. Fig. S44. ^19^F NMR (471 MHz, benzene-*d*_6_, 25°C) spectrum of **10**. Fig. S45. ^31^P{^1^H} NMR (162 MHz, benzene-*d*_6_, 25°C) spectrum of **10**. Fig. S46. ^31^P NMR (162 MHz, benzene-*d*_6_, 25°C) spectrum of **10**. Fig. S47. DART HRMS(Q-TOF) data corresponding to [C_15_H_27_]^+^ and [C_14_H_11_]^+^. Fig. S48. ^31^P{^1^H} NMR (162 MHz, benzene-*d*_6_, 25°C) spectrum of **10**. Fig. S49. ^31^P{^1^H} NMR (162 MHz, benzene-*d*_6_, 25°C) spectrum of **10** after 48 hours. Fig. S50. Labeling scheme for **11** and observed by-product. Fig. S51. Solvent supressed ^1^H NMR (400 MHz, THF, 25°C) spectrum of **11**. Fig. S52. ^31^P{^1^H} NMR (162 MHz, THF, 25°C) spectrum of **11**. Fig. S53. ^31^P NMR (162 MHz, THF, 25°C) spectrum of **11**. Fig. S54. ^31^P{^1^H} NMR (162 MHz, benzene-*d*_6_, 25°C) spectrum of **1** in benzene-*d*_6_ before air exposure. Fig. S55. ^31^P{^1^H} NMR (162 MHz, benzene-*d*_6_, 25°C) spectrum of **1** in benzene-*d*_6_ after being exposed to air for 30 min. Fig. S56. ^31^P{^1^H} NMR (162 MHz, benzene-*d*_6_, 25°C) spectrum of **1** in benzene-*d*_6_ after being exposed to air for 12 hours. Fig. S57. ^31^P{^1^H} NMR (162 MHz, benzene-*d*_6_, 25°C) spectrum of **1** in benzene-*d*_6_ before being heated. Fig. S58. ^31^P{^1^H} NMR (162 MHz, benzene-*d*_6_, 25°C) spectrum of **1** in benzene-*d*_6_ after being heated for 45 min at 75°C. Fig. S59. ^31^P{^1^H} NMR (162 MHz, toluene-*d*_8_, 25°C) spectrum of **1** in toluene-*d*_8_ before being heated. Fig. S60. ^31^P{^1^H} NMR (162 MHz, toluene-*d*_8_, 25°C) spectrum of **1** in toluene-*d*_8_ after being heated for 3 hours at 130°C. Fig. S61. ^31^P{^1^H} NMR (162 MHz, pentane, 25°C) spectrum of (*^t^*BuC)_3_P in pentane before being exposed to 254-nm light. Fig. S62. ^31^P{^1^H} NMR (162 MHz, pentane, 25°C) spectrum of (*^t^*BuC)_3_P in pentane after being exposed to 254-nm light for 5 min. Fig. S63. ^31^P{^1^H} NMR (162 MHz, THF, 25°C) spectrum of the crude reaction mixture, after treating (*^t^*BuC)_3_P with W(THF)(CO)_5_. Fig. S64. ^1^H NMR (400 MHz, benzene-*d*_6_, 25°C) spectrum of the crude reaction mixture, after treating (*^t^*BuC)_3_P with Ph_3_B and pyridine. Fig. S65. ^1^H NMR (400 MHz, benzene-*d*_6_, 25°C) spectrum of the crude reaction mixture, after treating (*^t^*BuC)_3_P with Ph_3_B and pyridine. Fig. S66. ^13^C{^1^H} NMR (101 MHz, benzene-*d*_6_, 25°C) spectrum of the crude reaction mixture, after treating (*^t^*BuC)_3_P with Ph_3_B and pyridine. Fig. S67. ^31^P{^1^H} NMR (101 MHz, benzene-*d*_6_, 25°C) spectrum of the crude reaction mixture, after treating (^t^BuC)_3_P with Ph_3_B and pyridine. Fig. S68. Molecular structure of Na[**8**], with thermal ellipsoids shown at the 50% probability level and hydrogen atoms omitted for clarity. Fig. S69. Molecular structure of **9**, with thermal ellipsoids shown at the 50% probability level and hydrogen atoms omitted for clarity. Fig. S70. Molecular structure of **1**, with thermal ellipsoids shown at the 50% probability level and hydrogen atoms omitted for clarity. Fig. S71. Crystals of **1** grown by sublimation. Table S1. Crystallographic data for Na[**8**]. Table S2. Bond lengths (Å) and angles (°) for Na[**8**]. Table S3. Crystallographic data for **9**. Table S4. Bond lengths (Å) and angles (°) for **9**. Table S5. Crystallographic data for **1**. Table S6. Bond lengths (Å) and angles (°) for **1**. Table S7. Coordinates of **1**. Table S8. Raman frequencies of **1**. Table S9. Initial coordinates of tetrahedrane. Table S10. Initial coordinates of white phosphorus. References (*38*–*47*)
