# Supplementary material for: Isolation of an elusive phosphatetrahedrane
Source: Sci Adv. 2020 Mar 25;6(13):eaaz3168. doi: 10.1126/sciadv.aaz3168 (PMC7096166; doi:10.1126/sciadv.aaz3168)
Supplement: aaz3168_SM.pdf [file aaz3168_SM.pdf]

Science Advances
AAAS

advances.sciencemag.org/cgi/content/full/6/13/eaaz3168/DC1

Supplementary Materials for

Isolation of an elusive phosphatetrahedrane

Martin-Louis Y. Riu, Rebecca L. Jones, Wesley J. Transue, Peter Müller, Christopher C. Cummins*

*Corresponding author. Email: ccummins@mit.edu

Published 25 March 2020, *Sci. Adv.* **6**, eaaz3168 (2020)
DOI: 10.1126/sciadv.aaz3168

This PDF file includes:

Section S1. Synthetic details and characterization of products
Section S2. X-ray diffraction studies
Section S3. Computational studies
Fig. S1. Labeling scheme for Na[**8**].
Fig. S2. ^{1}H NMR (400 MHz, THF-d_8, 25°C) spectrum of Na[**8**].
Fig. S3. ^{11}B{^{1}H} NMR (128 MHz, THF-d_8, 25°C) spectrum of Na[**8**].
Fig. S4. ^{13}C{^{1}H} NMR (101 MHz, THF-d_8, 25°C) spectrum of Na[**8**].
Fig. S5. ^{31}P{^{1}H} NMR (162 MHz, THF-d_8, 25°C) spectrum of Na[**8**].
Fig. S6. Labeling scheme for **9**.
Fig. S7. ^{1}H NMR (400 MHz, chloroform-*d*, 25°C) spectrum of **9**.
Fig. S8. ^{13}C{^{1}H} NMR (101 MHz, chloroform-*d*, 25°C) spectrum of **9**.
Fig. S9. ^{31}P{^{1}H} NMR (162 MHz, chloroform-*d*, 25°C) spectrum of **9**.
Fig. S10. ^{31}P{^{1}H} NMR (162 MHz, toluene-d_8, 25°C) spectrum after heating **9** to 110°C in toluene-d_8 for 22 hours.
Fig. S11. ^{1}H NMR (162 MHz, benzene-d_6, 25°C) spectrum after melting **9** at 130°C.
Fig. S12. ^{31}{^{1}H} NMR (162 MHz, benzene-d_6, 25°C) spectrum after melting **9** at 130°C.
Fig. S13. ^{31}P{^{1}H} NMR (162 MHz, hexanes, 25°C) spectrum of **9** in hexanes after being exposed to 254-nm light for 10 min.
Fig. S14. ^{31}P{^{1}H} NMR (162 MHz, hexanes, 25°C) spectrum of **9** in hexanes after being exposed to 254-nm light for 45 min.
Fig. S15. Trap-to-trap distillation of **1**.
Fig. S16. Labeling scheme for **1**.
Fig. S17. ^{1}H NMR (400 MHz, benzene-d_6, 25°C) spectrum of **1**.
Fig. S18. ^{13}C{^{1}H} NMR (101 MHz, benzene-d_6, 25°C) spectrum of **1** and traces of pentane.
Fig. S19. ^{31}P{1H} NMR (162 MHz, benzene-d_6, 25°C) spectrum of **1**.
Fig. S20. ^{1}H, ^{13}C-HSQC NMR (400 MHz, benzene-d_6, 25°C) spectrum of **1**.
Fig. S21. ^{1}H, ^{13}C-HMBC NMR (400 MHz, benzene-d_6, 25°C) spectrum of **1**.
Fig. S22. Comparison of ^{13}C{^{1}H} NMR (125 MHz, benzene-d_6, 25°C) and ^{13}C{^{1}H,^{31}P} NMR (125 MHz, benzene-d_6, 25°C) spectra of **1**.

Fig. S23. Comparison of $^{31}P\{^{1}H\}$ NMR (202 MHz, benzene-d_6, 25°C) and $^{31}P\{^{1}H,^{13}C\}$ NMR (202 MHz, benzene-d_6, 25°C) NMR spectra of **1**.
Fig. S24. ^{13}C, ^{31}P-HSQC NMR (202 MHz, benzene-d_6, 25°C) correlation experiment selective for one bond couplings in compound **1**.
Fig. S25. ^{13}C, ^{31}P-HSQC NMR (202 MHz, benzene-d_6, 25°C) correlation experiment selective for two bond couplings in compound **1**.
Fig. S26. ^{1}H NMR (400 MHz, benzene-d_6, 25°C) spectrum of **1** before distillation.
Fig. S27. $^{31}P\{^{1}H\}$ NMR (162 MHz, benzene-d_6, 25°C) spectrum of crude **1** before distillation.
Fig. S28. DART HRMS (Q-TOF) data corresponding to $[C_{15}H_{28}P]^+$ and $[C_{15}H_{27}]^+$.
Fig. S29. Labeling scheme for natural abundance ^{13}C satellites observed in $^{31}P\{^{1}H\}$ NMR spectra.
Fig. S30. $^{31}P\{^{1}H\}$ NMR (162 MHz, benzene-d_6, 25°C) spectrum of **1**.
Fig. S31. $^{31}P\{^{1}H\}$ NMR (202 MHz, benzene-d_6, 25°C) spectrum of **1**.
Fig. S32. Experimental (black) and calculated (red) Raman spectrum of **1**.
Fig. S33. Visualization of the totally symmetric breathing mode (a_1), according to pseudo-C_{3v} symmetry, of **1**.
Fig. S34. Labeling scheme for $[(^tBuC)_3P(H)$**A**$][OTf]$.
Fig. S35. ^{1}H NMR (500 MHz, THF-d_8, 25°C) spectrum of $[(^tBuC)_3P(H)$**A**$][OTf]$.
Fig. S36. ^{19}F NMR (471 MHz, THF-d_8, 25°C) spectrum of $[(^tBuC)_3P(H)$**A**$][OTf]$.
Fig. S37. $^{31}P\{^{1}H\}$ NMR (202 MHz, THF-d_8, 25°C) spectrum of $[(^tBuC)_3P(H)$**A**$][OTf]$.
Fig. S38. ^{31}P NMR (202 MHz, THF-d_8, 25°C) spectrum of $[(^tBuC)_3P(H)$**A**$][OTf]$.
Fig. S39. DART HRMS (Q-TOF) data corresponding to $[C_{15}H_{27}]^+$ and $[C_{14}H_{11}]^+$.
Fig. S40. Initial $^{31}P\{^{1}H\}$ NMR (162 MHz, THF, 25°C) spectrum of $[(^tBuC)_3P(H)$**A**$][OTf]$.
Fig. S41. $^{31}P\{^{1}H\}$ NMR (162 MHz, THF, 25°C) spectrum of $[(^tBuC)_3P(H)$**A**$][OTf]$ after 16 hours.
Fig. S42. Labeling scheme for **10**.
Fig. S43. ^{1}H NMR (400 MHz, benzene-d_6, 25°C) spectrum of **10**.
Fig. S44. ^{19}F NMR (471 MHz, benzene-d_6, 25°C) spectrum of **10**.
Fig. S45. $^{31}P\{^{1}H\}$ NMR (162 MHz, benzene-d_6, 25°C) spectrum of **10**.
Fig. S46. ^{31}P NMR (162 MHz, benzene-d_6, 25°C) spectrum of **10**.
Fig. S47. DART HRMS(Q-TOF) data corresponding to $[C_{15}H_{27}]^+$ and $[C_{14}H_{11}]^+$.
Fig. S48. $^{31}P\{^{1}H\}$ NMR (162 MHz, benzene-d_6, 25°C) spectrum of **10**.
Fig. S49. $^{31}P\{^{1}H\}$ NMR (162 MHz, benzene-d_6, 25°C) spectrum of **10** after 48 hours.
Fig. S50. Labeling scheme for **11** and observed by-product.
Fig. S51. Solvent supressed ^{1}H NMR (400 MHz, THF, 25°C) spectrum of **11**.
Fig. S52. $^{31}P\{^{1}H\}$ NMR (162 MHz, THF, 25°C) spectrum of **11**.
Fig. S53. ^{31}P NMR (162 MHz, THF, 25°C) spectrum of **11**.
Fig. S54. $^{31}P\{^{1}H\}$ NMR (162 MHz, benzene-d_6, 25°C) spectrum of **1** in benzene-d_6 before air exposure.
Fig. S55. $^{31}P\{^{1}H\}$ NMR (162 MHz, benzene-d_6, 25°C) spectrum of **1** in benzene-d_6 after being exposed to air for 30 min.
Fig. S56. $^{31}P\{^{1}H\}$ NMR (162 MHz, benzene-d_6, 25°C) spectrum of **1** in benzene-d_6 after being exposed to air for 12 hours.
Fig. S57. $^{31}P\{^{1}H\}$ NMR (162 MHz, benzene-d_6, 25°C) spectrum of **1** in benzene-d_6 before being heated.
Fig. S58. $^{31}P\{^{1}H\}$ NMR (162 MHz, benzene-d_6, 25°C) spectrum of **1** in benzene-d_6 after being heated for 45 min at 75°C.
Fig. S59. $^{31}P\{^{1}H\}$ NMR (162 MHz, toluene-d_8, 25°C) spectrum of **1** in toluene-d_8 before being heated.

Fig. S60. $^{31}P\{^1H\}$ NMR (162 MHz, toluene-d_8, 25°C) spectrum of **1** in toluene-d_8 after being heated for 3 hours at 130°C.
Fig. S61. $^{31}P\{^1H\}$ NMR (162 MHz, pentane, 25°C) spectrum of (^{t}BuC)$_3$P in pentane before being exposed to 254-nm light.
Fig. S62. $^{31}P\{^1H\}$ NMR (162 MHz, pentane, 25°C) spectrum of (^{t}BuC)$_3$P in pentane after being exposed to 254-nm light for 5 min.
Fig. S63. $^{31}P\{^1H\}$ NMR (162 MHz, THF, 25°C) spectrum of the crude reaction mixture, after treating (^{t}BuC)$_3$P with W(THF)(CO)$_5$.
Fig. S64. ^1H NMR (400 MHz, benzene-d_6, 25°C) spectrum of the crude reaction mixture, after treating (^{t}BuC)$_3$P with Ph_3B and pyridine.
Fig. S65. ^1H NMR (400 MHz, benzene-d_6, 25°C) spectrum of the crude reaction mixture, after treating (^{t}BuC)$_3$P with Ph_3B and pyridine.
Fig. S66. $^{13}C\{^1H\}$ NMR (101 MHz, benzene-d_6, 25°C) spectrum of the crude reaction mixture, after treating (^{t}BuC)$_3$P with Ph_3B and pyridine.
Fig. S67. $^{31}P\{^1H\}$ NMR (101 MHz, benzene-d_6, 25°C) spectrum of the crude reaction mixture, after treating (^{t}BuC)$_3$P with Ph_3B and pyridine.
Fig. S68. Molecular structure of Na[**8**], with thermal ellipsoids shown at the 50% probability level and hydrogen atoms omitted for clarity.
Fig. S69. Molecular structure of **9**, with thermal ellipsoids shown at the 50% probability level and hydrogen atoms omitted for clarity.
Fig. S70. Molecular structure of **1**, with thermal ellipsoids shown at the 50% probability level and hydrogen atoms omitted for clarity.
Fig. S71. Crystals of **1** grown by sublimation.
Table S1. Crystallographic data for Na[**8**].
Table S2. Bond lengths (Å) and angles (°) for Na[**8**].
Table S3. Crystallographic data for **9**.
Table S4. Bond lengths (Å) and angles (°) for **9**.
Table S5. Crystallographic data for **1**.
Table S6. Bond lengths (Å) and angles (°) for **1**.
Table S7. Coordinates of **1**.
Table S8. Raman frequencies of **1**.
Table S9. Initial coordinates of tetrahedrane.
Table S10. Initial coordinates of white phosphorus.
References (*38–47*)

Supplementary Materials

Section S1. Synthetic details and characterization of products

S1.1 General methods

Except as otherwise noted, all manipulations were performed in a Vacuum Atmospheres model MO-40M glovebox under an inert atmosphere of purified N_2. All solvents were obtained anhydrous and oxygen-free by bubble degassing (Ar) and purification through columns of alumina using a solvent purification system (Pure Process Technology, Nashua, NH) (32) and storage over 4.0 Å molecular sieves (33). Deuterated solvents were purchased from Cambridge Isotope Labs, then degassed and stored over molecular sieves for at least 48 h prior to use. Celite® (EM Science), 4.0 Å molecular sieves, silica, acidic alumina, and charcoal were dried by heating above 200 °C under dynamic vacuum (50 mTorr) for at least 48 h prior to use. All glassware was dried in an oven for at least two hours at temperatures greater than 150 °C.

Tri-*tert*-butyl cyclopropenyl tetrafluoroborate (18) and HP**A** (**7**, **A** = 9,10-dihydroanthracene-9,10-diyl) (17) were prepared according to literature procedures. Triphenylborane (Strem Chemicals), triphenylphosphine (Sigma-Aldrich), sodium bis(trimethylsilyl)amide (Sigma-Aldrich), lithium 2,2,6,6-tetramethylpiperidide (Sigma-Aldrich), trifluoromethanesulfonic acid (Strem Chemicals), and tungsten hexacarbonyl (Strem Chemicals) were used as received. Lithium 2,2,6,6-tetramethylpiperidide was also prepared according to a literature procedure (34). Pyridine (Sigma-Aldrich) was distilled under air, degassed three times by the freeze-pump-thaw method, and stored over 4 Å molecular sieves for 48 h prior to use. Tetramethylammonium fluoride (Sigma-Aldrich) was dried at 100 °C under reduced pressure (50 mTorr) for 48 h prior to use. Tetrabutylammonium chloride (Sigma-Aldrich) was dried at 60 °C under reduced pressure (50 mTorr) for 48 h and crystallized from acetonitrile/pentane prior to use.

NMR spectra were obtained on a Jeol ECZ-500 instrument equipped with an Oxford Instruments superconducting magnet, on a Bruker Avance 400 instrument equipped with a Magnex

Scientific or with a SpectroSpin superconducting magnet, or on a Bruker Avance 500 instrument equipped with a Magnex Scientific or with a SpectroSpin superconducting magnet. ^{1}H and ^{13}C NMR spectra were referenced internally to residual solvent signals (35). ^{31}P NMR spectra were externally referenced to 85% H_3PO_4 (0 ppm). ^{11}B NMR spectra were externally referenced to BF_3-OEt_2 (0 ppm). ^{19}F NMR spectra were externally referenced to $CFCl_3$ (0 ppm). Elemental combustion analyses were performed by Midwest Micro Laboratories (Indianapolis, IN, USA).

High resolution mass spectral (HRMS) data were collected using a Jeol AccuTOF 4G LC-Plus mass spectrometer equipped with an Ion-Sense DART source. Data were calibrated to a sample of PEG-600 and were collected in positive-ion mode. Samples were prepared in THF (10 μM concentration) and were briefly exposed to air ($<$5 s) before being placed in front of the DART source.

Photochemical reactions were performed using a Rayonet photochemical reactor RPR-200 (Southern New England Ultra Violet Company) loaded with 16 RPR2537A lamps, each emitting ca. 35 W at 253.7 nm.

Raman spectra were collected using a Renishaw Invia Reflex Micro Raman (see section S1.7.2).

S1.2 Synthesis of [Na(OEt_2)$_2$][Ph_3BPA] (Na[8])

Aluminum foil was used to limit exposure to ambient light during this experiment. A 250 mL flask charged with a solution of HP**A** (**7**, 0.850 g, 4.04 mmol, 1.00 equiv) and triphenylborane (1.10 g, 4.55 mmol, 1.13 equiv) in diethyl ether (60 mL) was frozen in the liquid nitrogen cooled coldwell of the glovebox. Separately, a solution of sodium bis(trimethylsilyl)amide (0.834 g, 4.55 mmol, 1.13 equiv) in diethyl ether (10 mL) was prepared and frozen in the coldwell of the glovebox. Upon thawing, the sodium bis(trimethylsilyl)amide solution was added rapidly to the thawing solution of **7**. The solution became white and heterogeneous as it warmed with

rapid stirring. After 20 min, the colorless precipitate was collected by vacuum filtration and was washed with Et_2O (2 × 10 mL). This afforded colorless powder of Na[**8**] (2.09 g, 3.34 mmol, 83%). The number of Et_2O molecules in the formula has been determined by X-ray crystallography as well as by integration of ^1H NMR signals. Melting point: 50-55 °C. This sensitive material has not passed elemental analysis, being reproducibly low in carbon. Attempts to purify this compound by crystallization in minimal Et_2O consistently results in the consumption of Na[**8**] and the formation of anthracene together with unidentified products. Elem. Anal. Found(Calc'd) for $C_{40}H_{45}BNaO_2P$ from two separate batch preparations, C, 75.16(77.17); H, 6.61(7.29); N, <0.02(0.00) and C, 75.59(77.17); H, 7.05(7.29); N, <0.02(0.00). ^1H NMR (400 MHz, THF-d_8, 25 °C, Fig. S2) δ 7.21 (d, J = 7.3 Hz, 6H), 6.98−6.91 (m, 2H), 6.85 (t, J = 7.3 Hz, 6H), 6.72 (t, J = 7.1 Hz, 3H), 6.66−6.62 (m, 2H), 6.62−6.58 (m, 2H), 6.24−6.20 (m, 2H), 3.86 (d, $^2J_{PH}$ = 13.2 Hz, 2H), 3.39 (q, J = 7.0 Hz, 4H), 1.12 (t, J = 7.0 Hz, 6H) ppm. $^{11}B\{^1H\}$ NMR (128 MHz, THF-d_8, 25 °C, Fig. S3) δ −6.47 (br s) ppm. $^{13}C\{^1H\}$ NMR (101 MHz, THF-d_8, 25 °C, Fig. S4) δ 154.76 (d, J = 12.3 Hz), 151.34, 136.25 (d, J = 8.6 Hz), 126.44, 123.67, 123.28, 122.69, 121.93, 120.51 (d, J = 6.4 Hz), 66.49, 54.66 (d, $^1J_{PC}$ = 17.3 Hz), 15.85 ppm. $^{31}P\{^1H\}$ NMR (162 MHz, THF-d_8, 25 °C, Fig. S5) δ 272.13 (br s) ppm.

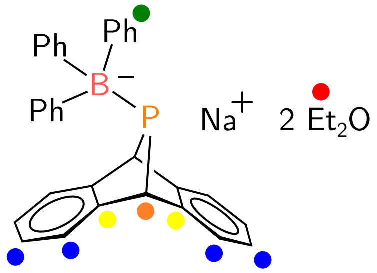

Fig. S1. Labeling scheme for Na[8].

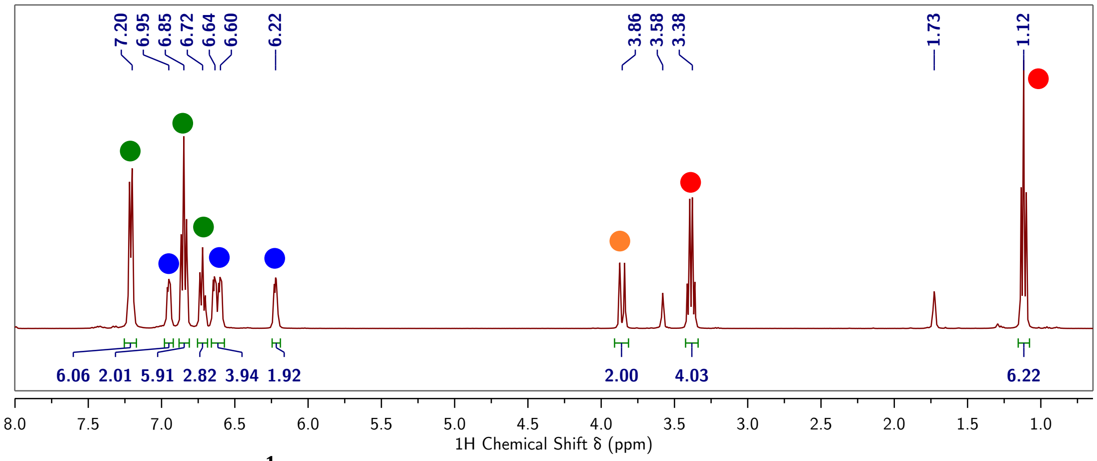

Fig. S2. ^{1}H NMR (400 MHz, THF-d_8, 25°C) spectrum of Na[8].

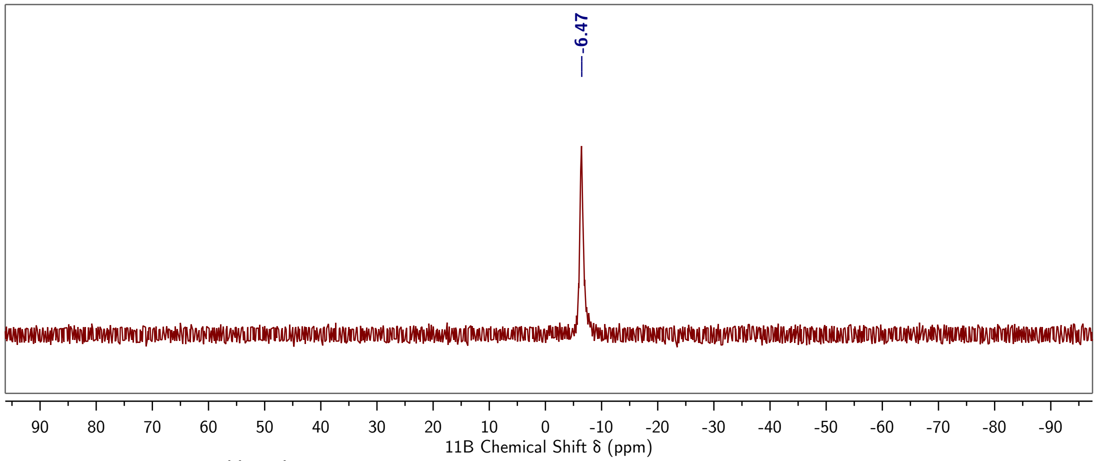

Fig. S3. ^{11}B{^{1}H} NMR (128 MHz, THF-d_8, 25°C) spectrum of Na[8].

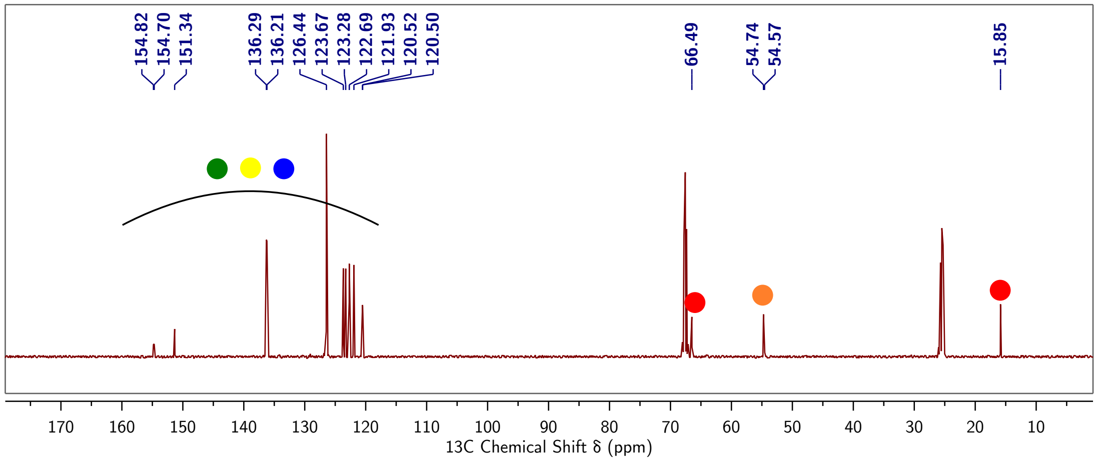

Fig. S4. ^{13}C{^{1}H} NMR (101 MHz, THF-d_8, 25°C) spectrum of Na[8].

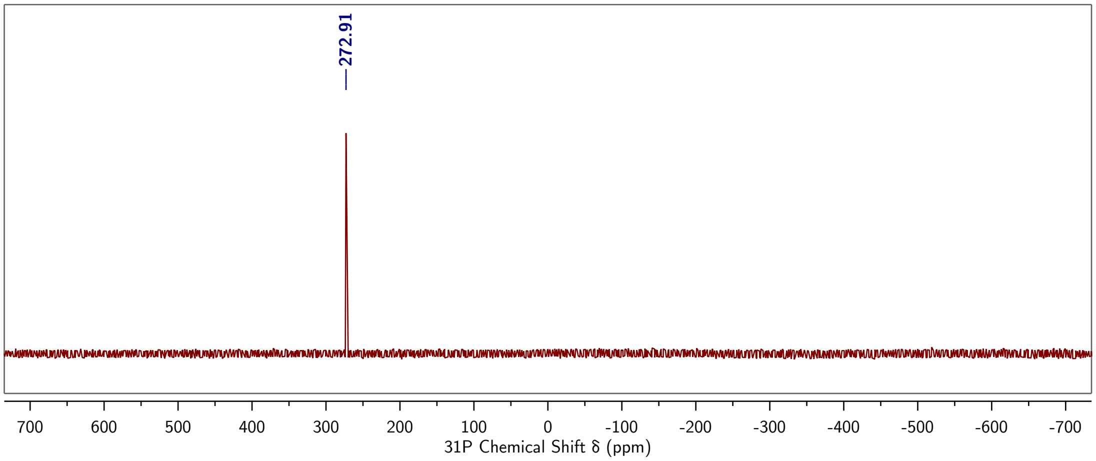

Fig. S5. $^{31}P\{^1H\}$ NMR (162 MHz, THF-d_8, 25°C) spectrum of Na[8].

S1.3 Synthesis of $(^t\text{BuC})_3\text{PA}$ (9)

Aluminum foil was used to limit exposure of the reaction mixture to ambient light during this experiment. A 100 mL flask charged with a solution of Na[**8**] (1.00 g, 1.61 mmol, 1.00 equiv) in THF (10 mL) and a Teflon®-coated magnetic stir bar was frozen in the liquid nitrogen cooled coldwell of the glovebox. Separately, a solution of tri-*tert*-butyl cyclopropenyl tetrafluoroborate (18) (0.473 g, 1.61 mmol, 1.00 equiv) in THF (20 mL) was prepared and frozen in the coldwell of the glovebox. Upon thawing, the solution of tri-*tert*-butyl cyclopropenyl tetrafluoroborate was rapidly added to the thawing solution of Na[**8**]. The solution became cloudy as it warmed with rapid stirring. After 1 h, the solution was filtered through a coarse sintered frit (15 mL) containing a one-inch plug of Celite®. All volatile materials were removed *in vacuo* and the resulting white solids were taken up in hexanes (16 mL). Pyridine (*ca.* 12 drops) was added to the solution, causing precipitation of the triphenylborane adduct of pyridine as a colorless solid (36). The reaction mixture was filtered through a coarse sintered frit (15 mL) containing a two-inch plug of charcoal and the plug was washed with hexanes (15 mL). All volatile materials were removed *in vacuo* from the combined filtrates yielding colorless solids. Crystallization from minimal pentane at -35 °C provided colorless crystals of **9** (576 mg, 1.38 mmol, 86%). Melting point 127-130 °C. While **9** was not observed by DART HRMS, anthracene ($[M+H]^+$ Calcd for $C_{14}H_{10}$ 179.0846; Found 179.0861) and $[^t\text{Bu}_3\text{C}_3]^+$ ($[M]^+$ Calcd for $C_{15}H_{27}$ 207.2113; Found 207.2129) were observed. This material has not passed elemental analysis, being reproducibly low in carbon. Elem. Anal. Found(Calc'd) for $C_{29}H_{37}P$ from two separate batch preparations, C, 81.84(83.61); H, 9.44 (8.70); N, <0.02(0.00) and 80.59(83.61); H, 8.95 (8.70); N, <0.02(0.00). ^{1}H NMR (400 MHz, chloroform-d, 25 °C, Fig. S7) δ 7.27 (ddd, J = 5.1, 3.2, 1.3 Hz, 2H), 7.22 (dd, J = 5.3, 3.1 Hz, 2H), 6.99 (dd, J = 5.4, 3.1 Hz, 2H), 6.88 (dd, J = 5.3, 3.1 Hz, 2H), 4.21 (d, $^2J_{\text{PH}}$ = 13.4 Hz, 2H), 1.12 (s, 18H), 0.92 (s, 9H) ppm. ^{13}C{^{1}H} NMR (101 MHz, chloroform-d, 25 °C, Fig. S8) δ 148.91 (d, J = 1.9 Hz), 147.40 (d, J = 20.4

Hz), 127.72 (d, J = 4.4 Hz), 125.52, 124.82, 123.93, 122.28 (d, J = 3.0 Hz), 54.44 (d, $^{1}J_{PC}$ = 23.4 Hz), 45.82 (d, J = 59.4 Hz), 37.55 (d, J = 19.5 Hz), 31.61, 31.25 (d, J = 2.3 Hz), 30.40 (d, J = 4.9 Hz) ppm. $^{31}P\{^{1}H\}$ NMR (162 MHz, chloroform-d, 25 °C, Fig. S9) δ 199.4 (t, $^{2}J_{PH}$= 13.4 Hz) ppm.

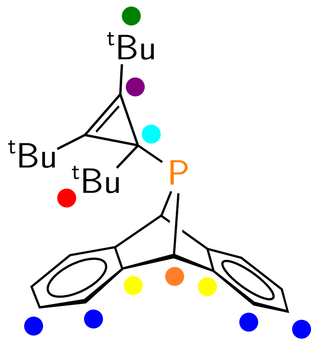

Fig. S6. Labeling scheme for 9.

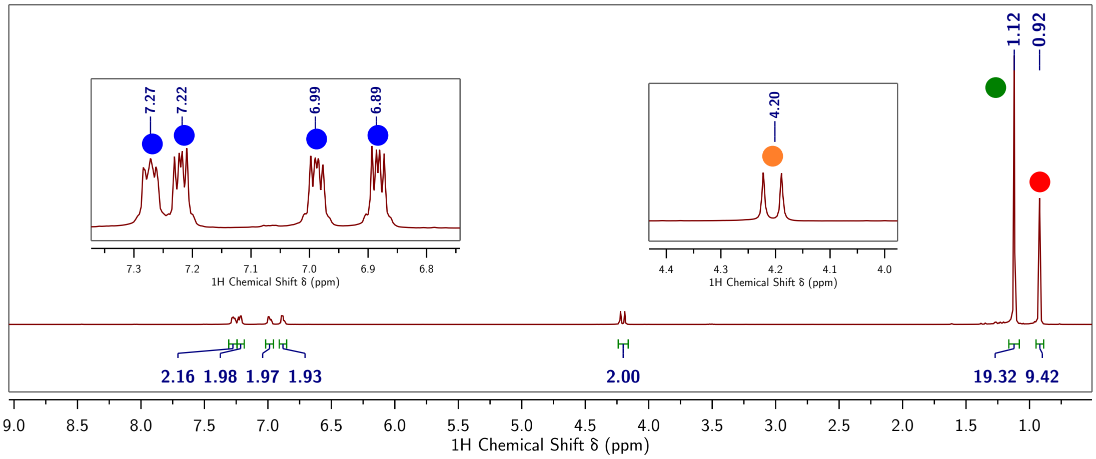

Fig. S7. ^{1}H NMR (400 MHz, chloroform-*d*, 25°C) spectrum of 9.

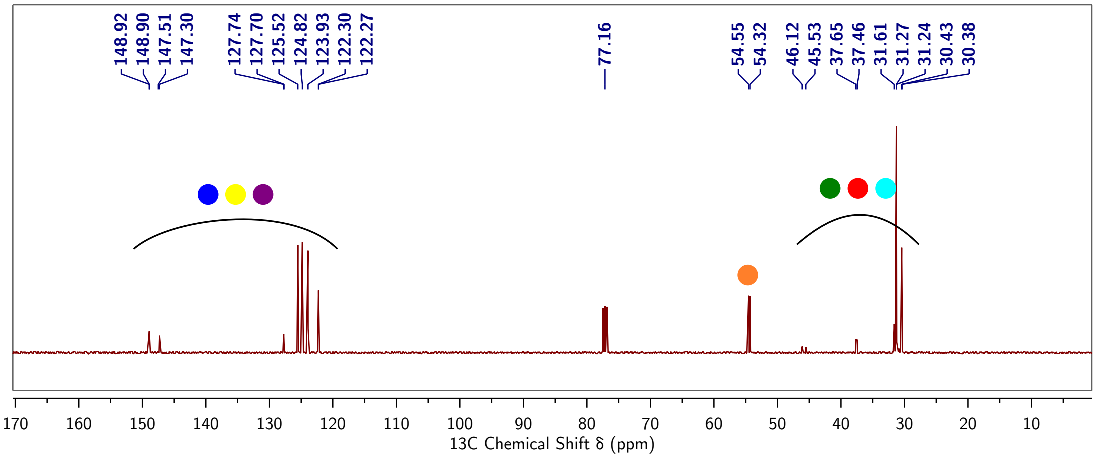

Fig. S8. $^{13}C\{^{1}H\}$ **NMR (101 MHz, chloroform-*d*, 25°C) spectrum of 9.**

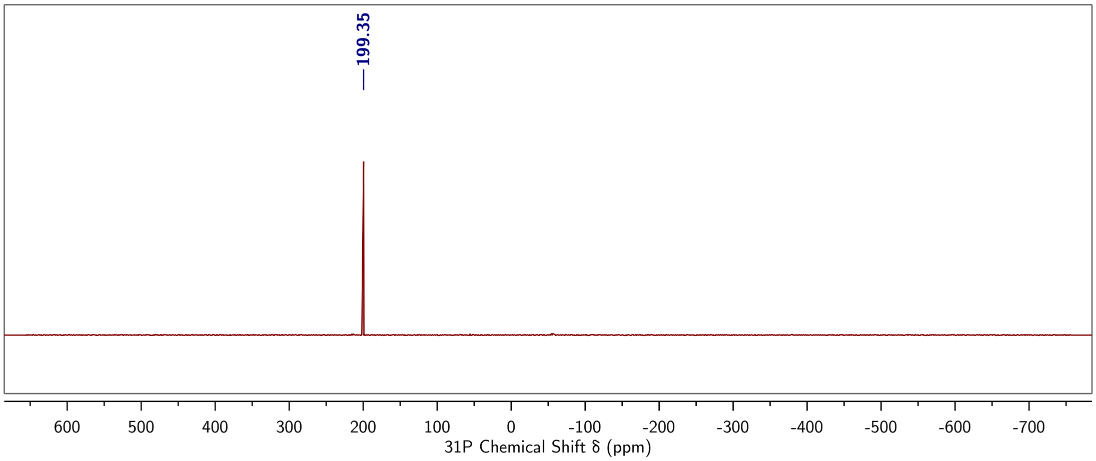

Fig. S9. $^{31}P\{^{1}H\}$ **NMR (162 MHz, chloroform-*d*, 25°C) spectrum of 9.**

S1.4 Thermolysis of (^{t}BuC)$_3$PA (9)

A 20 mM solution of (^{t}BuC)$_3$PA (**9**) in toluene-d_8 was prepared and was transferred to a J. Young tube. The tube was placed in a preheated (110 °C) oil bath for 22 h. Compound **9** was largely unchanged after the heating process and only a trace of (^{t}BuC)$_3$P (**1**) was observed (Fig. S10).

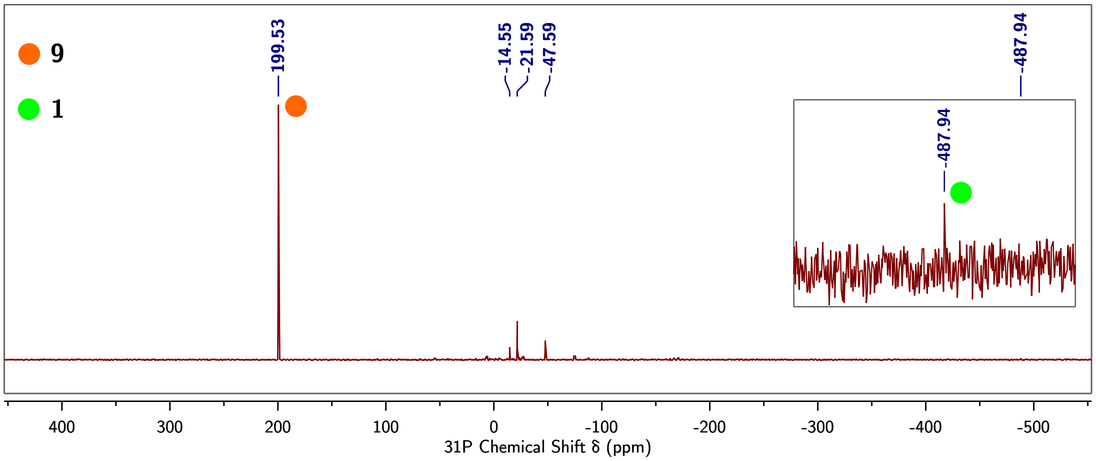

Fig. S10. ^{31}P{^{1}H} NMR (162 MHz, toluene-d_8, 25°C) spectrum after heating 9 to 110°C in toluene-d_8 for 22 hours.

S1.5 Characterization of melted $(^tBuC)_3PA$ (9)

$(^tBuC)_3PA$ (**9**, 2 mg) was loaded into a flame sealed glass capillary and was melted at 130 °C. No discoloration of the material was observed. The melted material was extracted from the capillary with benzene-d_6 (0.7 mL) and the homogeneous solution was transferred to an NMR tube. Compound **9** was unchanged after the melting process (Fig. S11 and Fig. S12).

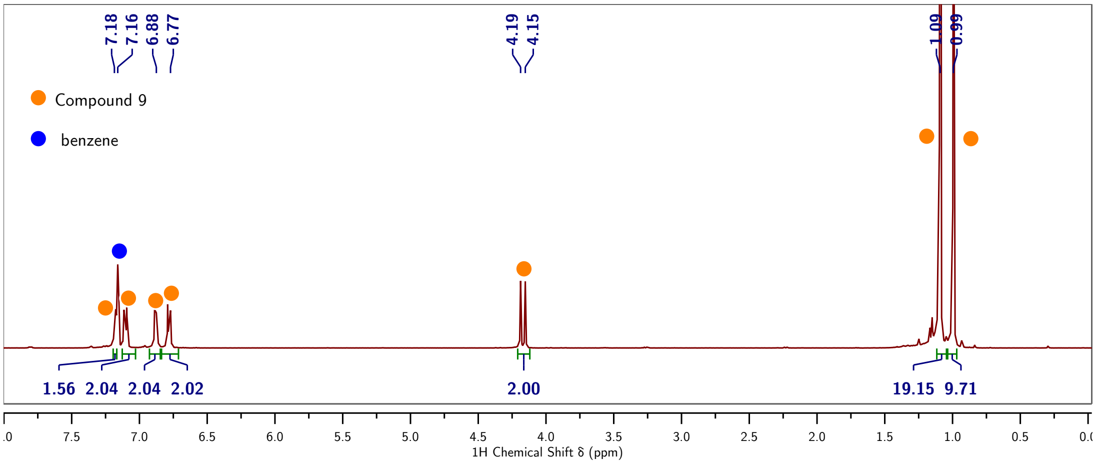

Fig. S11. ^{1}H NMR (162 MHz, benzene-d_6, 25°C) spectrum after melting 9 at 130°C.

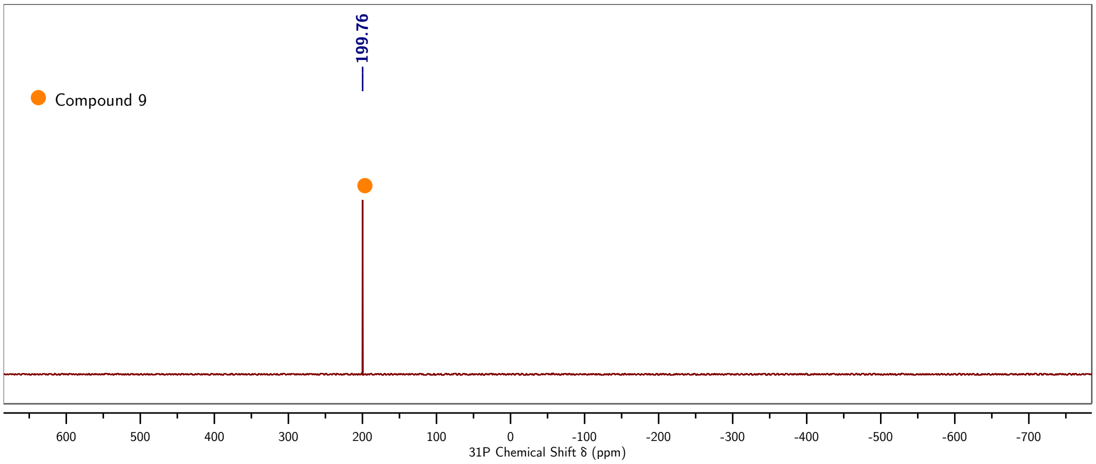

Fig. S12. $^{31}\{^1H\}$ NMR (162 MHz, benzene-d_6, 25°C) spectrum after melting 9 at 130°C.

S1.6 Photolysis of (^{t}BuC)$_3$PA (9)

A 20 mM solution of (^{t}BuC)$_3$PA (**9**) in hexanes was prepared and transferred to an NMR tube. The solution was irradiated with 254 nm light. ^{31}P{^{1}H} NMR spectra were collected after 10 min and 45 min. While traces of (^{t}BuC)$_3$P (**1**) were observed after 10 min of irradiation (Fig. S13), this signal disappeared after 45 min of irradiation (Fig. S14). Photolysis also generates a new ^{31}P NMR signal at −47.96 ppm, which we tentatively assign to a [2+2] dimer of tri-*tert*-butyl phosphacyclobutadiene (refer to section S1.13 for more information).

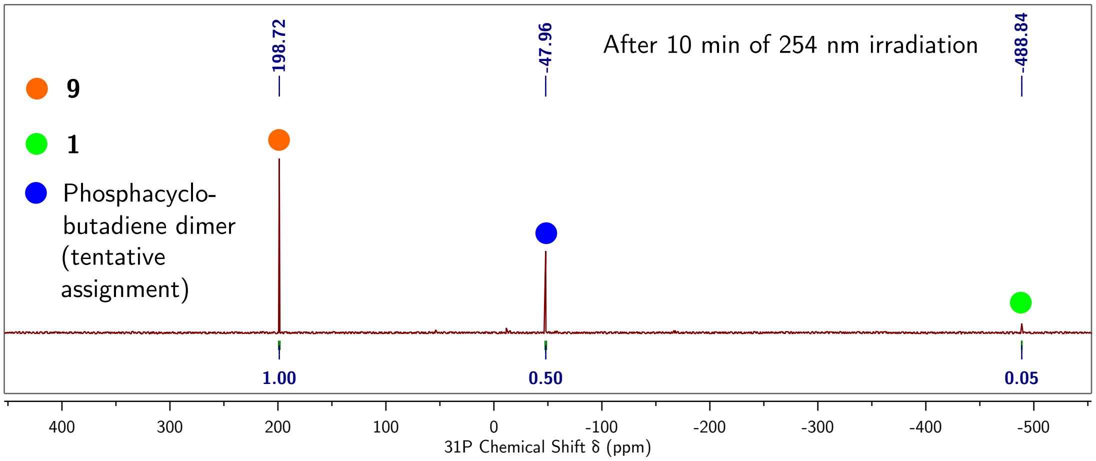

Fig. S13. ^{31}**P{**^{1}**H} NMR (162 MHz, hexanes, 25°C) spectrum of 9 in hexanes after being exposed to 254-nm light for 10 min.**

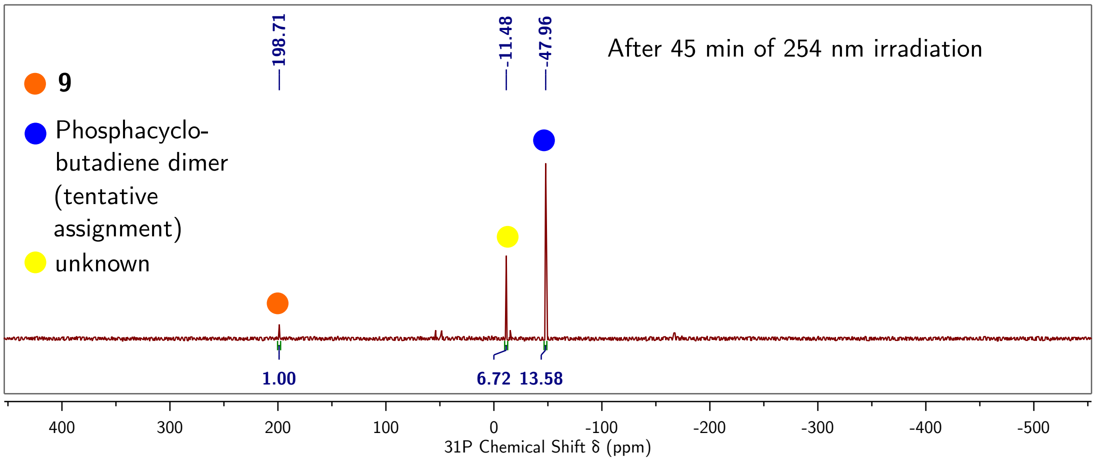

Fig. S14. $^{31}P\{^1H\}$ **NMR (162 MHz, hexanes, 25°C) spectrum of 9 in hexanes after being exposed to 254-nm light for 45 min.**

S1.7 Synthesis of $(^t\mathrm{BuC})_3\mathrm{P}$ (1)

To a 20 mL scintillation vial charged with a solution of $(^t\mathrm{BuC})_3\mathrm{P}$**A** (**9**, 0.300 g, 0.719 mmol, 1.00 equiv) in THF (2 mL) and a Teflon®-coated magnetic stir bar, was added a solution of trifluoromethanesulfonic acid (0.108 g, 0.719 mmol, 1.00 equiv) in THF (2 mL). After 20 min, a slurry of tetramethylammonium fluoride (0.067 g, 0.719 mmol, 1.00 equiv) in THF (1 mL) was added dropwise. After the addition, the colorless heterogeneous solution was stirred for 30 min. All volatile materials were then removed *in vacuo* from the solution, resulting in a colorless residue. This material was slurried in pentane (5 mL) and the solution was filtered through a coarse sintered frit (15 mL) containing a one-inch plug of charcoal. The plug was washed with pentane (10 mL). All volatile materials were then removed *in vacuo* from the combined filtrates, resulting in a colorless oil. This oil was taken up in THF (2 mL) to give a solution of $(^t\mathrm{BuC})_3\mathrm{P(F)H}$ (**10**) that was frozen in the liquid nitrogen cooled coldwell of the glovebox. Separately, a solution of lithium tetramethylpiperidide (0.106 g, 0.719 mmol, 1.00 equiv) in THF (2 mL) was prepared and frozen in the liquid nitrogen cooled coldwell of the glovebox. Upon thawing, the lithium tetramethylpiperidide solution was added dropwise to the thawing solution of **10**. After 20 min, all volatile materials were removed *in vacuo*, yielding colorless solids. The solids were taken up in pentane (2 mL) and the solution was filtered through a coarse sintered frit (15 mL) containing a one-inch plug of acidic alumina. The plug was subsequently washed with pentane (1 mL). Under reduced pressure, all volatile materials were removed from the combined filtrates, yielding a pale yellow oil (87 mg, see Fig. S26 and Fig. S27 for NMR characterization). This oil was transferred to a Teflon®-sealed trap-to-trap distillation apparatus, which was removed from the glovebox, connected to a Schlenk line, and placed under static vacuum (50 mTorr). One trap was kept at 23 °C by using an oil bath, while the other trap was cooled to −78 °C by using a mixture of dry ice and acetone (see Fig. S15). After 2 h, colorless oil collected in the −78 °C trap while a yellow gel formed in the 23 °C trap.

The apparatus was removed from the two baths, backfilled with nitrogen, and brought into the glovebox. The colorless oil was taken up in pentane (0.5 mL) and the solution was filtered through a glass fiber filter paper plugged pipette containing a two-inch plug of silica. The plug was subsequently washed with pentane (1.5 mL). All volatile materials were removed from the combined filtrates under reduced pressure, yielding colorless solids (33 mg, 0.138 mmol, 19%). Melting point 31-34 °C. DART HRMS(Q-TOF) m/z: $[M+H]^+$ Calcd for $C_{15}H_{28}P$ 239.1929; Found 239.1931 (Fig. S28). Elem. Anal. Found(Calc'd) for $C_{15}H_{27}P$, C, 73.95(75.59); H, 11.32 (11.42); N, <0.02(0.00). ^{1}H NMR (400 MHz, benzene-d_6, 25 °C, Fig. S17) δ 1.17 (s, 27H) ppm. ^{13}C{^{1}H} NMR (101 MHz, benzene-d_6, 25 °C, Fig. S18) δ 31.02, 27.62 (d, $^2J_{PC}$ = 6.3 Hz), 25.22 (d, $^1J_{PC}$ = 37.9 Hz) ppm. ^{31}P{^{1}H} NMR (162 MHz, benzene-d_6, 25 °C, Fig. S19) δ −487.98 ppm.

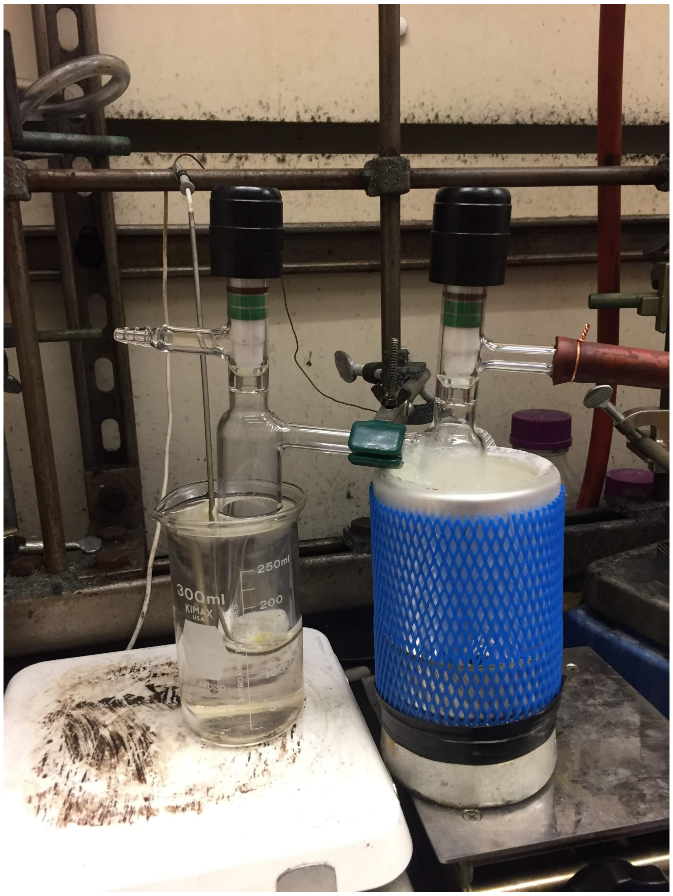

Fig. S15. Trap-to-trap distillation of 1. (Photo Credit: Martin-Louis Y. Riu, MIT)

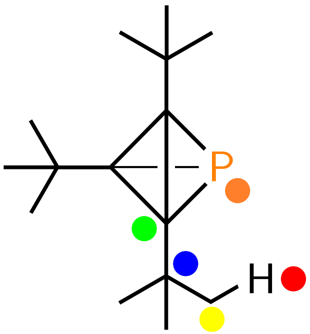

Fig. S16. Labeling scheme for 1.

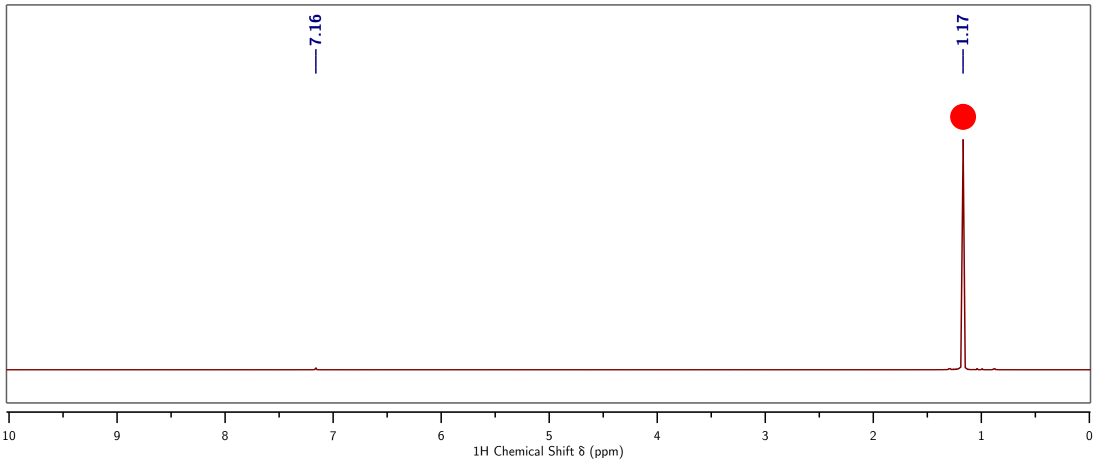

Fig. S17. ^{1}**H NMR (400 MHz, benzene-**d_6**, 25°C) spectrum of 1.**

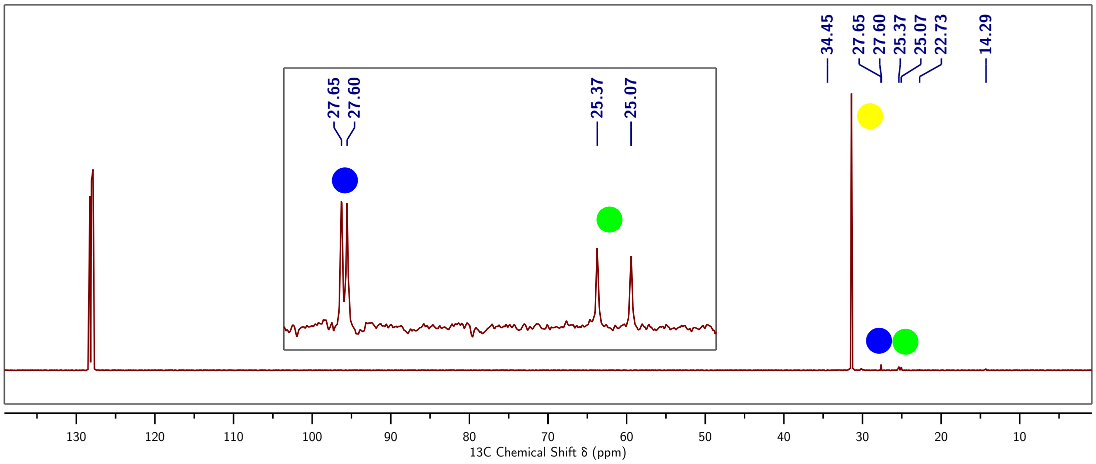

Fig. S18. ^{13}**C{**^{1}**H} NMR (101 MHz, benzene-**d_6**, 25°C) spectrum of 1 and traces of pentane.**

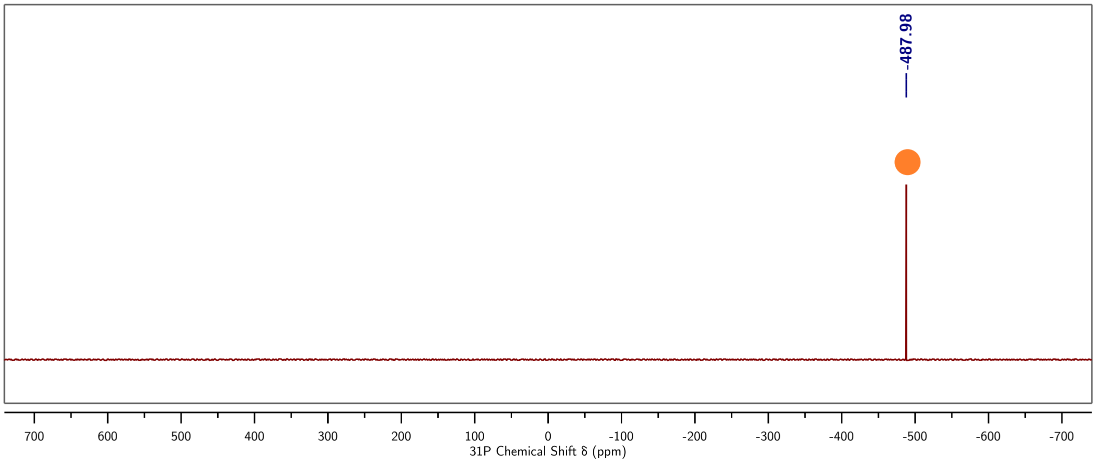

Fig. S19. ^{31}**P{1H} NMR (162 MHz, benzene-**d_6**, 25°C) spectrum of 1.**

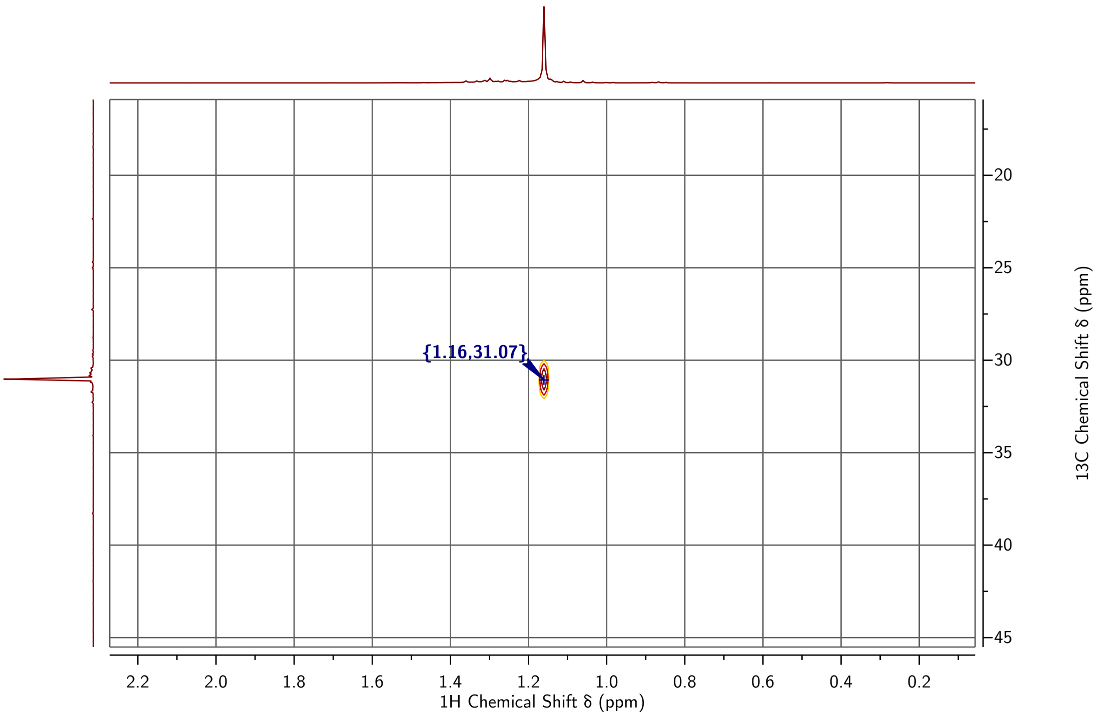

Fig. S20. **^{1}H, ^{13}C-HSQC NMR (400 MHz, benzene-d_6, 25°C) spectrum of 1.**

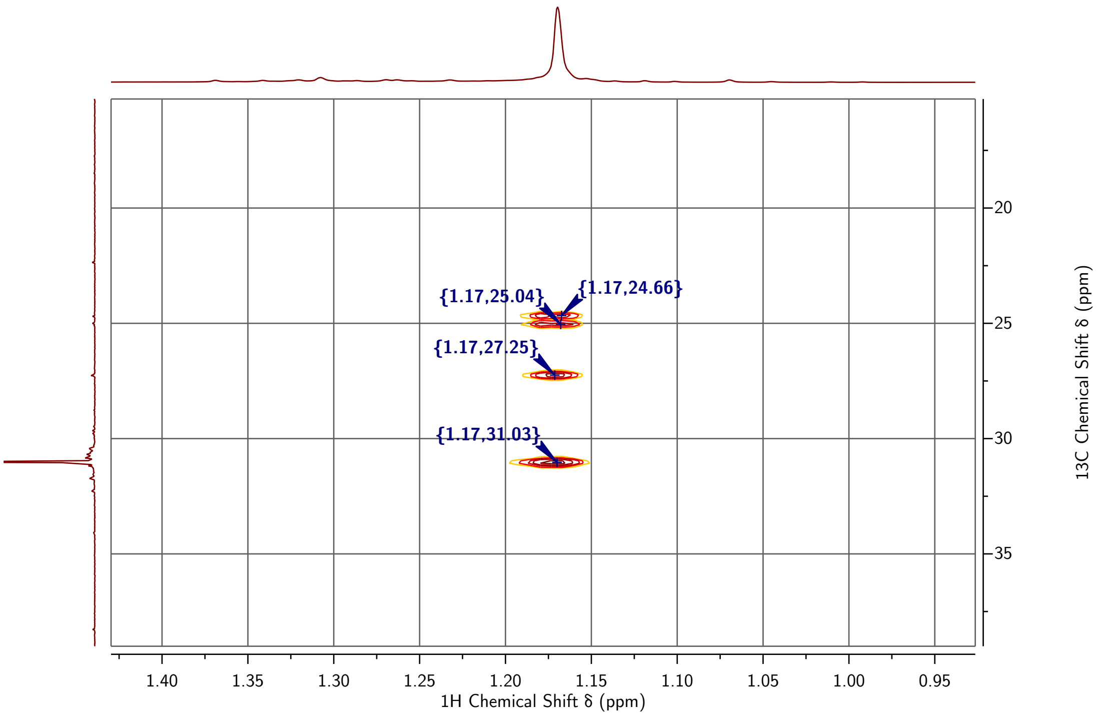

Fig. S21. **^{1}H, ^{13}C-HMBC NMR (400 MHz, benzene-d_6, 25°C) spectrum of 1.**

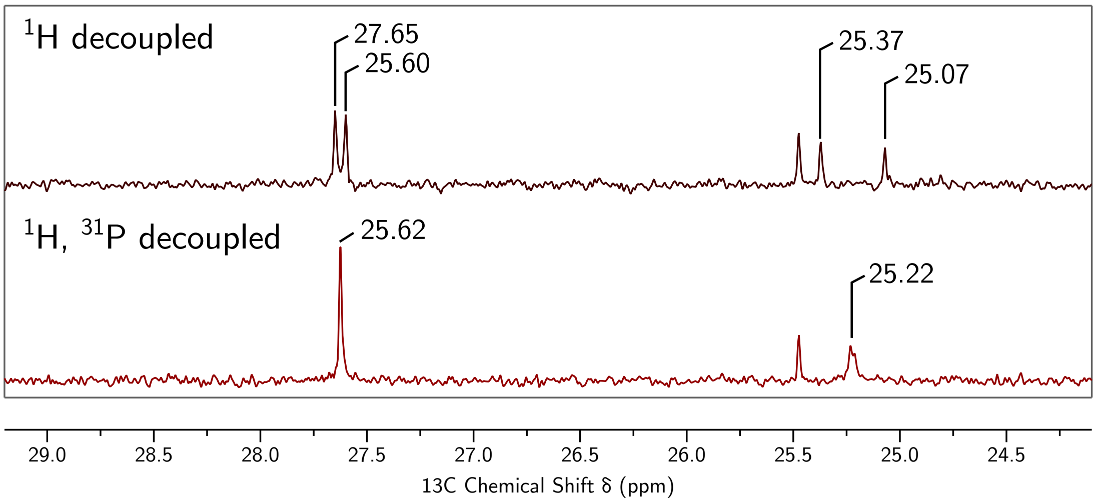

Fig. S22. Comparison of $^{13}C\{^1H\}$ NMR (125 MHz, benzene-d_6, 25°C) and $^{13}C\{^1H,^{31}P\}$ NMR (125 MHz, benzene-d_6, 25°C) spectra of 1.

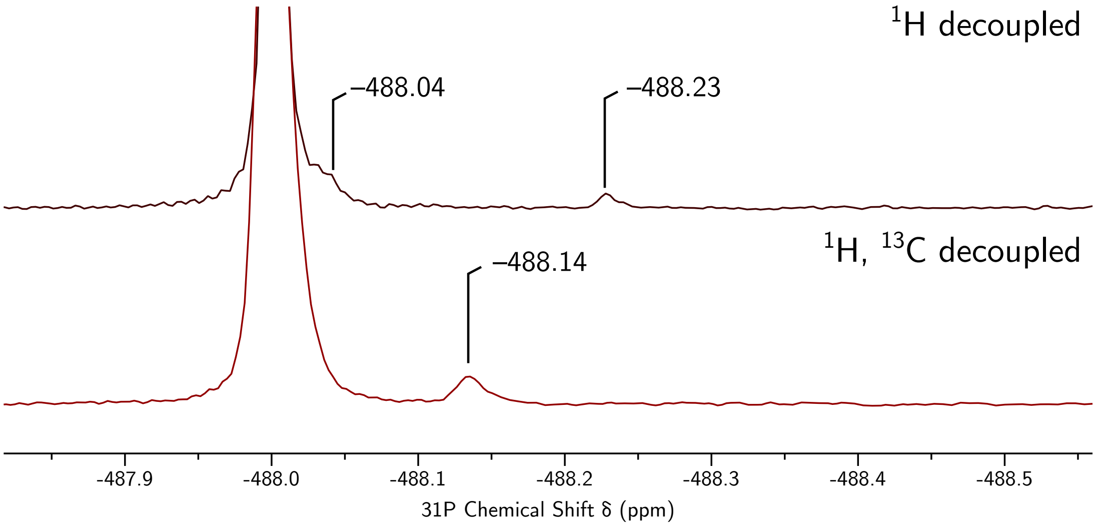

Fig. S23. Comparison of $^{31}P\{^1H\}$ NMR (202 MHz, benzene-d_6, 25°C) and $^{31}P\{^1H,^{13}C\}$ NMR (202 MHz, benzene-d_6, 25°C) NMR spectra of 1.

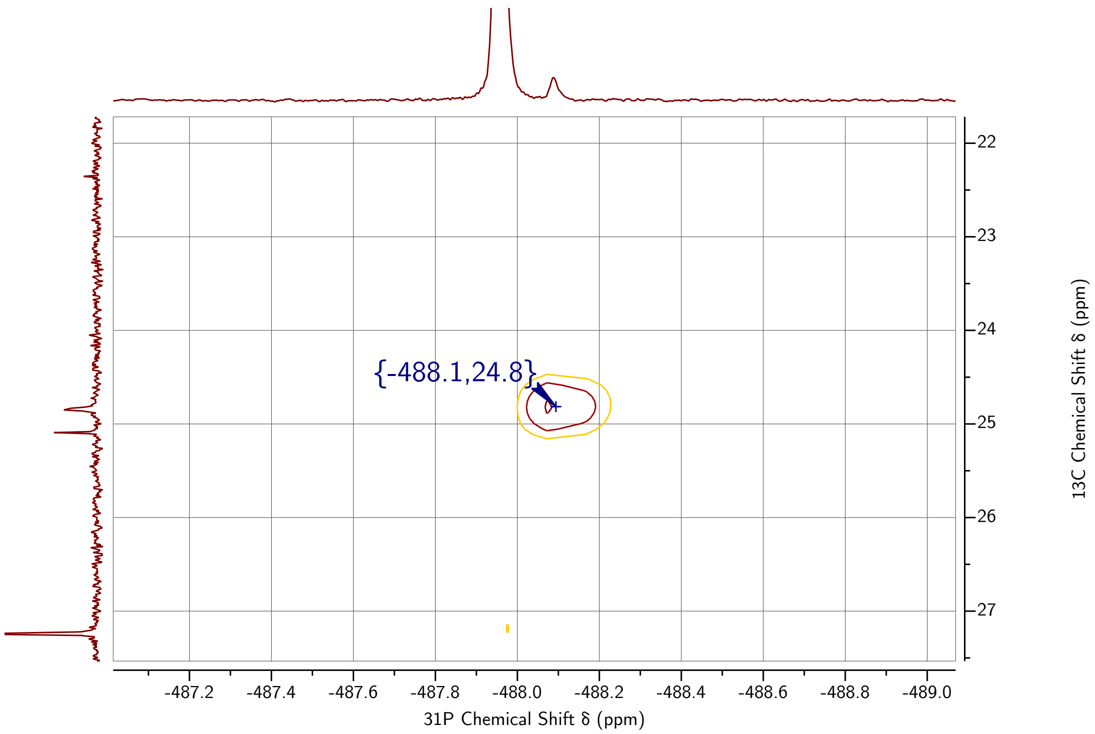

Fig. S24. ^{13}C, ^{31}P-HSQC NMR (202 MHz, benzene-d_6, 25°C) correlation experiment selective for one bond couplings in compound 1.

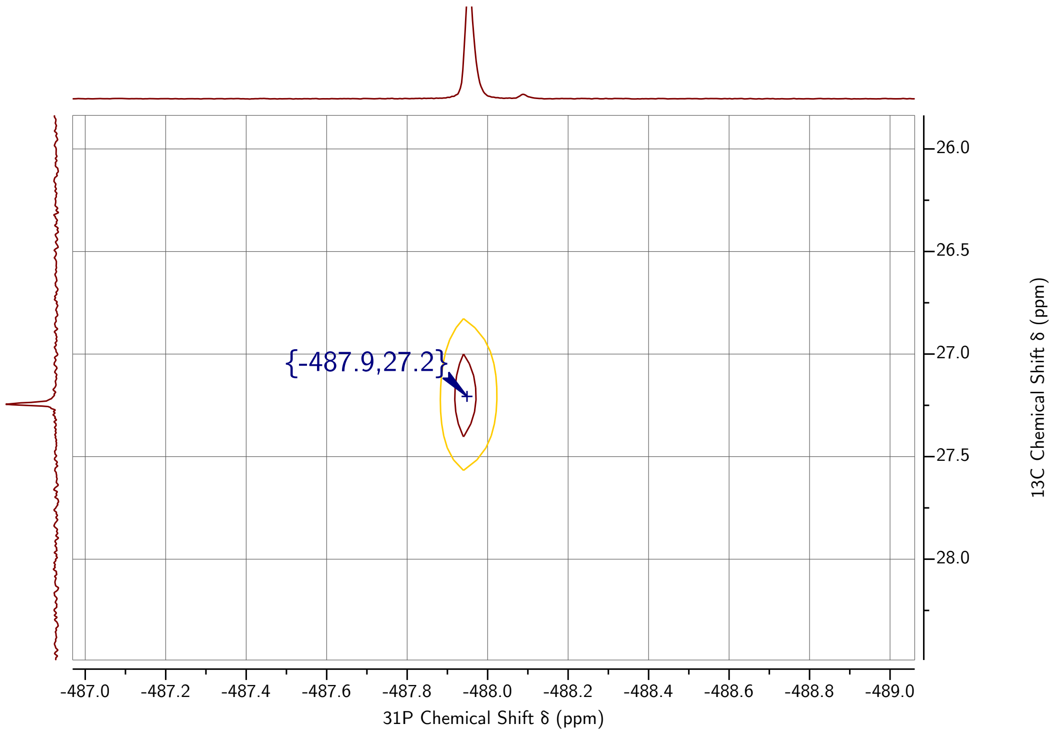

Fig. S25. ^{13}C, ^{31}P-HSQC NMR (202 MHz, benzene-d_6, 25°C) correlation experiment selective for two bond couplings in compound 1.

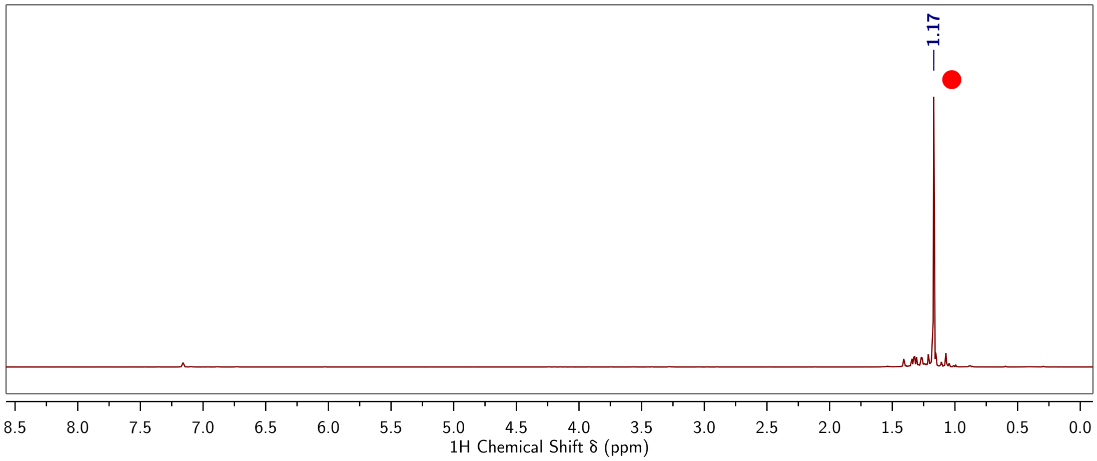

Fig. S26. ^{1}H NMR (400 MHz, benzene-d_6, 25°C) spectrum of 1 before distillation.

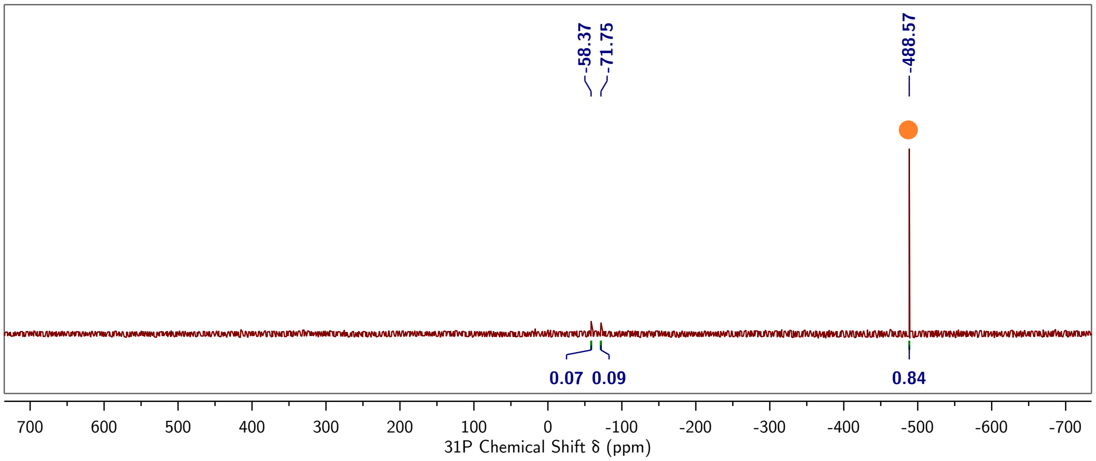

Fig. S27. $^{31}P\{^1H\}$ NMR (162 MHz, benzene-d_6, 25°C) spectrum of crude 1 before distillation.

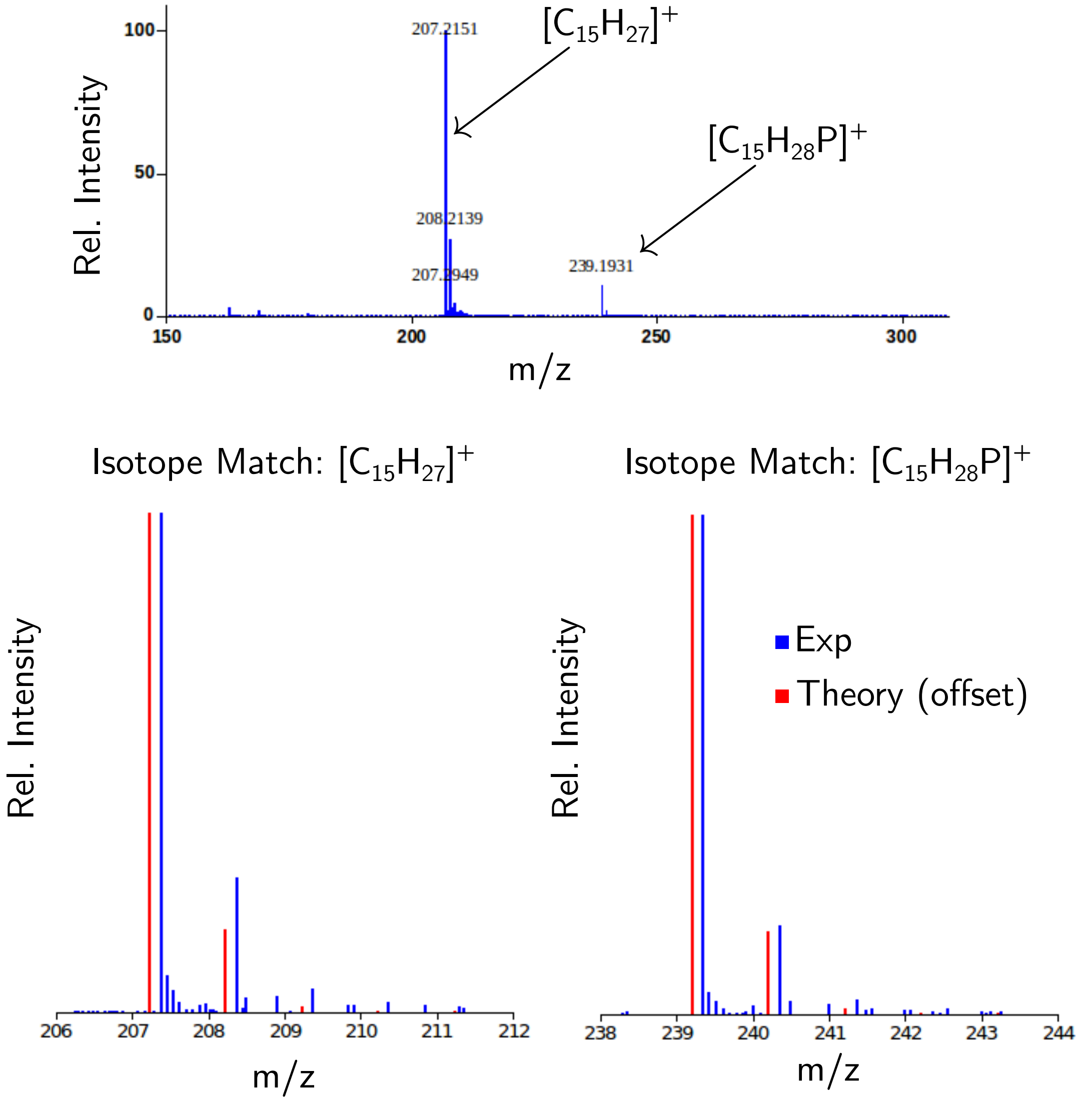

Fig. S28. DART HRMS (Q-TOF) data corresponding to $[C_{15}H_{28}P]^+$ and $[C_{15}H_{27}]^+$.

S1.7.1 Carbon-13 NMR satellites of (^{t}BuC)$_3$P (1)

Collection of ^{31}P{^{1}H} NMR data on a 162 MHz instrument led to the observation of two sets of isotope shifted satellites associated with carbon-13 incorporation in the tetrahedrane core (green) and in the tert-butyl groups (blue) (Fig. S29 and Fig. S30). However, the corresponding signal of each set was obscured by the main, unsubstituted phosphatetrahedrane ^{31}P NMR signal (orange). Collection of ^{31}P{^{1}H} NMR data on a 202 MHz instrument resolved the ^{13}C satellites associated with carbon-13 incorporation in the tetrahedrane core (green). Also note that the $^1J_{\mathrm{PC}}$ coupling constant of this set of satellites is 37.9 Hz, identical to what is measured in the ^{13}C{^{1}H} NMR spectrum (Fig. S18). Integration of the natural abundance ^{13}C satellites is consistent with three equivalent carbon atoms bonded to a single phosphorus atom.

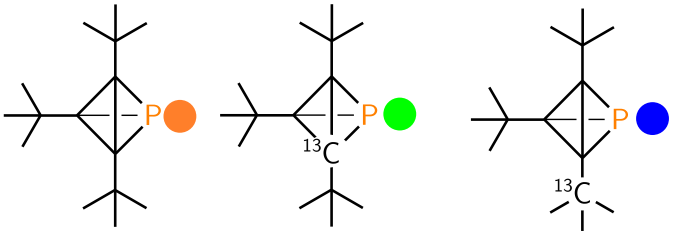

Fig. S29. Labeling scheme for natural abundance ^{13}C satellites observed in ^{31}P{^{1}H} NMR spectra.

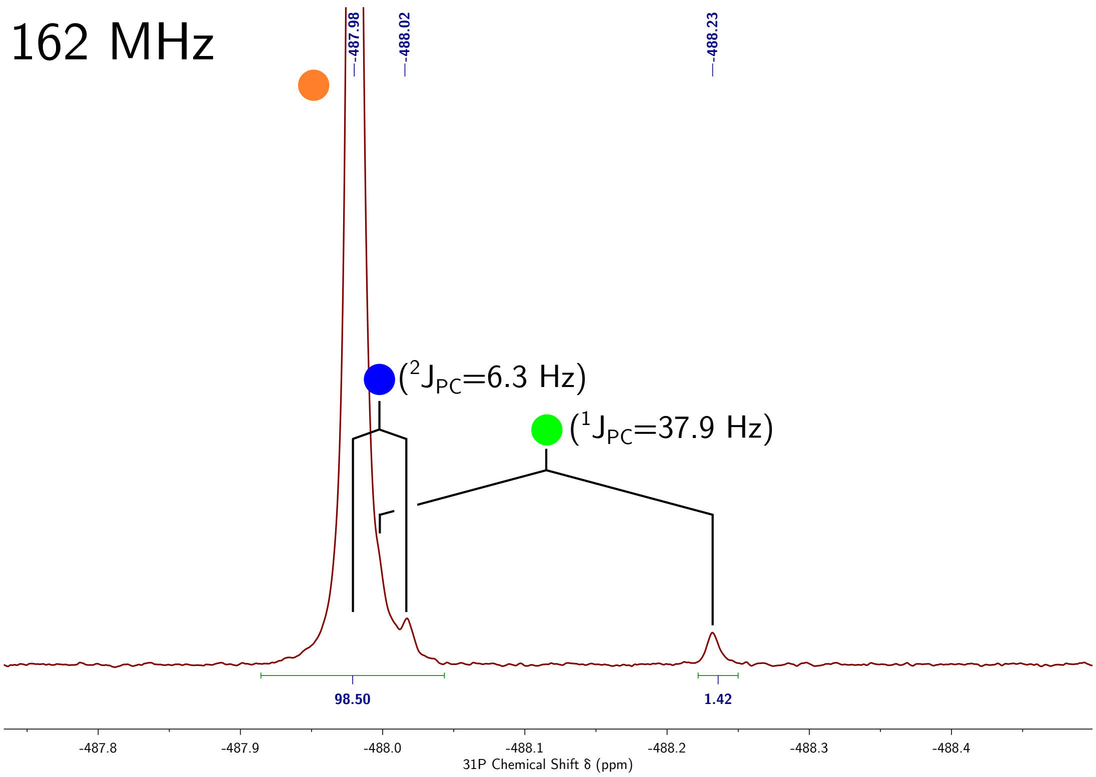

Fig. S30. $^{31}P\{^1H\}$ NMR (162 MHz, benzene-d_6, 25°C) spectrum of 1.

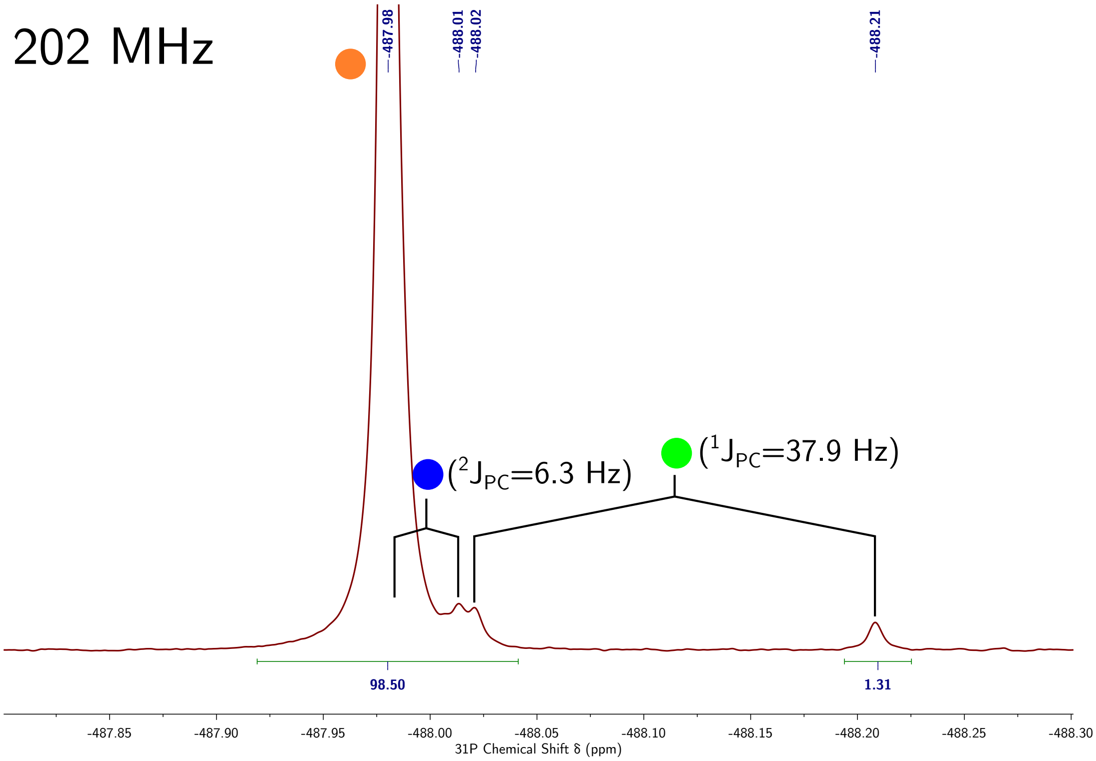

Fig. S31. $^{31}P\{^{1}H\}$ NMR (202 MHz, benzene-d_6, 25°C) spectrum of 1.

S1.7.2 Raman spectrum of $(^t\text{BuC})_3\text{P}$ (1)

In a glovebox, $(^t\text{BuC})_3\text{P}$ (**1**, 2 mg, 0.008 mmol) was loaded into a quartz capillary, and the opening was sealed with vacuum grease. The capillary was placed in the instrument and analyzed using 538 nm excitation. Density functional theory (DFT) calculations were carried out as described in section S3.1. A band was observed in the experimental spectrum S32 at 1580 cm^{-1}, corresponding to the totally symmetric breathing mode (a_1), according to pseudo-C_{3v} symmetry.

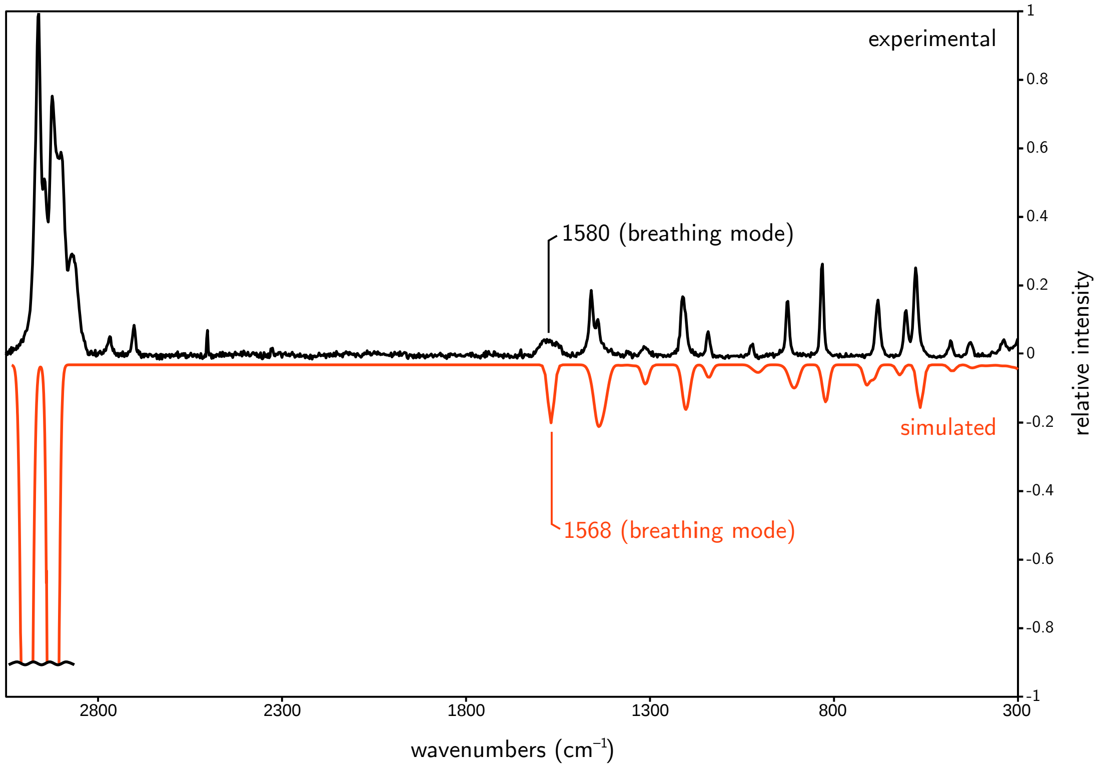

Fig. S32. Experimental (black) and calculated (red) Raman spectrum of 1. A scaling factor of 0.955 was applied to the predicted vibrational frequencies of **1** (38), as discussed in S3.1.

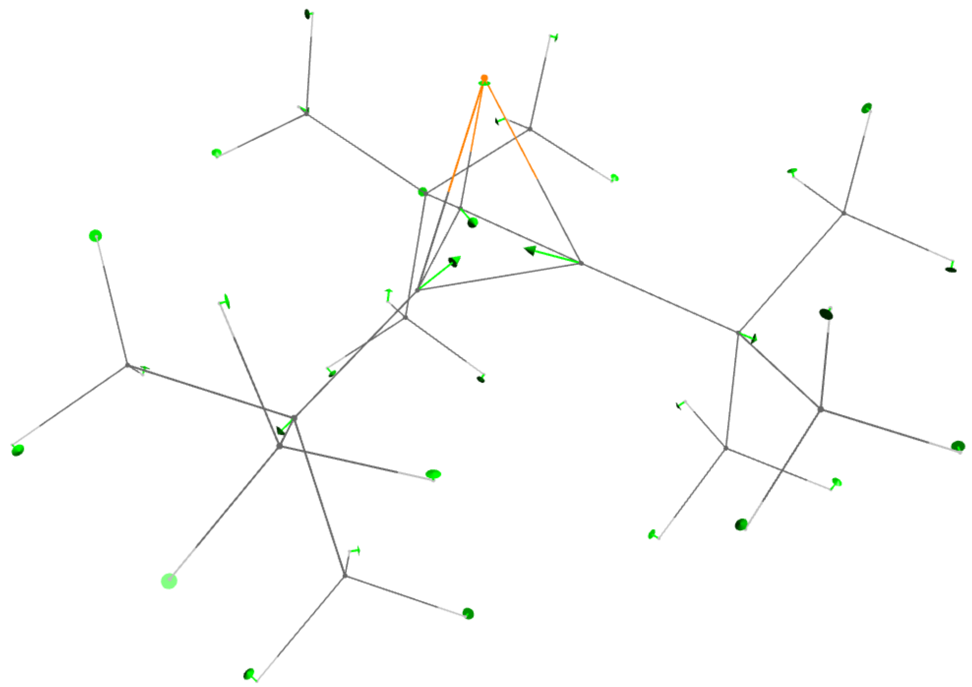

Fig. S33. Visualization of the totally symmetric breathing mode (a_1), according to pseudo-C_{3v} symmetry, of 1.

S1.7.3 Generation of [(^{t}BuC)$_3$P(H)A][OTf]

To a 20 mL scintillation vial charged with a solution of (^{t}BuC)$_3$P**A** (**9**, 0.050 g, 0.12 mmol, 1.00 equiv) in THF (1 mL) was added a solution of trifluoromethanesulfonic acid (0.018 g, 0.12 mmol, 1.00 eq) in THF (1 mL). After vigorously stirring for 20 min, all volatile materials were removed in vacuo, resulting in white solids. While [(^{t}BuC)$_3$P(H)**A**][OTf] was not observed by DART HRMS(Q-TOF), anthracene ($[M+H]^+$ Calcd for $C_{14}H_{10}$ 179.0860; Found 179.0844) and [^{t}Bu$_3$C$_3$]$^+$ ($[M]^+$ Calcd for $C_{15}H_{27}$ 207.2113; Found 207.2197) were observed (Fig. S39). ^{1}H NMR (500 MHz, THF-d_8, 25 °C, Fig. S35) δ 7.33 (dd, J = 4.8, 2.7 Hz, 2H), 7.16 (dd, J = 5.2, 3.1 Hz, 2H), 6.99 (dd, J = 5.3, 3.1 Hz, 2H), 6.77 (dd, J = 5.3, 3.1 Hz, 2H), 5.39 (d, $^1J_{PH}$ = 160.9 Hz, 1H), 4.35 (d, $^2J_{PH}$ = 14.3 Hz, 2H), 1.56 (s, 27H) ppm. ^{19}F{^{1}H} NMR (471 MHz, THF-d_8, 25 °C, Fig. S36) δ −79.27 ppm. ^{31}P{^{1}H} NMR (202 MHz, THF-d_8, 25 °C, Fig. S38) δ 161.38 (dt, J_{PH}= 160.6, 12.8 Hz) ppm.

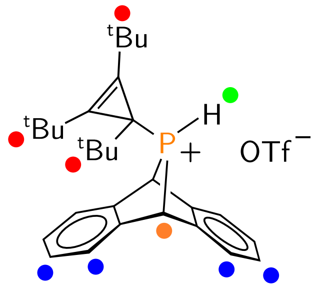

Fig. S34. Labeling scheme for [(^{t}BuC)$_3$P(H)A][OTf].

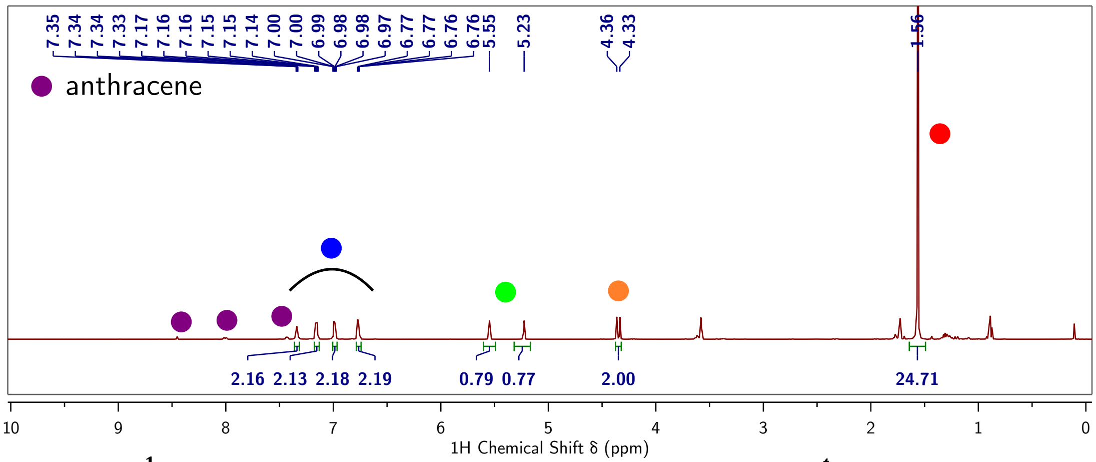

Fig. S35. **^{1}H NMR (500 MHz, THF-d_8, 25°C) spectrum of [(^{t}BuC)$_3$P(H)A][OTf].**

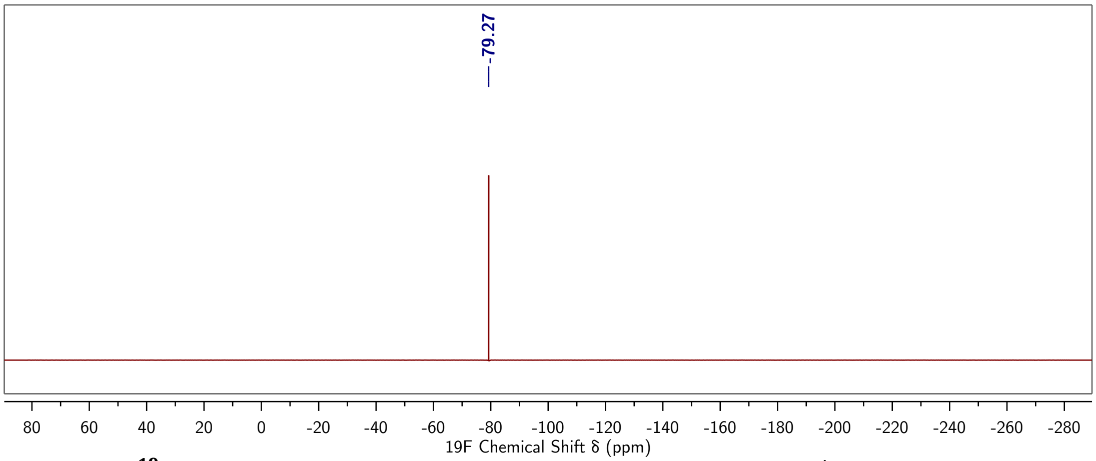

Fig. S36. **^{19}F NMR (471 MHz, THF-d_8, 25°C) spectrum of [(^{t}BuC)$_3$P(H)A][OTf].**

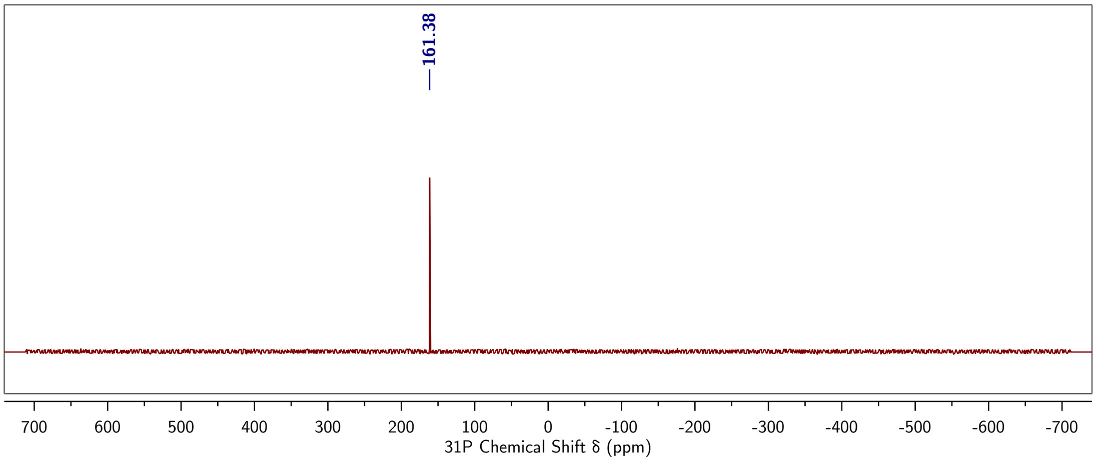

Fig. S37. **^{31}P{^{1}H} NMR (202 MHz, THF-d_8, 25°C) spectrum of [(^{t}BuC)$_3$P(H)A][OTf].**

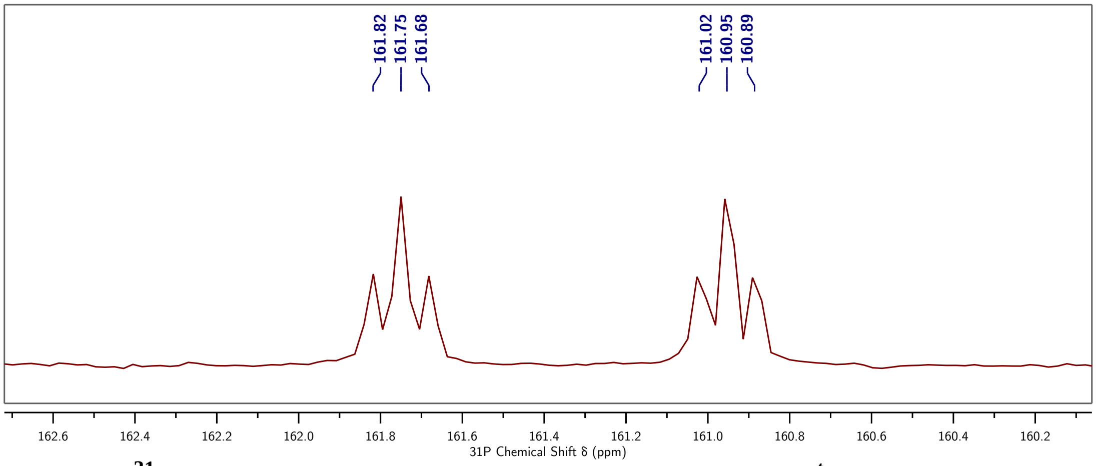

Fig. S38. ^{31}**P NMR (202 MHz, THF-*d*$_8$, 25°C) spectrum of [(^{t}BuC)$_3$P(H)A][OTf].**

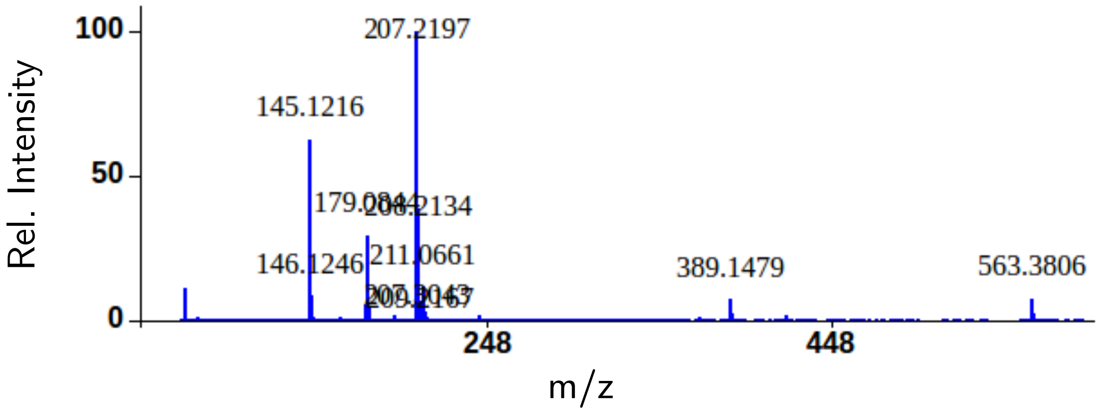

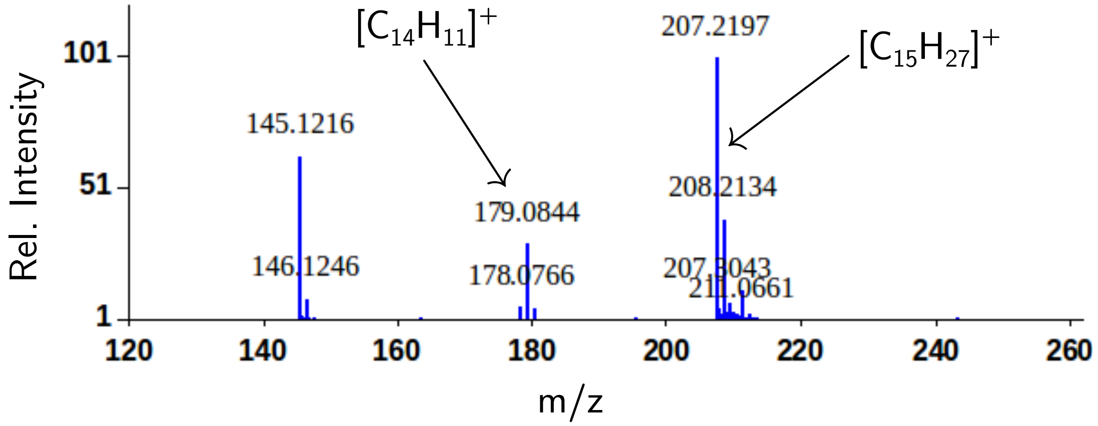

Fig. S39. DART HRMS (Q-TOF) data corresponding to [$C_{15}H_{27}$]$^+$ and [$C_{14}H_{11}$]$^+$.

S1.7.4 Stability of [(^{t}BuC)$_3$P(H)A][OTf] in solution

To a 20 mL scintillation vial charged with a solution of (^{t}BuC)$_3$P**A** (**9**, 0.050 g, 0.12 mmol, 1.00 equiv) in THF (1 mL) was added a solution of trifluoromethanesulfonic acid (0.018 g, 0.12 mmol, 1.00 eq) in THF (1 mL). After stirring for 20 min, the solution was then filtered through Celite®. An aliquot (0.5 mL) of the solution was transferred to an NMR tube. To this NMR tube was added a glass capillary containing a 0.67 M solution of Ph_3P in benzene-d_6. This reaction was monitored by $^{31}P\{^1H\}$ NMR (see Fig. S40 and Fig. S41) over a period of 16 h. After 16 h at 23 °C, the solution became pale yellow and cloudy and the $^{31}P\{^1H\}$ NMR spectrum revealed moderate consumption of [(^{t}BuC)$_3$P(H)**A**][OTf] and the formation of PH_3 and (^{t}BuC)$_3$PH$_2$ (39).

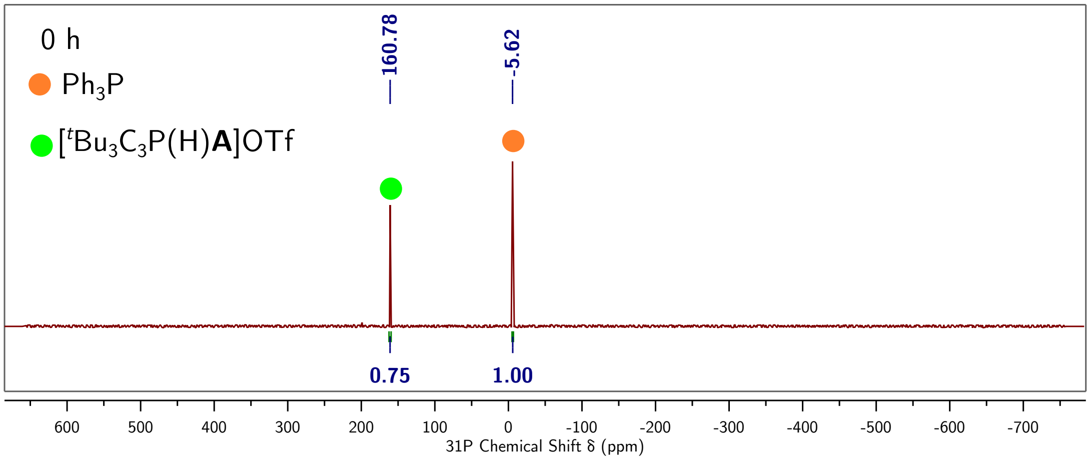

Fig. S40. Initial $^{31}P\{^1H\}$ NMR (162 MHz, THF, 25°C) spectrum of [(^{t}BuC)$_3$P(H)A][OTf].

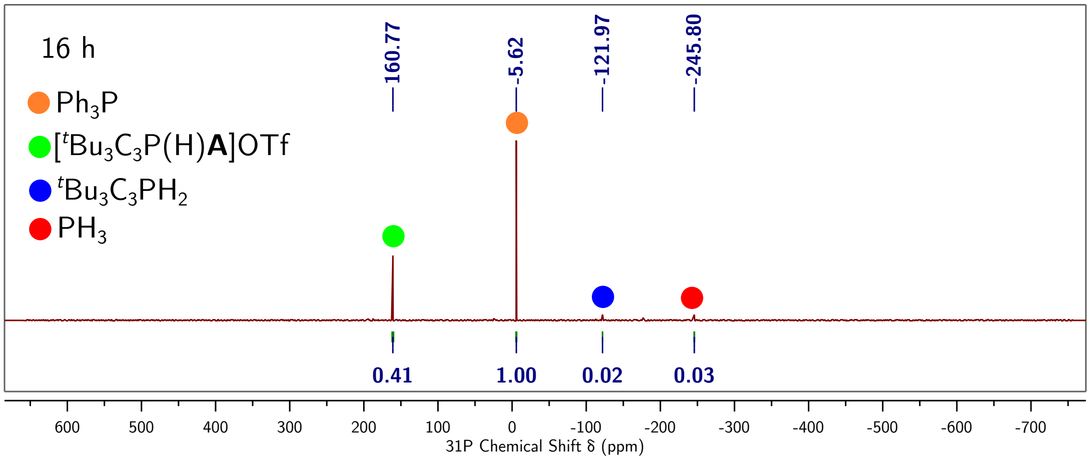

Fig. S41. $^{31}P\{^{1}H\}$ **NMR (162 MHz, THF, 25°C) spectrum of** $[(^{t}BuC)_3P(H)A][OTf]$ **after 16 hours.**

S1.7.5 Generation of (^{t}BuC)$_3$P(F)H (10)

To a 20 mL scintillation vial charged with a solution of (^{t}BuC)$_3$PA (**9**, 0.050 g, 0.12 mmol, 1.0 equiv) in THF (1 mL) was added a solution of trifluoromethanesulfonic acid (0.018 g, 0.12 mmol, 1.00 eq) in THF (1 mL). After stirring for 20 min, a slurry of tetramethylammonium fluoride (0.011 g, 0.12 mmol, 1.0 equiv) in THF (1 mL) was added dropwise. After stirring for 30 min, all volatile materials were then removed *in vacuo* from the solution, resulting in a colorless residue. This material was slurried in pentane (2 mL) and the solution was filtered through a glass fiber filter paper plugged pipette (2 mL) containing a one-inch plug of charcoal. All volatile materials were then removed *in vacuo*, resulting in a colorless oil (22 mg). While **10** was not observed by DART HRMS(Q-TOF), [^{t}Bu$_3$C$_3$]$^+$ ([M]$^+$ Calcd for $C_{15}H_{27}$ 207.2113; Found 207.2132) was observed (Fig. S47). ^{1}H NMR (400 MHz, benzene-d_6, 25 °C, Fig. S43) δ 7.28 (dd, $^1J_{PH}$ = 187.5 Hz, $^2J_{FH}$ = 45.4 Hz, 1H), 1.11 (s, 9H), 1.11 (s, 18H) ppm. ^{19}F{^{1}H} NMR (471 MHz, benzene-d_6, 25 °C, Fig. S44) δ −226.32 (d, $^1J_{PF}$ = 801.3 Hz) ppm. ^{31}P{^{1}H} NMR (162 MHz, benzene-d_6, 25 °C, Fig. S46) δ 182.05 (dd, $^1J_{PF}$ = 801.3 Hz, $^1J_{PH}$ = 187.4 Hz) ppm.

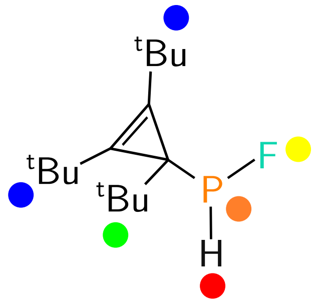

Fig. S42. Labeling scheme for 10.

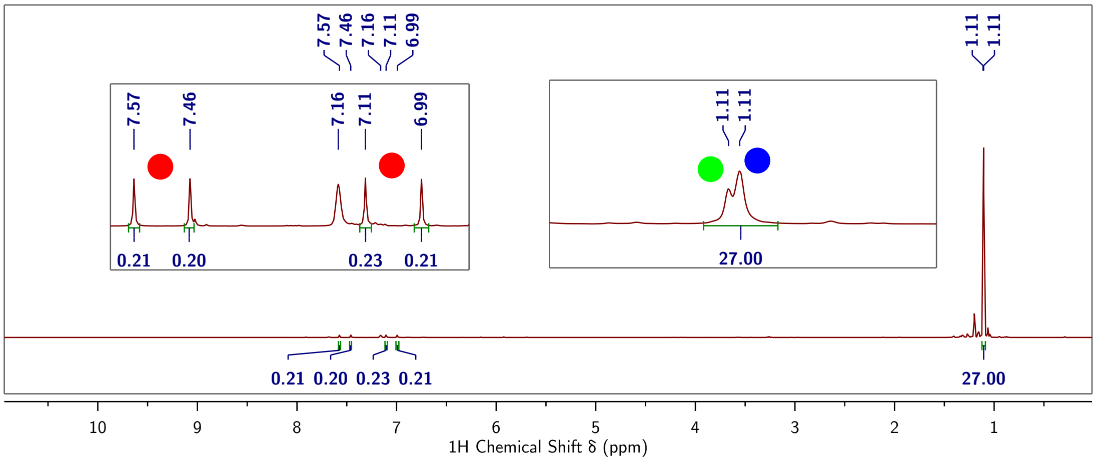

Fig. S43. ^{1}H NMR (400 MHz, benzene-d_6, 25°C) spectrum of **10**.

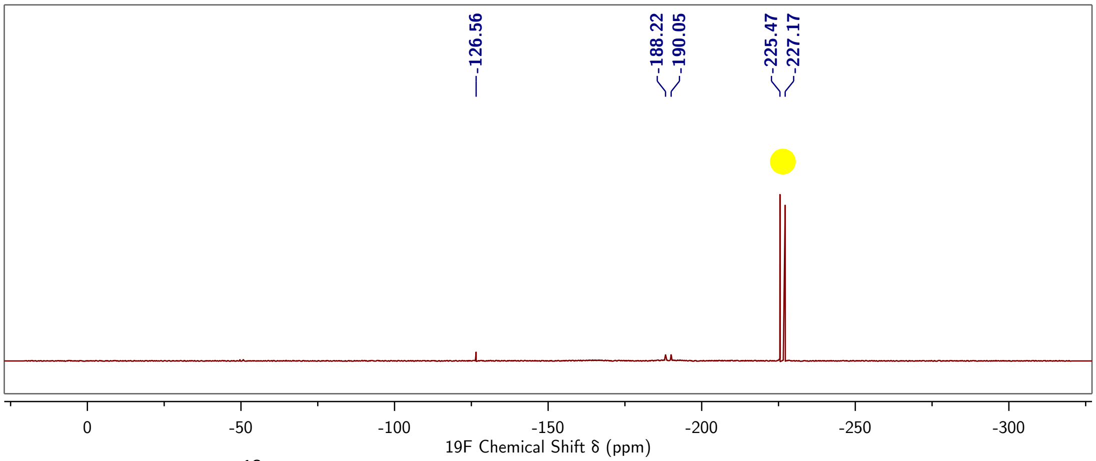

Fig. S44. ^{19}F NMR (471 MHz, benzene-d_6, 25°C) spectrum of **10**.

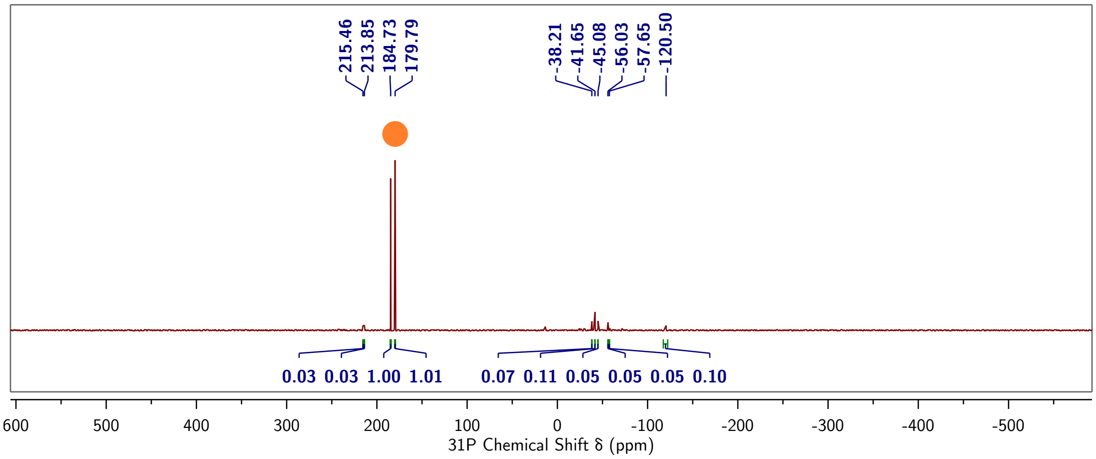

Fig. S45. ^{31}P{^{1}H} NMR (162 MHz, benzene-d_6, 25°C) spectrum of **10**.

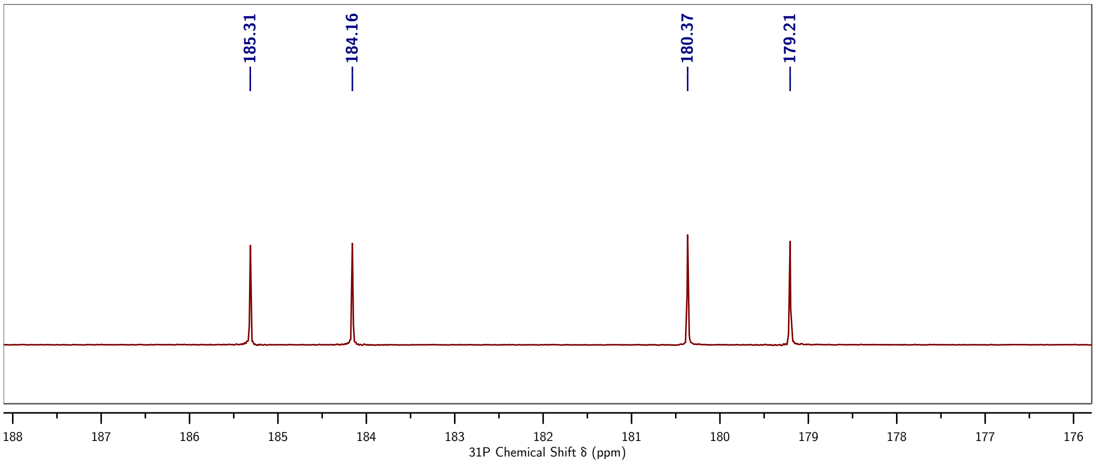

Fig. S46. ^{31}P NMR (162 MHz, benzene-d_6, 25°C) spectrum of 10.

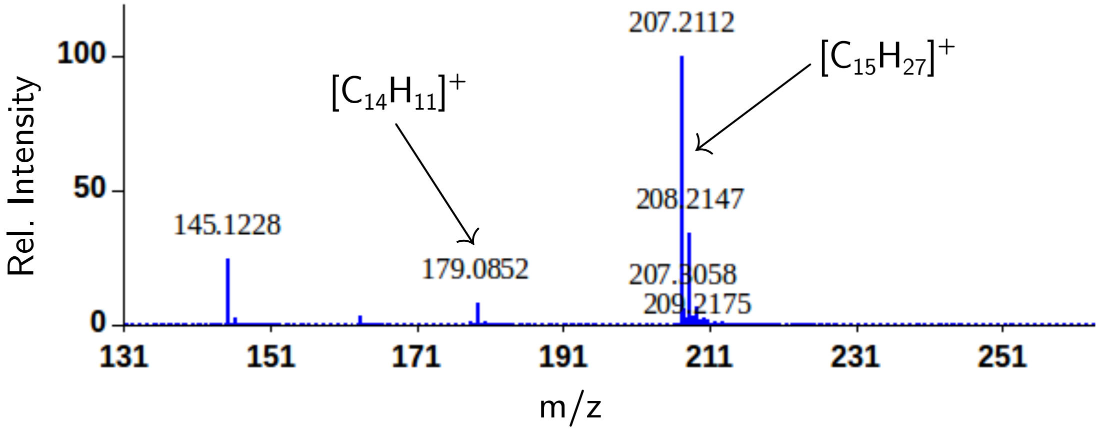

Fig. S47. DART HRMS(Q-TOF) data corresponding to $[C_{15}H_{27}]^+$ and $[C_{14}H_{11}]^+$.

S1.7.6 Stability of (^{t}BuC)$_3$P(F)H (10) in solution

A solution of (^{t}BuC)$_3$P(F)H (**10**) in benzene-d_6 was transferred to an NMR tube. To this NMR tube was added a glass capillary containing a 0.67 M solution of Ph_3P in benzene-d_6. This sample was monitored by $^{31}P\{^1H\}$ NMR spectroscopy over a period of 48 h (see Fig. S48 and Fig. S49). Slight consumption of **10** was observed after 48 h at 23 °C.

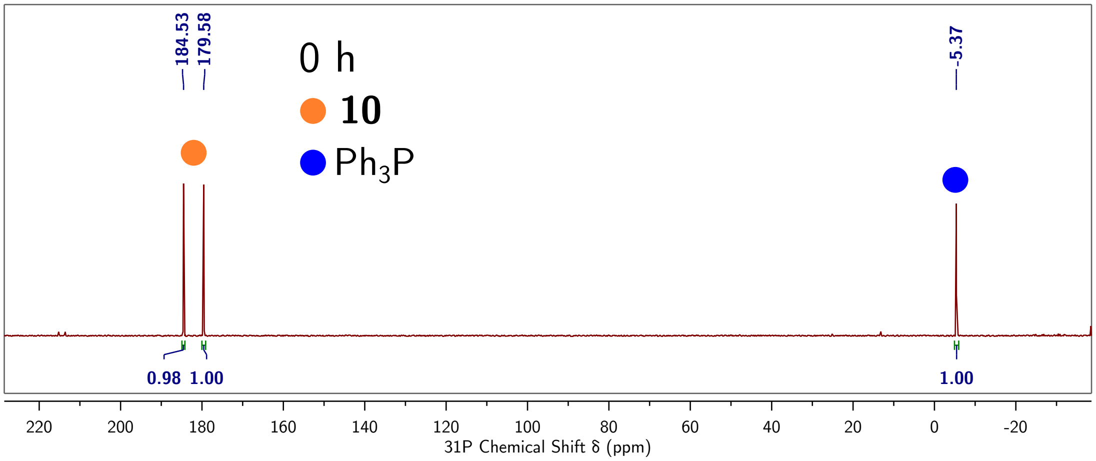

Fig. S48. ^{31}**P{**^{1}**H} NMR (162 MHz, benzene-*d*$_6$, 25°C) spectrum of 10.**

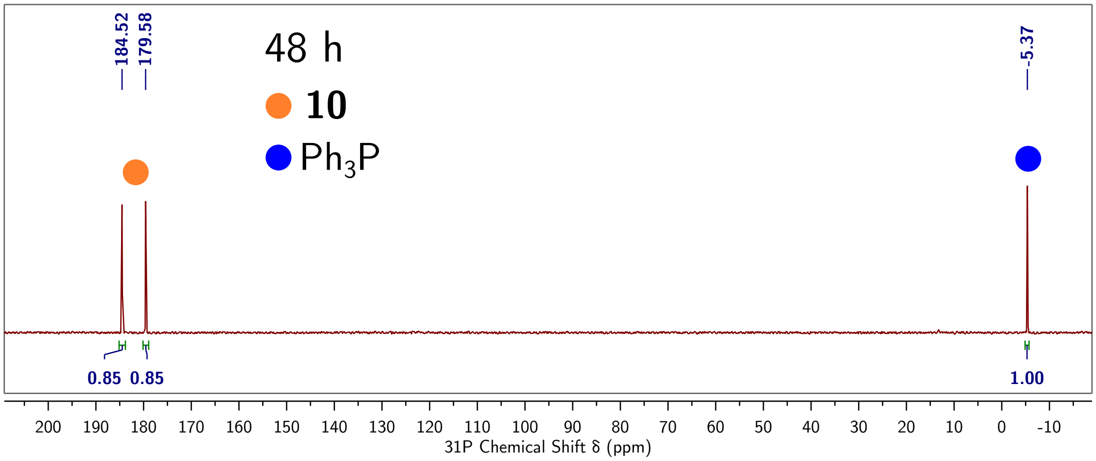

Fig. S49. ^{31}**P{**^{1}**H} NMR (162 MHz, benzene-*d*$_6$, 25°C) spectrum of 10 after 48 hours.**

S1.7.7 Generation of $(^t\text{BuC})_3\text{P(Cl)H}$ (11)

To a 20 mL scintillation vial charged with a solution of $(^t\text{BuC})_3\text{P}$**A** (**9**, 0.050 g, 0.12 mmol, 1.0 equiv) in THF (1 mL) was added a solution of trifluoromethanesulfonic acid (0.018 g, 0.012 mmol, 1.00 eq) in THF (1 mL). After stirring for 20 min, the heterogeneous solution was added to a vial containing tetrabutylammonium chloride (0.033 g, 0.12 mmol, 1.00 eq). After vigorously stirring for 20 min, the reaction mixture became homogeneous. This solution was filtered through a glass fiber filter paper plugged pipette (2 mL) containing a one-inch plug of Celite®. Removal of volatile materials under reduced pressure was found to result in the complete consumption of **11** and formation of unidentified phosphorus-containing species. ^{1}H NMR (400 MHz, benzene-d_6, 25 °C, Fig. S51) δ 5.67 (d, $^1J_{\text{PH}}$ = 187.1 Hz). ^{31}P{^{1}H} NMR (162 MHz, benzene-d_6, 25 °C, Fig. S46) δ 82.21 (d, $^1J_{\text{PH}}$ = 187.5 Hz) ppm.

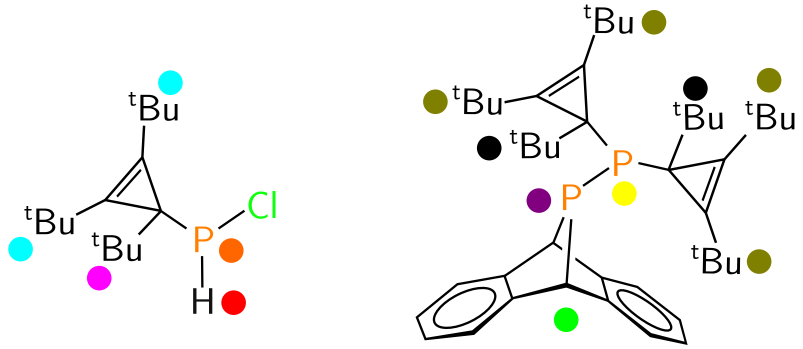

11 **Observed byproduct**

Fig. S50. Labeling scheme for 11 and observed by-product.

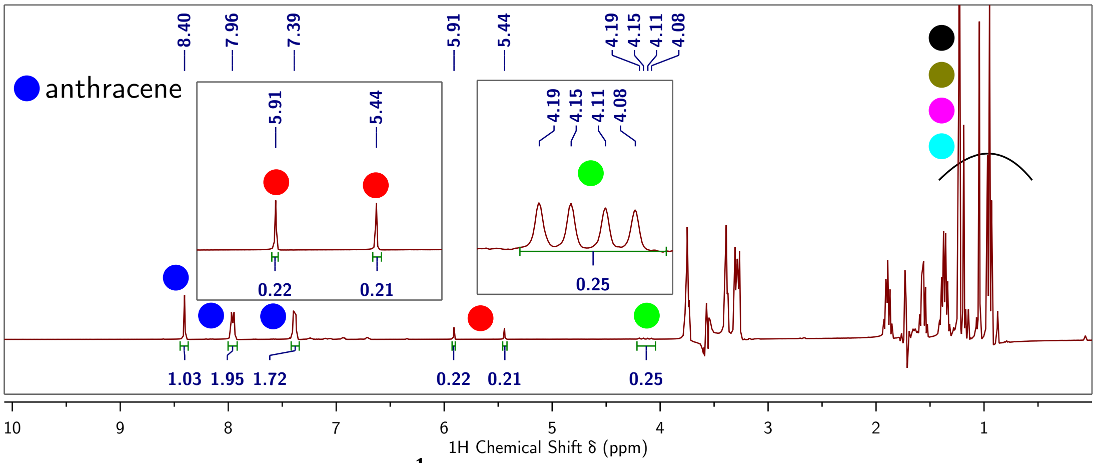

Fig. S51. Solvent supressed ^{1}H NMR (400 MHz, THF, 25°C) spectrum of 11.

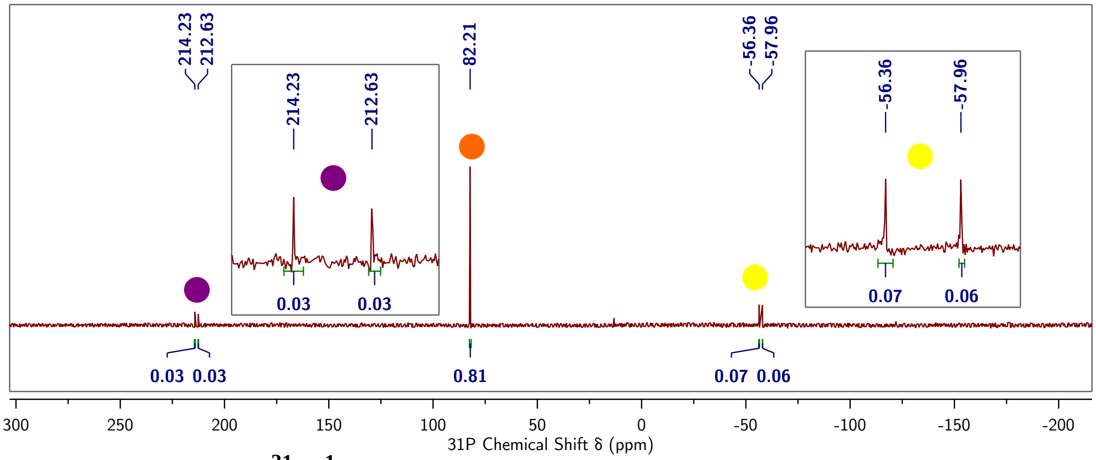

Fig. S52. $^{31}P\{^{1}H\}$ NMR (162 MHz, THF, 25°C) spectrum of 11.

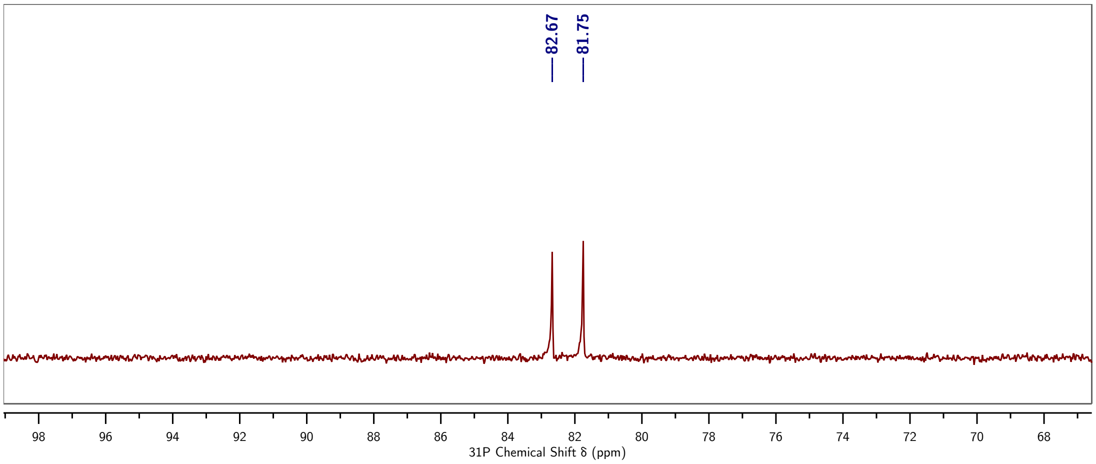

Fig. S53. ^{31}P NMR (162 MHz, THF, 25°C) spectrum of 11.

S1.8 Air stability of $({}^{t}BuC)_3P$ (1) in solution

A 0.007 M solution of **1** in benzene-d_6 was prepared and transferred to an NMR tube. To this tube was added a glass capillary containing a 0.67 M solution of Ph_3P in benzene-d_6 and an initial $^{31}P\{^1H\}$ NMR spectrum was collected (Fig. S54). The cap of the tube was removed outside of the glovebox for a period of 30 min. After this period, a $^{31}P\{^1H\}$ NMR spectrum was collected (Fig. S55), revealing no consumption of **1**. An additional $^{31}P\{^1H\}$ NMR spectrum was collected after being exposed to air for 12 h (Fig. S56), revealing complete consumption of **1**.

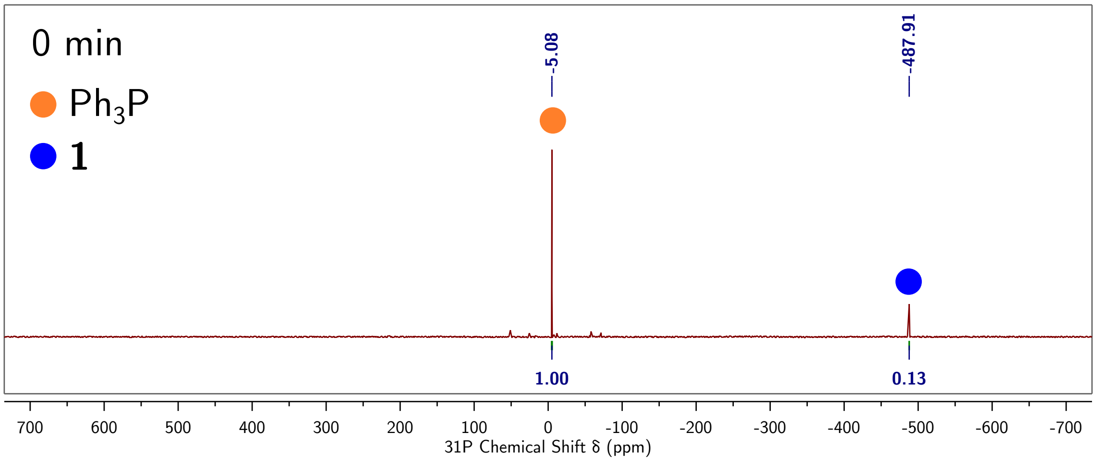

Fig. S54. $^{31}P\{^1H\}$ NMR (162 MHz, benzene-d_6, 25°C) spectrum of 1 in benzene-d_6 before air exposure.

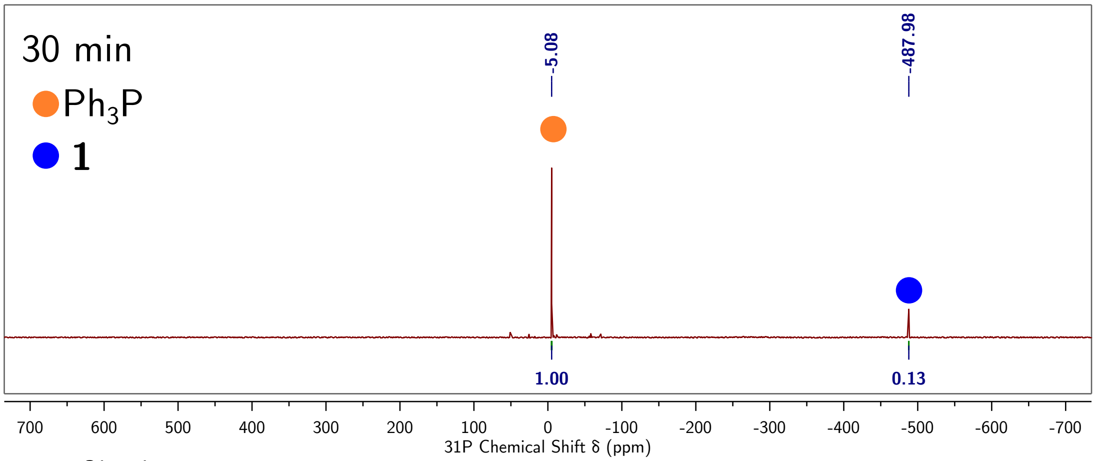

Fig. S55. $^{31}P\{^1H\}$ **NMR (162 MHz, benzene-*d*$_6$, 25°C) spectrum of 1 in benzene-*d*$_6$ after being exposed to air for 30 min.**

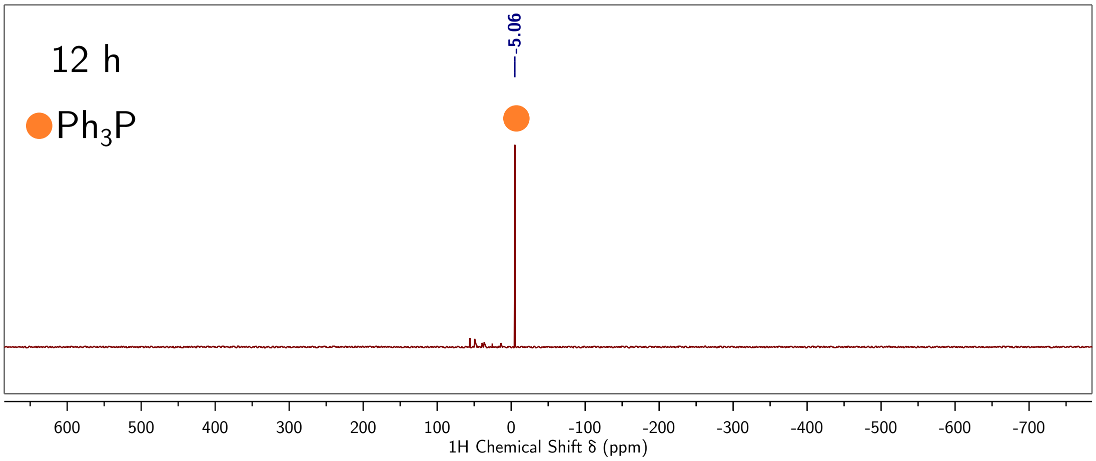

Fig. S56. $^{31}P\{^1H\}$ **NMR (162 MHz, benzene-*d*$_6$, 25°C) spectrum of 1 in benzene-*d*$_6$ after being exposed to air for 12 hours.**

S1.9 Thermal stability of $(^{t}BuC)_3P$ (1) in solution (75 °C)

A 0.02 M solution of **1** in benzene-d_6 was prepared and transferred to an J. Young tube. To this tube was added a glass capillary containing a 0.67 M solution of Ph_3P in benzene-d_6 and an initial $^{31}P\{^{1}H\}$ NMR spectrum was collected (Fig. S57). This tube was then placed in a preheated 75 °C oil bath for 45 min. After this period a $^{31}P\{^{1}H\}$ NMR spectrum was collected (Fig. S58), revealing no consumption of **1**.

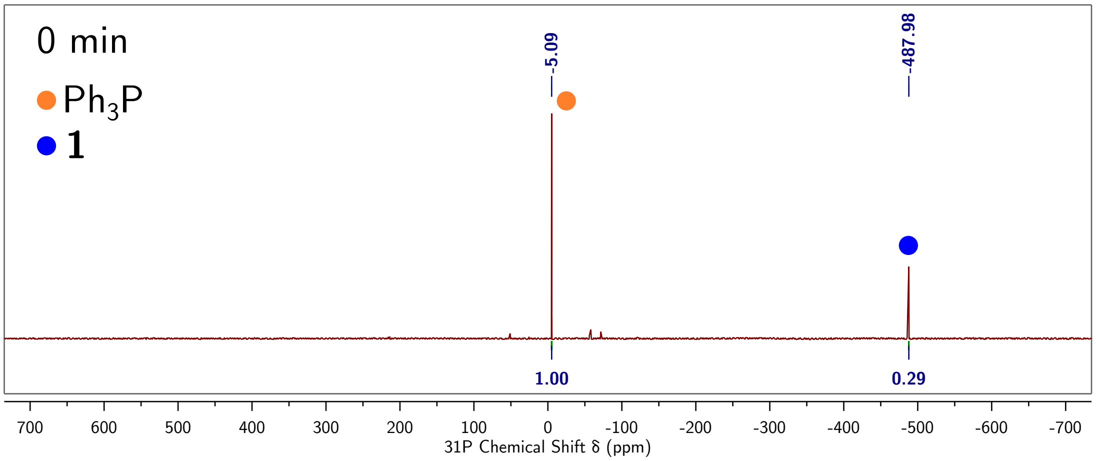

Fig. S57. ^{31}**P{**^{1}**H} NMR (162 MHz, benzene-*d*$_6$, 25°C) spectrum of 1 in benzene-*d*$_6$ before being heated.**

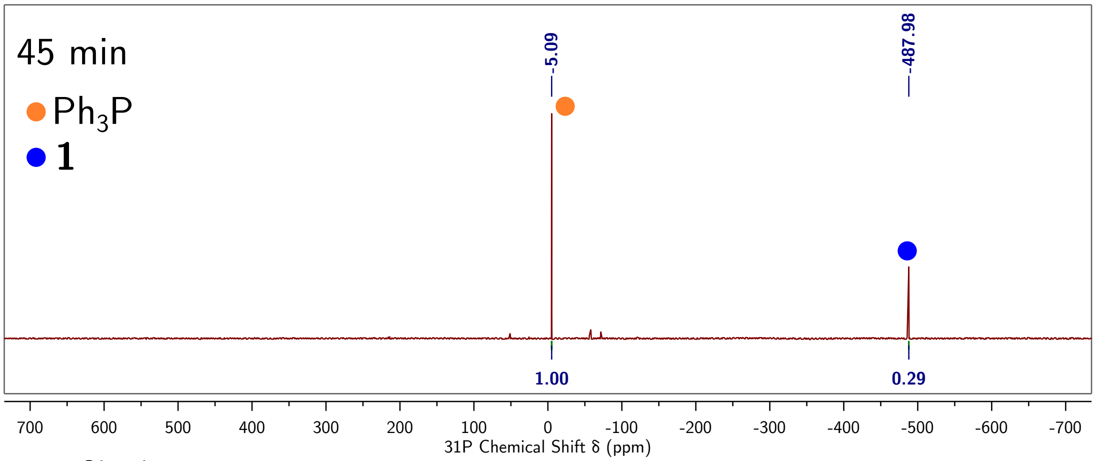

Fig. S58. ^{31}**P{**^{1}**H} NMR (162 MHz, benzene-*d*$_6$, 25°C) spectrum of 1 in benzene-*d*$_6$ after being heated for 45 min at 75°C.**

S1.10 Thermal stability of (^{t}BuC)$_3$P (1) in solution (130 °C)

A 0.06 M solution of **1** in toluene-d_8 was prepared and transferred to a flame-sealed NMR tube. An initial ^{31}P{^{1}H} NMR spectrum was collected (Fig. S59). This tube was transferred to a preheated 130 °C oil bath. After 3 h, a ^{31}P{^{1}H} NMR spectrum was collected (Fig. S60), revealing formation of a diphosphahousene previously reported by Slootweg et al. (14) and a small amount of what we tentatively assign as phosphacyclobutadiene dimer (refer to section S1.13 for more information).

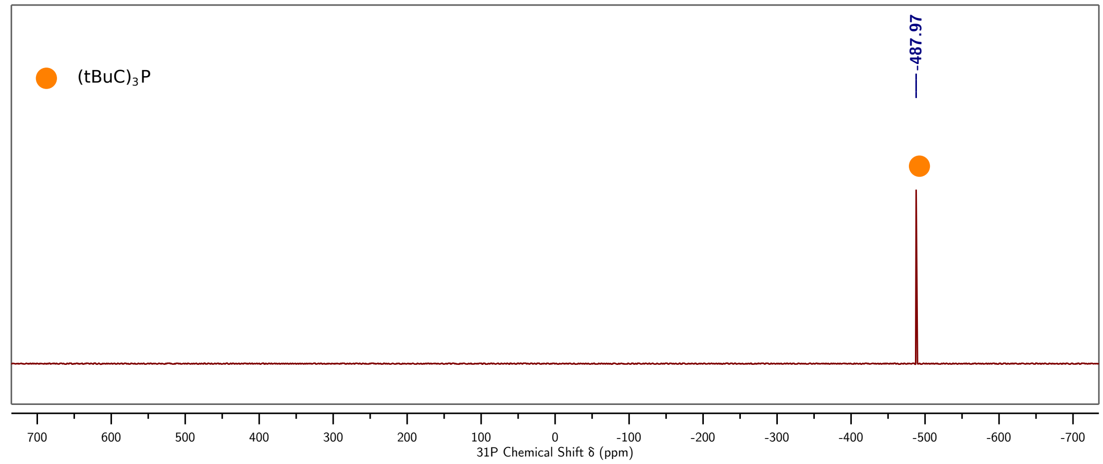

Fig. S59. ^{31}P{^{1}H} NMR (162 MHz, toluene-d_8, 25°C) spectrum of 1 in toluene-d_8 before being heated.

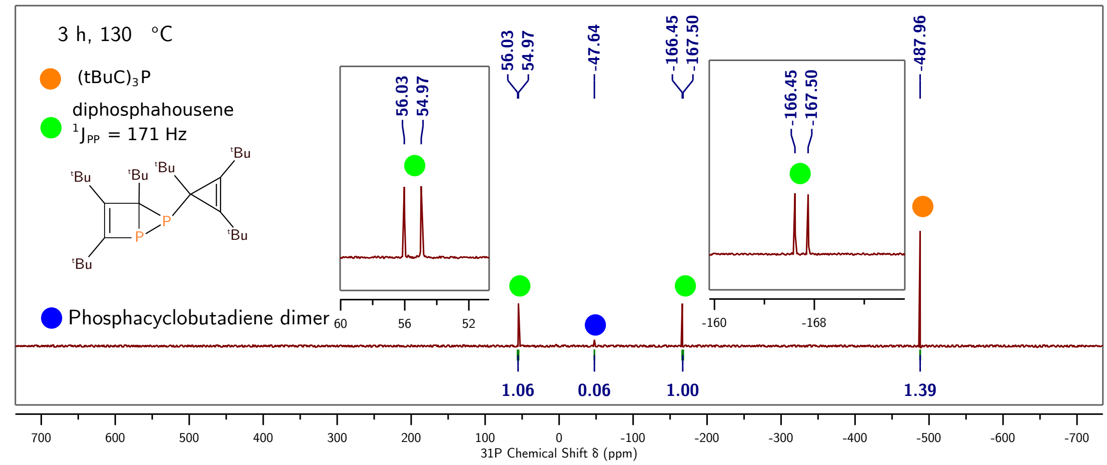

Fig. S60. $^{31}P\{^1H\}$ **NMR (162 MHz, toluene-d_8, 25°C) spectrum of 1 in toluene-d_8 after being heated for 3 hours at 130°C.**

S1.11 Photolysis of (^{t}BuC)$_3$P (1)

A 0.020 M solution of (^{t}BuC)$_3$P (**1**) in pentane was prepared and transferred to a quartz NMR tube. The solution was irradiated with 254 nm light for five minutes. ^{31}P{^{1}H} NMR spectra were collected before (Fig. S61) and after irradiation (Fig. S62). Irradiation of **1** produced a number of unidentified species, a diphosphene previously reported by Slootweg et al. (14), and what we tentatively assign as phosphacyclobutadiene dimer (refer to section S1.13 for more information).

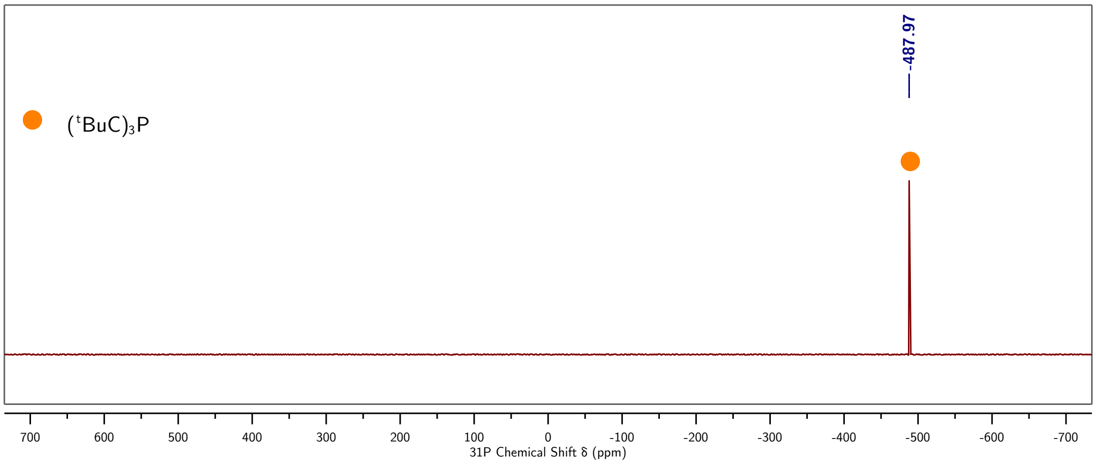

Fig. S61. ^{31}P{^{1}H} NMR (162 MHz, pentane, 25°C) spectrum of (^{t}BuC)$_3$P in pentane before being exposed to 254-nm light.

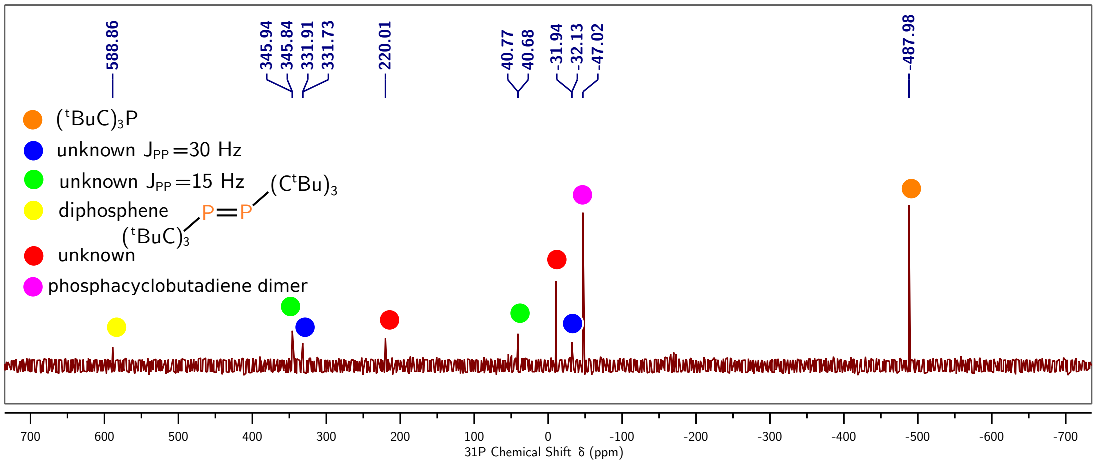

Fig. S62. $^{31}P\{^1H\}$ **NMR (162 MHz, pentane, 25°C) spectrum of $(^tBuC)_3P$ in pentane after being exposed to 254-nm light for 5 min.**

S1.12 Treatment of (^{t}BuC)$_3$P (1) with W(THF)(CO)$_5$

W(THF)(CO)$_5$ was prepared by irradiating a solution of W(CO)$_6$ (THF, 0.1 M) with 254 nm light for 1 h at 23 °C (40). The bright yellow homogeneous solution of W(THF)(CO)$_5$ was brought into the glovebox and used immediately. To a vial containing (^{t}BuC)$_3$P (**1**, 10 mg, 0.04 mmol) was added 0.5 mL (0.05 mmol, 1.25 equiv) of the freshly prepared solution. After stirring for 24 h, the solution was transferred to an NMR tube and a ^{31}P{^{1}H} NMR spectrum was collected (Fig. S63). This reaction produces a number of unknown species; however, the major product of this reaction is diphosphahousene **5**.

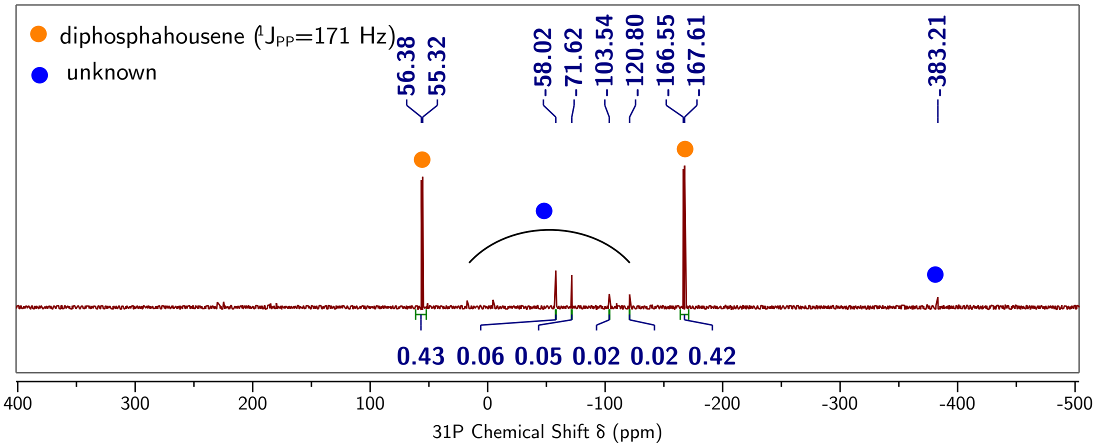

Fig. S63. ^{31}**P{**^{1}**H} NMR (162 MHz, THF, 25°C) spectrum of the crude reaction mixture, after treating (**^{t}**BuC)**$_3$**P with W(THF)(CO)**$_5$**.**

S1.13 Treatment of (^{t}BuC)$_3$P (1) with BPh$_3$

To a stirring solution of (^{t}BuC)$_3$P (**1**, 0.040 g, 0.04 mmol, 1 equiv) in benzene (1 mL) was added triphenylborane (0.004 g, 0.004 mmol, 0.1 equiv). After 10 min, all volatile materials were removed from the reaction mixture under reduced pressure. The colorless solids were taken up in pentane (2 mL) and one drop of pyridine was added to the solution. The cloudy solution was filtered through a glass fiber filter paper plugged pipette, and the all volatile materials were removed from the filtrate *in vacuo*. The colorless solids were taken up in benzene-d_6 and trans-

ferred to an NMR tube for NMR characterization (Fig. S64−S67). The major product of this reaction has been tentatively assigned as a [2+2] dimer of tri-*tert*-butyl phosphacyclobutadiene. ^{1}H NMR (400 MHz, benzene-d_6, 25 °C, Fig. S51) δ 1.47, 1.35 ppm.^{31}P{^{1}H} NMR (162 MHz, benzene-d_6, 25 °C, Fig. S46) δ −47.60 ppm. A preliminary X-ray structure determination that is consistent with our assignment has also been obtained. Further characterization of this dimer is in progress. This reaction also produces a small amount of diphosphahousene **5**.

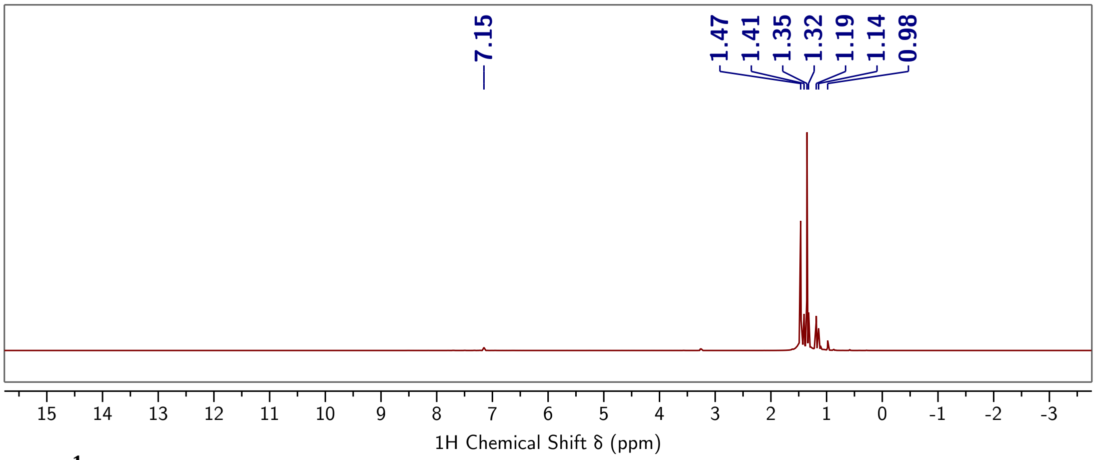

Fig. S64. ^{1}H NMR (400 MHz, benzene-d_6, 25°C) spectrum of the crude reaction mixture, after treating (^{t}BuC)$_3$P with Ph$_3$B and pyridine.

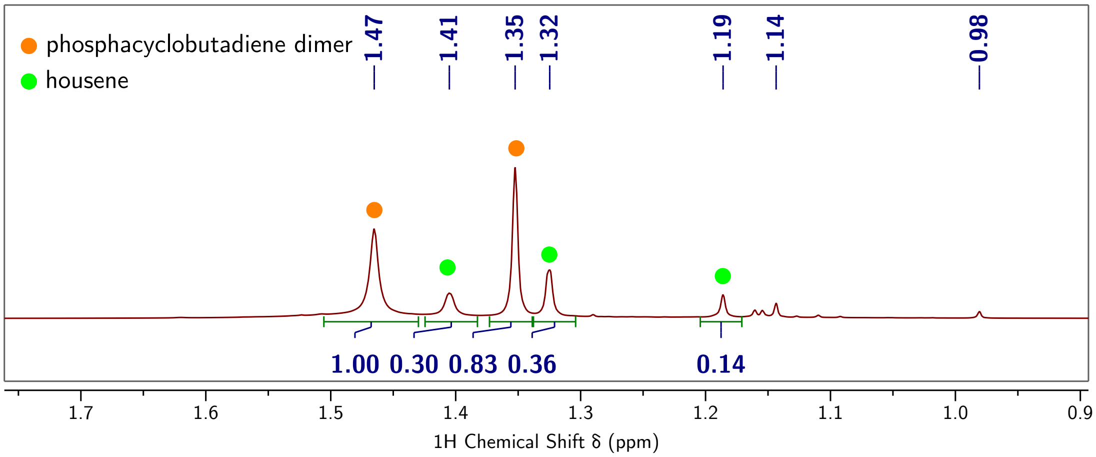

Fig. S65. ^{1}H NMR (400 MHz, benzene-d_6, 25°C) spectrum of the crude reaction mixture, after treating (^{t}BuC)$_3$P with Ph$_3$B and pyridine.

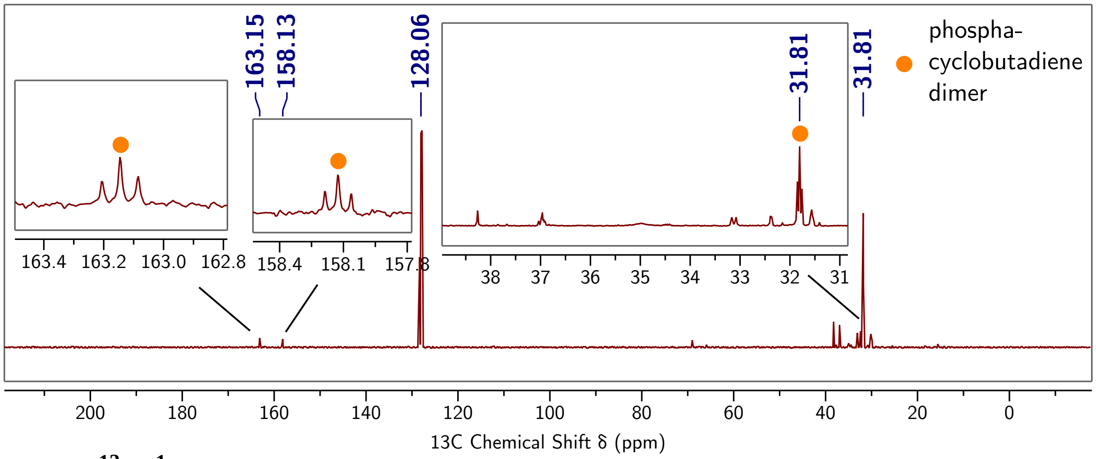

Fig. S66. $^{13}C\{^1H\}$ **NMR (101 MHz, benzene-d_6, 25°C) spectrum of the crude reaction mixture, after treating (^{t}BuC)$_3$P with Ph_3B and pyridine.**

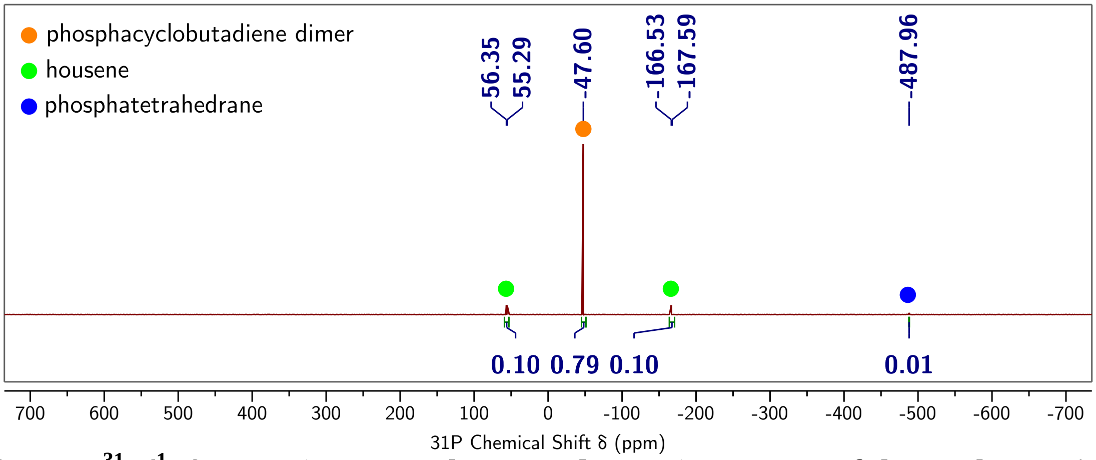

Fig. S67. $^{31}P\{^1H\}$ **NMR (101 MHz, benzene-d_6, 25°C) spectrum of the crude reaction mixture, after treating (^{t}BuC)$_3$P with Ph_3B and pyridine.**

Section S2. X-ray diffraction studies

Low-temperature diffraction data were collected on a Bruker-AXS X8 Kappa Duo diffractometer with $I\mu S$ micro-sources, coupled to a Photon 3 CPAD detector using Cu K_α radiation (λ = 1.54178 Å) for the structure of **1** and a Smart APEX2 CCD detector using Mo K_α radiation (λ = 0.71073 Å) for the structures of Na[**8**] and **9**, performing ϕ- and ω-scans. The structures were solved by dual-space methods using SHELXT (41) and refined against F^2 on all data by full-matrix least squares with SHELXL-2017 (41) following established refinement strategies (42). All non-hydrogen atoms were refined anisotropically. All hydrogen atoms were included into the model at geometrically calculated positions and refined using a riding model. The isotropic displacement parameters of all hydrogen atoms were fixed to 1.2 times the U-value of the atoms they are linked to (1.5 times for methyl groups). Details of the data quality and a summary of the residual values of the refinement are listed in tables S1, S3, and S5. Tables S2, S4, and S6 give all bond lengths and angles for the structures.

Compound Na[**8**] crystallizes in the monoclinic centrosymmetric space group $P2_1/c$ with two molecules of Na[**8**] and one half molecule of diethyl ether per asymmetric unit. The additional half molecule of Et_2O is located on a crystallographic inversion center and disordered accordingly. This was addressed by reducing the occupancy of all ether atoms to 50% while suppressing entries into the connectivity array of bonds between those atoms and their symmetry equivalents. In addition, both diethyl ether molecules coordinating to sodium cations in one of the two crystallographically independent molecules are disordered over two positions. Disorders were refined with the help of similarity restraints on $1-2$ and $1-3$ distances and displacement parameters. The disorder ratios of the coordinated ether molecules were refined freely and converged at 0.709(8) and 0.776(8), respectively. The crystal at hand was found to be twinned by pseudo-merohedry. The twin law corresponds to a 180° rotation about the

crystallographic a-axis and the twin ratio refined to 0.4303(8). As is often needed with twinned structures and always for disorders, similarity restraints on displacement parameters as well as rigid bond restraints for anisotropic displacement parameters were applied to all atoms to stabilize the refinement. The thermal ellipsoid plot is shown in Fig. S68.

Compound **9** crystallizes in the triclinic centrosymmetric space group $P\bar{1}$ with one molecule of **9** per asymmetric unit. The crystal quality was only mediocre; however structure determination was straightforward and no restraints were applied. The thermal ellipsoid plot is shown in Fig. S69.

Compound **1** crystallizes in the monoclinic centrosymmetric space group $P2_1/n$ with one molecule of **1** per asymmetric unit. In spite of the low crystal and data quality, structure determination was surprisingly straightforward. To stabilize the refinement and to counteract the low data-to-parameter ratio resulting from the low resolution of the data, similarity restraints on displacement parameters as well as rigid bond restraints for anisotropic displacement parameters were applied to all atoms. The thermal ellipsoid plot is shown in Fig. S70.

Table S1. Crystallographic data for Na[8].

Reciprocal Net code / CCDC	X8_19022 / CCDC 1928412
Empirical formula, FW (g/mol)	$C_{41}H_{47.50}BNaO_{2.25}P$, 641.06
Color / Morphology	Colourless / Plate
Crystal size (mm^3)	0.400 × 0.150 × 0.080
Temperature (K)	100(2)
Wavelength (Å)	0.71073
Crystal system, Space group	Monoclinic, $P2_1/c$
Unit cell dimensions (Å, °)	a = 19.3402(16), α = 90
	b = 18.1077(15), β = 90.108(5)
	c = 20.7944(17), γ = 90
Volume (Å^3)	7282.3(10)
Z	8
Density (calc., g/cm^3)	1.169
Absorption coefficient (mm^{-1})	0.122
$F(000)$	2740
Theta range for data collection (°)	1.053 to 23.391
Index ranges	-21 ≤ h ≤ 21, -20 ≤ k ≤ 20,
	-23 ≤ l ≤ 23
Reflections collected	158459
Independent reflections, R_{int}	10569, 0.1239
Completeness to $\theta_{\max}$ (%)	99.6
Absorption correction	Semi-empirical from equivalents
Refinement method	Full-matrix least-squares on F^2
Data / Restraints / Parameters	10569 / 2022 / 953
Goodness-of-fit[a]	1.053
Final R indices[b] $[I > 2\sigma(I)]$	$R_1 = 0.0477$, $wR_2 = 0.0983$
R indices[b] (all data)	$R_1 = 0.0716$, $wR_2 = 0.1110$
Largest diff. peak and hole (e·Å^{-3})	0.218 and −0.406

[a] GooF = $\sqrt{\frac{\Sigma[w(F_o^2-F_c^2)^2]}{(n-p)}}$ [b] $R_1 = \frac{\Sigma||F_o|-|F_c||}{\Sigma|F_o|}$; $wR_2 = \sqrt{\frac{\Sigma[w(F_o^2-F_c^2)^2}{\Sigma[w(F_o^2)^2]}}$; $w = \frac{1}{\sigma^2(F_o^2)+(aP)^2+bP}$; $P = \frac{2F_c^2+\max(F_o^2,0)}{3}$

Table S2. Bond lengths (Å) and angles (°) for Na[8].

B(1)-C(21)	1.626(5)	C(81)-H(81A)	0.99
B(1)-C(15)	1.629(6)	C(81)-H(81B)	0.99
B(1)-C(27)	1.634(6)	O(5)-C(83)	1.419(11)
B(1)-P(1)	2.015(4)	C(83)-C(84)	1.511(12)
P(1)-C(8)	1.902(3)	C(83)-H(83A)	0.99
P(1)-C(1)	1.904(4)	C(83)-H(83B)	0.99
P(1)-Na(1)	2.8323(19)	C(84)-H(84A)	0.98
C(1)-C(2)	1.512(5)	C(84)-H(84B)	0.98
C(1)-C(14)	1.516(5)	C(84)-H(84C)	0.98
C(1)-H(1)	1	C(21)-B(1)-C(15)	115.9(3)
C(2)-C(3)	1.373(5)	C(21)-B(1)-C(27)	105.5(3)
C(2)-C(7)	1.401(5)	C(15)-B(1)-C(27)	114.7(3)
C(3)-C(4)	1.387(6)	C(21)-B(1)-P(1)	115.3(3)
C(3)-H(3)	0.95	C(15)-B(1)-P(1)	100.1(2)
C(4)-C(5)	1.369(7)	C(27)-B(1)-P(1)	105.1(2)
C(4)-H(4)	0.95	C(8)-P(1)-C(1)	79.27(16)
C(5)-C(6)	1.409(6)	C(8)-P(1)-B(1)	115.73(17)
C(5)-H(5)	0.95	C(1)-P(1)-B(1)	116.37(17)
C(6)-C(7)	1.384(6)	C(8)-P(1)-Na(1)	126.44(12)
C(6)-H(6)	0.95	C(1)-P(1)-Na(1)	125.60(12)
C(7)-C(8)	1.516(5)	B(1)-P(1)-Na(1)	95.45(13)
C(8)-C(9)	1.524(5)	C(2)-C(1)-C(14)	104.8(3)
C(8)-H(8)	1	C(2)-C(1)-P(1)	102.9(2)
C(9)-C(10)	1.371(5)	C(14)-C(1)-P(1)	98.3(2)
C(9)-C(14)	1.407(5)	C(2)-C(1)-H(1)	116.2
C(10)-C(11)	1.402(6)	C(14)-C(1)-H(1)	116.2
C(10)-H(10)	0.95	P(1)-C(1)-H(1)	116.2
C(11)-C(12)	1.377(6)	C(3)-C(2)-C(7)	120.9(4)
C(11)-H(11)	0.95	C(3)-C(2)-C(1)	129.8(4)
C(12)-C(13)	1.396(6)	C(7)-C(2)-C(1)	109.2(3)
C(12)-H(12)	0.95	C(2)-C(3)-C(4)	119.0(4)
C(13)-C(14)	1.374(5)	C(2)-C(3)-H(3)	120.5
C(13)-H(13)	0.95	C(4)-C(3)-H(3)	120.5
C(15)-C(16)	1.400(5)	C(5)-C(4)-C(3)	120.4(4)
C(15)-C(20)	1.406(5)	C(5)-C(4)-H(4)	119.8
C(16)-C(17)	1.388(5)	C(3)-C(4)-H(4)	119.8
C(16)-H(16)	0.95	C(4)-C(5)-C(6)	121.8(4)
C(17)-C(18)	1.380(5)	C(4)-C(5)-H(5)	119.1
C(17)-H(17)	0.95	C(6)-C(5)-H(5)	119.1
C(18)-C(19)	1.378(6)	C(7)-C(6)-C(5)	117.3(4)
C(18)-H(18)	0.95	C(7)-C(6)-H(6)	121.4
C(19)-C(20)	1.384(5)	C(5)-C(6)-H(6)	121.4

C(19)-H(19)	0.95	C(6)-C(7)-C(2)	120.7(4)
C(20)-H(20)	0.95	C(6)-C(7)-C(8)	128.9(4)
C(21)-C(22)	1.385(5)	C(2)-C(7)-C(8)	110.4(3)
C(21)-C(26)	1.392(5)	C(7)-C(8)-C(9)	104.6(3)
C(22)-C(23)	1.388(5)	C(7)-C(8)-P(1)	102.2(2)
C(22)-H(22)	0.95	C(9)-C(8)-P(1)	97.2(2)
C(23)-C(24)	1.372(6)	C(7)-C(8)-H(8)	116.7
C(23)-H(23)	0.95	C(9)-C(8)-H(8)	116.7
C(24)-C(25)	1.374(6)	P(1)-C(8)-H(8)	116.7
C(24)-H(24)	0.95	C(10)-C(9)-C(14)	120.8(3)
C(25)-C(26)	1.387(5)	C(10)-C(9)-C(8)	129.1(3)
C(25)-H(25)	0.95	C(14)-C(9)-C(8)	110.1(3)
C(26)-H(26)	0.95	C(9)-C(10)-C(11)	118.2(4)
C(27)-C(28)	1.397(5)	C(9)-C(10)-H(10)	120.9
C(27)-C(32)	1.408(5)	C(11)-C(10)-H(10)	120.9
C(28)-C(29)	1.401(6)	C(12)-C(11)-C(10)	121.0(4)
C(28)-Na(1)	3.074(4)	C(12)-C(11)-H(11)	119.5
C(28)-H(28)	0.95	C(10)-C(11)-H(11)	119.5
C(29)-C(30)	1.387(6)	C(11)-C(12)-C(13)	120.7(4)
C(29)-H(29)	0.95	C(11)-C(12)-H(12)	119.6
C(30)-C(31)	1.376(5)	C(13)-C(12)-H(12)	119.6
C(30)-H(30)	0.95	C(14)-C(13)-C(12)	118.5(4)
C(31)-C(32)	1.392(5)	C(14)-C(13)-H(13)	120.8
C(31)-H(31)	0.95	C(12)-C(13)-H(13)	120.8
C(32)-H(32)	0.95	C(13)-C(14)-C(9)	120.8(4)
Na(1)-O(2A)	2.00(2)	C(13)-C(14)-C(1)	130.1(4)
Na(1)-O(1)	2.310(5)	C(9)-C(14)-C(1)	109.1(3)
Na(1)-O(2)	2.318(5)	C(16)-C(15)-C(20)	114.7(3)
Na(1)-O(1A)	2.364(14)	C(16)-C(15)-B(1)	122.1(3)
Na(1)-C(39A)	2.93(2)	C(20)-C(15)-B(1)	123.0(3)
Na(1)-C(37A)	3.09(2)	C(17)-C(16)-C(15)	123.0(4)
C(34)-C(33)	1.523(9)	C(17)-C(16)-H(16)	118.5
C(34)-H(34A)	0.98	C(15)-C(16)-H(16)	118.5
C(34)-H(34B)	0.98	C(18)-C(17)-C(16)	120.8(4)
C(34)-H(34C)	0.98	C(18)-C(17)-H(17)	119.6
C(33)-O(1)	1.439(7)	C(16)-C(17)-H(17)	119.6
C(33)-H(33A)	0.99	C(19)-C(18)-C(17)	117.8(4)
C(33)-H(33B)	0.99	C(19)-C(18)-H(18)	121.1
O(1)-C(35)	1.439(9)	C(17)-C(18)-H(18)	121.1
C(35)-C(36)	1.534(9)	C(18)-C(19)-C(20)	121.6(4)
C(35)-H(35A)	0.99	C(18)-C(19)-H(19)	119.2
C(35)-H(35B)	0.99	C(20)-C(19)-H(19)	119.2
C(36)-H(36A)	0.98	C(19)-C(20)-C(15)	122.2(4)
C(36)-H(36B)	0.98	C(19)-C(20)-H(20)	118.9

C(36)-H(36C)	0.98	C(15)-C(20)-H(20)	118.9
C(34A)-C(33A)	1.513(14)	C(22)-C(21)-C(26)	115.7(3)
C(34A)-H(34D)	0.98	C(22)-C(21)-B(1)	120.0(3)
C(34A)-H(34E)	0.98	C(26)-C(21)-B(1)	124.3(3)
C(34A)-H(34F)	0.98	C(21)-C(22)-C(23)	123.6(4)
C(33A)-O(1A)	1.437(14)	C(21)-C(22)-H(22)	118.2
C(33A)-H(33C)	0.99	C(23)-C(22)-H(22)	118.2
C(33A)-H(33D)	0.99	C(24)-C(23)-C(22)	118.7(4)
O(1A)-C(35A)	1.424(13)	C(24)-C(23)-H(23)	120.6
C(35A)-C(36A)	1.519(14)	C(22)-C(23)-H(23)	120.6
C(35A)-H(35C)	0.99	C(23)-C(24)-C(25)	119.8(4)
C(35A)-H(35D)	0.99	C(23)-C(24)-H(24)	120.1
C(36A)-H(36D)	0.98	C(25)-C(24)-H(24)	120.1
C(36A)-H(36E)	0.98	C(24)-C(25)-C(26)	120.4(4)
C(36A)-H(36F)	0.98	C(24)-C(25)-H(25)	119.8
C(38)-C(37)	1.510(8)	C(26)-C(25)-H(25)	119.8
C(38)-H(38A)	0.98	C(25)-C(26)-C(21)	121.7(4)
C(38)-H(38B)	0.98	C(25)-C(26)-H(26)	119.1
C(38)-H(38C)	0.98	C(21)-C(26)-H(26)	119.1
C(37)-O(2)	1.443(7)	C(28)-C(27)-C(32)	114.5(4)
C(37)-H(37A)	0.99	C(28)-C(27)-B(1)	124.3(3)
C(37)-H(37B)	0.99	C(32)-C(27)-B(1)	120.8(3)
O(2)-C(39)	1.432(7)	C(27)-C(28)-C(29)	123.2(4)
C(39)-C(40)	1.496(9)	C(27)-C(28)-Na(1)	87.2(2)
C(39)-H(39A)	0.99	C(29)-C(28)-Na(1)	113.3(3)
C(39)-H(39B)	0.99	C(27)-C(28)-H(28)	118.4
C(40)-H(40A)	0.98	C(29)-C(28)-H(28)	118.4
C(40)-H(40B)	0.98	Na(1)-C(28)-H(28)	68.7
C(40)-H(40C)	0.98	C(30)-C(29)-C(28)	120.4(4)
C(38A)-C(37A)	1.502(14)	C(30)-C(29)-H(29)	119.8
C(38A)-H(38D)	0.98	C(28)-C(29)-H(29)	119.8
C(38A)-H(38E)	0.98	C(31)-C(30)-C(29)	118.0(4)
C(38A)-H(38F)	0.98	C(31)-C(30)-H(30)	121
C(37A)-O(2A)	1.424(14)	C(29)-C(30)-H(30)	121
C(37A)-H(37C)	0.99	C(30)-C(31)-C(32)	121.2(4)
C(37A)-H(37D)	0.99	C(30)-C(31)-H(31)	119.4
O(2A)-C(39A)	1.426(14)	C(32)-C(31)-H(31)	119.4
C(39A)-C(40A)	1.505(15)	C(31)-C(32)-C(27)	122.8(4)
C(39A)-H(39C)	0.99	C(31)-C(32)-H(32)	118.6
C(39A)-H(39D)	0.99	C(27)-C(32)-H(32)	118.6
C(40A)-H(40D)	0.98	O(1)-Na(1)-O(2)	116.6(2)
C(40A)-H(40E)	0.98	O(2A)-Na(1)-O(1A)	119.5(9)
C(40A)-H(40F)	0.98	O(2A)-Na(1)-P(1)	106.2(7)
B(2)-C(55)	1.632(6)	O(1)-Na(1)-P(1)	117.97(15)

B(2)-C(61)	1.638(5)	O(2)-Na(1)-P(1)	111.80(16)
B(2)-C(67)	1.642(6)	O(1A)-Na(1)-P(1)	126.4(3)
B(2)-P(2)	2.029(4)	O(2A)-Na(1)-C(39A)	25.9(5)
P(2)-C(48)	1.900(4)	O(1A)-Na(1)-C(39A)	119.5(5)
P(2)-C(41)	1.909(4)	P(1)-Na(1)-C(39A)	90.2(4)
P(2)-Na(2)	2.8452(17)	O(2A)-Na(1)-C(28)	148.3(8)
C(41)-C(54)	1.508(5)	O(1)-Na(1)-C(28)	96.16(16)
C(41)-C(42)	1.523(5)	O(2)-Na(1)-C(28)	141.36(18)
C(41)-H(41)	1	O(1A)-Na(1)-C(28)	86.8(4)
C(42)-C(43)	1.389(5)	P(1)-Na(1)-C(28)	63.91(8)
C(42)-C(47)	1.392(5)	C(39A)-Na(1)-C(28)	151.7(4)
C(43)-C(44)	1.403(5)	O(2A)-Na(1)-C(37A)	21.4(5)
C(43)-H(43)	0.95	O(1A)-Na(1)-C(37A)	113.8(6)
C(44)-C(45)	1.370(5)	P(1)-Na(1)-C(37A)	118.8(5)
C(44)-H(44)	0.95	C(39A)-Na(1)-C(37A)	47.3(5)
C(45)-C(46)	1.407(5)	C(28)-Na(1)-C(37A)	134.8(4)
C(45)-H(45)	0.95	C(33)-C(34)-H(34A)	109.5
C(46)-C(47)	1.376(5)	C(33)-C(34)-H(34B)	109.5
C(46)-H(46)	0.95	H(34A)-C(34)-H(34B)	109.5
C(47)-C(48)	1.523(5)	C(33)-C(34)-H(34C)	109.5
C(48)-C(49)	1.531(5)	H(34A)-C(34)-H(34C)	109.5
C(48)-H(48)	1	H(34B)-C(34)-H(34C)	109.5
C(49)-C(54)	1.383(5)	O(1)-C(33)-C(34)	110.0(6)
C(49)-C(50)	1.386(5)	O(1)-C(33)-H(33A)	109.7
C(50)-C(51)	1.389(6)	C(34)-C(33)-H(33A)	109.7
C(50)-H(50)	0.95	O(1)-C(33)-H(33B)	109.7
C(51)-C(52)	1.372(7)	C(34)-C(33)-H(33B)	109.7
C(51)-H(51)	0.95	H(33A)-C(33)-H(33B)	108.2
C(52)-C(53)	1.383(7)	C(35)-O(1)-C(33)	110.5(6)
C(52)-H(52)	0.95	C(35)-O(1)-Na(1)	115.7(5)
C(53)-C(54)	1.384(5)	C(33)-O(1)-Na(1)	128.6(4)
C(53)-H(53)	0.95	O(1)-C(35)-C(36)	108.9(7)
C(55)-C(60)	1.392(5)	O(1)-C(35)-H(35A)	109.9
C(55)-C(56)	1.394(5)	C(36)-C(35)-H(35A)	109.9
C(56)-C(57)	1.396(5)	O(1)-C(35)-H(35B)	109.9
C(56)-H(56)	0.95	C(36)-C(35)-H(35B)	109.9
C(57)-C(58)	1.374(6)	H(35A)-C(35)-H(35B)	108.3
C(57)-H(57)	0.95	C(35)-C(36)-H(36A)	109.5
C(58)-C(59)	1.379(6)	C(35)-C(36)-H(36B)	109.5
C(58)-H(58)	0.95	H(36A)-C(36)-H(36B)	109.5
C(59)-C(60)	1.401(5)	C(35)-C(36)-H(36C)	109.5
C(59)-H(59)	0.95	H(36A)-C(36)-H(36C)	109.5
C(60)-H(60)	0.95	H(36B)-C(36)-H(36C)	109.5
C(61)-C(66)	1.379(5)	C(33A)-C(34A)-H(34D)	109.5

C(61)-C(62)	1.402(5)	C(33A)-C(34A)-H(34E)	109.5
C(62)-C(63)	1.384(5)	H(34D)-C(34A)-H(34E)	109.5
C(62)-H(62)	0.95	C(33A)-C(34A)-H(34F)	109.5
C(63)-C(64)	1.368(5)	H(34D)-C(34A)-H(34F)	109.5
C(63)-H(63)	0.95	H(34E)-C(34A)-H(34F)	109.5
C(64)-C(65)	1.367(5)	O(1A)-C(33A)-C(34A)	109.2(16)
C(64)-H(64)	0.95	O(1A)-C(33A)-H(33C)	109.8
C(65)-C(66)	1.390(5)	C(34A)-C(33A)-H(33C)	109.8
C(65)-H(65)	0.95	O(1A)-C(33A)-H(33D)	109.8
C(66)-H(66)	0.95	C(34A)-C(33A)-H(33D)	109.8
C(67)-C(68)	1.389(5)	H(33C)-C(33A)-H(33D)	108.3
C(67)-C(72)	1.404(5)	C(35A)-O(1A)-C(33A)	112.2(14)
C(67)-Na(2)	2.938(4)	C(35A)-O(1A)-Na(1)	114.2(9)
C(68)-C(69)	1.381(5)	C(33A)-O(1A)-Na(1)	132.8(13)
C(68)-Na(2)	3.086(4)	O(1A)-C(35A)-C(36A)	109.7(14)
C(68)-H(68)	0.95	O(1A)-C(35A)-H(35C)	109.7
C(69)-C(70)	1.384(6)	C(36A)-C(35A)-H(35C)	109.7
C(69)-H(69)	0.95	O(1A)-C(35A)-H(35D)	109.7
C(70)-C(71)	1.363(6)	C(36A)-C(35A)-H(35D)	109.7
C(70)-H(70)	0.95	H(35C)-C(35A)-H(35D)	108.2
C(71)-C(72)	1.385(5)	C(35A)-C(36A)-H(36D)	109.5
C(71)-H(71)	0.95	C(35A)-C(36A)-H(36E)	109.5
C(72)-H(72)	0.95	H(36D)-C(36A)-H(36E)	109.5
Na(2)-O(3)	2.235(3)	C(35A)-C(36A)-H(36F)	109.5
Na(2)-O(4)	2.286(3)	H(36D)-C(36A)-H(36F)	109.5
Na(2)-C(74)	3.054(5)	H(36E)-C(36A)-H(36F)	109.5
C(74)-C(73)	1.492(6)	C(37)-C(38)-H(38A)	109.5
C(74)-H(74A)	0.98	C(37)-C(38)-H(38B)	109.5
C(74)-H(74B)	0.98	H(38A)-C(38)-H(38B)	109.5
C(74)-H(74C)	0.98	C(37)-C(38)-H(38C)	109.5
C(73)-O(3)	1.414(5)	H(38A)-C(38)-H(38C)	109.5
C(73)-H(73A)	0.99	H(38B)-C(38)-H(38C)	109.5
C(73)-H(73B)	0.99	O(2)-C(37)-C(38)	107.5(5)
O(3)-C(75)	1.437(5)	O(2)-C(37)-H(37A)	110.2
C(75)-C(76)	1.477(6)	C(38)-C(37)-H(37A)	110.2
C(75)-H(75A)	0.99	O(2)-C(37)-H(37B)	110.2
C(75)-H(75B)	0.99	C(38)-C(37)-H(37B)	110.2
C(76)-H(76A)	0.98	H(37A)-C(37)-H(37B)	108.5
C(76)-H(76B)	0.98	C(39)-O(2)-C(37)	111.4(5)
C(76)-H(76C)	0.98	C(39)-O(2)-Na(1)	132.0(4)
C(78)-C(77)	1.502(7)	C(37)-O(2)-Na(1)	116.1(4)
C(78)-H(78A)	0.98	O(2)-C(39)-C(40)	110.7(5)
C(78)-H(78B)	0.98	O(2)-C(39)-H(39A)	109.5
C(78)-H(78C)	0.98	C(40)-C(39)-H(39A)	109.5

C(77)-O(4)	1.426(5)	O(2)-C(39)-H(39B)	109.5
C(77)-H(77A)	0.99	C(40)-C(39)-H(39B)	109.5
C(77)-H(77B)	0.99	H(39A)-C(39)-H(39B)	108.1
O(4)-C(79)	1.440(5)	C(39)-C(40)-H(40A)	109.5
C(79)-C(80)	1.480(6)	C(39)-C(40)-H(40B)	109.5
C(79)-H(79A)	0.99	H(40A)-C(40)-H(40B)	109.5
C(79)-H(79B)	0.99	C(39)-C(40)-H(40C)	109.5
C(80)-H(80A)	0.98	H(40A)-C(40)-H(40C)	109.5
C(80)-H(80B)	0.98	H(40B)-C(40)-H(40C)	109.5
C(80)-H(80C)	0.98	C(37A)-C(38A)-H(38D)	109.5
C(82)-C(81)	1.517(11)	C(37A)-C(38A)-H(38E)	109.5
C(82)-H(82A)	0.98	H(38D)-C(38A)-H(38E)	109.5
C(82)-H(82B)	0.98	C(37A)-C(38A)-H(38F)	109.5
C(82)-H(82C)	0.98	H(38D)-C(38A)-H(38F)	109.5
C(81)-O(5)	1.424(9)	H(38E)-C(38A)-H(38F)	109.5

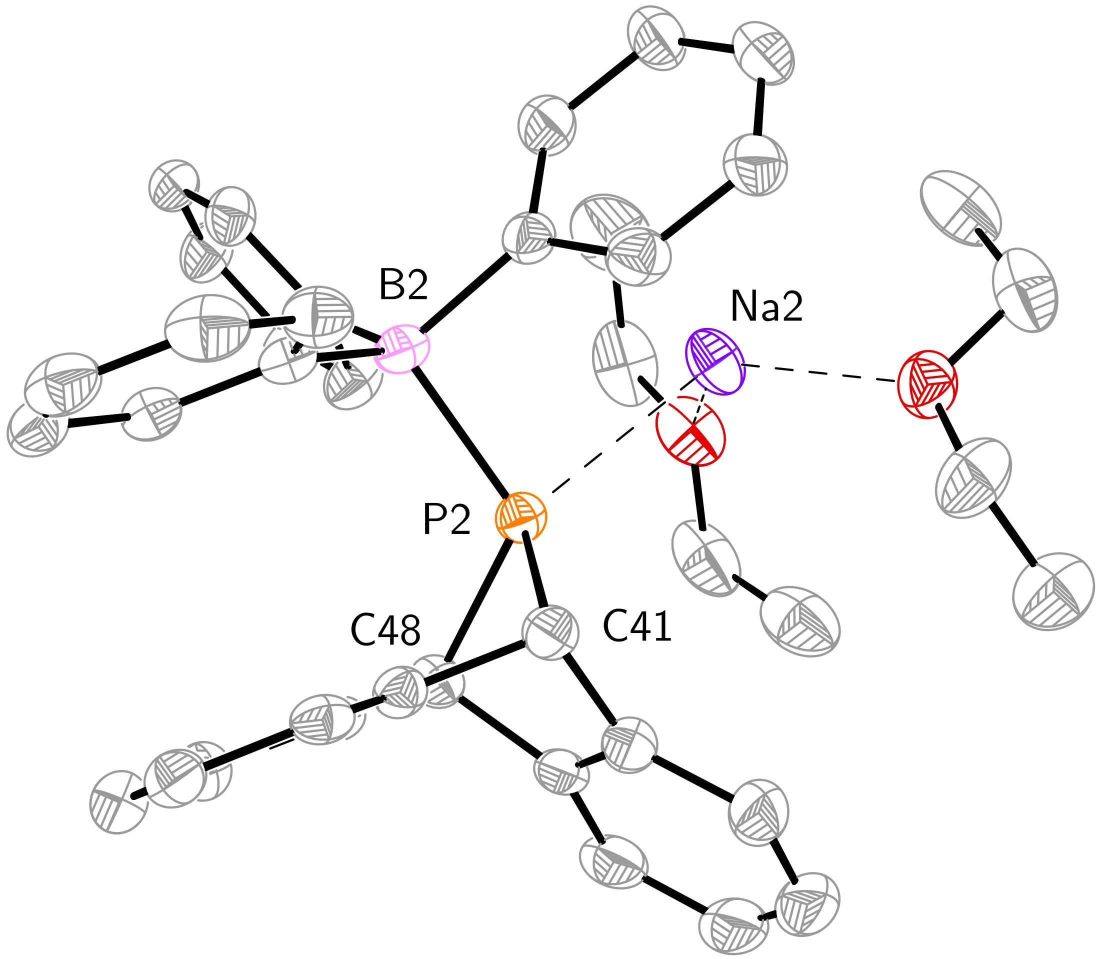

Fig. S68. Molecular structure of Na[8], with thermal ellipsoids shown at the 50% probability level and hydrogen atoms omitted for clarity.

Table S3. Crystallographic data for 9.

Reciprocal Net code / CCDC	X8_19029 / CCDC 1928418
Empirical formula, FW (g/mol)	$C_{29}H_{37}P$, 416.55
Color / Morphology	Colourless / Needle
Crystal size (mm^3)	0.543 $\times$ 0.142 $\times$ 0.087
Temperature (K)	100(2)
Wavelength (Å)	0.71073
Crystal system, Space group	Triclinic, $P\bar{1}$
Unit cell dimensions (Å, °)	a = 8.969(16), α = 84.23(8)
	b = 9.60(2), β = 81.18(6)
	c = 17.08(3), γ = 62.32(9)
Volume (Å^3)	1286(4)
Z	2
Density (calc., g/cm^3)	1.076
Absorption coefficient (mm^{-1})	0.119
F(000)	452
Theta range for data collection (°)	2.398 to 29.134
Index ranges	-11 $\leq$ h $\leq$ 11, -12 $\leq$ k $\leq$ 12,
	-22 $\leq$ l $\leq$ 22
Reflections collected	65555
Independent reflections, R_{int}	5884, 0.0919
Completeness to θ_{max} (%)	99.8
Absorption correction	Semi-empirical from equivalents
Refinement method	Full-matrix least-squares on F^2
Data / Restraints / Parameters	5884 / 0 / 280
Goodness-of-fit[a]	1.080
Final R indices[b] $[I > 2\sigma(I)]$	$R_1 = 0.0634$, $wR_2 = 0.1761$
R indices[b] (all data)	$R_1 = 0.0727$, $wR_2 = 0.1858$
Largest diff. peak and hole (e·Å^{-3})	0.850 and -0.594

[a] GooF = $\sqrt{\frac{\Sigma[w(F_o^2-F_c^2)^2]}{(n-p)}}$ [b] $R_1 = \frac{\Sigma||F_o|-|F_c||}{\Sigma|F_o|}$; $wR_2 = \sqrt{\frac{\Sigma[w(F_o^2-F_c^2)^2}{\Sigma[w(F_o^2)^2]}}$; $w = \frac{1}{\sigma^2(F_o^2)+(aP)^2+bP}$; $P = \frac{2F_c^2+\max(F_o^2,0)}{3}$

Table S4. Bond lengths (Å) and angles (°) for 9.

P(1)-C(15)	1.907(3)	C(6)-C(7)-C(8)	130.2(2)
P(1)-C(1)	1.950(3)	C(14)-C(9)-C(10)	121.4(2)
P(1)-C(8)	1.972(4)	C(14)-C(9)-C(8)	108.1(2)
C(1)-C(2)	1.546(4)	C(10)-C(9)-C(8)	130.46(19)
C(1)-C(14)	1.555(4)	C(11)-C(10)-C(9)	119.4(2)
C(1)-H(1)	1	C(11)-C(10)-H(10)	120.3
C(2)-C(7)	1.386(3)	C(9)-C(10)-H(10)	120.3
C(2)-C(3)	1.418(3)	C(14)-C(13)-C(12)	119.5(2)
C(3)-C(4)	1.422(4)	C(14)-C(13)-H(13)	120.2
C(3)-H(3)	0.95	C(12)-C(13)-H(13)	120.2
C(4)-C(5)	1.371(4)	C(11)-C(12)-C(13)	121.6(2)
C(4)-H(4)	0.95	C(11)-C(12)-H(12)	119.2
C(5)-C(6)	1.432(3)	C(13)-C(12)-H(12)	119.2
C(5)-H(5)	0.95	C(12)-C(11)-C(10)	119.2(2)
C(6)-C(7)	1.405(4)	C(12)-C(11)-H(11)	120.4
C(6)-H(6)	0.95	C(10)-C(11)-H(11)	120.4
C(8)-C(9)	1.529(3)	C(21)-C(15)-C(17)	120.67(17)
C(8)-C(7)	1.550(3)	C(21)-C(15)-C(18)	117.52(18)
C(8)-H(8)	1	C(17)-C(15)-C(18)	50.74(14)
C(9)-C(14)	1.394(3)	C(21)-C(15)-P(1)	116.68(18)
C(9)-C(10)	1.416(4)	C(17)-C(15)-P(1)	121.49(17)
C(10)-C(11)	1.394(3)	C(18)-C(15)-P(1)	109.74(17)
C(10)-H(10)	0.95	C(13)-C(14)-C(9)	118.9(2)
C(13)-C(14)	1.382(3)	C(13)-C(14)-C(1)	130.45(19)
C(13)-C(12)	1.426(4)	C(9)-C(14)-C(1)	110.6(2)
C(13)-H(13)	0.95	C(17)-C(19)-C(191)	109.92(17)
C(12)-C(11)	1.375(4)	C(17)-C(19)-C(193)	112.27(19)
C(12)-H(12)	0.95	C(191)-C(19)-C(193)	106.42(18)
C(11)-H(11)	0.95	C(17)-C(19)-C(192)	106.7(2)
C(15)-C(21)	1.536(3)	C(191)-C(19)-C(192)	110.1(2)
C(15)-C(17)	1.540(4)	C(193)-C(19)-C(192)	111.48(19)
C(15)-C(18)	1.568(4)	C(202)-C(20)-C(18)	104.49(19)
C(19)-C(17)	1.529(3)	C(202)-C(20)-C(203)	109.20(18)
C(19)-C(191)	1.537(4)	C(18)-C(20)-C(203)	111.88(19)
C(19)-C(193)	1.553(4)	C(202)-C(20)-C(201)	110.6(2)
C(19)-C(192)	1.597(4)	C(18)-C(20)-C(201)	112.79(19)
C(20)-C(202)	1.526(4)	C(203)-C(20)-C(201)	107.9(2)
C(20)-C(18)	1.547(4)	C(15)-C(21)-C(213)	110.74(17)
C(20)-C(203)	1.548(4)	C(15)-C(21)-C(212)	108.21(18)
C(20)-C(201)	1.595(4)	C(213)-C(21)-C(212)	108.8(2)
C(21)-C(213)	1.555(4)	C(15)-C(21)-C(211)	110.4(2)
C(21)-C(212)	1.576(5)	C(213)-C(21)-C(211)	107.2(2)

C(21)-C(211)	1.593(4)	C(212)-C(21)-C(211)	111.50(18)
C(191)-H(19A)	0.98	C(19)-C(191)-H(19A)	109.5
C(191)-H(19B)	0.98	C(19)-C(191)-H(19B)	109.5
C(191)-H(19C)	0.98	H(19A)-C(191)-H(19B)	109.5
C(192)-H(19D)	0.98	C(19)-C(191)-H(19C)	109.5
C(192)-H(19E)	0.98	H(19A)-C(191)-H(19C)	109.5
C(192)-H(19F)	0.98	H(19B)-C(191)-H(19C)	109.5
C(193)-H(19G)	0.98	C(19)-C(192)-H(19D)	109.5
C(193)-H(19H)	0.98	C(19)-C(192)-H(19E)	109.5
C(193)-H(19I)	0.98	H(19D)-C(192)-H(19E)	109.5
C(201)-H(20A)	0.98	C(19)-C(192)-H(19F)	109.5
(201)-H(20B)	0.98	H(19D)-C(192)-H(19F)	109.5
C(201)-H(20C)	0.98	H(19E)-C(192)-H(19F)	109.5
C(202)-H(20D)	0.98	C(19)-C(193)-H(19G)	109.5
C(202)-H(20E)	0.98	C(19)-C(193)-H(19H)	109.5
C(202)-H(20F)	0.98	H(19G)-C(193)-H(19H)	109.5
C(203)-H(20G)	0.98	C(19)-C(193)-H(19I)	109.5
C(203)-H(20H)	0.98	H(19G)-C(193)-H(19I)	109.5
C(203)-H(20I)	0.98	H(19H)-C(193)-H(19I)	109.5
C(211)-H(21A)	0.98	C(20)-C(201)-H(20A)	109.5
C(211)-H(21B)	0.98	C(20)-C(201)-H(20B)	109.5
C(211)-H(21C)	0.98	H(20A)-C(201)-H(20B)	109.5
C(212)-H(21D)	0.98	C(20)-C(201)-H(20C)	109.5
C(212)-H(21E)	0.98	H(20A)-C(201)-H(20C)	109.5
C(212)-H(21F)	0.98	H(20B)-C(201)-H(20C)	109.5
C(213)-H(21G)	0.98	C(20)-C(202)-H(20D)	109.5
C(213)-H(21H)	0.98	C(20)-C(202)-H(20E)	109.5
C(213)-H(21I)	0.98	H(20D)-C(202)-H(20E)	109.5
C(18)-C(17)	1.332(3)	C(20)-C(202)-H(20F)	109.5
C(15)-P(1)-C(1)	110.66(14)	H(20D)-C(202)-H(20F)	109.5
C(15)-P(1)-C(8)	116.94(14)	H(20E)-C(202)-H(20F)	109.5
C(1)-P(1)-C(8)	76.09(14)	C(20)-C(203)-H(20G)	109.5
C(2)-C(1)-C(14)	103.14(18)	C(20)-C(203)-H(20H)	109.5
C(2)-C(1)-P(1)	107.82(15)	H(20G)-C(203)-H(20H)	109.5
C(14)-C(1)-P(1)	95.43(16)	C(20)-C(203)-H(20I)	109.5
C(2)-C(1)-H(1)	116	H(20G)-C(203)-H(20I)	109.5
C(14)-C(1)-H(1)	116	H(20H)-C(203)-H(20I)	109.5
P(1)-C(1)-H(1)	116	C(21)-C(211)-H(21A)	109.5
C(7)-C(2)-C(3)	119.9(2)	C(21)-C(211)-H(21B)	109.5
C(7)-C(2)-C(1)	108.92(19)	H(21A)-C(211)-H(21B)	109.5
C(3)-C(2)-C(1)	130.83(19)	C(21)-C(211)-H(21C)	109.5
C(2)-C(3)-C(4)	120.5(2)	H(21A)-C(211)-H(21C)	109.5
C(2)-C(3)-H(3)	119.7	H(21B)-C(211)-H(21C)	109.5
C(4)-C(3)-H(3)	119.7	C(21)-C(212)-H(21D)	109.5

C(5)-C(4)-C(3)	119.4(2)	C(21)-C(212)-H(21E)	109.5
C(5)-C(4)-H(4)	120.3	H(21D)-C(212)-H(21E)	109.5
C(3)-C(4)-H(4)	120.3	C(21)-C(212)-H(21F)	109.5
C(4)-C(5)-C(6)	120.1(2)	H(21D)-C(212)-H(21F)	109.5
C(4)-C(5)-H(5)	120	H(21E)-C(212)-H(21F)	109.5
C(6)-C(5)-H(5)	120	C(21)-C(213)-H(21G)	109.5
C(7)-C(6)-C(5)	120.4(2)	C(21)-C(213)-H(21H)	109.5
C(7)-C(6)-H(6)	119.8	H(21G)-C(213)-H(21H)	109.5
C(5)-C(6)-H(6)	119.8	C(21)-C(213)-H(21I)	109.5
C(9)-C(8)-C(7)	104.80(18)	H(21G)-C(213)-H(21I)	109.5
C(9)-C(8)-P(1)	96.87(17)	H(21H)-C(213)-H(21I)	109.5
C(7)-C(8)-P(1)	106.0(2)	C(17)-C(18)-C(20)	151.2(2)
C(9)-C(8)-H(8)	115.7	C(17)-C(18)-C(15)	63.5(2)
C(7)-C(8)-H(8)	115.7	C(20)-C(18)-C(15)	144.47(19)
P(1)-C(8)-H(8)	115.7	C(18)-C(17)-C(19)	146.54(19)
C(2)-C(7)-C(6)	119.6(2)	C(18)-C(17)-C(15)	65.71(18)
C(2)-C(7)-C(8)	110.0(2)	C(19)-C(17)-C(15)	147.73(19)

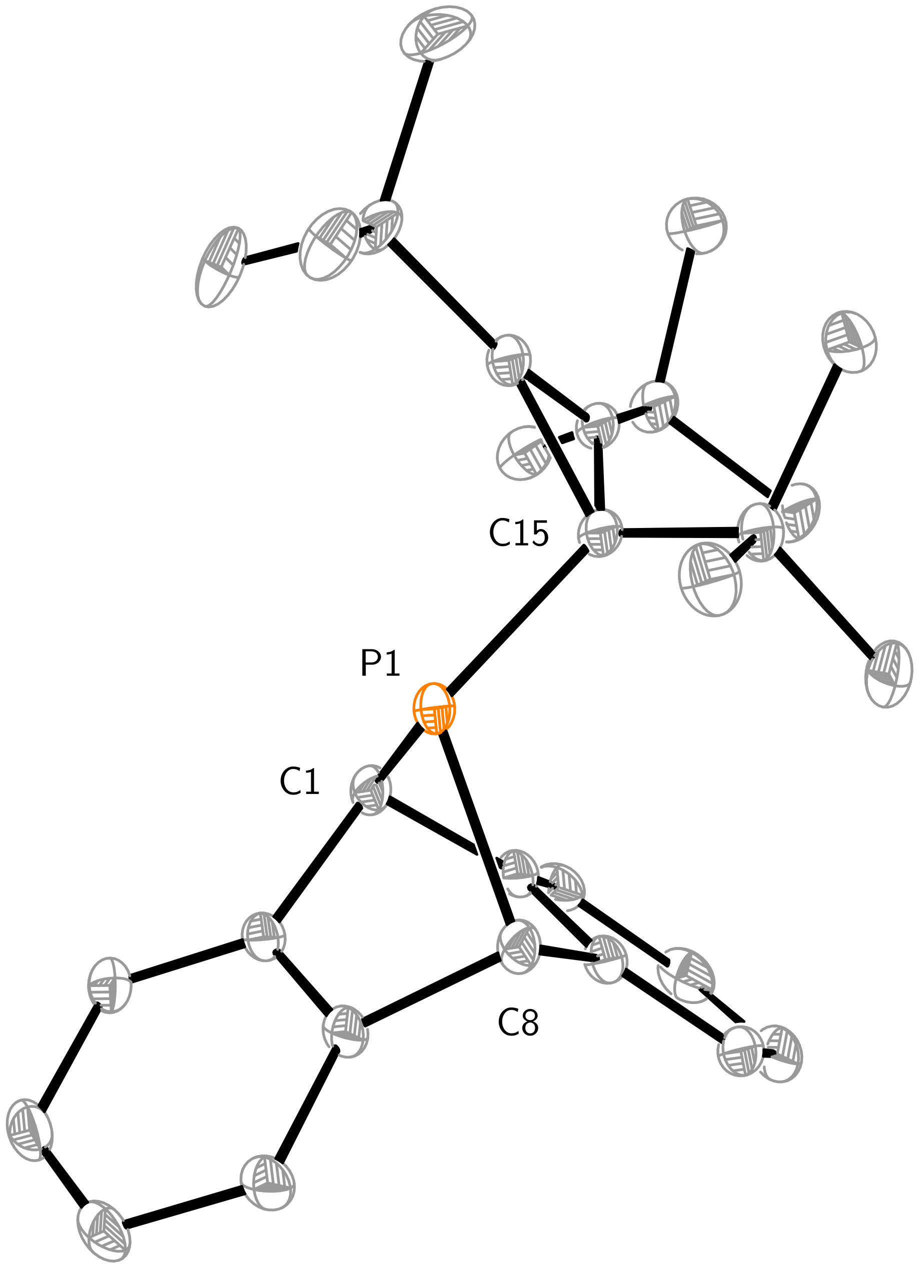

Fig. S69. Molecular structure of 9, with thermal ellipsoids shown at the 50% probability level and hydrogen atoms omitted for clarity.

Table S5. Crystallographic data for 1.

Reciprocal Net code / CCDC	P8_19077 / CCDC 1942402
Empirical formula, FW (g/mol)	$C_{15}H_{27}P$
Color / Morphology	Colourless / Needle
Crystal size (mm^3)	0.050 × 0.015 × 0.010
Temperature (K)	100(2)
Wavelength (Å)	1.54178
Crystal system, Space group	Monoclinic, $P2_1/n$
Unit cell dimensions (Å, °)	a = 8.987(8), α = 90
	b = 13.070(12), β = 91.90(5)
	c = 13.521(16), γ = 90
Volume (Å^3)	1587(3)
Z	4
Density (calc., g/cm^3)	0.997
Absorption coefficient (mm^{-1})	1.322
F(000)	528
Theta range for data collection (°)	8.389 to 53.432
Index ranges	-9 ≤ h ≤ 9, -13 ≤ k ≤ 7,
	-12 ≤ l ≤ 14
Reflections collected	3716
Independent reflections, R_{int}	1751, 0.1141
Completeness to θ_{max} (%)	92.9
Absorption correction	Semi-empirical from equivalents
Refinement method	Full-matrix least-squares on F^2
Data / Restraints / Parameters	1751 / 243 / 154
Goodness-of-fit[a]	1.436
Final R indices[b] $[I > 2\sigma(I)]$	$R_1 = 0.1256$,, $wR_2 = 0.2702$
R indices[b] (all data)	$R_1 = 0.2224$, $wR_2 = 0.3104$
Largest diff. peak and hole (e·Å^{-3})	0.451 and −0.463

[a] GooF = $\sqrt{\frac{\Sigma[w(F_o^2-F_c^2)^2]}{(n-p)}}$ [b] $R_1 = \frac{\Sigma||F_o|-|F_c||}{\Sigma|F_o|}$; $wR_2 = \sqrt{\frac{\Sigma[w(F_o^2-F_c^2)^2}{\Sigma[w(F_o^2)^2]}}$; $w = \frac{1}{\sigma^2(F_o^2)+(aP)^2+bP}$; $P = \frac{2F_c^2+\max(F_o^2,0)}{3}$

Table S6. Bond lengths (Å) and angles (°) for 1.

P(1)-C(1)	1.836(10)	C(2)-C(4)-H(4C)	109.5
P(1)-C(11)	1.840(10)	H(4A)-C(4)-H(4C)	109.5
P(1)-C(6)	1.860(10)	H(4B)-C(4)-H(4C)	109.5
C(1)-C(6)	1.467(11)	C(2)-C(5)-H(5A)	109.5
C(1)-C(11)	1.473(13)	C(2)-C(5)-H(5B)	109.5
C(1)-C(2)	1.500(13)	H(5A)-C(5)-H(5B)	109.5
C(2)-C(4)	1.513(15)	C(2)-C(5)-H(5C)	109.5
C(2)-C(3)	1.523(14)	H(5A)-C(5)-H(5C)	109.5
C(2)-C(5)	1.553(11)	H(5B)-C(5)-H(5C)	109.5
C(3)-H(3A)	0.98	C(1)-C(6)-C(11)	60.0(5)
C(3)-H(3B)	0.98	C(1)-C(6)-C(7)	145.8(8)
C(3)-H(3C)	0.98	C(11)-C(6)-C(7)	142.9(8)
C(4)-H(4A)	0.98	C(1)-C(6)-P(1)	65.8(5)
C(4)-H(4B)	0.98	C(11)-C(6)-P(1)	65.7(5)
C(4)-H(4C)	0.98	C(7)-C(6)-P(1)	138.5(6)
C(5)-H(5A)	0.98	C(9)-C(7)-C(6)	109.6(6)
C(5)-H(5B)	0.98	C(9)-C(7)-C(8)	111.0(9)
C(5)-H(5C)	0.98	C(6)-C(7)-C(8)	108.4(8)
C(6)-C(11)	1.481(11)	C(9)-C(7)-C(10)	110.6(9)
C(6)-C(7)	1.527(12)	C(6)-C(7)-C(10)	107.4(8)
C(7)-C(9)	1.501(14)	C(8)-C(7)-C(10)	109.8(6)
C(7)-C(8)	1.528(13)	C(7)-C(8)-H(8A)	109.5
C(7)-C(10)	1.547(14)	C(7)-C(8)-H(8B)	109.5
C(8)-H(8A)	0.98	H(8A)-C(8)-H(8B)	109.5
C(8)-H(8B)	0.98	C(7)-C(8)-H(8C)	109.5
C(8)-H(8C)	0.98	H(8A)-C(8)-H(8C)	109.5
C(9)-H(9A)	0.98	H(8B)-C(8)-H(8C)	109.5
C(9)-H(9B)	0.98	C(7)-C(9)-H(9A)	109.5
C(9)-H(9C)	0.98	C(7)-C(9)-H(9B)	109.5
C(10)-H(10A)	0.98	H(9A)-C(9)-H(9B)	109.5
C(10)-H(10B)	0.98	C(7)-C(9)-H(9C)	109.5
C(10)-H(10C)	0.98	H(9A)-C(9)-H(9C)	109.5
C(11)-C(12)	1.485(13)	H(9B)-C(9)-H(9C)	109.5
C(12)-C(14)	1.517(14)	C(7)-C(10)-H(10A)	109.5
C(12)-C(15)	1.522(14)	C(7)-C(10)-H(10B)	109.5
C(12)-C(13)	1.557(12)	H(10A)-C(10)-H(10B)	109.5
C(13)-H(13A)	0.98	C(7)-C(10)-H(10C)	109.5
C(13)-H(13B)	0.98	H(10A)-C(10)-H(10C)	109.5
C(13)-H(13C)	0.98	H(10B)-C(10)-H(10C)	109.5
C(14)-H(14A)	0.98	C(1)-C(11)-C(6)	59.6(6)
C(14)-H(14B)	0.98	C(1)-C(11)-C(12)	143.0(7)
C(14)-H(14C)	0.98	C(6)-C(11)-C(12)	143.9(9)

C(15)-H(15A)	0.98	C(1)-C(11)-P(1)	66.2(5)
C(15)-H(15B)	0.98	C(6)-C(11)-P(1)	67.1(5)
C(15)-H(15C)	0.98	C(12)-C(11)-P(1)	139.3(8)
C(1)-P(1)-C(11)	47.2(4)	C(11)-C(12)-C(14)	108.6(6)
C(1)-P(1)-C(6)	46.8(4)	C(11)-C(12)-C(15)	109.6(9)
C(11)-P(1)-C(6)	47.2(4)	C(14)-C(12)-C(15)	111.4(9)
C(6)-C(1)-C(11)	60.5(6)	C(11)-C(12)-C(13)	108.3(8)
C(6)-C(1)-C(2)	142.7(8)	C(14)-C(12)-C(13)	109.9(9)
C(11)-C(1)-C(2)	143.2(7)	C(15)-C(12)-C(13)	109.0(6)
C(6)-C(1)-P(1)	67.5(5)	C(12)-C(13)-H(13A)	109.5
C(11)-C(1)-P(1)	66.5(5)	C(12)-C(13)-H(13B)	109.5
C(2)-C(1)-P(1)	139.4(7)	H(13A)-C(13)-H(13B)	109.5
C(1)-C(2)-C(4)	108.6(7)	C(12)-C(13)-H(13C)	109.5
C(1)-C(2)-C(3)	112.1(8)	H(13A)-C(13)-H(13C)	109.5
C(4)-C(2)-C(3)	109.6(9)	H(13B)-C(13)-H(13C)	109.5
C(1)-C(2)-C(5)	108.4(8)	C(12)-C(14)-H(14A)	109.5
C(4)-C(2)-C(5)	108.3(8)	C(12)-C(14)-H(14B)	109.5
C(3)-C(2)-C(5)	109.8(6)	H(14A)-C(14)-H(14B)	109.5
C(2)-C(3)-H(3A)	109.5	C(12)-C(14)-H(14C)	109.5
C(2)-C(3)-H(3B)	109.5	H(14A)-C(14)-H(14C)	109.5
H(3A)-C(3)-H(3B)	109.5	H(14B)-C(14)-H(14C)	109.5
C(2)-C(3)-H(3C)	109.5	C(12)-C(15)-H(15A)	109.5
H(3A)-C(3)-H(3C)	109.5	C(12)-C(15)-H(15B)	109.5
H(3B)-C(3)-H(3C)	109.5	H(15A)-C(15)-H(15B)	109.5
C(2)-C(4)-H(4A)	109.5	C(12)-C(15)-H(15C)	109.5
C(2)-C(4)-H(4B)	109.5	H(15A)-C(15)-H(15C)	109.5
H(4A)-C(4)-H(4B)	109.5	H(15B)-C(15)-H(15C)	109.5

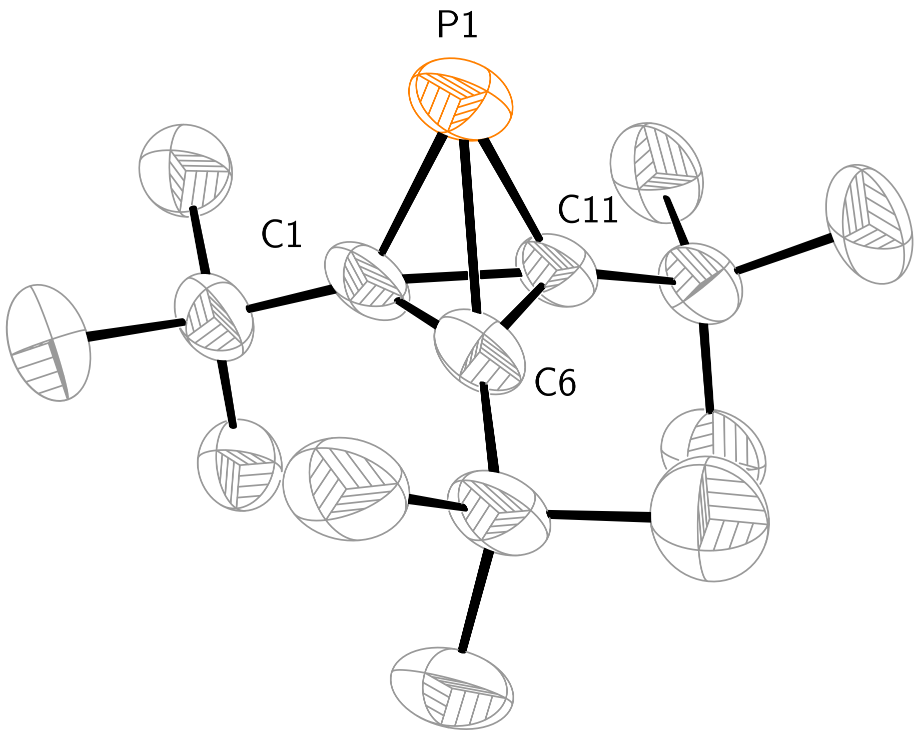

Fig. S70. Molecular structure of 1, with thermal ellipsoids shown at the 50% probability level and hydrogen atoms omitted for clarity.

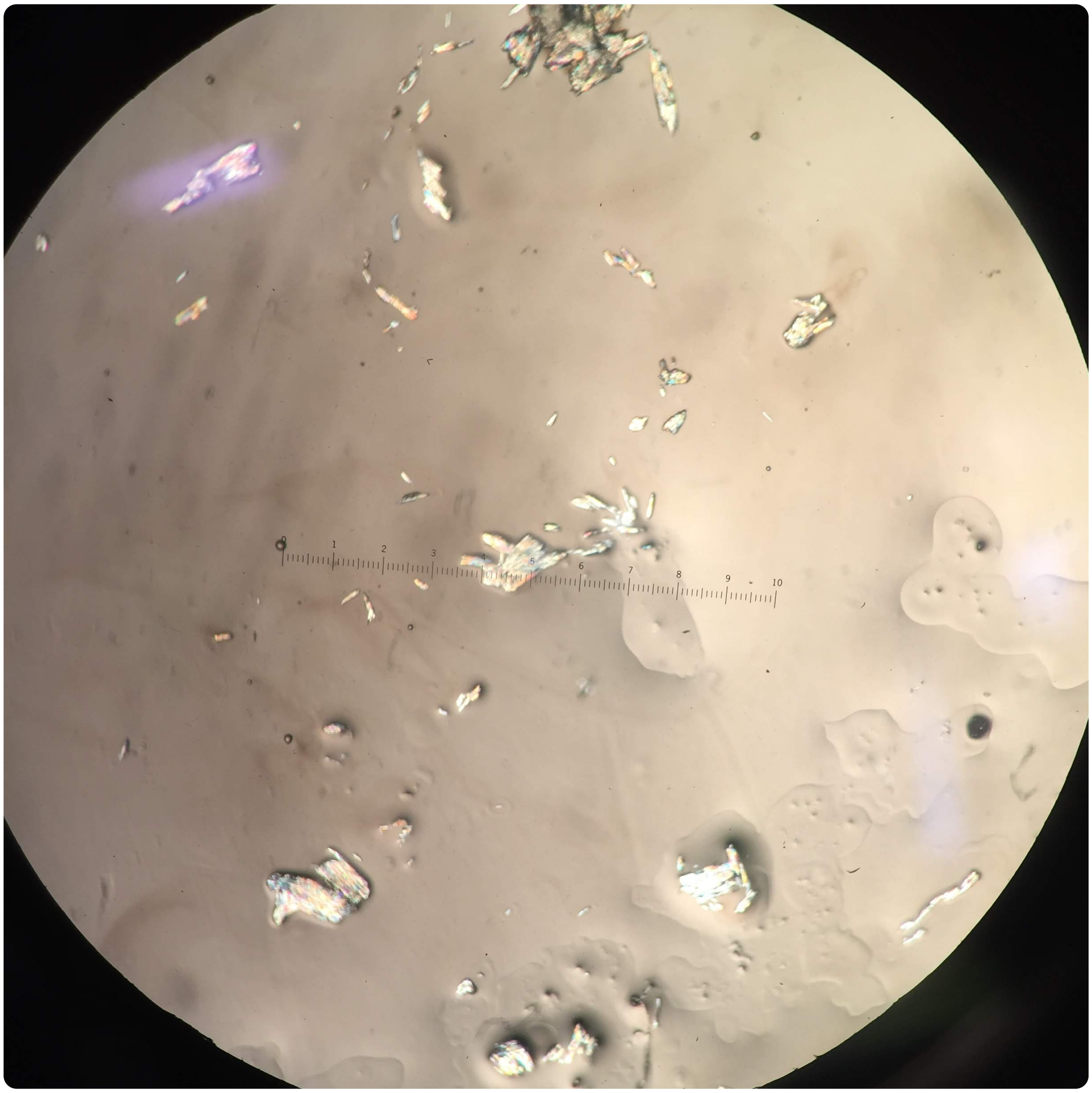

Fig. S71. Crystals of 1 grown by sublimation. (Photo Credit: Martin-Louis Y. Riu, MIT)

Section S3. Computational studies

S3.1 Predicted vibrational frequencies of (^{t}BuC)$_3$P (1)

S3.1.1 General procedure

All calculations were performed with the ORCA 4.0.1 quantum chemistry package from the development team at the University of Bonn (43). Initial geometries were constructed in Avogadro (44). Geometry optimizations were performed at the wB97X-D3/Def2-TZVPP level of theory using keywords `wB97X-D3 def2-TZVPP def2/J TightSCF RIJCOSX Grid4 FinalGrid5 Opt NumFreq`. Frequency calculations were performed to ensure a lack of imaginary frequencies. A scaling factor of 0.955 was applied to the predicted vibrational frequencies of **1** (38).

S3.1.2 Optimized coordinates

Table S7. Coordinates of 1.

Atom	x	y	z
C	-6.10855344156217	6.40588501051557	2.51667905757589
C	-4.50189068182423	4.45854339864194	2.59874979940856
C	-4.50189068182423	4.45854339864194	2.59874979940856
C	-4.88736955775334	5.74720362302510	1.85930289594924
C	-7.66486477522581	3.14305352376569	0.60857695382515
C	-3.69817567795503	6.71016546193290	1.89217828258831
C	-8.66848107985338	5.26222037970595	-0.24162883469423
C	-5.21236786397527	5.44056571074028	0.42593935425237
C	-7.57903918941800	4.21417473607217	-0.48473030231470
C	-6.22795921916441	4.86184309637044	-0.45820692005699
C	-4.80273251621919	4.63504841761628	-0.73329427200545
C	-7.79363118872361	3.60671034290170	-1.87436379116786
C	-3.79798900191821	3.63002038024641	-1.22964295554597
C	-2.38249888165479	4.15186515710283	-0.95867692680044
C	-3.96901821002181	3.44162216919757	-2.73956161453239
C	-3.98422617695565	2.27019936222841	-0.54521875310425
P	-5.38441509407812	6.33135415544918	-1.17983531072911
H	-5.85048179879206	6.83508654285774	3.48819193384614
H	-6.90877762049865	5.68017248853670	2.67165080914974

H	-5.85048179879206	6.83508654285774	3.48819193384614
H	-6.90877762049865	5.68017248853670	2.67165080914974
H	-3.37784093185691	6.89786127700380	2.91999481050620
H	-8.64429997231222	2.65928678288011	0.59709798106675
H	-8.58174539976510	5.71704954523422	0.74740793824233
H	-6.90742153600165	2.37044188061845	0.47407677220361
H	-3.96658763171982	7.66239818188825	1.43433541456705
H	-3.63970871276099	3.98871994604618	2.12359193589391
H	-9.65253634602015	4.79361928578052	-0.32315392299526
H	-2.85353190524349	6.29235280864393	1.34102078454645
H	-8.60677817780306	6.06049245176741	-0.98094137982110
H	-8.79201155830632	3.17253919746464	-1.96661244532655
H	-7.06335982980832	2.82445854796839	-2.08278550252367
H	-2.25551149403073	5.15803055669386	-1.36267705437992
H	-3.78668112673072	4.37866008482071	-3.26699139145504
H	-7.68345500328907	4.38134943788809	-2.63524979032696
H	-2.16458906973890	4.18192761430312	0.11029996314704
H	-4.97173393787210	3.08629945836443	-2.98698359421346
H	-1.64499127669397	3.50162762172414	-1.43057961148561
H	-3.82562041879871	2.32997553180697	0.53277820621859
H	-4.98545030765702	1.87540111869373	-0.72936126142988
H	-3.25691139467547	2.70222028913902	-3.10869445737936
H	-3.26535135095274	1.55329644750053	-0.94440602489572

S3.1.3 Predicted vibrational frequencies

Table S8. Raman frequencies of 1.

Mode	Freq (cm^{-1})	Activity	Depolarization
1	28.37	0.22288	0.581719
2	59.56	0.188822	0.648687
3	76.84	0.067563	0.554192
4	91.46	0.351685	0.655320
5	109.10	0.30399	0.735702
6	120.46	0.344163	0.612269
7	153.76	0.222419	0.600513
8	161.54	0.11145	0.668351
9	179.33	0.159459	0.707460

10	186.32	0.1193	0.507184
11	192.73	0.208252	0.709317
12	211.41	0.51537	0.596744
13	237.09	0.700132	0.470771
14	263.94	1.565329	0.735554
15	266.12	2.230207	0.116573
16	267.92	1.997293	0.217101
17	279.56	3.701941	0.748680
18	286.03	0.83121	0.439724
19	292.34	3.226941	0.734493
20	306.20	0.615879	0.548163
21	315.39	1.128857	0.173629
22	326.76	0.214035	0.715760
23	332.40	0.271901	0.733620
24	344.47	0.609959	0.657163
25	363.49	0.196507	0.702204
26	374.26	0.278853	0.496420
27	387.20	0.585411	0.367759
28	402.97	0.246073	0.518309
29	414.79	0.99087	0.396171
30	427.03	1.768258	0.559623
31	474.29	2.626921	0.721955
32	481.65	2.455356	0.687290
33	565.10	30.384983	0.000214
34	620.06	3.79542	0.749688
35	621.39	3.419692	0.696030
36	638.36	0.041345	0.748123
37	688.91	5.009656	0.659771
38	692.93	4.86818	0.730817
39	710.94	13.713998	0.260001
40	818.84	10.26232	0.111494
41	822.57	11.028061	0.124912
42	826.03	6.500701	0.286820
43	896.02	4.311497	0.742384
44	900.40	3.727725	0.749908
45	905.35	4.091922	0.749853
46	910.70	4.683301	0.749998
47	914.65	4.105048	0.749737
48	920.96	3.315189	0.749596
49	924.33	0.457658	0.691542
50	933.79	0.656317	0.746852
51	936.23	0.116488	0.417400
52	995.47	1.887517	0.693602
53	1003.51	1.817885	0.749981

54	1005.14	0.982188	0.673661
55	1011.50	1.622728	0.696398
56	1017.30	1.065663	0.647703
57	1024.27	1.057895	0.736214
58	1137.44	5.087466	0.749422
59	1142.28	4.390585	0.748597
60	1190.29	2.922877	0.616060
61	1193.71	3.187021	0.744883
62	1194.77	2.208892	0.710899
63	1197.33	7.913043	0.729236
64	1203.70	4.002668	0.742063
65	1205.34	8.368664	0.744785
66	1206.17	9.788147	0.749465
67	1308.80	7.446811	0.741489
68	1315.04	7.2414	0.749549
69	1336.92	0.151368	0.722220
70	1338.38	0.064317	0.536277
71	1340.13	0.211059	0.709182
72	1345.25	0.125035	0.433200
73	1348.29	0.182059	0.306618
74	1352.15	0.049449	0.678669
75	1367.80	0.052115	0.741584
76	1371.95	0.048891	0.747193
77	1378.45	0.364406	0.356531
78	1410.94	3.25661	0.748137
79	1411.76	1.781033	0.749921
80	1415.34	1.245295	0.708724
81	1417.75	3.041585	0.736853
82	1422.65	3.619563	0.743794
83	1424.96	7.194428	0.748864
84	1426.43	5.827381	0.749573
85	1427.18	4.952087	0.749952
86	1431.31	1.026641	0.735066
87	1435.07	5.59304	0.746258
88	1437.26	7.157858	0.739170
89	1440.65	10.439778	0.748303
90	1441.42	3.450156	0.700926
91	1444.72	11.372054	0.747778
92	1452.53	4.112796	0.738348
93	1453.02	3.866146	0.749830
94	1458.40	1.681869	0.712534
95	1464.86	1.654983	0.727856
96	1568.27	41.213619	0.011179
97	2911.19	2.155568	0.741706

98	2912.78	30.619342	0.148925
99	2913.83	12.418228	0.164248
100	2915.57	26.691788	0.034234
101	2916.11	11.016707	0.465165
102	2917.79	258.906663	0.060294
103	2918.23	3.584185	0.733238
104	2921.22	195.809865	0.077226
105	2923.99	756.132645	0.010687
106	2982.24	42.490084	0.748288
107	2983.95	39.510731	0.620783
108	2984.35	58.037737	0.744944
109	2986.74	21.036858	0.715004
110	2987.96	121.464584	0.735210
111	2989.94	127.195196	0.717676
112	2991.39	4.693873	0.744249
113	2991.99	12.850003	0.730867
114	2993.23	20.84245	0.731829
115	2994.40	36.659543	0.749295
116	2994.87	52.018823	0.669252
117	2996.27	137.086313	0.671617
118	2997.20	14.17892	0.657097
119	2998.41	32.532474	0.748843
120	2999.85	49.789028	0.749975
121	3000.97	16.355535	0.726570
122	3002.15	52.791885	0.695914
123	28.37	50.905293	0.744453

S3.2 Computed heats of formation for tetrahedrane, white phosphorus, and phosphatetrahedrane

Heats of formation were computed using GAMESS (37) (VERSION = 14 FEB 2018). As an example, here is the input file used for the phosphatetrahedrane calculation:

```
$CONTRL SCFTYP=RHF MULT=1 RUNTYP=G3MP2 LOCAL=NONE UNITS=ANGS
$END
$SYSTEM MEMORY=128000000 memddi=2500 $END
$GUESS GUESS=HUCKEL $END
```

```
$CONTRL COORD=UNIQUE $END
$DATA
phosphatetrahedrane structure optimized b3lyp/6-31G**
C1
P 15.0 -0.0000000000  0.0000000000  1.7427417372
C  6.0 -0.4224738461  0.7317461663  0.0813891179
C  6.0 -0.4224738461 -0.7317461663  0.0813891179
C  6.0  0.8449476921  0.0000000000  0.0813891179
H  1.0 -0.9042542357  1.5662142791 -0.3956363636
H  1.0 -0.9042542357 -1.5662142791 -0.3956363636
H  1.0  1.8085084713  0.0000000000 -0.3956363636
$END
```

In each case the initial structure was one previously optimized at the B3LYP/6-31G** level of theory. The G3MP2 procedure reoptimizes the structure at different levels of theory.

S3.2.1 Initial coordinates of tetrahedrane

Table S9. Initial coordinates of tetrahedrane.

Atom	electrons	x	y	z
C	6.0	0.5231656349	-0.5231656349	-0.5231656349
C	6.0	-0.5231656349	0.5231656349	-0.5231656349
C	6.0	-0.5231656349	-0.5231656349	0.5231656349
C	6.0	0.5231656349	0.5231656349	0.5231656349
H	1.0	1.1420938874	-1.1420938874	-1.1420938874
H	1.0	-1.1420938874	1.1420938874	-1.1420938874
H	1.0	-1.1420938874	-1.1420938874	1.1420938874
H	1.0	1.1420938874	1.1420938874	1.1420938874

S3.2.2 Initial coordinates of white phosphorus

Table S10. Initial coordinates of white phosphorus.

Atom	electrons	x	y	z
P	15.0	0.7842817029	-0.7842817029	-0.7842817029
P	15.0	-0.7842817029	0.7842817029	-0.7842817029
P	15.0	-0.7842817029	-0.7842817029	0.7842817029
P	15.0	0.7842817029	0.7842817029	0.7842817029

S3.2.3 Summary of G3MP2 results for phosphatetrahedrane

```
MP2/6-31G(D) = -456.419009 CCSD(T)/6-31G(D) = -456.467859

MP2/G3MP2LARGE = -456.618117 BASIS CONTRIBUT = -0.199108

ZPE(HF/6-31G(D))= 0.044692 ZPE SCALE FACTOR = 0.892900

HLC = -0.091700 FREE ENERGY = 0.024141

THERMAL ENERGY = 0.053589 THERMAL ENTHALPY = 0.054533
----------------------------------------------------------------
E(G3(MP2)) @ 0K = -456.713975 E(G3(MP2)) @298K = -456.710439

H(G3(MP2)) = -456.709495 G(G3(MP2)) = -456.739887
----------------------------------------------------------------
----------------------------------------------------------------
HEAT OF FORMATION (0K): 103.07 KCAL/MOL

HEAT OF FORMATION (298K): 101.06 KCAL/MOL
----------------------------------------------------------------
HEATS OF FORMATIONS BASED ON NIST DATABASE FROM

COMPUTATIONAL CHEMISTRY COMPARISON AND BENCHMARK DATABASE
----------------------------------------------------------------
```

S3.2.4 Summary of G3MP2 results for tetrahedrane

```
MP2/6-31G(D) = -154.107705 CCSD(T)/6-31G(D) = -154.156222
MP2/G3MP2LARGE = -154.284793 BASIS CONTRIBUT = -0.177087
ZPE(HF/6-31G(D))= 0.057883 ZPE SCALE FACTOR = 0.892900
HLC = -0.091700 FREE ENERGY = 0.039915
THERMAL ENERGY = 0.068308 THERMAL ENTHALPY = 0.069252
----------------------------------------------------------------
E(G3(MP2)) @ 0K = -154.367125 E(G3(MP2)) @298K = -154.363644
H(G3(MP2)) = -154.362700 G(G3(MP2)) = -154.392037
----------------------------------------------------------------
----------------------------------------------------------------
HEAT OF FORMATION (0K): 130.86 KCAL/MOL
HEAT OF FORMATION (298K): 128.92 KCAL/MOL
----------------------------------------------------------------
HEATS OF FORMATIONS BASED ON NIST DATABASE FROM
COMPUTATIONAL CHEMISTRY COMPARISON AND BENCHMARK DATABASE
----------------------------------------------------------------
```

S3.2.5 Summary of G3MP2 results for white phosphorus

```
MP2/6-31G(D) = -1363.334900 CCSD(T)/6-31G(D) = -1363.389028
MP2/G3MP2LARGE = -1363.618440 BASIS CONTRIBUT = -0.283540
ZPE(HF/6-31G(D))= 0.006242 ZPE SCALE FACTOR = 0.892900
HLC = -0.091700 FREE ENERGY = -0.021537
THERMAL ENERGY = 0.011171 THERMAL ENTHALPY = 0.012115
----------------------------------------------------------------
E(G3(MP2)) @ 0K = -1363.758026 E(G3(MP2)) @298K = -1363.753846
H(G3(MP2)) = -1363.752902 G(G3(MP2)) = -1363.786554
----------------------------------------------------------------
----------------------------------------------------------------
HEAT OF FORMATION (0K): 17.47 KCAL/MOL
HEAT OF FORMATION (298K): 15.56 KCAL/MOL
----------------------------------------------------------------
HEATS OF FORMATIONS BASED ON NIST DATABASE FROM
COMPUTATIONAL CHEMISTRY COMPARISON AND BENCHMARK DATABASE
----------------------------------------------------------------
```

S3.3 Computed heats of formation for the tetrahedrane and cyclobutadiene isomers of $P(C^tBu)_3$

Heats of formation were computed using GAMESS (37) (VERSION = 30 JUN 2019). The structures for the tetrahedrane and cyclobutadiene isomers of $P(C^tBu)_3$ were initially optimized at the B3LYP-D3/6-311G** level of theory to provide starting geometries for evaluating heat of formation by the G3(MP2,CCSD(T)) methodology as implemented in GAMESS.

S3.3.1 Input file used for the phosphatetrahedrane form of $P(C^tBu)_3$

```
 $CONTRL SCFTYP=RHF MULT=1 RUNTYP=G3MP2  LOCAL=NONE
  UNITS=ANGS $END
 $SYSTEM MEMORY=128000000 memddi=2500 $END
 $GUESS  GUESS=HUCKEL $END
 $CONTRL COORD=UNIQUE $END
 $DATA
phosphatetrahedrane structure optimized b3lyp-d3/6-31G**
C1
 C           6.0  -0.4492449595   0.7193649442   0.2074601845
 C           6.0   0.8473118248   0.0279780559   0.2103291003
 C           6.0  -0.3994448578  -0.7486066091   0.2105848702
 P          15.0  -0.0032541875   0.0029035683   1.8917061811
 C           6.0  -1.1836338923   1.9035621111  -0.3610267380
 C           6.0  -1.0590376505  -1.9776209764  -0.3541786338
 C           6.0   2.2418353149   0.0725875001  -0.3530772036
 C           6.0  -1.3224108318  -1.7874326302  -1.8638142890
 C           6.0  -0.1356000808  -3.1921731585  -0.1380186356
 C           6.0  -2.4002624117  -2.2127359786   0.3695066493
 C           6.0  -0.8925878346   2.0323763246  -1.8720274070
 C           6.0  -2.6964884635   1.7138587667  -0.1375380286
 C           6.0  -0.7146803733   3.1858088977   0.3563858671
 C           6.0   2.2100735449  -0.2391028796  -1.8654850262
 C           6.0   2.8354875808   1.4760537097  -0.1247472487
 C           6.0   3.1162137586  -0.9775042046   0.3617637951
 H           1.0   4.1310287140  -0.9748878004  -0.0520632508
 H           1.0   3.1778060533  -0.7631300365   1.4329529452
 H           1.0   2.7021789196  -1.9837472945   0.2427209018
 H           1.0   3.8762003735   1.5115955360  -0.4658014029
 H           1.0   2.2734534038   2.2362065498  -0.6765513488
 H           1.0   2.8141446028   1.7390012766   0.9376708853
 H           1.0   3.2157199339  -0.1481790052  -2.2910550404
 H           1.0   1.8597204248  -1.2598576059  -2.0466799873
 H           1.0   1.5502045244   0.4485857946  -2.4019785058
 H           1.0  -0.6288479382  -4.1132630545  -0.4679950009
 H           1.0   0.7946490182  -3.0844045349  -0.7048148399
 H           1.0   0.1208417293  -3.3012301123   0.9207881546
 H           1.0  -2.9229279536  -3.0748163953  -0.0606278737
 H           1.0  -2.2357193912  -2.4032243209   1.4340952637
 H           1.0  -3.0526900490  -1.3385625160   0.2797726525
 H           1.0  -1.7339237906  -2.7072522306  -2.2948165841
 H           1.0  -2.0445741425  -0.9837940191  -2.0360311568
 H           1.0  -0.4024688595  -1.5427576864  -2.4027431760
 H           1.0  -1.4732695697   2.8574280182  -2.3002837264
 H           1.0   0.1669084970   2.2403054560  -2.0505014829
 H           1.0  -1.1554811737   1.1176511188  -2.4111953010
 H           1.0  -1.2063666942   4.0662860379  -0.0728100366
 H           1.0  -0.9531067097   3.1400267249   1.4230808986
 H           1.0   0.3673074825   3.3183337057   0.2574589307
```

```
H          1.0  -3.2490986031   2.5970699034  -0.4772228266
H          1.0  -3.0709905986   0.8463490137  -0.6906135777
H          1.0  -2.9149786841   1.5609520349   0.9242750496
$END
```

S3.3.2 G3(MP2,CCSD(T)) output for the phosphatetrahedrane form of $P(C^tBu)_3$

```
----------------------------------------------------------------
    DETAILS OF G3(MP2) CALCULATIONS
----------------------------------------------------------------
E(G3(MP2))@0K   = E(CCSD(T))   + DE(BASIS)   + ZPE(SCALED) + HLC
E(G3(MP2))@298K = E(G3(MP2))@0K + (E(THERMAL) - ZPE)
H(G3(MP2))      = E(G3(MP2))@298K + KT
G(G3(MP2))      = H(G3(MP2)) - TDS

    E(CCSD(T))      = CCSD(T)/6-31G(D)//MP2/6-31G(D) FROZEN CORE
    DE(BASIS)       = E(MP2/G3MP2LARGE) - E(MP2/6-31G(D)) FROZEN CORE
    GEOMETRY MP2/6-31G(D) CORE CORRELATION INCLUDED
    ZPE AND OTHER THERMAL CALCULATIONS AT HF/6-31G(D)
    HLCS USED ARE:  A = 0.009170     B = 0.004455 HARTREE

----------------------------------------------------------------
               SUMMARY OF G3(MP2) CALCULATIONS
----------------------------------------------------------------
MP2/6-31G(D)     =  -926.469932   CCSD(T)/6-31G(D) =  -926.697402
MP2/G3MP2LARGE   =  -927.293465   BASIS CONTRIBUT  =    -0.823533
ZPE(HF/6-31G(D))=      0.368595   ZPE SCALE FACTOR =     0.892900
HLC              =    -0.421820   FREE ENERGY      =     0.368162
THERMAL ENERGY   =     0.431968   THERMAL ENTHALPY =     0.432912
----------------------------------------------------------------
E(G3(MP2)) @ 0K =  -927.574160   E(G3(MP2)) @298K =  -927.554999
H(G3(MP2))      =  -927.554055   G(G3(MP2))       =  -927.618805
----------------------------------------------------------------

----------------------------------------------------------------
      HEAT OF FORMATION   (0K):      24.88 KCAL/MOL
      HEAT OF FORMATION (298K):       6.42 KCAL/MOL
----------------------------------------------------------------
      HEATS OF FORMATIONS BASED ON NIST DATABASE FROM
      COMPUTATIONAL CHEMISTRY COMPARISON AND BENCHMARK DATABASE
----------------------------------------------------------------
```

S3.3.3 Input file used for the phosphacyclobutadiene form of $P(C^tBu)_3$

```
$CONTRL SCFTYP=RHF MULT=1 RUNTYP=G3MP2  LOCAL=NONE
 UNITS=ANGS $END
$SYSTEM MEMORY=128000000 memddi=2500 $END
$GUESS  GUESS=HUCKEL $END
```

```
 $CONTRL COORD=UNIQUE $END
 $DATA
phosphacyclobutadiene structure optimized b3lyp-d3/6-31G**
C1
 P          15.0   0.1464779113   0.0248247502  -0.1951744257
 C           6.0   0.0420733412   0.1124618907   1.7674414181
 C           6.0   1.7432413888  -0.0606812639   0.3760173168
 C           6.0   1.3874973430   0.0354866776   1.9069750479
 C           6.0  -1.3108032720   0.2018094897   2.4451503667
 C           6.0   2.3442652200  -0.0553140284   3.1008968606
 C           6.0   2.9892824846  -0.1292372706  -0.4980493840
 C           6.0   2.5665300960  -0.4973631189  -1.9414781505
 C           6.0   3.6620885671   1.2687199822  -0.5695366696
 C           6.0   4.0094261862  -1.2010608642  -0.0493383793
 C           6.0   3.5955493527   0.8267377391   2.8879271897
 C           6.0   1.7184525416   0.4219304898   4.4237090541
 C           6.0   2.7645257278  -1.5343859733   3.2874528514
 C           6.0  -1.3883823679   0.1136331668   3.9793942486
 C           6.0  -1.9463182437   1.5497272030   2.0123850924
 C           6.0  -2.1731027849  -0.9617201188   1.8826526079
 H           1.0  -2.9731443710   1.6173898387   2.3896735921
 H           1.0  -1.3748399064   2.3925672283   2.4141394210
 H           1.0  -1.9828650436   1.6523942126   0.9229714977
 H           1.0  -3.1877133016  -0.9046314385   2.2919970249
 H           1.0  -2.2523181784  -0.9280284970   0.7918762917
 H           1.0  -1.7457700532  -1.9310402692   2.1607220006
 H           1.0  -2.4412418142   0.1365054799   4.2828639150
 H           1.0  -0.9622945330  -0.8221954551   4.3516246276
 H           1.0  -0.8909364933   0.9507805865   4.4702780779
 H           1.0   2.4926395579   0.4617122949   5.1977121955
 H           1.0   1.2909312279   1.4242084282   4.3246115399
 H           1.0   0.9465945800  -0.2573899591   4.7770904792
 H           1.0   3.5016966188  -1.6254251903   4.0946748074
 H           1.0   1.8933330473  -2.1435920749   3.5494411408
 H           1.0   3.2038743893  -1.9566333360   2.3822944764
 H           1.0   4.2335539519   0.7757160529   3.7766293040
 H           1.0   4.1974348293   0.5078246006   2.0411591719
 H           1.0   3.3135087910   1.8739674244   2.7347242908
 H           1.0   4.5519156499   1.2171255975  -1.2080032568
 H           1.0   2.9684718728   1.9947396880  -1.0040245361
 H           1.0   3.9694313943   1.6423598531   0.4064821391
 H           1.0   4.8174238258  -1.2610670986  -0.7868476696
 H           1.0   4.4673494733  -0.9846214319   0.9156342326
 H           1.0   3.5348899063  -2.1860050764   0.0093106949
 H           1.0   3.4432649170  -0.4845198045  -2.5973783441
 H           1.0   2.1205646890  -1.4958466390  -1.9838380004
 H           1.0   1.8428594807   0.2181422342  -2.3462651593
 $END
```

S3.3.4 G3(MP2,CCSD(T)) output for the phosphacyclobutadiene form of $P(C^tBu)_3$

```
------------------------------------------------------------------
    DETAILS OF G3(MP2) CALCULATIONS
------------------------------------------------------------------
E(G3(MP2))@0K   = E(CCSD(T))   + DE(BASIS)   + ZPE(SCALED) + HLC
E(G3(MP2))@298K = E(G3(MP2))@0K + (E(THERMAL) - ZPE)
H(G3(MP2))      = E(G3(MP2))@298K + KT
G(G3(MP2))      = H(G3(MP2)) - TDS

    E(CCSD(T))      = CCSD(T)/6-31G(D)//MP2/6-31G(D) FROZEN CORE
    DE(BASIS)       = E(MP2/G3MP2LARGE) - E(MP2/6-31G(D)) FROZEN CORE
    GEOMETRY MP2/6-31G(D) CORE CORRELATION INCLUDED
    ZPE AND OTHER THERMAL CALCULATIONS AT HF/6-31G(D)
    HLCS USED ARE:  A = 0.009170     B = 0.004455 HARTREE

------------------------------------------------------------------
               SUMMARY OF G3(MP2) CALCULATIONS
------------------------------------------------------------------
MP2/6-31G(D)    =  -926.443676   CCSD(T)/6-31G(D) =  -926.685069
MP2/G3MP2LARGE  =  -927.264884   BASIS CONTRIBUT  =    -0.821207
ZPE(HF/6-31G(D))=     0.370324   ZPE SCALE FACTOR =     0.892900
HLC             =    -0.421820   FREE ENERGY      =     0.369574
THERMAL ENERGY  =     0.433559   THERMAL ENTHALPY =     0.434504
------------------------------------------------------------------
E(G3(MP2)) @ 0K =  -927.557772   E(G3(MP2)) @298K =  -927.538956
H(G3(MP2))      =  -927.538012   G(G3(MP2))       =  -927.602941
------------------------------------------------------------------

------------------------------------------------------------------
      HEAT OF FORMATION   (0K):      35.16 KCAL/MOL
      HEAT OF FORMATION (298K):      16.49 KCAL/MOL
------------------------------------------------------------------
      HEATS OF FORMATIONS BASED ON NIST DATABASE FROM
      COMPUTATIONAL CHEMISTRY COMPARISON AND BENCHMARK DATABASE
------------------------------------------------------------------
```

S3.4 IQA atomic energy calculations

Molecular structures were optimized at the M062X/6-31G** level of theory using GAMESS to generate a wfn (wavefunction) file. The wfn files were modified by the addition of MODEL M062X to the end of the second line in order to instruct AIMAll to use the M062X model for the IQA calculations as described by Joubert et al. (30). These energies are what is termed the "model IQA approach" has been coded into AIMAll for a small number of density functionals (LSDA, B3LYP, M062X, PBE and PBE0). The results below are taken from the .sum file generated by the AIMALL run. AIMSum (Version 19.02.13, Professional), a component of the AIMAll package (45), was used.

The E_IQA(A) atomic energies for P_4 (S3.4.1), tetrahedrane (S3.4.2), and phosphatetrahedrane (S3.4.3) were converted into kcal/mol and lead to the following main conclusions:

• The C_3H_3 group in phosphatetrahedrane is more stable by 65.7 kcal/mol than in tetrahedrane.

• The P atom in phosphatetrahedrane is less stable than in P_4 by 67.3 kcal/mol.

• The above cancellation of effects is consistent with the near equivalence of the phosphatetrahedrane heat of formation with the sum of 0.25 times the P_4 heat of formation and 0.75 times the tetrahedrane heat of formation. Stated alternatively, the energetic stabilization of C_3H_3 in phosphatetrahedrane versus tetrahedrane comes at the expense of energetic destabilizaiton of P relative to P_4.

S3.4.1 Results for P_4

Nuclear Charges and Cartesian Coordinates:

Atom	Charge	X	Y	Z
P1	15.0	1.4582584900E+00	-1.4582584900E+00	-1.4582584900E+00
P2	15.0	-1.4582584900E+00	1.4582584900E+00	-1.4582584900E+00
P3	15.0	-1.4582584900E+00	-1.4582584900E+00	1.4582584900E+00
P4	15.0	1.4582584900E+00	1.4582584900E+00	1.4582584900E+00

IQA Atomic Energy Components:

```
  E_IQA(A) = Contribution of Atom A to Total Energy E of Molecule, Using IQA Additive Atomic Energy Definition
           = T(A) + Vne(A,Mol)/2 + Ven(A,Mol)/2 + Vee(A) + Vnn(A,Mol)/2
  T(A) = Electronic Kinetic Energy of Atom A (Hamiltonian Form)
  Vne(A,Mol)/2 = Half of Attraction Energy Between Nucleus of Atom A and Electron Density Distribution of Molecule
  Ven(A,Mol)/2 = Half of Attraction Energy Between Electron Density Distribution of Atom A and Nuclei of Molecule
  Vee(A) = Vee(A,A) + Vee(A,A')/2
         = Two-Electron Interaction Energy of Atom A with Itself Plus Half of Two-Electron Interaction Energy
           Between Atom A and Other Atoms of Molecule
  Vnn(A,Mol)/2 = Vnn(A,A')/2 = Half of Repulsion Energy Between Nucleus of Atom A and Other Nuclei of Molecule
  VeeC(A) = Coulomb Part of Vee(A)
  VeeX(A) = Exchange-Correlation Part of Vee(A)
```

See "2EDM Note" and "DFT Note".

Atom A	E_IQA(A)	T(A)	Vne(A,Mol)/2	Ven(A,Mol)/2	Vee(A)	Vnn(A,Mol)/2	VeeC(A)	VeeX(A)
P1	-3.4132653027E+02	3.4040474313E+02	-4.8773788952E+02	-4.8773769106E+02	2.1191775165E+02	8.1826555541E+01	2.3516211267E+02	-2.3244361026E+01
P2	-3.4132653410E+02	3.4040474747E+02	-4.8773788952E+02	-4.8773782573E+02	2.1191787814E+02	8.1826555541E+01	2.3516224388E+02	-2.3244365746E+01
P3	-3.4132653550E+02	3.4040474642E+02	-4.8773788952E+02	-4.8773783243E+02	2.1191788450E+02	8.1826555541E+01	2.3516225060E+02	-2.3244366102E+01
P4	-3.4132652742E+02	3.4040474488E+02	-4.8773788952E+02	-4.8773765416E+02	2.1191771584E+02	8.1826555541E+01	2.3516207553E+02	-2.3244359691E+01
Total	-1.3653061273E+03	1.3616189819E+03	-1.9509515581E+03	-1.9509510034E+03	8.4767123013E+02	3.2730622216E+02	9.4064868268E+02	-9.2977452565E+01

S3.4.2 Results for tetrahedrane

Nuclear Charges and Cartesian Coordinates:

Atom	Charge	X	Y	Z
C1	6.0	9.8233458000E-01	-9.8233458000E-01	-9.8233458000E-01
C2	6.0	-9.8233458000E-01	9.8233458000E-01	-9.8233458000E-01
C3	6.0	-9.8233458000E-01	-9.8233458000E-01	9.8233458000E-01
C4	6.0	9.8233458000E-01	9.8233458000E-01	9.8233458000E-01
H5	1.0	2.1501130400E+00	-2.1501130400E+00	-2.1501130400E+00
H6	1.0	-2.1501130400E+00	2.1501130400E+00	-2.1501130400E+00
H7	1.0	-2.1501130400E+00	-2.1501130400E+00	2.1501130400E+00
H8	1.0	2.1501130400E+00	2.1501130400E+00	2.1501130400E+00

IQA Atomic Energy Components:

```
  E_IQA(A) = Contribution of Atom A to Total Energy E of Molecule, Using IQA Additive Atomic Energy Definition
           = T(A) + Vne(A,Mol)/2 + Ven(A,Mol)/2 + Vee(A) + Vnn(A,Mol)/2
  T(A) = Electronic Kinetic Energy of Atom A (Hamiltonian Form)
  Vne(A,Mol)/2 = Half of Attraction Energy Between Nucleus of Atom A and Electron Density Distribution of Molecule
  Ven(A,Mol)/2 = Half of Attraction Energy Between Electron Density Distribution of Atom A and Nuclei of Molecule
  Vee(A) = Vee(A,A) + Vee(A,A')/2
         = Two-Electron Interaction Energy of Atom A with Itself Plus Half of Two-Electron Interaction Energy
           Between Atom A and Other Atoms of Molecule
  Vnn(A,Mol)/2 = Vnn(A,A')/2 = Half of Repulsion Energy Between Nucleus of Atom A and Other Nuclei of Molecule
  VeeC(A) = Coulomb Part of Vee(A)
  VeeX(A) = Exchange-Correlation Part of Vee(A)
```

See "2EDM Note" and "DFT Note".

Atom A	E_IQA(A)	T(A)	Vne(A,Mol)/2	Ven(A,Mol)/2	Vee(A)	Vnn(A,Mol)/2	VeeC(A)	VeeX(A)
C1	-3.8078082538E+01	3.7733636501E+01	-6.7044126379E+01	-6.7438947438E+01	3.5788424155E+01	2.2882930623E+01	4.1228839211E+01	-5.4404150557E+00
C2	-3.8078081860E+01	3.7733649935E+01	-6.7044126379E+01	-6.7439033230E+01	3.5788497191E+01	2.2882930623E+01	4.1228919028E+01	-5.4404218367E+00
C3	-3.8078087264E+01	3.7733643582E+01	-6.7044126379E+01	-6.7439031537E+01	3.5788496447E+01	2.2882930623E+01	4.1228918232E+01	-5.4404217855E+00
C4	-3.8078076628E+01	3.7733642635E+01	-6.7044126379E+01	-6.7438926573E+01	3.5788403066E+01	2.2882930623E+01	4.1228817361E+01	-5.4404142946E+00
H5	-5.6982757658E-01	5.8538176486E-01	-4.2328357635E+00	-3.8378891994E+00	3.2211471265E+00	3.6943684950E+00	3.5625668752E+00	-3.4141974870E-01
H6	-5.6982845459E-01	5.8538281617E-01	-4.2328357635E+00	-3.8379119750E+00	3.2211679727E+00	3.6943684950E+00	3.5625898664E+00	-3.4142189373E-01
H7	-5.6982862979E-01	5.8538254883E-01	-4.2328357635E+00	-3.8379136465E+00	3.2211697364E+00	3.6943684950E+00	3.5625915093E+00	-3.4142177286E-01
H8	-5.6982658117E-01	5.8538210091E-01	-4.2328357635E+00	-3.8378865413E+00	3.2211451277E+00	3.6943684950E+00	3.5625643529E+00	-3.4141922520E-01
Total	-1.5459163953E+02	1.5327610188E+02	-2.8510784857E+02	-2.8510754014E+02	1.5603845082E+02	1.0630919647E+02	1.7916580644E+02	-2.3127355613E+01

S3.4.3 Results for phosphatetrahedrane

Nuclear Charges and Cartesian Coordinates:

Atom	Charge	X	Y	Z
P1	15.0	0.0000000000E+00	0.0000000000E+00	3.2507346700E+00
C2	6.0	-7.9647166000E-01	1.3795293900E+00	1.6380437000E-01
C3	6.0	-7.9647166000E-01	-1.3795293900E+00	1.6380437000E-01
C4	6.0	1.5929433300E+00	0.0000000000E+00	1.6380437000E-01
H5	1.0	-1.7035236200E+00	2.9505894700E+00	-7.4345566000E-01
H6	1.0	-1.7035236200E+00	-2.9505894700E+00	-7.4345566000E-01
H7	1.0	3.4070472500E+00	0.0000000000E+00	-7.4345566000E-01

IQA Atomic Energy Components:

```
  E_IQA(A) = Contribution of Atom A to Total Energy E of Molecule, Using IQA Additive Atomic Energy Definition
           = T(A) + Vne(A,Mol)/2 + Ven(A,Mol)/2 + Vee(A) + Vnn(A,Mol)/2
  T(A) = Electronic Kinetic Energy of Atom A (Hamiltonian Form)
  Vne(A,Mol)/2 = Half of Attraction Energy Between Nucleus of Atom A and Electron Density Distribution of Molecule
  Ven(A,Mol)/2 = Half of Attraction Energy Between Electron Density Distribution of Atom A and Nuclei of Molecule
  Vee(A) = Vee(A,A) + Vee(A,A')/2
         = Two-Electron Interaction Energy of Atom A with Itself Plus Half of Two-Electron Interaction Energy
           Between Atom A and Other Atoms of Molecule
  Vnn(A,Mol)/2 = Vnn(A,A')/2 = Half of Repulsion Energy Between Nucleus of Atom A and Other Nuclei of Molecule
  VeeC(A) = Coulomb Part of Vee(A)
  VeeX(A) = Exchange-Correlation Part of Vee(A)
See "2EDM Note" and "DFT Note".
```

Atom A	E_IQA(A)	T(A)	Vne(A,Mol)/2	Ven(A,Mol)/2	Vee(A)	Vnn(A,Mol)/2	VeeC(A)	VeeX(A)
P1	-3.4121933885E+02	3.3982112622E+02	-4.4922925463E+02	-4.4164846207E+02	1.6668802687E+02	4.3149224765E+01	1.8956637171E+02	-2.2878344845E+01
C2	-3.8114601580E+01	3.7883584897E+01	-7.2928909369E+01	-7.5925819609E+01	4.4046522558E+01	2.8810019942E+01	4.9602884410E+01	-5.5563618522E+00
C3	-3.8114624329E+01	3.7883552289E+01	-7.2928909369E+01	-7.5925899095E+01	4.4046611904E+01	2.8810019942E+01	4.9602979342E+01	-5.5563674376E+00
C4	-3.8114611998E+01	3.7883559507E+01	-7.2928909339E+01	-7.5925753030E+01	4.4046470918E+01	2.8810019946E+01	4.9602828492E+01	-5.5563575744E+00
H5	-5.6817506937E-01	5.8477268286E-01	-4.9403583996E+00	-4.4702148716E+00	3.8519574602E+00	4.4056680587E+00	4.1921095597E+00	-3.4015209949E-01
H6	-5.6817379737E-01	5.8477263962E-01	-4.9403583996E+00	-4.4702001387E+00	3.8519440426E+00	4.4056680587E+00	4.1920947399E+00	-3.4015069730E-01
H7	-5.6817331355E-01	5.8477338634E-01	-4.9403583992E+00	-4.4701996616E+00	3.8519433019E+00	4.4056680590E+00	4.1920937352E+00	-3.4015043334E-01
Total	-4.5726769894E+02	4.5522614162E+02	-6.8283705791E+02	-6.8283654848E+02	3.1038347705E+02	1.4279628877E+02	3.5095136199E+02	-4.0567884939E+01

S3.5 Generation of the molecular graph for $({}^{t}BuC)_3P$ (1)

The electron density is from a GAMESS run using the following input file:

```
!  File created by MacMolPlt 7.7
$CONTRL SCFTYP=RHF RUNTYP=ENERGY DFTTYP=B3LYP MAXIT=30 MULT=1
AIMPAC=.t.  $END
$SYSTEM MEMORY=128000000 $END
$BASIS GBASIS=N31 NGAUSS=6 NDFUNC=1 NPFUNC=1 $END
$SCF DIRSCF=.TRUE. $END
$DFT dc=.t.  $END
$DATA
C1
tri-tert-butyl tetrahedrane optimized starting in C3 symmetry
 C  6.0 -0.4492449595  0.7193649442  0.2074601845
 C  6.0  0.8473118248  0.0279780559  0.2103291003
 C  6.0 -0.3994448578 -0.7486066091  0.2105848702
 P 15.0 -0.0032541875  0.0029035683  1.8917061811
 C  6.0 -1.1836338923  1.9035621111 -0.3610267380
 C  6.0 -1.0590376505 -1.9776209764 -0.3541786338
 C  6.0  2.2418353149  0.0725875001 -0.3530772036
 C  6.0 -1.3224108318 -1.7874326302 -1.8638142890
 C  6.0 -0.1356000808 -3.1921731585 -0.1380186356
 C  6.0 -2.4002624117 -2.2127359786  0.3695066493
 C  6.0 -0.8925878346  2.0323763246 -1.8720274070
 C  6.0 -2.6964884635  1.7138587667 -0.1375380286
 C  6.0 -0.7146803733  3.1858088977  0.3563858671
```

C	6.0	2.2100735449	-0.2391028796	-1.8654850262
C	6.0	2.8354875808	1.4760537097	-0.1247472487
C	6.0	3.1162137586	-0.9775042046	0.3617637951
H	1.0	4.1310287140	-0.9748878004	-0.0520632508
H	1.0	3.1778060533	-0.7631300365	1.4329529452
H	1.0	2.7021789196	-1.9837472945	0.2427209018
H	1.0	3.8762003735	1.5115955360	-0.4658014029
H	1.0	2.2734534038	2.2362065498	-0.6765513488
H	1.0	2.8141446028	1.7390012766	0.9376708853
H	1.0	3.2157199339	-0.1481790052	-2.2910550404
H	1.0	1.8597204248	-1.2598576059	-2.0466799873
H	1.0	1.5502045244	0.4485857946	-2.4019785058
H	1.0	-0.6288479382	-4.1132630545	-0.4679950009
H	1.0	0.7946490182	-3.0844045349	-0.7048148399
H	1.0	0.1208417293	-3.3012301123	0.9207881546
H	1.0	-2.9229279536	-3.0748163953	-0.0606278737
H	1.0	-2.2357193912	-2.4032243209	1.4340952637
H	1.0	-3.0526900490	-1.3385625160	0.2797726525
H	1.0	-1.7339237906	-2.7072522306	-2.2948165841
H	1.0	-2.0445741425	-0.9837940191	-2.0360311568
H	1.0	-0.4024688595	-1.5427576864	-2.4027431760
H	1.0	-1.4732695697	2.8574280182	-2.3002837264
H	1.0	0.1669084970	2.2403054560	-2.0505014829
H	1.0	-1.1554811737	1.1176511188	-2.4111953010
H	1.0	-1.2063666942	4.0662860379	-0.0728100366

```
H  1.0 -0.9531067097  3.1400267249  1.4230808986
H  1.0  0.3673074825  3.3183337057  0.2574589307
H  1.0 -3.2490986031  2.5970699034 -0.4772228266
H  1.0 -3.0709905986  0.8463490137 -0.6906135777
H  1.0 -2.9149786841  1.5609520349  0.9242750496
$END
```

The above calculation is at the B3LYP-D3/6-31G** level of theory (46); the structure had previously been optimized with no symmetry starting from a model having C3 symmetry. The *.wfn file was opened with Multiwfn (47) and then the following sequence of menu items was chosen:

```
2 Topology analysis
2 Search CPs from nuclear positions
3 Search CPs from midpoint of atom pairs
4 Search CPs from triangle center of three atoms
5 Search CPs from pyramid center of four atoms
8 Generating the paths connecting (3,-3) and (3,-1) CPs
0 Print and visualize all generated CPs, paths and interbasin
surfaces
```

In the GUI that pops up the desired quantities were added to the graphic window and a molecular graph graphic was generated.

S3.6 Natural bond orbital analysis

Examination of the natural bond orbitals (27) indicates that there is no significant change in the lone pair composition from P_4 to phosphatetrahedrane (ca. 80% s in character), and likewise the atomic orbital contributions to the bonds in these molecules changes almost not at all. The

central bonds of the tetrahedral cores of the molecules shown in Fig. **??**B are very high in p-orbital content, moreso for P than for C. The C atom directs an external hybrid orbital that is a rich 40% in s character which, as discussed by Wiberg and Bader (31), is the origin of the high strain energy for tetrahedrane (C is stabilized relative to the carbon in the standard methine group, but H is destabilized by an even greater amount).

NBO analysis was carried out for tetrahedrane $(CH)_4$, phosphatetrahedrane $P(CH)_3$, and white phosphorus P_4, in order to determine the nature (s versus p character) of the cage and terminal hybrid orbitals utilized by carbon, and to determine the nature of the bond and lone pairs at phosphorus. The results reported below were computed using GAMESS/NBO6 at the B3LYP/6-31G** level of theory.

S3.6.1 Tetrahedrane

```
*********************************** NBO 6.0 ***********************************
             N A T U R A L   A T O M I C   O R B I T A L   A N D
          N A T U R A L   B O N D   O R B I T A L   A N A L Y S I S
 **************** Massachusetts Institute of Technology (100648) ***************
  (c) Copyright 1996-2018 Board of Regents of the University of Wisconsin System
      on behalf of the Theoretical Chemistry Institute.  All rights reserved.

          Cite this program [NBO 6.0.18a (11-Mar-2018)] as:

          NBO 6.0.  E. D. Glendening, J. K. Badenhoop, A. E. Reed,
          J. E. Carpenter, J. A. Bohmann, C. M. Morales, C. R. Landis,
          and F. Weinhold (Theoretical Chemistry Institute, University
          of Wisconsin, Madison, WI, 2013); http://nbo6.chem.wisc.edu/

       /MEMORY / : Allocate 150000000 words of dynamic memory
       /NBO    / : Natural Bond Orbital Analysis
       /NRT    / : Natural Resonance Theory Analysis
       /MOLDEN / : Write NBOs to MOLDEN-formatted file

 Job title:  tetrahedrane optimization


 NATURAL POPULATIONS:  Natural atomic orbital occupancies

  NAO Atom No lang   Type(AO)    Occupancy      Energy
 -------------------------------------------------------
   1    C  1  s      Cor( 1s)     1.99999     -10.19551
   2    C  1  s      Val( 2s)     0.95461      -0.13405
   3    C  1  s      Ryd( 3s)     0.00068       1.17975
   4    C  1  s      Ryd( 4s)     0.00005       4.45903
   5    C  1  px     Val( 2p)     1.09708      -0.05232
   6    C  1  px     Ryd( 3p)     0.00306       0.77347
   7    C  1  py     Val( 2p)     1.09708      -0.05232
```

8	C	1	py	Ryd(3p)	0.00306	0.77347
9	C	1	pz	Val(2p)	1.09708	-0.05232
10	C	1	pz	Ryd(3p)	0.00306	0.77347
11	C	1	dxy	Ryd(3d)	0.00131	2.31587
12	C	1	dxz	Ryd(3d)	0.00131	2.31587
13	C	1	dyz	Ryd(3d)	0.00131	2.31587
14	C	1	dx2y2	Ryd(3d)	0.00133	2.02914
15	C	1	dz2	Ryd(3d)	0.00133	2.02914
16	C	2	s	Cor(1s)	1.99999	-10.19551
17	C	2	s	Val(2s)	0.95461	-0.13405
18	C	2	s	Ryd(3s)	0.00068	1.17975
19	C	2	s	Ryd(4s)	0.00005	4.45903
20	C	2	px	Val(2p)	1.09708	-0.05232
21	C	2	px	Ryd(3p)	0.00306	0.77347
22	C	2	py	Val(2p)	1.09708	-0.05232
23	C	2	py	Ryd(3p)	0.00306	0.77347
24	C	2	pz	Val(2p)	1.09708	-0.05232
25	C	2	pz	Ryd(3p)	0.00306	0.77347
26	C	2	dxy	Ryd(3d)	0.00131	2.31587
27	C	2	dxz	Ryd(3d)	0.00131	2.31587
28	C	2	dyz	Ryd(3d)	0.00131	2.31587
29	C	2	dx2y2	Ryd(3d)	0.00133	2.02914
30	C	2	dz2	Ryd(3d)	0.00133	2.02914
31	C	3	s	Cor(1s)	1.99999	-10.19551
32	C	3	s	Val(2s)	0.95461	-0.13405
33	C	3	s	Ryd(3s)	0.00068	1.17975
34	C	3	s	Ryd(4s)	0.00005	4.45903
35	C	3	px	Val(2p)	1.09708	-0.05232
36	C	3	px	Ryd(3p)	0.00306	0.77347
37	C	3	py	Val(2p)	1.09708	-0.05232
38	C	3	py	Ryd(3p)	0.00306	0.77347
39	C	3	pz	Val(2p)	1.09708	-0.05232
40	C	3	pz	Ryd(3p)	0.00306	0.77347
41	C	3	dxy	Ryd(3d)	0.00131	2.31587
42	C	3	dxz	Ryd(3d)	0.00131	2.31587
43	C	3	dyz	Ryd(3d)	0.00131	2.31587
44	C	3	dx2y2	Ryd(3d)	0.00133	2.02914
45	C	3	dz2	Ryd(3d)	0.00133	2.02914
46	C	4	s	Cor(1s)	1.99999	-10.19551
47	C	4	s	Val(2s)	0.95461	-0.13405
48	C	4	s	Ryd(3s)	0.00068	1.17975
49	C	4	s	Ryd(4s)	0.00005	4.45903
50	C	4	px	Val(2p)	1.09708	-0.05232
51	C	4	px	Ryd(3p)	0.00306	0.77347
52	C	4	py	Val(2p)	1.09708	-0.05232
53	C	4	py	Ryd(3p)	0.00306	0.77347
54	C	4	pz	Val(2p)	1.09708	-0.05232
55	C	4	pz	Ryd(3p)	0.00306	0.77347
56	C	4	dxy	Ryd(3d)	0.00131	2.31587
57	C	4	dxz	Ryd(3d)	0.00131	2.31587
58	C	4	dyz	Ryd(3d)	0.00131	2.31587
59	C	4	dx2y2	Ryd(3d)	0.00133	2.02914
60	C	4	dz2	Ryd(3d)	0.00133	2.02914
61	H	5	s	Val(1s)	0.73669	0.12723
62	H	5	s	Ryd(2s)	0.00033	0.53884
63	H	5	px	Ryd(2p)	0.00020	2.64727
64	H	5	py	Ryd(2p)	0.00020	2.64727
65	H	5	pz	Ryd(2p)	0.00020	2.64727

66	H	6	s	Val(1s)	0.73669	0.12723
67	H	6	s	Ryd(2s)	0.00033	0.53884
68	H	6	px	Ryd(2p)	0.00020	2.64727
69	H	6	py	Ryd(2p)	0.00020	2.64727
70	H	6	pz	Ryd(2p)	0.00020	2.64727
71	H	7	s	Val(1s)	0.73669	0.12723
72	H	7	s	Ryd(2s)	0.00033	0.53884
73	H	7	px	Ryd(2p)	0.00020	2.64727
74	H	7	py	Ryd(2p)	0.00020	2.64727
75	H	7	pz	Ryd(2p)	0.00020	2.64727
76	H	8	s	Val(1s)	0.73669	0.12723
77	H	8	s	Ryd(2s)	0.00033	0.53884
78	H	8	px	Ryd(2p)	0.00020	2.64727
79	H	8	py	Ryd(2p)	0.00020	2.64727
80	H	8	pz	Ryd(2p)	0.00020	2.64727

Summary of Natural Population Analysis:

Atom No	Natural Charge	Natural Population Core	Valence	Rydberg	Total
C 1	-0.26237	1.99999	4.24586	0.01651	6.26237
C 2	-0.26237	1.99999	4.24586	0.01651	6.26237
C 3	-0.26237	1.99999	4.24586	0.01651	6.26237
C 4	-0.26237	1.99999	4.24586	0.01651	6.26237
H 5	0.26237	0.00000	0.73669	0.00095	0.73763
H 6	0.26237	0.00000	0.73669	0.00095	0.73763
H 7	0.26237	0.00000	0.73669	0.00095	0.73763
H 8	0.26237	0.00000	0.73669	0.00095	0.73763
* Total *	0.00000	7.99997	19.93019	0.06984	28.00000

	Natural Population
Core	7.99997 (99.9996% of 8)
Valence	19.93019 (99.6510% of 20)
Natural Minimal Basis	27.93016 (99.7506% of 28)
Natural Rydberg Basis	0.06984 (0.2494% of 28)

Atom No	Natural Electron Configuration
C 1	[core]2s(0.95)2p(3.29)3p(0.01)3d(0.01)
C 2	[core]2s(0.95)2p(3.29)3p(0.01)3d(0.01)
C 3	[core]2s(0.95)2p(3.29)3p(0.01)3d(0.01)
C 4	[core]2s(0.95)2p(3.29)3p(0.01)3d(0.01)
H 5	1s(0.74)
H 6	1s(0.74)
H 7	1s(0.74)
H 8	1s(0.74)

NATURAL BOND ORBITAL ANALYSIS:

Cycle	Max Ctr	Occ Thresh	Occupancies Lewis	non-Lewis	Lewis Structure CR	BD	nC	LP	Low occ (L)	High occ (NL)

```
==============================================================================
   1     2     1.90    27.75728   0.24272      4  10   0   0     0      0
------------------------------------------------------------------------------

Structure accepted: No low occupancy Lewis orbitals

-------------------------------------------------------
  Core                      7.99997 (100.000% of   8)
  Valence Lewis            19.75731 ( 98.787% of  20)
 ==================      =============================
  Total Lewis              27.75728 ( 99.133% of  28)
 -----------------------------------------------------
  Valence non-Lewis         0.21031 (  0.751% of  28)
  Rydberg non-Lewis         0.03241 (  0.116% of  28)
 ==================      =============================
  Total non-Lewis           0.24272 (  0.867% of  28)
-------------------------------------------------------


    (Occupancy)   Bond orbital / Coefficients / Hybrids
------------------ Lewis ------------------------------------------------------
  1. (1.99999) CR ( 1) C  1           s(100.00%)
                                        1.0000  0.0000  0.0000  0.0000  0.0000
                                        0.0000  0.0000  0.0000  0.0000  0.0000
                                        0.0000  0.0000  0.0000  0.0000  0.0000
  2. (1.99999) CR ( 1) C  2           s(100.00%)
                                        1.0000  0.0000  0.0000  0.0000  0.0000
                                        0.0000  0.0000  0.0000  0.0000  0.0000
                                        0.0000  0.0000  0.0000  0.0000  0.0000
  3. (1.99999) CR ( 1) C  3           s(100.00%)
                                        1.0000  0.0000  0.0000  0.0000  0.0000
                                        0.0000  0.0000  0.0000  0.0000  0.0000
                                        0.0000  0.0000  0.0000  0.0000  0.0000
  4. (1.99999) CR ( 1) C  4           s(100.00%)
                                        1.0000  0.0000  0.0000  0.0000  0.0000
                                        0.0000  0.0000  0.0000  0.0000  0.0000
                                        0.0000  0.0000  0.0000  0.0000  0.0000
  5. (1.96127) BD ( 1) C  1- C  2
               ( 50.00%)   0.7071* C  1 s( 19.70%)p 4.07( 80.18%)d 0.01(  0.11%)
                                        0.0000  0.4438  0.0076 -0.0025 -0.5447
                                        0.0183  0.5447 -0.0183 -0.4545 -0.0337
                                       -0.0291  0.0003 -0.0003  0.0000 -0.0168
               ( 50.00%)   0.7071* C  2 s( 19.70%)p 4.07( 80.18%)d 0.01(  0.11%)
                                        0.0000  0.4438  0.0076 -0.0025  0.5447
                                       -0.0183 -0.5447  0.0183 -0.4545 -0.0337
                                       -0.0291 -0.0003  0.0003  0.0000 -0.0168
  6. (1.96127) BD ( 1) C  1- C  3
               ( 50.00%)   0.7071* C  1 s( 19.70%)p 4.07( 80.18%)d 0.01(  0.11%)
                                        0.0000  0.4438  0.0076 -0.0025 -0.5447
                                        0.0183 -0.4545 -0.0337  0.5447 -0.0183
                                        0.0003 -0.0291 -0.0003  0.0146  0.0084
               ( 50.00%)   0.7071* C  3 s( 19.70%)p 4.07( 80.18%)d 0.01(  0.11%)
                                        0.0000  0.4438  0.0076 -0.0025  0.5447
                                       -0.0183 -0.4545 -0.0337 -0.5447  0.0183
                                       -0.0003 -0.0291  0.0003  0.0146  0.0084
  7. (1.96127) BD ( 1) C  1- C  4
               ( 50.00%)   0.7071* C  1 s( 19.70%)p 4.07( 80.18%)d 0.01(  0.11%)
                                        0.0000  0.4438  0.0076 -0.0025  0.4545
                                        0.0337  0.5447 -0.0183  0.5447 -0.0183
                                        0.0003  0.0003  0.0291 -0.0146  0.0084
               ( 50.00%)   0.7071* C  4 s( 19.70%)p 4.07( 80.18%)d 0.01(  0.11%)
                                        0.0000  0.4438  0.0076 -0.0025  0.4545
```

```
                                         0.0337 -0.5447  0.0183 -0.5447  0.0183
                                        -0.0003 -0.0003  0.0291 -0.0146  0.0084
   8. (1.99742) BD ( 1) C  1- H  5
              ( 63.99%)   0.7999* C  1 s( 40.52%)p 1.47( 59.44%)d 0.00(  0.04%)
                                         0.0000  0.6365 -0.0080  0.0031  0.4451
                                        -0.0016 -0.4451  0.0016 -0.4451  0.0016
                                        -0.0119 -0.0119  0.0119  0.0000  0.0000
              ( 36.01%)   0.6001* H  5 s( 99.95%)p 0.00(  0.05%)
                                         0.9998  0.0034 -0.0126  0.0126  0.0126
   9. (1.96127) BD ( 1) C  2- C  3
              ( 50.00%)   0.7071* C  2 s( 19.70%)p 4.07( 80.18%)d 0.01(  0.11%)
                                         0.0000  0.4438  0.0076 -0.0025 -0.4545
                                        -0.0337 -0.5447  0.0183  0.5447 -0.0183
                                         0.0003 -0.0003 -0.0291 -0.0146  0.0084
              ( 50.00%)   0.7071* C  3 s( 19.70%)p 4.07( 80.18%)d 0.01(  0.11%)
                                         0.0000  0.4438  0.0076 -0.0025 -0.4545
                                        -0.0337  0.5447 -0.0183 -0.5447  0.0183
                                        -0.0003  0.0003 -0.0291 -0.0146  0.0084
  10. (1.96127) BD ( 1) C  2- C  4
              ( 50.00%)   0.7071* C  2 s( 19.70%)p 4.07( 80.18%)d 0.01(  0.11%)
                                         0.0000  0.4438  0.0076 -0.0025  0.5447
                                        -0.0183  0.4545  0.0337  0.5447 -0.0183
                                         0.0003  0.0291  0.0003  0.0146  0.0084
              ( 50.00%)   0.7071* C  4 s( 19.70%)p 4.07( 80.18%)d 0.01(  0.11%)
                                         0.0000  0.4438  0.0076 -0.0025 -0.5447
                                         0.0183  0.4545  0.0337 -0.5447  0.0183
                                        -0.0003  0.0291 -0.0003  0.0146  0.0084
  11. (1.99742) BD ( 1) C  2- H  6
              ( 63.99%)   0.7999* C  2 s( 40.52%)p 1.47( 59.44%)d 0.00(  0.04%)
                                         0.0000  0.6365 -0.0080  0.0031 -0.4451
                                         0.0016  0.4451 -0.0016 -0.4451  0.0016
                                        -0.0119  0.0119 -0.0119  0.0000  0.0000
              ( 36.01%)   0.6001* H  6 s( 99.95%)p 0.00(  0.05%)
                                         0.9998  0.0034  0.0126 -0.0126  0.0126
  12. (1.96127) BD ( 1) C  3- C  4
              ( 50.00%)   0.7071* C  3 s( 19.70%)p 4.07( 80.18%)d 0.01(  0.11%)
                                         0.0000  0.4438  0.0076 -0.0025  0.5447
                                        -0.0183  0.5447 -0.0183  0.4545  0.0337
                                         0.0291  0.0003  0.0003  0.0000 -0.0168
              ( 50.00%)   0.7071* C  4 s( 19.70%)p 4.07( 80.18%)d 0.01(  0.11%)
                                         0.0000  0.4438  0.0076 -0.0025 -0.5447
                                         0.0183 -0.5447  0.0183  0.4545  0.0337
                                         0.0291 -0.0003 -0.0003  0.0000 -0.0168
  13. (1.99742) BD ( 1) C  3- H  7
              ( 63.99%)   0.7999* C  3 s( 40.52%)p 1.47( 59.44%)d 0.00(  0.04%)
                                         0.0000  0.6365 -0.0080  0.0031 -0.4451
                                         0.0016 -0.4451  0.0016  0.4451 -0.0016
                                         0.0119 -0.0119 -0.0119  0.0000  0.0000
              ( 36.01%)   0.6001* H  7 s( 99.95%)p 0.00(  0.05%)
                                         0.9998  0.0034  0.0126  0.0126 -0.0126
  14. (1.99742) BD ( 1) C  4- H  8
              ( 63.99%)   0.7999* C  4 s( 40.52%)p 1.47( 59.44%)d 0.00(  0.04%)
                                         0.0000  0.6365 -0.0080  0.0031  0.4451
                                        -0.0016  0.4451 -0.0016  0.4451 -0.0016
                                         0.0119  0.0119  0.0119  0.0000  0.0000
              ( 36.01%)   0.6001* H  8 s( 99.95%)p 0.00(  0.05%)
                                         0.9998  0.0034 -0.0126 -0.0126 -0.0126
---------------- non-Lewis ----------------------------------------------------
  15. (0.01653) BD*( 1) C  1- C  2
              ( 50.00%)   0.7071* C  1 s( 19.70%)p 4.07( 80.18%)d 0.01(  0.11%)
                                         0.0000  0.4438  0.0076 -0.0025 -0.5447
                                         0.0183  0.5447 -0.0183 -0.4545 -0.0337
```

```
                                    -0.0291  0.0003 -0.0003  0.0000 -0.0168
           ( 50.00%)  -0.7071* C  2 s( 19.70%)p 4.07( 80.18%)d 0.01(  0.11%)
                                     0.0000  0.4438  0.0076 -0.0025  0.5447
                                    -0.0183 -0.5447  0.0183 -0.4545 -0.0337
                                    -0.0291 -0.0003  0.0003  0.0000 -0.0168
16. (0.01653) BD*( 1) C  1- C  3
           ( 50.00%)   0.7071* C  1 s( 19.70%)p 4.07( 80.18%)d 0.01(  0.11%)
                                     0.0000  0.4438  0.0076 -0.0025 -0.5447
                                     0.0183 -0.4545 -0.0337  0.5447 -0.0183
                                     0.0003 -0.0291 -0.0003  0.0146  0.0084
           ( 50.00%)  -0.7071* C  3 s( 19.70%)p 4.07( 80.18%)d 0.01(  0.11%)
                                     0.0000  0.4438  0.0076 -0.0025  0.5447
                                    -0.0183 -0.4545 -0.0337 -0.5447  0.0183
                                    -0.0003 -0.0291  0.0003  0.0146  0.0084
17. (0.01653) BD*( 1) C  1- C  4
           ( 50.00%)   0.7071* C  1 s( 19.70%)p 4.07( 80.18%)d 0.01(  0.11%)
                                     0.0000  0.4438  0.0076 -0.0025  0.4545
                                     0.0337  0.5447 -0.0183  0.5447 -0.0183
                                     0.0003  0.0003  0.0291 -0.0146  0.0084
           ( 50.00%)  -0.7071* C  4 s( 19.70%)p 4.07( 80.18%)d 0.01(  0.11%)
                                     0.0000  0.4438  0.0076 -0.0025  0.4545
                                     0.0337 -0.5447  0.0183 -0.5447  0.0183
                                    -0.0003 -0.0003  0.0291 -0.0146  0.0084
18. (0.02778) BD*( 1) C  1- H  5
           ( 36.01%)   0.6001* C  1 s( 40.52%)p 1.47( 59.44%)d 0.00(  0.04%)
                                     0.0000 -0.6365  0.0080 -0.0031 -0.4451
                                     0.0016  0.4451 -0.0016  0.4451 -0.0016
                                     0.0119  0.0119 -0.0119  0.0000  0.0000
           ( 63.99%)  -0.7999* H  5 s( 99.95%)p 0.00(  0.05%)
                                    -0.9998 -0.0034  0.0126 -0.0126 -0.0126
19. (0.01653) BD*( 1) C  2- C  3
           ( 50.00%)   0.7071* C  2 s( 19.70%)p 4.07( 80.18%)d 0.01(  0.11%)
                                     0.0000  0.4438  0.0076 -0.0025 -0.4545
                                    -0.0337 -0.5447  0.0183  0.5447 -0.0183
                                     0.0003 -0.0003 -0.0291 -0.0146  0.0084
           ( 50.00%)  -0.7071* C  3 s( 19.70%)p 4.07( 80.18%)d 0.01(  0.11%)
                                     0.0000  0.4438  0.0076 -0.0025 -0.4545
                                    -0.0337  0.5447 -0.0183 -0.5447  0.0183
                                    -0.0003  0.0003 -0.0291 -0.0146  0.0084
20. (0.01653) BD*( 1) C  2- C  4
           ( 50.00%)   0.7071* C  2 s( 19.70%)p 4.07( 80.18%)d 0.01(  0.11%)
                                     0.0000  0.4438  0.0076 -0.0025  0.5447
                                    -0.0183  0.4545  0.0337  0.5447 -0.0183
                                     0.0003  0.0291  0.0003  0.0146  0.0084
           ( 50.00%)  -0.7071* C  4 s( 19.70%)p 4.07( 80.18%)d 0.01(  0.11%)
                                     0.0000  0.4438  0.0076 -0.0025 -0.5447
                                     0.0183  0.4545  0.0337 -0.5447  0.0183
                                    -0.0003  0.0291 -0.0003  0.0146  0.0084
21. (0.02778) BD*( 1) C  2- H  6
           ( 36.01%)   0.6001* C  2 s( 40.52%)p 1.47( 59.44%)d 0.00(  0.04%)
                                     0.0000 -0.6365  0.0080 -0.0031  0.4451
                                    -0.0016 -0.4451  0.0016  0.4451 -0.0016
                                     0.0119 -0.0119  0.0119  0.0000  0.0000
           ( 63.99%)  -0.7999* H  6 s( 99.95%)p 0.00(  0.05%)
                                    -0.9998 -0.0034 -0.0126  0.0126 -0.0126
22. (0.01653) BD*( 1) C  3- C  4
           ( 50.00%)   0.7071* C  3 s( 19.70%)p 4.07( 80.18%)d 0.01(  0.11%)
                                     0.0000  0.4438  0.0076 -0.0025  0.5447
                                    -0.0183  0.5447 -0.0183  0.4545  0.0337
                                     0.0291  0.0003  0.0003  0.0000 -0.0168
           ( 50.00%)  -0.7071* C  4 s( 19.70%)p 4.07( 80.18%)d 0.01(  0.11%)
                                     0.0000  0.4438  0.0076 -0.0025 -0.5447
```

```
                                     0.0183 -0.5447  0.0183  0.4545  0.0337
                                     0.0291 -0.0003 -0.0003  0.0000 -0.0168
 23. (0.02778) BD*( 1) C  3- H  7
            ( 36.01%)   0.6001* C  3 s( 40.52%)p 1.47( 59.44%)d 0.00(  0.04%)
                                     0.0000 -0.6365  0.0080 -0.0031  0.4451
                                    -0.0016  0.4451 -0.0016 -0.4451  0.0016
                                    -0.0119  0.0119  0.0119  0.0000  0.0000
            ( 63.99%)  -0.7999* H  7 s( 99.95%)p 0.00(  0.05%)
                                    -0.9998 -0.0034 -0.0126 -0.0126  0.0126
 24. (0.02778) BD*( 1) C  4- H  8
            ( 36.01%)   0.6001* C  4 s( 40.52%)p 1.47( 59.44%)d 0.00(  0.04%)
                                     0.0000 -0.6365  0.0080 -0.0031 -0.4451
                                     0.0016 -0.4451  0.0016 -0.4451  0.0016
                                    -0.0119 -0.0119 -0.0119  0.0000  0.0000
            ( 63.99%)  -0.7999* H  8 s( 99.95%)p 0.00(  0.05%)
                                    -0.9998 -0.0034  0.0126  0.0126  0.0126
 25. (0.00278) RY ( 1) C  1          s(  0.00%)p 1.00( 68.66%)d 0.46( 31.34%)
                                     0.0000  0.0000  0.0000  0.0000 -0.0145
                                     0.6761 -0.0069  0.3223 -0.0076  0.3538
                                    -0.0849 -0.0773 -0.1622 -0.4461  0.2738
 26. (0.00278) RY ( 2) C  1          s(  0.00%)p 1.00( 68.66%)d 0.46( 31.34%)
                                     0.0000  0.0000  0.0000  0.0000 -0.0004
                                     0.0182 -0.0127  0.5946  0.0123 -0.5765
                                     0.1383 -0.1427 -0.0044 -0.2738 -0.4461
 27. (0.00159) RY ( 3) C  1          s( 32.82%)p 0.79( 26.02%)d 1.25( 41.16%)
                                     0.0000 -0.0070  0.5648  0.0956  0.0175
                                     0.2940 -0.0175 -0.2940 -0.0175 -0.2940
                                     0.3704  0.3704 -0.3704  0.0000  0.0000
 28. (0.00025) RY ( 4) C  1          s( 36.05%)p 1.70( 61.44%)d 0.07(  2.50%)
                                     0.0000  0.0437  0.5631 -0.2038 -0.0161
                                    -0.4523  0.0161  0.4523  0.0161  0.4523
                                     0.0913  0.0913 -0.0913  0.0000  0.0000
 29. (0.00004) RY ( 5) C  1          s(  0.00%)p 1.00( 24.90%)d 3.02( 75.10%)
 30. (0.00004) RY ( 6) C  1          s(  0.00%)p 1.00( 24.90%)d 3.02( 75.10%)
 31. (0.00000) RY ( 7) C  1          s( 83.49%)p 0.11(  9.36%)d 0.09(  7.15%)
 32. (0.00000) RY ( 8) C  1          s(  0.00%)p 1.00(  6.58%)d14.20( 93.42%)
 33. (0.00000) RY ( 9) C  1          s(  0.00%)p 1.00(  6.58%)d14.20( 93.42%)
 34. (0.00000) RY (10) C  1          s( 48.01%)p 0.06(  2.92%)d 1.02( 49.07%)
 35. (0.00278) RY ( 1) C  2          s(  0.00%)p 1.00( 68.66%)d 0.46( 31.34%)
                                     0.0000  0.0000  0.0000  0.0000 -0.0125
                                     0.5854 -0.0125  0.5861  0.0000  0.0007
                                    -0.0002 -0.1406 -0.1404  0.5234  0.0005
 36. (0.00278) RY ( 2) C  2          s(  0.00%)p 1.00( 68.66%)d 0.46( 31.34%)
                                     0.0000  0.0000  0.0000  0.0000  0.0072
                                    -0.3388 -0.0072  0.3376 -0.0145  0.6764
                                    -0.1623 -0.0810  0.0813 -0.0005  0.5234
 37. (0.00159) RY ( 3) C  2          s( 32.82%)p 0.79( 26.02%)d 1.25( 41.16%)
                                     0.0000 -0.0070  0.5648  0.0956 -0.0175
                                    -0.2940  0.0175  0.2940 -0.0175 -0.2940
                                     0.3704 -0.3704  0.3704  0.0000  0.0000
 38. (0.00025) RY ( 4) C  2          s( 36.05%)p 1.70( 61.44%)d 0.07(  2.50%)
                                     0.0000  0.0437  0.5631 -0.2038  0.0161
                                     0.4523 -0.0161 -0.4523  0.0161  0.4523
                                     0.0913 -0.0913  0.0913  0.0000  0.0000
 39. (0.00004) RY ( 5) C  2          s(  0.00%)p 1.00( 24.90%)d 3.02( 75.10%)
 40. (0.00004) RY ( 6) C  2          s(  0.00%)p 1.00( 24.90%)d 3.02( 75.10%)
 41. (0.00000) RY ( 7) C  2          s( 83.49%)p 0.11(  9.36%)d 0.09(  7.15%)
 42. (0.00000) RY ( 8) C  2          s(  0.00%)p 1.00(  6.58%)d14.20( 93.42%)
 43. (0.00000) RY ( 9) C  2          s(  0.00%)p 1.00(  6.58%)d14.20( 93.42%)
 44. (0.00000) RY (10) C  2          s( 48.01%)p 0.06(  2.92%)d 1.02( 49.07%)
 45. (0.00278) RY ( 1) C  3          s(  0.00%)p 1.00( 68.66%)d 0.46( 31.34%)
                                     0.0000  0.0000  0.0000  0.0000  0.0125
```

```
                                         -0.5858 -0.0125  0.5858  0.0000  0.0000
                                          0.0000 -0.1405  0.1405 -0.5234  0.0000
  46. (0.00278) RY ( 2) C  3            s(  0.00%)p 1.00( 68.66%)d 0.46( 31.34%)
                                          0.0000  0.0000  0.0000  0.0000 -0.0072
                                          0.3382 -0.0072  0.3382 -0.0145  0.6764
                                         -0.1623 -0.0811 -0.0811  0.0000 -0.5234
  47. (0.00159) RY ( 3) C  3            s( 32.82%)p 0.79( 26.02%)d 1.25( 41.16%)
                                          0.0000 -0.0070  0.5648  0.0956 -0.0175
                                         -0.2940 -0.0175 -0.2940  0.0175  0.2940
                                         -0.3704  0.3704  0.3704  0.0000  0.0000
  48. (0.00025) RY ( 4) C  3            s( 36.05%)p 1.70( 61.44%)d 0.07(  2.50%)
                                          0.0000  0.0437  0.5631 -0.2038  0.0161
                                          0.4523  0.0161  0.4523 -0.0161 -0.4523
                                         -0.0913  0.0913  0.0913  0.0000  0.0000
  49. (0.00004) RY ( 5) C  3            s(  0.00%)p 1.00( 24.90%)d 3.02( 75.10%)
  50. (0.00004) RY ( 6) C  3            s(  0.00%)p 1.00( 24.90%)d 3.02( 75.10%)
  51. (0.00000) RY ( 7) C  3            s( 83.49%)p 0.11(  9.36%)d 0.09(  7.15%)
  52. (0.00000) RY ( 8) C  3            s(  0.00%)p 1.00(  6.58%)d14.20( 93.42%)
  53. (0.00000) RY ( 9) C  3            s(  0.00%)p 1.00(  6.58%)d14.20( 93.42%)
  54. (0.00000) RY (10) C  3            s( 48.01%)p 0.06(  2.92%)d 1.02( 49.07%)
  55. (0.00278) RY ( 1) C  4            s(  0.00%)p 1.00( 68.66%)d 0.46( 31.34%)
                                          0.0000  0.0000  0.0000  0.0000 -0.0125
                                          0.5856  0.0125 -0.5860  0.0000  0.0004
                                         -0.0001  0.1406 -0.1405 -0.5234 -0.0003
  56. (0.00278) RY ( 2) C  4            s(  0.00%)p 1.00( 68.66%)d 0.46( 31.34%)
                                          0.0000  0.0000  0.0000  0.0000  0.0072
                                         -0.3385  0.0072 -0.3378 -0.0145  0.6764
                                         -0.1623  0.0811  0.0812  0.0003 -0.5234
  57. (0.00159) RY ( 3) C  4            s( 32.82%)p 0.79( 26.02%)d 1.25( 41.16%)
                                          0.0000 -0.0070  0.5648  0.0956  0.0175
                                          0.2940  0.0175  0.2940  0.0175  0.2940
                                         -0.3704 -0.3704 -0.3704  0.0000  0.0000
  58. (0.00025) RY ( 4) C  4            s( 36.05%)p 1.70( 61.44%)d 0.07(  2.50%)
                                          0.0000  0.0437  0.5631 -0.2038 -0.0161
                                         -0.4523 -0.0161 -0.4523 -0.0161 -0.4523
                                         -0.0913 -0.0913 -0.0913  0.0000  0.0000
  59. (0.00004) RY ( 5) C  4            s(  0.00%)p 1.00( 24.90%)d 3.02( 75.10%)
  60. (0.00004) RY ( 6) C  4            s(  0.00%)p 1.00( 24.90%)d 3.02( 75.10%)
  61. (0.00000) RY ( 7) C  4            s( 83.49%)p 0.11(  9.36%)d 0.09(  7.15%)
  62. (0.00000) RY ( 8) C  4            s(  0.00%)p 1.00(  6.58%)d14.20( 93.42%)
  63. (0.00000) RY ( 9) C  4            s(  0.00%)p 1.00(  6.58%)d14.20( 93.42%)
  64. (0.00000) RY (10) C  4            s( 48.01%)p 0.06(  2.92%)d 1.02( 49.07%)
  65. (0.00034) RY ( 1) H  5            s( 99.87%)p 0.00(  0.13%)
                                         -0.0026  0.9993  0.0212 -0.0212 -0.0212
  66. (0.00013) RY ( 2) H  5            s(  0.00%)p 1.00(100.00%)
                                          0.0000  0.0000  0.7071  0.7072 -0.0001
  67. (0.00013) RY ( 3) H  5            s(  0.00%)p 1.00(100.00%)
                                          0.0000  0.0000  0.4083 -0.4082  0.8165
  68. (0.00000) RY ( 4) H  5            s(  0.18%)p99.99( 99.82%)
  69. (0.00034) RY ( 1) H  6            s( 99.87%)p 0.00(  0.13%)
                                         -0.0026  0.9993 -0.0212  0.0212 -0.0212
  70. (0.00013) RY ( 2) H  6            s(  0.00%)p 1.00(100.00%)
                                          0.0000  0.0000  0.7071  0.7071  0.0000
  71. (0.00013) RY ( 3) H  6            s(  0.00%)p 1.00(100.00%)
                                          0.0000  0.0000 -0.4082  0.4082  0.8165
  72. (0.00000) RY ( 4) H  6            s(  0.18%)p99.99( 99.82%)
  73. (0.00034) RY ( 1) H  7            s( 99.87%)p 0.00(  0.13%)
                                         -0.0026  0.9993 -0.0212 -0.0212  0.0212
  74. (0.00013) RY ( 2) H  7            s(  0.00%)p 1.00(100.00%)
                                          0.0000  0.0000 -0.7071  0.7072  0.0001
  75. (0.00013) RY ( 3) H  7            s(  0.00%)p 1.00(100.00%)
                                          0.0000  0.0000  0.4083  0.4082  0.8165
```

```
 76. (0.00000) RY ( 4) H  7           s(  0.18%)p99.99( 99.82%)
 77. (0.00034) RY ( 1) H  8           s( 99.87%)p 0.00(  0.13%)
                                      -0.0026  0.9993  0.0212  0.0212  0.0212
 78. (0.00013) RY ( 2) H  8           s(  0.00%)p 1.00(100.00%)
                                       0.0000  0.0000  0.7071 -0.7071  0.0000
 79. (0.00013) RY ( 3) H  8           s(  0.00%)p 1.00(100.00%)
                                       0.0000  0.0000 -0.4082 -0.4082  0.8165
 80. (0.00000) RY ( 4) H  8           s(  0.18%)p99.99( 99.82%)


NHO DIRECTIONALITY AND BOND BENDING (deviation from line of nuclear centers at
                                     the position of maximum hybrid amplitude)

        [Thresholds for printing:  angular deviation  >  1.0 degree]
                                   p- or d-character  > 25.0%
                                   orbital occupancy  >  0.10e

                       Line of Centers        Hybrid 1           Hybrid 2
                       ---------------  -------------------  ------------------
          NBO           Theta   Phi    Theta   Phi    Dev    Theta   Phi    Dev
===============================================================================
  5. BD ( 1) C  1- C  2   90.0  135.0   114.6  135.0  24.6   114.6  315.0  24.6
  6. BD ( 1) C  1- C  3   45.0  180.0    50.0  212.9  24.6   130.0  327.1  24.6
  7. BD ( 1) C  1- C  4   45.0   90.0    50.0   57.1  24.6   130.0  302.9  24.6
  9. BD ( 1) C  2- C  3   45.0  270.0    50.0  237.1  24.6   130.0  122.9  24.6
 10. BD ( 1) C  2- C  4   45.0    0.0    50.0   32.9  24.6   130.0  147.1  24.6
 12. BD ( 1) C  3- C  4   90.0   45.0    65.4   45.0  24.6    65.4  225.0  24.6


SECOND ORDER PERTURBATION THEORY ANALYSIS OF FOCK MATRIX IN NBO BASIS

    Threshold for printing:   0.50 kcal/mol
                                                          E(2) E(NL)-E(L) F(L,NL)
     Donor (L) NBO              Acceptor (NL) NBO      kcal/mol   a.u.    a.u.
===============================================================================

within unit  1
   5. BD ( 1) C  1- C  2      16. BD*( 1) C  1- C  3      3.78    0.78   0.049
   5. BD ( 1) C  1- C  2      17. BD*( 1) C  1- C  4      3.78    0.78   0.049
   5. BD ( 1) C  1- C  2      18. BD*( 1) C  1- H  5      0.64    0.93   0.022
   5. BD ( 1) C  1- C  2      19. BD*( 1) C  2- C  3      3.78    0.78   0.049
   5. BD ( 1) C  1- C  2      20. BD*( 1) C  2- C  4      3.78    0.78   0.049
   5. BD ( 1) C  1- C  2      21. BD*( 1) C  2- H  6      0.64    0.93   0.022
   5. BD ( 1) C  1- C  2      23. BD*( 1) C  3- H  7      9.23    0.93   0.083
   5. BD ( 1) C  1- C  2      24. BD*( 1) C  4- H  8      9.23    0.93   0.083
   5. BD ( 1) C  1- C  2      46. RY ( 2) C  3            2.04    1.43   0.048
   5. BD ( 1) C  1- C  2      56. RY ( 2) C  4            2.04    1.43   0.048
   6. BD ( 1) C  1- C  3      15. BD*( 1) C  1- C  2      3.78    0.78   0.049
   6. BD ( 1) C  1- C  3      17. BD*( 1) C  1- C  4      3.78    0.78   0.049
   6. BD ( 1) C  1- C  3      18. BD*( 1) C  1- H  5      0.64    0.93   0.022
   6. BD ( 1) C  1- C  3      19. BD*( 1) C  2- C  3      3.78    0.78   0.049
   6. BD ( 1) C  1- C  3      21. BD*( 1) C  2- H  6      9.23    0.93   0.083
   6. BD ( 1) C  1- C  3      22. BD*( 1) C  3- C  4      3.78    0.78   0.049
   6. BD ( 1) C  1- C  3      23. BD*( 1) C  3- H  7      0.64    0.93   0.022
   6. BD ( 1) C  1- C  3      24. BD*( 1) C  4- H  8      9.23    0.93   0.083
   6. BD ( 1) C  1- C  3      35. RY ( 1) C  2            1.53    1.43   0.042
   6. BD ( 1) C  1- C  3      36. RY ( 2) C  2            0.51    1.43   0.024
   6. BD ( 1) C  1- C  3      55. RY ( 1) C  4            1.53    1.43   0.042
   6. BD ( 1) C  1- C  3      56. RY ( 2) C  4            0.51    1.43   0.024
   7. BD ( 1) C  1- C  4      15. BD*( 1) C  1- C  2      3.78    0.78   0.049
   7. BD ( 1) C  1- C  4      16. BD*( 1) C  1- C  3      3.78    0.78   0.049
   7. BD ( 1) C  1- C  4      18. BD*( 1) C  1- H  5      0.64    0.93   0.022
```

```
   7. BD ( 1) C  1- C  4      20. BD*( 1) C  2- C  4      3.78    0.78   0.049
   7. BD ( 1) C  1- C  4      21. BD*( 1) C  2- H  6      9.23    0.93   0.083
   7. BD ( 1) C  1- C  4      22. BD*( 1) C  3- C  4      3.78    0.78   0.049
   7. BD ( 1) C  1- C  4      23. BD*( 1) C  3- H  7      9.23    0.93   0.083
   7. BD ( 1) C  1- C  4      24. BD*( 1) C  4- H  8      0.64    0.93   0.022
   7. BD ( 1) C  1- C  4      35. RY ( 1) C  2            1.53    1.43   0.042
   7. BD ( 1) C  1- C  4      36. RY ( 2) C  2            0.51    1.43   0.024
   7. BD ( 1) C  1- C  4      45. RY ( 1) C  3            1.53    1.43   0.042
   7. BD ( 1) C  1- C  4      46. RY ( 2) C  3            0.51    1.43   0.024
   9. BD ( 1) C  2- C  3      15. BD*( 1) C  1- C  2      3.78    0.78   0.049
   9. BD ( 1) C  2- C  3      16. BD*( 1) C  1- C  3      3.78    0.78   0.049
   9. BD ( 1) C  2- C  3      18. BD*( 1) C  1- H  5      9.23    0.93   0.083
   9. BD ( 1) C  2- C  3      20. BD*( 1) C  2- C  4      3.78    0.78   0.049
   9. BD ( 1) C  2- C  3      21. BD*( 1) C  2- H  6      0.64    0.93   0.022
   9. BD ( 1) C  2- C  3      22. BD*( 1) C  3- C  4      3.78    0.78   0.049
   9. BD ( 1) C  2- C  3      23. BD*( 1) C  3- H  7      0.64    0.93   0.022
   9. BD ( 1) C  2- C  3      24. BD*( 1) C  4- H  8      9.23    0.93   0.083
   9. BD ( 1) C  2- C  3      25. RY ( 1) C  1            2.04    1.43   0.048
   9. BD ( 1) C  2- C  3      55. RY ( 1) C  4            1.53    1.43   0.042
   9. BD ( 1) C  2- C  3      56. RY ( 2) C  4            0.51    1.43   0.024
  10. BD ( 1) C  2- C  4      15. BD*( 1) C  1- C  2      3.78    0.78   0.049
  10. BD ( 1) C  2- C  4      17. BD*( 1) C  1- C  4      3.78    0.78   0.049
  10. BD ( 1) C  2- C  4      18. BD*( 1) C  1- H  5      9.23    0.93   0.083
  10. BD ( 1) C  2- C  4      19. BD*( 1) C  2- C  3      3.78    0.78   0.049
  10. BD ( 1) C  2- C  4      21. BD*( 1) C  2- H  6      0.64    0.93   0.022
  10. BD ( 1) C  2- C  4      22. BD*( 1) C  3- C  4      3.78    0.78   0.049
  10. BD ( 1) C  2- C  4      23. BD*( 1) C  3- H  7      9.23    0.93   0.083
  10. BD ( 1) C  2- C  4      24. BD*( 1) C  4- H  8      0.64    0.93   0.022
  10. BD ( 1) C  2- C  4      26. RY ( 2) C  1            1.58    1.43   0.042
  10. BD ( 1) C  2- C  4      45. RY ( 1) C  3            1.53    1.43   0.042
  10. BD ( 1) C  2- C  4      46. RY ( 2) C  3            0.51    1.43   0.024
  12. BD ( 1) C  3- C  4      16. BD*( 1) C  1- C  3      3.78    0.78   0.049
  12. BD ( 1) C  3- C  4      17. BD*( 1) C  1- C  4      3.78    0.78   0.049
  12. BD ( 1) C  3- C  4      18. BD*( 1) C  1- H  5      9.23    0.93   0.083
  12. BD ( 1) C  3- C  4      19. BD*( 1) C  2- C  3      3.78    0.78   0.049
  12. BD ( 1) C  3- C  4      20. BD*( 1) C  2- C  4      3.78    0.78   0.049
  12. BD ( 1) C  3- C  4      21. BD*( 1) C  2- H  6      9.23    0.93   0.083
  12. BD ( 1) C  3- C  4      23. BD*( 1) C  3- H  7      0.64    0.93   0.022
  12. BD ( 1) C  3- C  4      24. BD*( 1) C  4- H  8      0.64    0.93   0.022
  12. BD ( 1) C  3- C  4      25. RY ( 1) C  1            0.56    1.43   0.025
  12. BD ( 1) C  3- C  4      26. RY ( 2) C  1            1.48    1.43   0.041
  12. BD ( 1) C  3- C  4      36. RY ( 2) C  2            2.04    1.43   0.048


NATURAL BOND ORBITALS (Summary):

                                                     Principal Delocalizations
          NBO                 Occupancy    Energy   (geminal,vicinal,remote)
===============================================================================
Molecular unit  1  (C4H4)
------ Lewis --------------------------------------
   1. CR ( 1) C  1             1.99999   -10.19551
   2. CR ( 1) C  2             1.99999   -10.19551
   3. CR ( 1) C  3             1.99999   -10.19551
   4. CR ( 1) C  4             1.99999   -10.19551
   5. BD ( 1) C  1- C  2       1.96127    -0.43543  23(v),24(v),16(g),17(g)
                                                    19(g),20(g),46(v),56(v)
                                                    18(g),21(g)
   6. BD ( 1) C  1- C  3       1.96127    -0.43543  21(v),24(v),15(g),17(g)
                                                    19(g),22(g),35(v),55(v)
                                                    18(g),23(g),56(v),36(v)
   7. BD ( 1) C  1- C  4       1.96127    -0.43543  21(v),23(v),15(g),16(g)
```

```
                                                                20(g),22(g),45(v),35(v)
                                                                18(g),24(g),36(v),46(v)
   8. BD ( 1) C  1- H  5       1.99742    -0.51530
   9. BD ( 1) C  2- C  3       1.96127    -0.43543  18(v),24(v),15(g),16(g)
                                                    20(g),22(g),25(v),55(v)
                                                    21(g),23(g),56(v)
  10. BD ( 1) C  2- C  4       1.96127    -0.43543  18(v),23(v),15(g),17(g)
                                                    19(g),22(g),26(v),45(v)
                                                    21(g),24(g),46(v)
  11. BD ( 1) C  2- H  6       1.99742    -0.51530
  12. BD ( 1) C  3- C  4       1.96127    -0.43543  18(v),21(v),16(g),17(g)
                                                    19(g),20(g),36(v),26(v)
                                                    23(g),24(g),25(v)
  13. BD ( 1) C  3- H  7       1.99742    -0.51530
  14. BD ( 1) C  4- H  8       1.99742    -0.51530
------ non-Lewis ----------------------------------
  15. BD*( 1) C  1- C  2       0.01653     0.34664
  16. BD*( 1) C  1- C  3       0.01653     0.34664
  17. BD*( 1) C  1- C  4       0.01653     0.34664
  18. BD*( 1) C  1- H  5       0.02778     0.49644
  19. BD*( 1) C  2- C  3       0.01653     0.34664
  20. BD*( 1) C  2- C  4       0.01653     0.34664
  21. BD*( 1) C  2- H  6       0.02778     0.49644
  22. BD*( 1) C  3- C  4       0.01653     0.34664
  23. BD*( 1) C  3- H  7       0.02778     0.49644
  24. BD*( 1) C  4- H  8       0.02778     0.49644
  25. RY ( 1) C  1             0.00278     0.99252
  26. RY ( 2) C  1             0.00278     0.99252
  27. RY ( 3) C  1             0.00159     1.81349
  28. RY ( 4) C  1             0.00025     0.88852
  29. RY ( 5) C  1             0.00004     1.55532
  30. RY ( 6) C  1             0.00004     1.55532
  31. RY ( 7) C  1             0.00000     3.88649
  32. RY ( 8) C  1             0.00000     2.20581
  33. RY ( 9) C  1             0.00000     2.20581
  34. RY (10) C  1             0.00000     2.86595
  35. RY ( 1) C  2             0.00278     0.99252
  36. RY ( 2) C  2             0.00278     0.99252
  37. RY ( 3) C  2             0.00159     1.81349
  38. RY ( 4) C  2             0.00025     0.88852
  39. RY ( 5) C  2             0.00004     1.55532
  40. RY ( 6) C  2             0.00004     1.55532
  41. RY ( 7) C  2             0.00000     3.88649
  42. RY ( 8) C  2             0.00000     2.20581
  43. RY ( 9) C  2             0.00000     2.20581
  44. RY (10) C  2             0.00000     2.86595
  45. RY ( 1) C  3             0.00278     0.99252
  46. RY ( 2) C  3             0.00278     0.99252
  47. RY ( 3) C  3             0.00159     1.81349
  48. RY ( 4) C  3             0.00025     0.88852
  49. RY ( 5) C  3             0.00004     1.55532
  50. RY ( 6) C  3             0.00004     1.55532
  51. RY ( 7) C  3             0.00000     3.88649
  52. RY ( 8) C  3             0.00000     2.20581
  53. RY ( 9) C  3             0.00000     2.20581
  54. RY (10) C  3             0.00000     2.86595
  55. RY ( 1) C  4             0.00278     0.99252
  56. RY ( 2) C  4             0.00278     0.99252
  57. RY ( 3) C  4             0.00159     1.81349
  58. RY ( 4) C  4             0.00025     0.88852
  59. RY ( 5) C  4             0.00004     1.55532
  60. RY ( 6) C  4             0.00004     1.55532
```

```
  61. RY ( 7) C  4             0.00000     3.88649
  62. RY ( 8) C  4             0.00000     2.20581
  63. RY ( 9) C  4             0.00000     2.20581
  64. RY (10) C  4             0.00000     2.86595
  65. RY ( 1) H  5             0.00034     0.53040
  66. RY ( 2) H  5             0.00013     2.35924
  67. RY ( 3) H  5             0.00013     2.35924
  68. RY ( 4) H  5             0.00000     3.22337
  69. RY ( 1) H  6             0.00034     0.53040
  70. RY ( 2) H  6             0.00013     2.35924
  71. RY ( 3) H  6             0.00013     2.35924
  72. RY ( 4) H  6             0.00000     3.22337
  73. RY ( 1) H  7             0.00034     0.53040
  74. RY ( 2) H  7             0.00013     2.35924
  75. RY ( 3) H  7             0.00013     2.35924
  76. RY ( 4) H  7             0.00000     3.22337
  77. RY ( 1) H  8             0.00034     0.53040
  78. RY ( 2) H  8             0.00013     2.35924
  79. RY ( 3) H  8             0.00013     2.35924
  80. RY ( 4) H  8             0.00000     3.22337
         -------------------------------
                Total Lewis   27.75728  ( 99.1332%)
          Valence non-Lewis    0.21031  (  0.7511%)
          Rydberg non-Lewis    0.03241  (  0.1157%)
         -------------------------------
              Total unit  1   28.00000  (100.0000%)
             Charge unit  1    0.00000

$CHOOSE
  BOND S 1 2 S 1 3 S 1 4 S 1 5 S 2 3 S 2 4 S 2 6 S 3 4 S 3 7 S 4 8 END
$END


NATURAL RESONANCE THEORY ANALYSIS:

Maximum reference structures : 20
Maximum resonance structures : 5000
Memory requirements : 91593218 words of 149780616 available

13 candidate reference structure(s) calculated by SR LEWIS
Initial loops searched 13 bonding pattern(s); 1 was retained
Delocalization list threshold set to 1.00 kcal/mol for reference 1
Reference   1:  rho*=0.24272, f(w)=0.90306 converged after   5 iterations

                                              fractional accuracy f(w)
                non-Lewis              ------------------------------------
 Ref     Wgt      density      d(0)      all NBOs     val+core     valence
----------------------------------------------------------------------------
  1    1.00000    0.24272    0.01315     0.90306      0.93565      0.93565


TOPO matrix for the leading resonance structure:

    Atom  1   2   3   4   5   6   7   8
    ---- --- --- --- --- --- --- --- ---
  1.  C   0   1   1   1   1   0   0   0
  2.  C   1   0   1   1   0   1   0   0
  3.  C   1   1   0   1   0   0   1   0
  4.  C   1   1   1   0   0   0   0   1
  5.  H   1   0   0   0   0   0   0   0
  6.  H   0   1   0   0   0   0   0   0
  7.  H   0   0   1   0   0   0   0   0
```

```
  8.  H    0    0    0    1    0    0    0    0

       Resonance
   RS   Weight(%)                  Added(Removed)
---------------------------------------------------------------------------
   1*     88.79
   2 (2)   0.45   ( C  1- C  2), ( C  1- C  3),  C  2- C  3,  C  1
   3 (2)   0.45   ( C  1- C  2), ( C  1- C  4),  C  2- C  4,  C  1
   4 (2)   0.45   ( C  1- C  2),  C  1- C  3, ( C  2- C  3),  C  2
   5 (2)   0.45   ( C  1- C  2),  C  1- C  4, ( C  2- C  4),  C  2
   6 (2)   0.45   ( C  1- C  3), ( C  1- C  4),  C  3- C  4,  C  1
   7 (2)   0.45    C  1- C  2, ( C  1- C  3), ( C  2- C  3),  C  3
   8 (2)   0.45   ( C  1- C  3),  C  1- C  4, ( C  3- C  4),  C  3
   9 (2)   0.45    C  1- C  2, ( C  1- C  4), ( C  2- C  4),  C  4
  10 (2)   0.45    C  1- C  3, ( C  1- C  4), ( C  3- C  4),  C  4
  11 (2)   0.45   ( C  2- C  3), ( C  2- C  4),  C  3- C  4,  C  2
  12 (2)   0.45   ( C  2- C  3),  C  2- C  4, ( C  3- C  4),  C  3
  13 (2)   0.45    C  2- C  3, ( C  2- C  4), ( C  3- C  4),  C  4
  14       0.24   ( C  1- C  2),  C  1- C  3, ( C  3- H  7),  H  7
  15       0.24   ( C  1- C  2),  C  2- C  3, ( C  3- H  7),  H  7
  16       0.24   ( C  1- C  2),  C  1- C  4, ( C  4- H  8),  H  8
  17       0.24   ( C  1- C  2),  C  2- C  4, ( C  4- H  8),  H  8
  18       0.24    C  1- C  2, ( C  1- C  3), ( C  2- H  6),  H  6
  19       0.24   ( C  1- C  3),  C  2- C  3, ( C  2- H  6),  H  6
  20       0.24   ( C  1- C  3),  C  1- C  4, ( C  4- H  8),  H  8
  21       0.24   ( C  1- C  3),  C  3- C  4, ( C  4- H  8),  H  8
  22       0.24    C  1- C  2, ( C  1- C  4), ( C  2- H  6),  H  6
  23       0.24   ( C  1- C  4),  C  2- C  4, ( C  2- H  6),  H  6
  24       0.24    C  1- C  3, ( C  1- C  4), ( C  3- H  7),  H  7
  25       0.24   ( C  1- C  4),  C  3- C  4, ( C  3- H  7),  H  7
  26       0.24    C  1- C  2, ( C  1- H  5), ( C  2- C  3),  H  5
  27       0.24    C  1- C  3, ( C  1- H  5), ( C  2- C  3),  H  5
  28       0.24   ( C  2- C  3),  C  2- C  4, ( C  4- H  8),  H  8
  29       0.24   ( C  2- C  3),  C  3- C  4, ( C  4- H  8),  H  8
  30       0.24    C  1- C  2, ( C  1- H  5), ( C  2- C  4),  H  5
  31       0.24    C  1- C  4, ( C  1- H  5), ( C  2- C  4),  H  5
  32       0.24    C  2- C  3, ( C  2- C  4), ( C  3- H  7),  H  7
  33       0.24   ( C  2- C  4),  C  3- C  4, ( C  3- H  7),  H  7
  34       0.24    C  1- C  3, ( C  1- H  5), ( C  3- C  4),  H  5
  35       0.24    C  1- C  4, ( C  1- H  5), ( C  3- C  4),  H  5
  36       0.24    C  2- C  3, ( C  2- H  6), ( C  3- C  4),  H  6
  37       0.24    C  2- C  4, ( C  2- H  6), ( C  3- C  4),  H  6
---------------------------------------------------------------------------
        100.00   * Total *                [* = reference structure]


Natural Bond Order:  (total/covalent/ionic)

    Atom       1      2      3      4      5      6      7      8
    ----    ------ ------ ------ ------ ------ ------ ------ ------
  1.  C  t 0.0134 0.9910 0.9910 0.9910 0.9854 0.0000 0.0000 0.0000
         c   ---  0.9724 0.9724 0.9724 0.7097 0.0000 0.0000 0.0000
         i   ---  0.0186 0.0186 0.0186 0.2757 0.0000 0.0000 0.0000

  2.  C  t 0.9910 0.0134 0.9910 0.9910 0.0000 0.9854 0.0000 0.0000
         c 0.9724   ---  0.9724 0.9724 0.0000 0.7097 0.0000 0.0000
         i 0.0186   ---  0.0186 0.0186 0.0000 0.2757 0.0000 0.0000

  3.  C  t 0.9910 0.9910 0.0134 0.9910 0.0000 0.0000 0.9854 0.0000
         c 0.9724 0.9724   ---  0.9724 0.0000 0.0000 0.7097 0.0000
         i 0.0186 0.0186   ---  0.0186 0.0000 0.0000 0.2757 0.0000
```

```
  4.  C  t 0.9910 0.9910 0.9910 0.0134 0.0000 0.0000 0.0000 0.9854
         c 0.9724 0.9724 0.9724   ---  0.0000 0.0000 0.0000 0.7097
         i 0.0186 0.0186 0.0186   ---  0.0000 0.0000 0.0000 0.2757

  5.  H  t 0.9854 0.0000 0.0000 0.0000 0.0146 0.0000 0.0000 0.0000
         c 0.7097 0.0000 0.0000 0.0000   ---  0.0000 0.0000 0.0000
         i 0.2757 0.0000 0.0000 0.0000   ---  0.0000 0.0000 0.0000

  6.  H  t 0.0000 0.9854 0.0000 0.0000 0.0000 0.0146 0.0000 0.0000
         c 0.0000 0.7097 0.0000 0.0000 0.0000   ---  0.0000 0.0000
         i 0.0000 0.2757 0.0000 0.0000 0.0000   ---  0.0000 0.0000

  7.  H  t 0.0000 0.0000 0.9854 0.0000 0.0000 0.0000 0.0146 0.0000
         c 0.0000 0.0000 0.7097 0.0000 0.0000 0.0000   ---  0.0000
         i 0.0000 0.0000 0.2757 0.0000 0.0000 0.0000   ---  0.0000

  8.  H  t 0.0000 0.0000 0.0000 0.9854 0.0000 0.0000 0.0000 0.0146
         c 0.0000 0.0000 0.0000 0.7097 0.0000 0.0000 0.0000   ---
         i 0.0000 0.0000 0.0000 0.2757 0.0000 0.0000 0.0000   ---


Natural Atomic Valencies:

                    Co-    Electro-
    Atom  Valency  Valency  Valency
    ----  -------  -------  -------
  1.  C    3.9586   3.6269   0.3316
  2.  C    3.9586   3.6269   0.3316
  3.  C    3.9586   3.6269   0.3316
  4.  C    3.9586   3.6269   0.3316
  5.  H    0.9854   0.7097   0.2757
  6.  H    0.9854   0.7097   0.2757
  7.  H    0.9854   0.7097   0.2757
  8.  H    0.9854   0.7097   0.2757


$NRTSTR
  STR        ! Wgt = 88.79%
    BOND S 1 2 S 1 3 S 1 4 S 1 5 S 2 3 S 2 4 S 2 6 S 3 4 S 3 7 S 4 8 END
  END
$END

NBO analysis completed in 0.17 CPU seconds (0 wall seconds)
Maximum scratch memory used by NBO was 91886785 words (701.04 MB)
..... done with NBO analysis .....
```

S3.6.2 Phosphatetrahedrane

```
********************************** NBO 6.0 **********************************
          N A T U R A L   A T O M I C   O R B I T A L   A N D
       N A T U R A L   B O N D   O R B I T A L   A N A L Y S I S
**************** Massachusetts Institute of Technology (100648) ***************
 (c) Copyright 1996-2018 Board of Regents of the University of Wisconsin System
     on behalf of the Theoretical Chemistry Institute.  All rights reserved.

         Cite this program [NBO 6.0.18a (11-Mar-2018)] as:

         NBO 6.0.  E. D. Glendening, J. K. Badenhoop, A. E. Reed,
         J. E. Carpenter, J. A. Bohmann, C. M. Morales, C. R. Landis,
```

```
         and F. Weinhold (Theoretical Chemistry Institute, University
         of Wisconsin, Madison, WI, 2013); http://nbo6.chem.wisc.edu/

      /MEMORY / : Allocate 150000000 words of dynamic memory
      /NBO    / : Natural Bond Orbital Analysis
      /NRT    / : Natural Resonance Theory Analysis
      /MOLDEN / : Write NBOs to MOLDEN-formatted file

Job title:  phosphatetrahedrane optimization


NATURAL POPULATIONS:  Natural atomic orbital occupancies

 NAO Atom No lang   Type(AO)    Occupancy      Energy
 -------------------------------------------------------
   1    P  1  s      Cor( 1s)     2.00000     -75.96290
   2    P  1  s      Cor( 2s)     1.99999      -7.66537
   3    P  1  s      Val( 3s)     1.72499      -0.39674
   4    P  1  s      Ryd( 4s)     0.00424       0.47309
   5    P  1  s      Ryd( 5s)     0.00001       3.51120
   6    P  1  px     Cor( 2p)     1.99999      -4.70809
   7    P  1  px     Val( 3p)     0.95199      -0.10405
   8    P  1  px     Ryd( 4p)     0.00178       0.38234
   9    P  1  py     Cor( 2p)     1.99999      -4.70809
  10    P  1  py     Val( 3p)     0.95199      -0.10405
  11    P  1  py     Ryd( 4p)     0.00178       0.38234
  12    P  1  pz     Cor( 2p)     1.99998      -4.71152
  13    P  1  pz     Val( 3p)     0.80123      -0.04175
  14    P  1  pz     Ryd( 4p)     0.00108       0.38270
  15    P  1  dxy    Ryd( 3d)     0.00232       0.77009
  16    P  1  dxz    Ryd( 3d)     0.00706       1.09791
  17    P  1  dyz    Ryd( 3d)     0.00706       1.09791
  18    P  1  dx2y2  Ryd( 3d)     0.00232       0.77009
  19    P  1  dz2    Ryd( 3d)     0.00661       1.04024

  20    C  2  s      Cor( 1s)     1.99999     -10.21276
  21    C  2  s      Val( 2s)     1.01977      -0.19466
  22    C  2  s      Ryd( 3s)     0.00330       1.13471
  23    C  2  s      Ryd( 4s)     0.00005       4.39068
  24    C  2  px     Val( 2p)     1.10896      -0.08215
  25    C  2  px     Ryd( 3p)     0.00370       0.66068
  26    C  2  py     Val( 2p)     1.13266      -0.06690
  27    C  2  py     Ryd( 3p)     0.00735       0.80561
  28    C  2  pz     Val( 2p)     1.16114      -0.13504
  29    C  2  pz     Ryd( 3p)     0.00735       0.73206
  30    C  2  dxy    Ryd( 3d)     0.00153       2.26043
  31    C  2  dxz    Ryd( 3d)     0.00114       1.89812
  32    C  2  dyz    Ryd( 3d)     0.00211       2.06721
  33    C  2  dx2y2  Ryd( 3d)     0.00147       2.28583
  34    C  2  dz2    Ryd( 3d)     0.00116       2.09654

  35    C  3  s      Cor( 1s)     1.99999     -10.21276
  36    C  3  s      Val( 2s)     1.01977      -0.19466
  37    C  3  s      Ryd( 3s)     0.00330       1.13471
  38    C  3  s      Ryd( 4s)     0.00005       4.39068
  39    C  3  px     Val( 2p)     1.10896      -0.08215
  40    C  3  px     Ryd( 3p)     0.00370       0.66068
  41    C  3  py     Val( 2p)     1.13266      -0.06690
  42    C  3  py     Ryd( 3p)     0.00735       0.80561
  43    C  3  pz     Val( 2p)     1.16114      -0.13504
  44    C  3  pz     Ryd( 3p)     0.00735       0.73206
  45    C  3  dxy    Ryd( 3d)     0.00153       2.26043
```

46	C	3	dxz	Ryd(3d)	0.00114	1.89812
47	C	3	dyz	Ryd(3d)	0.00211	2.06721
48	C	3	dx2y2	Ryd(3d)	0.00147	2.28583
49	C	3	dz2	Ryd(3d)	0.00116	2.09654
50	C	4	s	Cor(1s)	1.99999	-10.21276
51	C	4	s	Val(2s)	1.01977	-0.19466
52	C	4	s	Ryd(3s)	0.00330	1.13471
53	C	4	s	Ryd(4s)	0.00005	4.39068
54	C	4	px	Val(2p)	1.14452	-0.05928
55	C	4	px	Ryd(3p)	0.00918	0.87807
56	C	4	py	Val(2p)	1.09711	-0.08977
57	C	4	py	Ryd(3p)	0.00187	0.58822
58	C	4	pz	Val(2p)	1.16114	-0.13504
59	C	4	pz	Ryd(3p)	0.00735	0.73206
60	C	4	dxy	Ryd(3d)	0.00143	2.29853
61	C	4	dxz	Ryd(3d)	0.00259	2.15176
62	C	4	dyz	Ryd(3d)	0.00066	1.81358
63	C	4	dx2y2	Ryd(3d)	0.00156	2.24773
64	C	4	dz2	Ryd(3d)	0.00116	2.09654
65	H	5	s	Val(1s)	0.72575	0.12620
66	H	5	s	Ryd(2s)	0.00052	0.53779
67	H	5	px	Ryd(2p)	0.00016	2.50597
68	H	5	py	Ryd(2p)	0.00026	2.83354
69	H	5	pz	Ryd(2p)	0.00017	2.50376
70	H	6	s	Val(1s)	0.72575	0.12620
71	H	6	s	Ryd(2s)	0.00052	0.53779
72	H	6	px	Ryd(2p)	0.00016	2.50597
73	H	6	py	Ryd(2p)	0.00026	2.83354
74	H	6	pz	Ryd(2p)	0.00017	2.50376
75	H	7	s	Val(1s)	0.72575	0.12620
76	H	7	s	Ryd(2s)	0.00052	0.53779
77	H	7	px	Ryd(2p)	0.00031	2.99732
78	H	7	py	Ryd(2p)	0.00011	2.34218
79	H	7	pz	Ryd(2p)	0.00017	2.50376

Summary of Natural Population Analysis:

	Natural	Natural Population			
Atom No	Charge	Core	Valence	Rydberg	Total
P 1	0.53559	9.99995	4.43022	0.03424	14.46441
C 2	-0.45167	1.99999	4.42253	0.02914	6.45167
C 3	-0.45167	1.99999	4.42253	0.02914	6.45167
C 4	-0.45167	1.99999	4.42253	0.02914	6.45167
H 5	0.27314	0.00000	0.72575	0.00111	0.72686
H 6	0.27314	0.00000	0.72575	0.00111	0.72686
H 7	0.27314	0.00000	0.72575	0.00111	0.72686
* Total *	0.00000	15.99993	19.87507	0.12500	36.00000

	Natural Population
Core	15.99993 (99.9995% of 16)
Valence	19.87507 (99.3754% of 20)
Natural Minimal Basis	35.87500 (99.6528% of 36)
Natural Rydberg Basis	0.12500 (0.3472% of 36)

```
 ----------------------------------------------------------

   Atom No          Natural Electron Configuration
 ----------------------------------------------------------------------------
      P  1      [core]3s( 1.72)3p( 2.71)3d( 0.03)
      C  2      [core]2s( 1.02)2p( 3.40)3p( 0.02)3d( 0.01)
      C  3      [core]2s( 1.02)2p( 3.40)3p( 0.02)3d( 0.01)
      C  4      [core]2s( 1.02)2p( 3.40)3p( 0.02)3d( 0.01)
      H  5            1s( 0.73)
      H  6            1s( 0.73)
      H  7            1s( 0.73)


 NATURAL BOND ORBITAL ANALYSIS:

                           Occupancies       Lewis Structure    Low   High
          Max    Occ    -------------------  -----------------   occ   occ
  Cycle   Ctr   Thresh    Lewis   non-Lewis     CR  BD  nC  LP    (L)   (NL)
 ============================================================================
    1     2     1.90    35.73365   0.26635      8   9   0   1     0      0
 ----------------------------------------------------------------------------

 Structure accepted: No low occupancy Lewis orbitals

 --------------------------------------------------------
   Core                      15.99993 (100.000% of  16)
   Valence Lewis             19.73373 ( 98.669% of  20)
  ==================       =============================
   Total Lewis               35.73365 ( 99.260% of  36)
  -----------------------------------------------------
   Valence non-Lewis          0.22020 (  0.612% of  36)
   Rydberg non-Lewis          0.04614 (  0.128% of  36)
  ==================       =============================
   Total non-Lewis            0.26635 (  0.740% of  36)
 --------------------------------------------------------


     (Occupancy)   Bond orbital / Coefficients / Hybrids
 ------------------ Lewis ------------------------------------------------------
   1. (2.00000) CR ( 1) P  1           s(100.00%)
                                         1.0000  0.0000  0.0000  0.0000  0.0000
                                         0.0000  0.0000  0.0000  0.0000  0.0000
                                         0.0000  0.0000  0.0000  0.0000  0.0000
                                         0.0000  0.0000  0.0000  0.0000
   2. (1.99999) CR ( 2) P  1           s(100.00%)
                                         0.0000  1.0000  0.0000  0.0000  0.0000
                                         0.0000  0.0000  0.0000  0.0000  0.0000
                                         0.0000  0.0000  0.0000  0.0000  0.0000
                                         0.0000  0.0000  0.0000  0.0000
   3. (1.99999) CR ( 3) P  1           s(  0.00%)p 1.00(100.00%)
                                         0.0000  0.0000  0.0000  0.0000  0.0000
                                         1.0000  0.0000  0.0000  0.0000  0.0000
                                         0.0000  0.0000  0.0000  0.0000  0.0000
                                         0.0000  0.0000  0.0000  0.0000
   4. (1.99999) CR ( 4) P  1           s(  0.00%)p 1.00(100.00%)
                                         0.0000  0.0000  0.0000  0.0000  0.0000
                                         0.0000  0.0000  0.0000  1.0000  0.0000
                                         0.0000  0.0000  0.0000  0.0000  0.0000
                                         0.0000  0.0000  0.0000  0.0000
   5. (1.99998) CR ( 5) P  1           s(  0.00%)p 1.00(100.00%)
                                         0.0000  0.0000  0.0000  0.0000  0.0000
                                         0.0000  0.0000  0.0000  0.0000  0.0000
```

```
                                        0.0000  1.0000  0.0000  0.0000  0.0000
                                        0.0000  0.0000  0.0000  0.0000
  6. (1.99999) CR ( 1) C  2           s(100.00%)
                                        1.0000  0.0000  0.0000  0.0000  0.0000
                                        0.0000  0.0000  0.0000  0.0000  0.0000
                                        0.0000  0.0000  0.0000  0.0000  0.0000
  7. (1.99999) CR ( 1) C  3           s(100.00%)
                                        1.0000  0.0000  0.0000  0.0000  0.0000
                                        0.0000  0.0000  0.0000  0.0000  0.0000
                                        0.0000  0.0000  0.0000  0.0000  0.0000
  8. (1.99999) CR ( 1) C  4           s(100.00%)
                                        1.0000  0.0000  0.0000  0.0000  0.0000
                                        0.0000  0.0000  0.0000  0.0000  0.0000
                                        0.0000  0.0000  0.0000  0.0000  0.0000
  9. (1.99860) LP ( 1) P  1           s( 81.35%)p 0.23( 18.62%)d 0.00(  0.02%)
                                        0.0000  0.0000  0.9019  0.0106  0.0001
                                        0.0000  0.0000  0.0000  0.0000  0.0000
                                        0.0000  0.0000  0.4315 -0.0057  0.0000
                                        0.0000  0.0000  0.0000 -0.0150
 10. (1.95481) BD ( 1) P  1- C  2
             ( 40.97%)   0.6401* P  1 s(  6.39%)p14.54( 92.90%)d 0.11(  0.70%)
                                        0.0000  0.0000  0.2483 -0.0474 -0.0007
                                        0.0000 -0.4069  0.0012  0.0000  0.7047
                                       -0.0022  0.0000 -0.5163 -0.0164 -0.0231
                                        0.0306 -0.0529 -0.0133  0.0510
             ( 59.03%)   0.7683* C  2 s( 13.26%)p 6.53( 86.61%)d 0.01(  0.13%)
                                        0.0000  0.3620  0.0391  0.0004 -0.0591
                                       -0.0325  0.1024  0.0564  0.9197 -0.0444
                                       -0.0048  0.0134 -0.0233 -0.0028  0.0241
 11. (1.95481) BD ( 1) P  1- C  3
             ( 40.97%)   0.6401* P  1 s(  6.39%)p14.54( 92.90%)d 0.11(  0.70%)
                                        0.0000  0.0000  0.2483 -0.0474 -0.0007
                                        0.0000 -0.4069  0.0012  0.0000 -0.7047
                                        0.0022  0.0000 -0.5163 -0.0164  0.0231
                                        0.0306  0.0529 -0.0133  0.0510
             ( 59.03%)   0.7683* C  3 s( 13.26%)p 6.53( 86.61%)d 0.01(  0.13%)
                                        0.0000  0.3620  0.0391  0.0004 -0.0591
                                       -0.0325 -0.1024 -0.0564  0.9197 -0.0444
                                        0.0048  0.0134  0.0233 -0.0028  0.0241
 12. (1.95481) BD ( 1) P  1- C  4
             ( 40.97%)   0.6401* P  1 s(  6.39%)p14.54( 92.90%)d 0.11(  0.70%)
                                        0.0000  0.0000  0.2483 -0.0474 -0.0007
                                        0.0000  0.8138 -0.0025  0.0000  0.0000
                                        0.0000  0.0000 -0.5163 -0.0164  0.0000
                                       -0.0611  0.0000  0.0266  0.0510
             ( 59.03%)   0.7683* C  4 s( 13.26%)p 6.53( 86.61%)d 0.01(  0.13%)
                                        0.0000  0.3620  0.0391  0.0004  0.1183
                                        0.0651  0.0000  0.0000  0.9197 -0.0444
                                        0.0000 -0.0268  0.0000  0.0056  0.0241
 13. (1.96259) BD ( 1) C  2- C  3
             ( 50.00%)   0.7071* C  2 s( 22.99%)p 3.35( 76.90%)d 0.00(  0.11%)
                                        0.0000  0.4794  0.0037 -0.0025 -0.3611
                                       -0.0405 -0.7868  0.0168 -0.1283 -0.0329
                                       -0.0008 -0.0002 -0.0049 -0.0299 -0.0149
             ( 50.00%)   0.7071* C  3 s( 22.99%)p 3.35( 76.90%)d 0.00(  0.11%)
                                        0.0000  0.4794  0.0037 -0.0025 -0.3611
                                       -0.0405  0.7868 -0.0168 -0.1283 -0.0329
                                        0.0008 -0.0002  0.0049 -0.0299 -0.0149
 14. (1.96259) BD ( 1) C  2- C  4
             ( 50.00%)   0.7071* C  2 s( 22.99%)p 3.35( 76.90%)d 0.00(  0.11%)
                                        0.0000  0.4794  0.0037 -0.0025  0.8620
                                        0.0057 -0.0807  0.0435 -0.1283 -0.0329
```

```
                                         -0.0263  0.0043 -0.0023  0.0143 -0.0149
               ( 50.00%)   0.7071* C  4 s( 22.99%)p 3.35( 76.90%)d 0.00(  0.11%)
                                          0.0000  0.4794  0.0037 -0.0025 -0.5009
                                          0.0348  0.7061  0.0267 -0.1283 -0.0329
                                         -0.0255 -0.0042  0.0026  0.0156 -0.0149
 15. (1.99431) BD ( 1) C  2- H  5
               ( 64.58%)   0.8036* C  2 s( 40.24%)p 1.48( 59.72%)d 0.00(  0.04%)
                                          0.0000  0.6340 -0.0213  0.0021 -0.3473
                                         -0.0037  0.6015  0.0065 -0.3387  0.0027
                                         -0.0127  0.0072 -0.0125 -0.0074 -0.0042
               ( 35.42%)   0.5951* H  5 s( 99.95%)p 0.00(  0.05%)
                                          0.9997  0.0043  0.0101 -0.0175  0.0097
 16. (1.96259) BD ( 1) C  3- C  4
               ( 50.00%)   0.7071* C  3 s( 22.99%)p 3.35( 76.90%)d 0.00(  0.11%)
                                          0.0000  0.4794  0.0037 -0.0025  0.8620
                                          0.0057  0.0807 -0.0435 -0.1283 -0.0329
                                          0.0263  0.0043  0.0023  0.0143 -0.0149
               ( 50.00%)   0.7071* C  4 s( 22.99%)p 3.35( 76.90%)d 0.00(  0.11%)
                                          0.0000  0.4794  0.0037 -0.0025 -0.5009
                                          0.0348 -0.7061 -0.0267 -0.1283 -0.0329
                                          0.0255 -0.0042 -0.0026  0.0156 -0.0149
 17. (1.99431) BD ( 1) C  3- H  6
               ( 64.58%)   0.8036* C  3 s( 40.24%)p 1.48( 59.72%)d 0.00(  0.04%)
                                          0.0000  0.6340 -0.0213  0.0021 -0.3473
                                         -0.0037 -0.6015 -0.0065 -0.3387  0.0027
                                          0.0127  0.0072  0.0125 -0.0074 -0.0042
               ( 35.42%)   0.5951* H  6 s( 99.95%)p 0.00(  0.05%)
                                          0.9997  0.0043  0.0101  0.0175  0.0097
 18. (1.99431) BD ( 1) C  4- H  7
               ( 64.58%)   0.8036* C  4 s( 40.24%)p 1.48( 59.72%)d 0.00(  0.04%)
                                          0.0000  0.6340 -0.0213  0.0021  0.6945
                                          0.0075  0.0000  0.0000 -0.3387  0.0027
                                          0.0000 -0.0145  0.0000  0.0147 -0.0042
               ( 35.42%)   0.5951* H  7 s( 99.95%)p 0.00(  0.05%)
                                          0.9997  0.0043 -0.0202  0.0000  0.0097
---------------- non-Lewis ----------------------------------------------------
 19. (0.02849) BD*( 1) P  1- C  2
               ( 59.03%)   0.7683* P  1 s(  6.39%)p14.54( 92.90%)d 0.11(  0.70%)
                                          0.0000  0.0000 -0.2483  0.0474  0.0007
                                          0.0000  0.4069 -0.0012  0.0000 -0.7047
                                          0.0022  0.0000  0.5163  0.0164  0.0231
                                         -0.0306  0.0529  0.0133 -0.0510
               ( 40.97%)  -0.6401* C  2 s( 13.26%)p 6.53( 86.61%)d 0.01(  0.13%)
                                          0.0000 -0.3620 -0.0391 -0.0004  0.0591
                                          0.0325 -0.1024 -0.0564 -0.9197  0.0444
                                          0.0048 -0.0134  0.0233  0.0028 -0.0241
 20. (0.02849) BD*( 1) P  1- C  3
               ( 59.03%)   0.7683* P  1 s(  6.39%)p14.54( 92.90%)d 0.11(  0.70%)
                                          0.0000  0.0000 -0.2483  0.0474  0.0007
                                          0.0000  0.4069 -0.0012  0.0000  0.7047
                                         -0.0022  0.0000  0.5163  0.0164 -0.0231
                                         -0.0306 -0.0529  0.0133 -0.0510
               ( 40.97%)  -0.6401* C  3 s( 13.26%)p 6.53( 86.61%)d 0.01(  0.13%)
                                          0.0000 -0.3620 -0.0391 -0.0004  0.0591
                                          0.0325  0.1024  0.0564 -0.9197  0.0444
                                         -0.0048 -0.0134 -0.0233  0.0028 -0.0241
 21. (0.02849) BD*( 1) P  1- C  4
               ( 59.03%)   0.7683* P  1 s(  6.39%)p14.54( 92.90%)d 0.11(  0.70%)
                                          0.0000  0.0000 -0.2483  0.0474  0.0007
                                          0.0000 -0.8138  0.0025  0.0000  0.0000
                                          0.0000  0.0000  0.5163  0.0164  0.0000
                                          0.0611  0.0000 -0.0266 -0.0510
```

```
             ( 40.97%)  -0.6401* C  4 s( 13.26%)p 6.53( 86.61%)d 0.01(  0.13%)
                                        0.0000 -0.3620 -0.0391 -0.0004 -0.1183
                                       -0.0651  0.0000  0.0000 -0.9197  0.0444
                                        0.0000  0.0268  0.0000 -0.0056 -0.0241
22. (0.01437) BD*( 1) C  2- C  3
             ( 50.00%)   0.7071* C  2 s( 22.99%)p 3.35( 76.90%)d 0.00(  0.11%)
                                        0.0000  0.4794  0.0037 -0.0025 -0.3611
                                       -0.0405 -0.7868  0.0168 -0.1283 -0.0329
                                       -0.0008 -0.0002 -0.0049 -0.0299 -0.0149
             ( 50.00%)  -0.7071* C  3 s( 22.99%)p 3.35( 76.90%)d 0.00(  0.11%)
                                        0.0000  0.4794  0.0037 -0.0025 -0.3611
                                       -0.0405  0.7868 -0.0168 -0.1283 -0.0329
                                        0.0008 -0.0002  0.0049 -0.0299 -0.0149
23. (0.01437) BD*( 1) C  2- C  4
             ( 50.00%)   0.7071* C  2 s( 22.99%)p 3.35( 76.90%)d 0.00(  0.11%)
                                        0.0000  0.4794  0.0037 -0.0025  0.8620
                                        0.0057 -0.0807  0.0435 -0.1283 -0.0329
                                       -0.0263  0.0043 -0.0023  0.0143 -0.0149
             ( 50.00%)  -0.7071* C  4 s( 22.99%)p 3.35( 76.90%)d 0.00(  0.11%)
                                        0.0000  0.4794  0.0037 -0.0025 -0.5009
                                        0.0348  0.7061  0.0267 -0.1283 -0.0329
                                       -0.0255 -0.0042  0.0026  0.0156 -0.0149
24. (0.03054) BD*( 1) C  2- H  5
             ( 35.42%)   0.5951* C  2 s( 40.24%)p 1.48( 59.72%)d 0.00(  0.04%)
                                        0.0000 -0.6340  0.0213 -0.0021  0.3473
                                        0.0037 -0.6015 -0.0065  0.3387 -0.0027
                                        0.0127 -0.0072  0.0125  0.0074  0.0042
             ( 64.58%)  -0.8036* H  5 s( 99.95%)p 0.00(  0.05%)
                                       -0.9997 -0.0043 -0.0101  0.0175 -0.0097
25. (0.01437) BD*( 1) C  3- C  4
             ( 50.00%)   0.7071* C  3 s( 22.99%)p 3.35( 76.90%)d 0.00(  0.11%)
                                        0.0000  0.4794  0.0037 -0.0025  0.8620
                                        0.0057  0.0807 -0.0435 -0.1283 -0.0329
                                        0.0263  0.0043  0.0023  0.0143 -0.0149
             ( 50.00%)  -0.7071* C  4 s( 22.99%)p 3.35( 76.90%)d 0.00(  0.11%)
                                        0.0000  0.4794  0.0037 -0.0025 -0.5009
                                        0.0348 -0.7061 -0.0267 -0.1283 -0.0329
                                        0.0255 -0.0042 -0.0026  0.0156 -0.0149
26. (0.03054) BD*( 1) C  3- H  6
             ( 35.42%)   0.5951* C  3 s( 40.24%)p 1.48( 59.72%)d 0.00(  0.04%)
                                        0.0000 -0.6340  0.0213 -0.0021  0.3473
                                        0.0037  0.6015  0.0065  0.3387 -0.0027
                                       -0.0127 -0.0072 -0.0125  0.0074  0.0042
             ( 64.58%)  -0.8036* H  6 s( 99.95%)p 0.00(  0.05%)
                                       -0.9997 -0.0043 -0.0101 -0.0175 -0.0097
27. (0.03054) BD*( 1) C  4- H  7
             ( 35.42%)   0.5951* C  4 s( 40.24%)p 1.48( 59.72%)d 0.00(  0.04%)
                                        0.0000 -0.6340  0.0213 -0.0021 -0.6945
                                       -0.0075  0.0000  0.0000  0.3387 -0.0027
                                        0.0000  0.0145  0.0000 -0.0147  0.0042
             ( 64.58%)  -0.8036* H  7 s( 99.95%)p 0.00(  0.05%)
                                       -0.9997 -0.0043  0.0202  0.0000 -0.0097
28. (0.00471) RY ( 1) P  1            s(  0.00%)p 1.00( 36.37%)d 1.75( 63.63%)
                                        0.0000  0.0000  0.0000  0.0000  0.0000
                                        0.0000  0.0110 -0.2289  0.0000  0.0268
                                       -0.5571  0.0000  0.0000  0.0000 -0.4575
                                        0.2379  0.5789  0.1880  0.0000
29. (0.00471) RY ( 2) P  1            s(  0.00%)p 1.00( 36.37%)d 1.75( 63.63%)
                                        0.0000  0.0000  0.0000  0.0000  0.0000
                                        0.0000  0.0268 -0.5571  0.0000 -0.0110
                                        0.2289  0.0000  0.0000  0.0000  0.1880
                                        0.5789 -0.2379  0.4575  0.0000
```

```
30. (0.00264) RY ( 3) P  1           s( 12.26%)p 1.82( 22.36%)d 5.33( 65.38%)
                                      0.0000  0.0000  0.0371  0.3452 -0.0450
                                      0.0000  0.0000  0.0000  0.0000  0.0000
                                      0.0000  0.0000 -0.1082  0.4603  0.0000
                                      0.0000  0.0000  0.0000 -0.8086
31. (0.00019) RY ( 4) P  1           s(  0.00%)p 1.00( 27.38%)d 2.65( 72.62%)
                                      0.0000  0.0000  0.0000  0.0000  0.0000
                                      0.0000 -0.0377  0.5219  0.0000  0.0000
                                      0.0000  0.0000  0.0000  0.0000  0.0000
                                     -0.1578  0.0000  0.8374  0.0000
32. (0.00019) RY ( 5) P  1           s(  0.00%)p 1.00( 27.38%)d 2.65( 72.62%)
                                      0.0000  0.0000  0.0000  0.0000  0.0000
                                      0.0000  0.0000  0.0000  0.0000  0.0377
                                     -0.5219  0.0000  0.0000  0.0000  0.8374
                                      0.0000  0.1578  0.0000  0.0000
33. (0.00003) RY ( 6) P  1           s(  1.82%)p41.84( 76.24%)d12.04( 21.94%)
34. (0.00000) RY ( 7) P  1           s( 87.21%)p 0.01(  0.96%)d 0.14( 11.83%)
35. (0.00000) RY ( 8) P  1           s(  0.00%)p 1.00( 36.92%)d 1.71( 63.08%)
36. (0.00000) RY ( 9) P  1           s(  0.00%)p 1.00( 36.92%)d 1.71( 63.08%)
37. (0.00000) RY (10) P  1           s( 98.18%)p 0.02(  1.77%)d 0.00(  0.05%)
38. (0.00740) RY ( 1) C  2           s( 12.47%)p 5.62( 70.09%)d 1.40( 17.45%)
                                      0.0000 -0.0040  0.3531 -0.0050 -0.0103
                                      0.1947  0.0179 -0.3372 -0.0164 -0.7407
                                     -0.0759 -0.1961  0.3397 -0.0438  0.1136
39. (0.00172) RY ( 2) C  2           s( 29.94%)p 0.90( 27.07%)d 1.44( 42.99%)
                                      0.0000 -0.0153  0.5391  0.0923 -0.0086
                                     -0.2587  0.0149  0.4480 -0.0491 -0.0173
                                      0.5558 -0.0671  0.1163  0.3209  0.0092
40. (0.00088) RY ( 3) C  2           s(  0.00%)p 1.00( 28.04%)d 2.57( 71.96%)
                                      0.0000  0.0000  0.0000  0.0000 -0.0188
                                      0.4582 -0.0109  0.2645  0.0000  0.0000
                                      0.0191  0.7339  0.4237 -0.0331  0.0000
41. (0.00036) RY ( 4) C  2           s( 40.37%)p 1.20( 48.60%)d 0.27( 11.03%)
                                      0.0000  0.0446  0.6085 -0.1776  0.0078
                                      0.2826 -0.0136 -0.4895  0.0150  0.4075
                                      0.0386  0.1396 -0.2417  0.0223 -0.1743
42. (0.00007) RY ( 5) C  2           s(  2.88%)p 6.78( 19.52%)d26.94( 77.60%)
43. (0.00004) RY ( 6) C  2           s(  0.00%)p 1.00( 70.06%)d 0.43( 29.94%)
44. (0.00000) RY ( 7) C  2           s( 28.45%)p 0.72( 20.61%)d 1.79( 50.94%)
45. (0.00000) RY ( 8) C  2           s( 55.97%)p 0.08(  4.20%)d 0.71( 39.83%)
46. (0.00000) RY ( 9) C  2           s( 30.44%)p 0.32(  9.66%)d 1.97( 59.90%)
47. (0.00000) RY (10) C  2           s(  0.00%)p 1.00(  2.03%)d48.29( 97.97%)
48. (0.00740) RY ( 1) C  3           s( 12.47%)p 5.62( 70.09%)d 1.40( 17.45%)
                                      0.0000 -0.0040  0.3531 -0.0050 -0.0103
                                      0.1947 -0.0179  0.3372 -0.0164 -0.7407
                                      0.0759 -0.1961 -0.3397 -0.0438  0.1136
49. (0.00172) RY ( 2) C  3           s( 29.94%)p 0.90( 27.07%)d 1.44( 42.99%)
                                      0.0000 -0.0153  0.5391  0.0923 -0.0086
                                     -0.2587 -0.0149 -0.4480 -0.0491 -0.0173
                                     -0.5558 -0.0671 -0.1163  0.3209  0.0092
50. (0.00088) RY ( 3) C  3           s(  0.00%)p 1.00( 28.04%)d 2.57( 71.96%)
                                      0.0000  0.0000  0.0000  0.0000 -0.0188
                                      0.4582  0.0109 -0.2645  0.0000  0.0000
                                     -0.0191  0.7339 -0.4237 -0.0331  0.0000
51. (0.00036) RY ( 4) C  3           s( 40.37%)p 1.20( 48.60%)d 0.27( 11.03%)
                                      0.0000  0.0446  0.6085 -0.1776  0.0078
                                      0.2826  0.0136  0.4895  0.0150  0.4075
                                     -0.0386  0.1396  0.2417  0.0223 -0.1743
52. (0.00007) RY ( 5) C  3           s(  2.88%)p 6.78( 19.52%)d26.94( 77.60%)
53. (0.00004) RY ( 6) C  3           s(  0.00%)p 1.00( 70.06%)d 0.43( 29.94%)
54. (0.00000) RY ( 7) C  3           s( 28.45%)p 0.72( 20.61%)d 1.79( 50.94%)
55. (0.00000) RY ( 8) C  3           s( 55.97%)p 0.08(  4.20%)d 0.71( 39.83%)
```

```
 56. (0.00000) RY ( 9) C  3           s( 30.44%)p 0.32(  9.66%)d 1.97( 59.90%)
 57. (0.00000) RY (10) C  3           s(  0.00%)p 1.00(  2.03%)d48.29( 97.97%)
 58. (0.00740) RY ( 1) C  4           s( 12.47%)p 5.62( 70.09%)d 1.40( 17.45%)
                                       0.0000 -0.0040  0.3531 -0.0050  0.0206
                                      -0.3893  0.0000  0.0000 -0.0164 -0.7407
                                       0.0000  0.3923  0.0000  0.0876  0.1136
 59. (0.00172) RY ( 2) C  4           s( 29.94%)p 0.90( 27.07%)d 1.44( 42.99%)
                                       0.0000 -0.0153  0.5391  0.0923  0.0172
                                       0.5173  0.0000  0.0000 -0.0491 -0.0173
                                       0.0000  0.1343  0.0000 -0.6417  0.0092
 60. (0.00088) RY ( 3) C  4           s(  0.00%)p 1.00( 28.04%)d 2.57( 71.96%)
                                       0.0000  0.0000  0.0000  0.0000  0.0000
                                       0.0000 -0.0217  0.5291  0.0000  0.0000
                                       0.0382  0.0000  0.8474  0.0000  0.0000
 61. (0.00036) RY ( 4) C  4           s( 40.37%)p 1.20( 48.60%)d 0.27( 11.03%)
                                       0.0000  0.0446  0.6085 -0.1776 -0.0157
                                      -0.5652  0.0000  0.0000  0.0150  0.4075
                                       0.0000 -0.2791  0.0000 -0.0445 -0.1743
 62. (0.00007) RY ( 5) C  4           s(  2.88%)p 6.78( 19.52%)d26.94( 77.60%)
 63. (0.00004) RY ( 6) C  4           s(  0.00%)p 1.00( 70.06%)d 0.43( 29.94%)
 64. (0.00000) RY ( 7) C  4           s( 28.45%)p 0.72( 20.61%)d 1.79( 50.94%)
 65. (0.00000) RY ( 8) C  4           s( 30.44%)p 0.32(  9.66%)d 1.97( 59.90%)
 66. (0.00000) RY ( 9) C  4           s( 55.97%)p 0.08(  4.20%)d 0.71( 39.83%)
 67. (0.00000) RY (10) C  4           s(  0.00%)p 1.00(  2.03%)d48.29( 97.97%)
 68. (0.00053) RY ( 1) H  5           s( 99.05%)p 0.01(  0.95%)
                                      -0.0036  0.9952  0.0083 -0.0143 -0.0962
 69. (0.00011) RY ( 2) H  5           s(  0.92%)p99.99( 99.08%)
                                      -0.0024  0.0959 -0.1747  0.3026  0.9321
 70. (0.00011) RY ( 3) H  5           s(  0.00%)p 1.00(100.00%)
                                       0.0000  0.0000  0.8660  0.5000  0.0000
 71. (0.00000) RY ( 4) H  5           s(  0.08%)p99.99( 99.92%)
 72. (0.00053) RY ( 1) H  6           s( 99.05%)p 0.01(  0.95%)
                                      -0.0036  0.9952  0.0083  0.0143 -0.0962
 73. (0.00011) RY ( 2) H  6           s(  0.92%)p99.99( 99.08%)
                                      -0.0024  0.0959 -0.1747 -0.3026  0.9321
 74. (0.00011) RY ( 3) H  6           s(  0.00%)p 1.00(100.00%)
                                       0.0000  0.0000  0.8660 -0.5000  0.0000
 75. (0.00000) RY ( 4) H  6           s(  0.08%)p99.99( 99.92%)
 76. (0.00053) RY ( 1) H  7           s( 99.05%)p 0.01(  0.95%)
                                      -0.0036  0.9952 -0.0166  0.0000 -0.0962
 77. (0.00011) RY ( 2) H  7           s(  0.92%)p99.99( 99.08%)
                                      -0.0024  0.0959  0.3494  0.0000  0.9321
 78. (0.00011) RY ( 3) H  7           s(  0.00%)p 1.00(100.00%)
                                       0.0000  0.0000  0.0000  1.0000  0.0000
 79. (0.00000) RY ( 4) H  7           s(  0.08%)p99.99( 99.92%)


NHO DIRECTIONALITY AND BOND BENDING (deviation from line of nuclear centers at
                                    the position of maximum hybrid amplitude)

        [Thresholds for printing:  angular deviation  >  1.0 degree]
                                   p- or d-character  > 25.0%
                                   orbital occupancy  >  0.10e

                       Line of Centers       Hybrid 1           Hybrid 2
                       ---------------  -------------------  ------------------
          NBO            Theta   Phi     Theta   Phi    Dev   Theta   Phi    Dev
===============================================================================
 10. BD ( 1) P  1- C  2  153.0  120.0   125.3  120.0  27.7     2.9  120.0  29.8
 11. BD ( 1) P  1- C  3  153.0  240.0   125.3  240.0  27.7     2.9  240.0  29.8
 12. BD ( 1) P  1- C  4  153.0    0.0   125.3    0.0  27.7     2.9    0.0  29.8
 13. BD ( 1) C  2- C  3   90.0  270.0    96.7  250.1  20.9    96.7  109.9  20.9
```

```
 14. BD ( 1) C  2- C  4   90.0  330.0    96.7  349.9  20.9    96.7  130.1  20.9
 16. BD ( 1) C  3- C  4   90.0   30.0    96.7   10.1  20.9    96.7  229.9  20.9


SECOND ORDER PERTURBATION THEORY ANALYSIS OF FOCK MATRIX IN NBO BASIS

    Threshold for printing:   0.50 kcal/mol
                                                          E(2) E(NL)-E(L) F(L,NL)
     Donor (L) NBO              Acceptor (NL) NBO      kcal/mol   a.u.    a.u.
===============================================================================

within unit  1
  10. BD ( 1) P  1- C  2      19. BD*( 1) P  1- C  2      0.57    0.47   0.015
  10. BD ( 1) P  1- C  2      20. BD*( 1) P  1- C  3      5.32    0.47   0.045
  10. BD ( 1) P  1- C  2      21. BD*( 1) P  1- C  4      5.32    0.47   0.045
  10. BD ( 1) P  1- C  2      22. BD*( 1) C  2- C  3      0.57    0.72   0.018
  10. BD ( 1) P  1- C  2      23. BD*( 1) C  2- C  4      0.57    0.72   0.018
  10. BD ( 1) P  1- C  2      26. BD*( 1) C  3- H  6      6.10    0.85   0.064
  10. BD ( 1) P  1- C  2      27. BD*( 1) C  4- H  7      6.10    0.85   0.064
  10. BD ( 1) P  1- C  2      30. RY ( 3) P  1            0.86    1.09   0.027
  10. BD ( 1) P  1- C  2      48. RY ( 1) C  3            2.44    1.23   0.049
  10. BD ( 1) P  1- C  2      50. RY ( 3) C  3            0.61    1.83   0.030
  10. BD ( 1) P  1- C  2      58. RY ( 1) C  4            2.44    1.23   0.049
  10. BD ( 1) P  1- C  2      60. RY ( 3) C  4            0.61    1.83   0.030
  11. BD ( 1) P  1- C  3      19. BD*( 1) P  1- C  2      5.32    0.47   0.045
  11. BD ( 1) P  1- C  3      20. BD*( 1) P  1- C  3      0.57    0.47   0.015
  11. BD ( 1) P  1- C  3      21. BD*( 1) P  1- C  4      5.32    0.47   0.045
  11. BD ( 1) P  1- C  3      22. BD*( 1) C  2- C  3      0.57    0.72   0.018
  11. BD ( 1) P  1- C  3      24. BD*( 1) C  2- H  5      6.10    0.85   0.064
  11. BD ( 1) P  1- C  3      25. BD*( 1) C  3- C  4      0.57    0.72   0.018
  11. BD ( 1) P  1- C  3      27. BD*( 1) C  4- H  7      6.10    0.85   0.064
  11. BD ( 1) P  1- C  3      30. RY ( 3) P  1            0.86    1.09   0.027
  11. BD ( 1) P  1- C  3      38. RY ( 1) C  2            2.44    1.23   0.049
  11. BD ( 1) P  1- C  3      40. RY ( 3) C  2            0.61    1.83   0.030
  11. BD ( 1) P  1- C  3      58. RY ( 1) C  4            2.44    1.23   0.049
  11. BD ( 1) P  1- C  3      60. RY ( 3) C  4            0.61    1.83   0.030
  12. BD ( 1) P  1- C  4      19. BD*( 1) P  1- C  2      5.32    0.47   0.045
  12. BD ( 1) P  1- C  4      20. BD*( 1) P  1- C  3      5.32    0.47   0.045
  12. BD ( 1) P  1- C  4      21. BD*( 1) P  1- C  4      0.57    0.47   0.015
  12. BD ( 1) P  1- C  4      23. BD*( 1) C  2- C  4      0.57    0.72   0.018
  12. BD ( 1) P  1- C  4      24. BD*( 1) C  2- H  5      6.10    0.85   0.064
  12. BD ( 1) P  1- C  4      25. BD*( 1) C  3- C  4      0.57    0.72   0.018
  12. BD ( 1) P  1- C  4      26. BD*( 1) C  3- H  6      6.10    0.85   0.064
  12. BD ( 1) P  1- C  4      30. RY ( 3) P  1            0.86    1.09   0.027
  12. BD ( 1) P  1- C  4      38. RY ( 1) C  2            2.44    1.23   0.049
  12. BD ( 1) P  1- C  4      40. RY ( 3) C  2            0.61    1.83   0.030
  12. BD ( 1) P  1- C  4      48. RY ( 1) C  3            2.44    1.23   0.049
  12. BD ( 1) P  1- C  4      50. RY ( 3) C  3            0.61    1.83   0.030
  13. BD ( 1) C  2- C  3      19. BD*( 1) P  1- C  2      0.64    0.62   0.018
  13. BD ( 1) C  2- C  3      20. BD*( 1) P  1- C  3      0.64    0.62   0.018
  13. BD ( 1) C  2- C  3      21. BD*( 1) P  1- C  4      0.59    0.62   0.017
  13. BD ( 1) C  2- C  3      23. BD*( 1) C  2- C  4      4.45    0.87   0.055
  13. BD ( 1) C  2- C  3      24. BD*( 1) C  2- H  5      1.05    0.99   0.029
  13. BD ( 1) C  2- C  3      25. BD*( 1) C  3- C  4      4.45    0.87   0.055
  13. BD ( 1) C  2- C  3      26. BD*( 1) C  3- H  6      1.05    0.99   0.029
  13. BD ( 1) C  2- C  3      27. BD*( 1) C  4- H  7     12.02    0.99   0.098
  13. BD ( 1) C  2- C  3      29. RY ( 2) P  1            1.63    1.23   0.040
  13. BD ( 1) C  2- C  3      59. RY ( 2) C  4            0.76    2.07   0.035
  14. BD ( 1) C  2- C  4      19. BD*( 1) P  1- C  2      0.64    0.62   0.018
  14. BD ( 1) C  2- C  4      20. BD*( 1) P  1- C  3      0.59    0.62   0.017
  14. BD ( 1) C  2- C  4      21. BD*( 1) P  1- C  4      0.64    0.62   0.018
  14. BD ( 1) C  2- C  4      22. BD*( 1) C  2- C  3      4.45    0.87   0.055
```

```
  14. BD ( 1) C  2- C  4      24. BD*( 1) C  2- H  5      1.05    0.99   0.029
  14. BD ( 1) C  2- C  4      25. BD*( 1) C  3- C  4      4.45    0.87   0.055
  14. BD ( 1) C  2- C  4      26. BD*( 1) C  3- H  6     12.02    0.99   0.098
  14. BD ( 1) C  2- C  4      27. BD*( 1) C  4- H  7      1.05    0.99   0.029
  14. BD ( 1) C  2- C  4      28. RY ( 1) P  1            1.87    1.23   0.043
  14. BD ( 1) C  2- C  4      49. RY ( 2) C  3            0.76    2.07   0.035
  16. BD ( 1) C  3- C  4      19. BD*( 1) P  1- C  2      0.59    0.62   0.017
  16. BD ( 1) C  3- C  4      20. BD*( 1) P  1- C  3      0.64    0.62   0.018
  16. BD ( 1) C  3- C  4      21. BD*( 1) P  1- C  4      0.64    0.62   0.018
  16. BD ( 1) C  3- C  4      22. BD*( 1) C  2- C  3      4.45    0.87   0.055
  16. BD ( 1) C  3- C  4      23. BD*( 1) C  2- C  4      4.45    0.87   0.055
  16. BD ( 1) C  3- C  4      24. BD*( 1) C  2- H  5     12.02    0.99   0.098
  16. BD ( 1) C  3- C  4      26. BD*( 1) C  3- H  6      1.05    0.99   0.029
  16. BD ( 1) C  3- C  4      27. BD*( 1) C  4- H  7      1.05    0.99   0.029
  16. BD ( 1) C  3- C  4      28. RY ( 1) P  1            0.71    1.23   0.026
  16. BD ( 1) C  3- C  4      29. RY ( 2) P  1            1.20    1.23   0.034
  16. BD ( 1) C  3- C  4      39. RY ( 2) C  2            0.76    2.07   0.035


NATURAL BOND ORBITALS (Summary):

                                                     Principal Delocalizations
          NBO                 Occupancy    Energy   (geminal,vicinal,remote)
===============================================================================
Molecular unit  1  (C3H3P)
------ Lewis --------------------------------------
    1. CR ( 1) P  1           2.00000   -75.96290
    2. CR ( 2) P  1           1.99999    -7.66537
    3. CR ( 3) P  1           1.99999    -4.70809
    4. CR ( 4) P  1           1.99999    -4.70809
    5. CR ( 5) P  1           1.99998    -4.71152
    6. CR ( 1) C  2           1.99999   -10.21276
    7. CR ( 1) C  3           1.99999   -10.21276
    8. CR ( 1) C  4           1.99999   -10.21276
    9. LP ( 1) P  1           1.99860    -0.42042
   10. BD ( 1) P  1- C  2     1.95481    -0.35226  26(v),27(v),20(g),21(g)
                                                    48(v),58(v),30(g),50(v)
                                                    60(v),22(g),23(g),19(g)
   11. BD ( 1) P  1- C  3     1.95481    -0.35226  24(v),27(v),19(g),21(g)
                                                    38(v),58(v),30(g),40(v)
                                                    60(v),22(g),25(g),20(g)
   12. BD ( 1) P  1- C  4     1.95481    -0.35226  24(v),26(v),19(g),20(g)
                                                    38(v),48(v),30(g),40(v)
                                                    50(v),23(g),25(g),21(g)
   13. BD ( 1) C  2- C  3     1.96259    -0.50057  27(v),23(g),25(g),29(v)
                                                    24(g),26(g),59(v),19(g)
                                                    20(g),21(v)
   14. BD ( 1) C  2- C  4     1.96259    -0.50057  26(v),22(g),25(g),28(v)
                                                    24(g),27(g),49(v),19(g)
                                                    21(g),20(v)
   15. BD ( 1) C  2- H  5     1.99431    -0.52537
   16. BD ( 1) C  3- C  4     1.96259    -0.50057  24(v),22(g),23(g),29(v)
                                                    26(g),27(g),39(v),28(v)
                                                    20(g),21(g),19(v)
   17. BD ( 1) C  3- H  6     1.99431    -0.52537
   18. BD ( 1) C  4- H  7     1.99431    -0.52537
------ non-Lewis ----------------------------------
   19. BD*( 1) P  1- C  2     0.02849     0.12243
   20. BD*( 1) P  1- C  3     0.02849     0.12243
   21. BD*( 1) P  1- C  4     0.02849     0.12243
   22. BD*( 1) C  2- C  3     0.01437     0.36516
   23. BD*( 1) C  2- C  4     0.01437     0.36516
```

```
24. BD*( 1) C  2- H  5       0.03054     0.49337
25. BD*( 1) C  3- C  4       0.01437     0.36516
26. BD*( 1) C  3- H  6       0.03054     0.49337
27. BD*( 1) C  4- H  7       0.03054     0.49337
28. RY ( 1) P  1             0.00471     0.72572
29. RY ( 2) P  1             0.00471     0.72572
30. RY ( 3) P  1             0.00264     0.73533
31. RY ( 4) P  1             0.00019     0.69573
32. RY ( 5) P  1             0.00019     0.69573
33. RY ( 6) P  1             0.00003     0.59748
34. RY ( 7) P  1             0.00000     0.89001
35. RY ( 8) P  1             0.00000     0.81679
36. RY ( 9) P  1             0.00000     0.81679
37. RY (10) P  1             0.00000     3.15532
38. RY ( 1) C  2             0.00740     0.88093
39. RY ( 2) C  2             0.00172     1.57219
40. RY ( 3) C  2             0.00088     1.47476
41. RY ( 4) C  2             0.00036     1.05449
42. RY ( 5) C  2             0.00007     1.95603
43. RY ( 6) C  2             0.00004     0.94845
44. RY ( 7) C  2             0.00000     2.22649
45. RY ( 8) C  2             0.00000     3.37199
46. RY ( 9) C  2             0.00000     2.57623
47. RY (10) C  2             0.00000     2.26641
48. RY ( 1) C  3             0.00740     0.88093
49. RY ( 2) C  3             0.00172     1.57219
50. RY ( 3) C  3             0.00088     1.47476
51. RY ( 4) C  3             0.00036     1.05449
52. RY ( 5) C  3             0.00007     1.95603
53. RY ( 6) C  3             0.00004     0.94845
54. RY ( 7) C  3             0.00000     2.22649
55. RY ( 8) C  3             0.00000     3.37199
56. RY ( 9) C  3             0.00000     2.57623
57. RY (10) C  3             0.00000     2.26641
58. RY ( 1) C  4             0.00740     0.88093
59. RY ( 2) C  4             0.00172     1.57219
60. RY ( 3) C  4             0.00088     1.47476
61. RY ( 4) C  4             0.00036     1.05449
62. RY ( 5) C  4             0.00007     1.95603
63. RY ( 6) C  4             0.00004     0.94845
64. RY ( 7) C  4             0.00000     2.22649
65. RY ( 8) C  4             0.00000     2.57623
66. RY ( 9) C  4             0.00000     3.37199
67. RY (10) C  4             0.00000     2.26641
68. RY ( 1) H  5             0.00053     0.54568
69. RY ( 2) H  5             0.00011     2.32441
70. RY ( 3) H  5             0.00011     2.34218
71. RY ( 4) H  5             0.00000     3.15975
72. RY ( 1) H  6             0.00053     0.54568
73. RY ( 2) H  6             0.00011     2.32441
74. RY ( 3) H  6             0.00011     2.34218
75. RY ( 4) H  6             0.00000     3.15975
76. RY ( 1) H  7             0.00053     0.54568
77. RY ( 2) H  7             0.00011     2.32441
78. RY ( 3) H  7             0.00011     2.34218
79. RY ( 4) H  7             0.00000     3.15975
       -------------------------------
              Total Lewis   35.73365  ( 99.2602%)
        Valence non-Lewis    0.22020  (  0.6117%)
        Rydberg non-Lewis    0.04614  (  0.1282%)
       -------------------------------
            Total unit  1   36.00000  (100.0000%)
```

```
             Charge unit  1    0.00000

$CHOOSE
  LONE 1 1 END
  BOND S 1 2 S 1 3 S 1 4 S 2 3 S 2 4 S 2 5 S 3 4 S 3 6 S 4 7 END
$END


NATURAL RESONANCE THEORY ANALYSIS:

Maximum reference structures : 20
Maximum resonance structures : 5000
Memory requirements : 90192056 words of 149781109 available

16 candidate reference structure(s) calculated by SR LEWIS
Initial loops searched 16 bonding pattern(s); 1 was retained
Delocalization list threshold set to 1.20 kcal/mol for reference 1
Reference   1:  rho*=0.26635, f(w)=0.84874 converged after  13 iterations

                                              fractional accuracy f(w)
                 non-Lewis              -------------------------------------
 Ref     Wgt      density      d(0)       all NBOs     val+core     valence
----------------------------------------------------------------------------
  1    1.00000    0.26635    0.01445     0.84874      0.90418      0.90418


TOPO matrix for the leading resonance structure:

    Atom  1   2   3   4   5   6   7
    ---- --- --- --- --- --- --- ---
  1.  P   1   1   1   1   0   0   0
  2.  C   1   0   1   1   1   0   0
  3.  C   1   1   0   1   0   1   0
  4.  C   1   1   1   0   0   0   1
  5.  H   0   1   0   0   0   0   0
  6.  H   0   0   1   0   0   0   0
  7.  H   0   0   0   1   0   0   0

        Resonance
   RS   Weight(%)                  Added(Removed)
---------------------------------------------------------------------------
   1*     88.16
   2 (2)   1.52   ( P  1- C  2), ( P  1- C  3),  C  2- C  3,  P  1
   3 (2)   1.52   ( P  1- C  2), ( P  1- C  4),  C  2- C  4,  P  1
   4 (2)   1.52   ( P  1- C  3), ( P  1- C  4),  C  3- C  4,  P  1
   5 (2)   0.81   ( C  2- C  3), ( C  2- C  4),  C  3- C  4,  C  2
   6 (2)   0.81   ( C  2- C  3),  C  2- C  4, ( C  3- C  4),  C  3
   7 (2)   0.81    C  2- C  3, ( C  2- C  4), ( C  3- C  4),  C  4
   8       0.48   ( C  2- C  3),  C  2- C  4, ( C  4- H  7),  H  7
   9       0.48   ( C  2- C  3),  C  3- C  4, ( C  4- H  7),  H  7
  10       0.48    C  2- C  3, ( C  2- C  4), ( C  3- H  6),  H  6
  11       0.48   ( C  2- C  4),  C  3- C  4, ( C  3- H  6),  H  6
  12       0.48    C  2- C  3, ( C  2- H  5), ( C  3- C  4),  H  5
  13       0.48    C  2- C  4, ( C  2- H  5), ( C  3- C  4),  H  5
  14       0.16   ( P  1- C  2),  C  2- C  3, ( C  3- H  6),  H  6
  15       0.16   ( P  1- C  2),  C  2- C  4, ( C  4- H  7),  H  7
  16       0.16   ( P  1- C  3),  C  2- C  3, ( C  2- H  5),  H  5
  17       0.16   ( P  1- C  3),  C  3- C  4, ( C  4- H  7),  H  7
  18       0.16   ( P  1- C  4),  C  2- C  4, ( C  2- H  5),  H  5
  19       0.16   ( P  1- C  4),  C  3- C  4, ( C  3- H  6),  H  6
  20       0.16   ( P  1- C  2),  P  1- C  3, ( C  3- H  6),  H  6
  21       0.16   ( P  1- C  2),  P  1- C  4, ( C  4- H  7),  H  7
```

```
  22       0.16    P  1- C  2, ( P  1- C  3), ( C  2- H  5),  H  5
  23       0.16   ( P  1- C  3),  P  1- C  4, ( C  4- H  7),  H  7
  24       0.16    P  1- C  2, ( P  1- C  4), ( C  2- H  5),  H  5
  25       0.16    P  1- C  3, ( P  1- C  4), ( C  3- H  6),  H  6
-----------------------------------------------------------------------------
         100.00   * Total *                [* = reference structure]


Natural Bond Order:  (total/covalent/ionic)

    Atom       1      2      3      4      5      6      7
    ----    ------ ------ ------ ------ ------ ------ ------
  1.  P  t 1.0455 0.9664 0.9664 0.9664 0.0000 0.0000 0.0000
         c   ---  0.7893 0.7893 0.7893 0.0000 0.0000 0.0000
         i   ---  0.1771 0.1771 0.1771 0.0000 0.0000 0.0000

  2.  C  t 0.9664 0.0081 1.0103 1.0103 0.9838 0.0000 0.0000
         c 0.7893   ---  0.9743 0.9743 0.6969 0.0000 0.0000
         i 0.1771   ---  0.0360 0.0360 0.2869 0.0000 0.0000

  3.  C  t 0.9664 1.0103 0.0081 1.0103 0.0000 0.9838 0.0000
         c 0.7893 0.9743   ---  0.9743 0.0000 0.6969 0.0000
         i 0.1771 0.0360   ---  0.0360 0.0000 0.2869 0.0000

  4.  C  t 0.9664 1.0103 1.0103 0.0081 0.0000 0.0000 0.9838
         c 0.7893 0.9743 0.9743   ---  0.0000 0.0000 0.6969
         i 0.1771 0.0360 0.0360   ---  0.0000 0.0000 0.2869

  5.  H  t 0.0000 0.9838 0.0000 0.0000 0.0162 0.0000 0.0000
         c 0.0000 0.6969 0.0000 0.0000   ---  0.0000 0.0000
         i 0.0000 0.2869 0.0000 0.0000   ---  0.0000 0.0000

  6.  H  t 0.0000 0.0000 0.9838 0.0000 0.0000 0.0162 0.0000
         c 0.0000 0.0000 0.6969 0.0000 0.0000   ---  0.0000
         i 0.0000 0.0000 0.2869 0.0000 0.0000   ---  0.0000

  7.  H  t 0.0000 0.0000 0.0000 0.9838 0.0000 0.0000 0.0162
         c 0.0000 0.0000 0.0000 0.6969 0.0000 0.0000   ---
         i 0.0000 0.0000 0.0000 0.2869 0.0000 0.0000   ---


Natural Atomic Valencies:

                      Co-    Electro-
    Atom  Valency  Valency  Valency
    ----  -------  -------  -------
  1.  P    2.8992   2.3679   0.5313
  2.  C    3.9709   3.4349   0.5360
  3.  C    3.9709   3.4349   0.5360
  4.  C    3.9709   3.4349   0.5360
  5.  H    0.9838   0.6969   0.2869
  6.  H    0.9838   0.6969   0.2869
  7.  H    0.9838   0.6969   0.2869


$NRTSTR
  STR        ! Wgt = 88.16%
    LONE 1 1 END
    BOND S 1 2 S 1 3 S 1 4 S 2 3 S 2 4 S 2 5 S 3 4 S 3 6 S 4 7 END
  END
$END
```

```
NBO analysis completed in 0.19 CPU seconds (0 wall seconds)
Maximum scratch memory used by NBO was 90526287 words (690.66 MB)
..... done with NBO analysis .....
```

S3.6.3 White phosphorus

```
*********************************** NBO 6.0 ***********************************
            N A T U R A L   A T O M I C   O R B I T A L   A N D
         N A T U R A L   B O N D   O R B I T A L   A N A L Y S I S
**************** Massachusetts Institute of Technology (100648) ***************
 (c) Copyright 1996-2018 Board of Regents of the University of Wisconsin System
     on behalf of the Theoretical Chemistry Institute.  All rights reserved.

         Cite this program [NBO 6.0.18a (11-Mar-2018)] as:

         NBO 6.0.  E. D. Glendening, J. K. Badenhoop, A. E. Reed,
         J. E. Carpenter, J. A. Bohmann, C. M. Morales, C. R. Landis,
         and F. Weinhold (Theoretical Chemistry Institute, University
         of Wisconsin, Madison, WI, 2013); http://nbo6.chem.wisc.edu/

      /MEMORY / : Allocate 150000000 words of dynamic memory
      /NBO    / : Natural Bond Orbital Analysis
      /NRT    / : Natural Resonance Theory Analysis
      /MOLDEN / : Write NBOs to MOLDEN-formatted file

Job title:  white  phosphorus optimization


NATURAL POPULATIONS:  Natural atomic orbital occupancies

 NAO Atom No lang   Type(AO)    Occupancy      Energy
-------------------------------------------------------
   1    P  1  s      Cor( 1s)     2.00000     -75.55593
   2    P  1  s      Cor( 2s)     1.99999      -8.12344
   3    P  1  s      Val( 3s)     1.79268      -0.48857
   4    P  1  s      Ryd( 4s)     0.00680       0.53072
   5    P  1  s      Ryd( 5s)     0.00003       3.37973
   6    P  1  px     Cor( 2p)     1.99999      -4.73745
   7    P  1  px     Val( 3p)     1.05175      -0.17568
   8    P  1  px     Ryd( 4p)     0.00152       0.32520
   9    P  1  py     Cor( 2p)     1.99999      -4.73745
  10    P  1  py     Val( 3p)     1.05175      -0.17568
  11    P  1  py     Ryd( 4p)     0.00152       0.32520
  12    P  1  pz     Cor( 2p)     1.99999      -4.73745
  13    P  1  pz     Val( 3p)     1.05175      -0.17568
  14    P  1  pz     Ryd( 4p)     0.00152       0.32520
  15    P  1  dxy    Ryd( 3d)     0.00914       0.75774
  16    P  1  dxz    Ryd( 3d)     0.00914       0.75774
  17    P  1  dyz    Ryd( 3d)     0.00914       0.75774
  18    P  1  dx2y2  Ryd( 3d)     0.00666       0.71165
  19    P  1  dz2    Ryd( 3d)     0.00666       0.71165

  20    P  2  s      Cor( 1s)     2.00000     -75.55593
  21    P  2  s      Cor( 2s)     1.99999      -8.12344
  22    P  2  s      Val( 3s)     1.79268      -0.48857
  23    P  2  s      Ryd( 4s)     0.00680       0.53072
  24    P  2  s      Ryd( 5s)     0.00003       3.37973
  25    P  2  px     Cor( 2p)     1.99999      -4.73745
  26    P  2  px     Val( 3p)     1.05175      -0.17568
  27    P  2  px     Ryd( 4p)     0.00152       0.32520
```

28	P	2	py	Cor(2p)	1.99999	-4.73745
29	P	2	py	Val(3p)	1.05175	-0.17568
30	P	2	py	Ryd(4p)	0.00152	0.32520
31	P	2	pz	Cor(2p)	1.99999	-4.73745
32	P	2	pz	Val(3p)	1.05175	-0.17568
33	P	2	pz	Ryd(4p)	0.00152	0.32520
34	P	2	dxy	Ryd(3d)	0.00914	0.75774
35	P	2	dxz	Ryd(3d)	0.00914	0.75774
36	P	2	dyz	Ryd(3d)	0.00914	0.75774
37	P	2	dx2y2	Ryd(3d)	0.00666	0.71165
38	P	2	dz2	Ryd(3d)	0.00666	0.71165
39	P	3	s	Cor(1s)	2.00000	-75.55593
40	P	3	s	Cor(2s)	1.99999	-8.12344
41	P	3	s	Val(3s)	1.79268	-0.48857
42	P	3	s	Ryd(4s)	0.00680	0.53072
43	P	3	s	Ryd(5s)	0.00003	3.37973
44	P	3	px	Cor(2p)	1.99999	-4.73745
45	P	3	px	Val(3p)	1.05175	-0.17568
46	P	3	px	Ryd(4p)	0.00152	0.32520
47	P	3	py	Cor(2p)	1.99999	-4.73745
48	P	3	py	Val(3p)	1.05175	-0.17568
49	P	3	py	Ryd(4p)	0.00152	0.32520
50	P	3	pz	Cor(2p)	1.99999	-4.73745
51	P	3	pz	Val(3p)	1.05175	-0.17568
52	P	3	pz	Ryd(4p)	0.00152	0.32520
53	P	3	dxy	Ryd(3d)	0.00914	0.75774
54	P	3	dxz	Ryd(3d)	0.00914	0.75774
55	P	3	dyz	Ryd(3d)	0.00914	0.75774
56	P	3	dx2y2	Ryd(3d)	0.00666	0.71165
57	P	3	dz2	Ryd(3d)	0.00666	0.71165
58	P	4	s	Cor(1s)	2.00000	-75.55593
59	P	4	s	Cor(2s)	1.99999	-8.12344
60	P	4	s	Val(3s)	1.79268	-0.48857
61	P	4	s	Ryd(4s)	0.00680	0.53072
62	P	4	s	Ryd(5s)	0.00003	3.37973
63	P	4	px	Cor(2p)	1.99999	-4.73745
64	P	4	px	Val(3p)	1.05175	-0.17568
65	P	4	px	Ryd(4p)	0.00152	0.32520
66	P	4	py	Cor(2p)	1.99999	-4.73745
67	P	4	py	Val(3p)	1.05175	-0.17568
68	P	4	py	Ryd(4p)	0.00152	0.32520
69	P	4	pz	Cor(2p)	1.99999	-4.73745
70	P	4	pz	Val(3p)	1.05175	-0.17568
71	P	4	pz	Ryd(4p)	0.00152	0.32520
72	P	4	dxy	Ryd(3d)	0.00914	0.75774
73	P	4	dxz	Ryd(3d)	0.00914	0.75774
74	P	4	dyz	Ryd(3d)	0.00914	0.75774
75	P	4	dx2y2	Ryd(3d)	0.00666	0.71165
76	P	4	dz2	Ryd(3d)	0.00666	0.71165

Summary of Natural Population Analysis:

Atom No	Natural Charge	Natural Population Core	Valence	Rydberg	Total
P 1	0.00000	9.99996	4.94794	0.05211	15.00000
P 2	0.00000	9.99996	4.94794	0.05211	15.00000
P 3	0.00000	9.99996	4.94794	0.05211	15.00000

```
   P  4    0.00000      9.99996     4.94794    0.05211    15.00000
====================================================================
* Total *  0.00000     39.99982    19.79174    0.20844    60.00000

                                Natural Population
---------------------------------------------------------
  Core                      39.99982 ( 99.9996% of   40)
  Valence                   19.79174 ( 98.9587% of   20)
  Natural Minimal Basis     59.79156 ( 99.6526% of   60)
  Natural Rydberg Basis      0.20844 (  0.3474% of   60)
---------------------------------------------------------

   Atom No         Natural Electron Configuration
----------------------------------------------------------------------------
     P  1      [core]3s( 1.79)3p( 3.16)4s( 0.01)3d( 0.04)
     P  2      [core]3s( 1.79)3p( 3.16)4s( 0.01)3d( 0.04)
     P  3      [core]3s( 1.79)3p( 3.16)4s( 0.01)3d( 0.04)
     P  4      [core]3s( 1.79)3p( 3.16)4s( 0.01)3d( 0.04)


NATURAL BOND ORBITAL ANALYSIS:

                           Occupancies       Lewis Structure    Low   High
        Max    Occ     -------------------  -----------------   occ   occ
 Cycle  Ctr   Thresh    Lewis   non-Lewis     CR  BD  nC  LP    (L)   (NL)
============================================================================
   1     2     1.90    59.88606   0.11394     20   6   0   4     0      0
----------------------------------------------------------------------------

Structure accepted: No low occupancy Lewis orbitals

-------------------------------------------------------
  Core                     39.99982 (100.000% of  40)
  Valence Lewis            19.88624 ( 99.431% of  20)
 ==================       =============================
  Total Lewis              59.88606 ( 99.810% of  60)
 -----------------------------------------------------
  Valence non-Lewis         0.03525 (  0.059% of  60)
  Rydberg non-Lewis         0.07869 (  0.131% of  60)
 ==================       =============================
  Total non-Lewis           0.11394 (  0.190% of  60)
-------------------------------------------------------


    (Occupancy)   Bond orbital / Coefficients / Hybrids
------------------ Lewis ------------------------------------------------------
  1. (2.00000) CR ( 1) P  1           s(100.00%)
                                       1.0000  0.0000  0.0000  0.0000  0.0000
                                       0.0000  0.0000  0.0000  0.0000  0.0000
                                       0.0000  0.0000  0.0000  0.0000  0.0000
                                       0.0000  0.0000  0.0000  0.0000
  2. (1.99999) CR ( 2) P  1           s(100.00%)
                                       0.0000  1.0000  0.0000  0.0000  0.0000
                                       0.0000  0.0000  0.0000  0.0000  0.0000
                                       0.0000  0.0000  0.0000  0.0000  0.0000
                                       0.0000  0.0000  0.0000  0.0000
  3. (1.99999) CR ( 3) P  1           s(  0.00%)p 1.00(100.00%)
                                       0.0000  0.0000  0.0000  0.0000  0.0000
                                       1.0000  0.0000  0.0000  0.0000  0.0000
                                       0.0000  0.0000  0.0000  0.0000  0.0000
                                       0.0000  0.0000  0.0000  0.0000
  4. (1.99999) CR ( 4) P  1           s(  0.00%)p 1.00(100.00%)
```

```
                                 0.0000  0.0000  0.0000  0.0000  0.0000
                                 0.0000  0.0000  0.0000  1.0000  0.0000
                                 0.0000  0.0000  0.0000  0.0000  0.0000
                                 0.0000  0.0000  0.0000  0.0000
 5. (1.99999) CR ( 5) P  1      s(  0.00%)p 1.00(100.00%)
                                 0.0000  0.0000  0.0000  0.0000  0.0000
                                 0.0000  0.0000  0.0000  0.0000  0.0000
                                 0.0000  1.0000  0.0000  0.0000  0.0000
                                 0.0000  0.0000  0.0000  0.0000
 6. (2.00000) CR ( 1) P  2      s(100.00%)
                                 1.0000  0.0000  0.0000  0.0000  0.0000
                                 0.0000  0.0000  0.0000  0.0000  0.0000
                                 0.0000  0.0000  0.0000  0.0000  0.0000
                                 0.0000  0.0000  0.0000  0.0000
 7. (1.99999) CR ( 2) P  2      s(100.00%)
                                 0.0000  1.0000  0.0000  0.0000  0.0000
                                 0.0000  0.0000  0.0000  0.0000  0.0000
                                 0.0000  0.0000  0.0000  0.0000  0.0000
                                 0.0000  0.0000  0.0000  0.0000
 8. (1.99999) CR ( 3) P  2      s(  0.00%)p 1.00(100.00%)
                                 0.0000  0.0000  0.0000  0.0000  0.0000
                                 1.0000  0.0000  0.0000  0.0000  0.0000
                                 0.0000  0.0000  0.0000  0.0000  0.0000
                                 0.0000  0.0000  0.0000  0.0000
 9. (1.99999) CR ( 4) P  2      s(  0.00%)p 1.00(100.00%)
                                 0.0000  0.0000  0.0000  0.0000  0.0000
                                 0.0000  0.0000  0.0000  1.0000  0.0000
                                 0.0000  0.0000  0.0000  0.0000  0.0000
                                 0.0000  0.0000  0.0000  0.0000
10. (1.99999) CR ( 5) P  2      s(  0.00%)p 1.00(100.00%)
                                 0.0000  0.0000  0.0000  0.0000  0.0000
                                 0.0000  0.0000  0.0000  0.0000  0.0000
                                 0.0000  1.0000  0.0000  0.0000  0.0000
                                 0.0000  0.0000  0.0000  0.0000
11. (2.00000) CR ( 1) P  3      s(100.00%)
                                 1.0000  0.0000  0.0000  0.0000  0.0000
                                 0.0000  0.0000  0.0000  0.0000  0.0000
                                 0.0000  0.0000  0.0000  0.0000  0.0000
                                 0.0000  0.0000  0.0000  0.0000
12. (1.99999) CR ( 2) P  3      s(100.00%)
                                 0.0000  1.0000  0.0000  0.0000  0.0000
                                 0.0000  0.0000  0.0000  0.0000  0.0000
                                 0.0000  0.0000  0.0000  0.0000  0.0000
                                 0.0000  0.0000  0.0000  0.0000
13. (1.99999) CR ( 3) P  3      s(  0.00%)p 1.00(100.00%)
                                 0.0000  0.0000  0.0000  0.0000  0.0000
                                 1.0000  0.0000  0.0000  0.0000  0.0000
                                 0.0000  0.0000  0.0000  0.0000  0.0000
                                 0.0000  0.0000  0.0000  0.0000
14. (1.99999) CR ( 4) P  3      s(  0.00%)p 1.00(100.00%)
                                 0.0000  0.0000  0.0000  0.0000  0.0000
                                 0.0000  0.0000  0.0000  1.0000  0.0000
                                 0.0000  0.0000  0.0000  0.0000  0.0000
                                 0.0000  0.0000  0.0000  0.0000
15. (1.99999) CR ( 5) P  3      s(  0.00%)p 1.00(100.00%)
                                 0.0000  0.0000  0.0000  0.0000  0.0000
                                 0.0000  0.0000  0.0000  0.0000  0.0000
                                 0.0000  1.0000  0.0000  0.0000  0.0000
                                 0.0000  0.0000  0.0000  0.0000
16. (2.00000) CR ( 1) P  4      s(100.00%)
                                 1.0000  0.0000  0.0000  0.0000  0.0000
                                 0.0000  0.0000  0.0000  0.0000  0.0000
```

```
                                   0.0000  0.0000  0.0000  0.0000  0.0000
                                   0.0000  0.0000  0.0000  0.0000
 17. (1.99999) CR ( 2) P  4           s(100.00%)
                                   0.0000  1.0000  0.0000  0.0000  0.0000
                                   0.0000  0.0000  0.0000  0.0000  0.0000
                                   0.0000  0.0000  0.0000  0.0000  0.0000
                                   0.0000  0.0000  0.0000  0.0000
 18. (1.99999) CR ( 3) P  4           s(  0.00%)p 1.00(100.00%)
                                   0.0000  0.0000  0.0000  0.0000  0.0000
                                   1.0000  0.0000  0.0000  0.0000  0.0000
                                   0.0000  0.0000  0.0000  0.0000  0.0000
                                   0.0000  0.0000  0.0000  0.0000
 19. (1.99999) CR ( 4) P  4           s(  0.00%)p 1.00(100.00%)
                                   0.0000  0.0000  0.0000  0.0000  0.0000
                                   0.0000  0.0000  0.0000  1.0000  0.0000
                                   0.0000  0.0000  0.0000  0.0000  0.0000
                                   0.0000  0.0000  0.0000  0.0000
 20. (1.99999) CR ( 5) P  4           s(  0.00%)p 1.00(100.00%)
                                   0.0000  0.0000  0.0000  0.0000  0.0000
                                   0.0000  0.0000  0.0000  0.0000  0.0000
                                   0.0000  1.0000  0.0000  0.0000  0.0000
                                   0.0000  0.0000  0.0000  0.0000
 21. (1.99473) LP ( 1) P  1           s( 81.79%)p 0.22( 18.18%)d 0.00(  0.03%)
                                   0.0000  0.0000  0.9043  0.0107  0.0007
                                   0.0000  0.2460 -0.0080  0.0000 -0.2460
                                   0.0080  0.0000 -0.2460  0.0080  0.0102
                                   0.0102 -0.0102  0.0000  0.0000
 22. (1.99473) LP ( 1) P  2           s( 81.79%)p 0.22( 18.18%)d 0.00(  0.03%)
                                   0.0000  0.0000  0.9043  0.0107  0.0007
                                   0.0000 -0.2460  0.0080  0.0000  0.2460
                                  -0.0080  0.0000 -0.2460  0.0080  0.0102
                                  -0.0102  0.0102  0.0000  0.0000
 23. (1.99473) LP ( 1) P  3           s( 81.79%)p 0.22( 18.18%)d 0.00(  0.03%)
                                   0.0000  0.0000  0.9043  0.0107  0.0007
                                   0.0000 -0.2460  0.0080  0.0000 -0.2460
                                   0.0080  0.0000  0.2460 -0.0080 -0.0102
                                   0.0102  0.0102  0.0000  0.0000
 24. (1.99473) LP ( 1) P  4           s( 81.79%)p 0.22( 18.18%)d 0.00(  0.03%)
                                   0.0000  0.0000  0.9043  0.0107  0.0007
                                   0.0000  0.2460 -0.0080  0.0000  0.2460
                                  -0.0080  0.0000  0.2460 -0.0080 -0.0102
                                  -0.0102 -0.0102  0.0000  0.0000
 25. (1.98456) BD ( 1) P  1- P  2
                ( 50.00%)   0.7071* P  1 s(  6.12%)p15.20( 92.98%)d 0.15(  0.90%)
                                   0.0000  0.0000  0.2461 -0.0249 -0.0022
                                   0.0000 -0.6314  0.0031  0.0000  0.6314
                                  -0.0031  0.0000 -0.3635 -0.0144 -0.0805
                                  -0.0020  0.0020  0.0000 -0.0502
                ( 50.00%)   0.7071* P  2 s(  6.12%)p15.20( 92.98%)d 0.15(  0.90%)
                                   0.0000  0.0000  0.2461 -0.0249 -0.0022
                                   0.0000  0.6314 -0.0031  0.0000 -0.6314
                                   0.0031  0.0000 -0.3635 -0.0144 -0.0805
                                   0.0020 -0.0020  0.0000 -0.0502
 26. (1.98456) BD ( 1) P  1- P  3
                ( 50.00%)   0.7071* P  1 s(  6.12%)p15.20( 92.98%)d 0.15(  0.90%)
                                   0.0000  0.0000  0.2461 -0.0249 -0.0022
                                   0.0000 -0.6314  0.0031  0.0000 -0.3635
                                  -0.0144  0.0000  0.6314 -0.0031 -0.0020
                                  -0.0805  0.0020  0.0435  0.0251
                ( 50.00%)   0.7071* P  3 s(  6.12%)p15.20( 92.98%)d 0.15(  0.90%)
                                   0.0000  0.0000  0.2461 -0.0249 -0.0022
                                   0.0000  0.6314 -0.0031  0.0000 -0.3635
```

```
                                        -0.0144  0.0000 -0.6314  0.0031  0.0020
                                        -0.0805 -0.0020  0.0435  0.0251
 27. (1.98456) BD ( 1) P  1- P  4
              ( 50.00%)   0.7071* P  1 s(  6.12%)p15.20( 92.98%)d 0.15(  0.90%)
                                         0.0000  0.0000  0.2461 -0.0249 -0.0022
                                         0.0000  0.3635  0.0144  0.0000  0.6314
                                        -0.0031  0.0000  0.6314 -0.0031 -0.0020
                                        -0.0020  0.0805 -0.0435  0.0251
              ( 50.00%)   0.7071* P  4 s(  6.12%)p15.20( 92.98%)d 0.15(  0.90%)
                                         0.0000  0.0000  0.2461 -0.0249 -0.0022
                                         0.0000  0.3635  0.0144  0.0000 -0.6314
                                         0.0031  0.0000 -0.6314  0.0031  0.0020
                                         0.0020  0.0805 -0.0435  0.0251
 28. (1.98456) BD ( 1) P  2- P  3
              ( 50.00%)   0.7071* P  2 s(  6.12%)p15.20( 92.98%)d 0.15(  0.90%)
                                         0.0000  0.0000  0.2461 -0.0249 -0.0022
                                         0.0000 -0.3635 -0.0144  0.0000 -0.6314
                                         0.0031  0.0000  0.6314 -0.0031 -0.0020
                                         0.0020 -0.0805 -0.0435  0.0251
              ( 50.00%)   0.7071* P  3 s(  6.12%)p15.20( 92.98%)d 0.15(  0.90%)
                                         0.0000  0.0000  0.2461 -0.0249 -0.0022
                                         0.0000 -0.3635 -0.0144  0.0000  0.6314
                                        -0.0031  0.0000 -0.6314  0.0031  0.0020
                                        -0.0020 -0.0805 -0.0435  0.0251
 29. (1.98456) BD ( 1) P  2- P  4
              ( 50.00%)   0.7071* P  2 s(  6.12%)p15.20( 92.98%)d 0.15(  0.90%)
                                         0.0000  0.0000  0.2461 -0.0249 -0.0022
                                         0.0000  0.6314 -0.0031  0.0000  0.3635
                                         0.0144  0.0000  0.6314 -0.0031 -0.0020
                                         0.0805 -0.0020  0.0435  0.0251
              ( 50.00%)   0.7071* P  4 s(  6.12%)p15.20( 92.98%)d 0.15(  0.90%)
                                         0.0000  0.0000  0.2461 -0.0249 -0.0022
                                         0.0000 -0.6314  0.0031  0.0000  0.3635
                                         0.0144  0.0000 -0.6314  0.0031  0.0020
                                         0.0805  0.0020  0.0435  0.0251
 30. (1.98456) BD ( 1) P  3- P  4
              ( 50.00%)   0.7071* P  3 s(  6.12%)p15.20( 92.98%)d 0.15(  0.90%)
                                         0.0000  0.0000  0.2461 -0.0249 -0.0022
                                         0.0000  0.6314 -0.0031  0.0000  0.6314
                                        -0.0031  0.0000  0.3635  0.0144  0.0805
                                        -0.0020 -0.0020  0.0000 -0.0502
              ( 50.00%)   0.7071* P  4 s(  6.12%)p15.20( 92.98%)d 0.15(  0.90%)
                                         0.0000  0.0000  0.2461 -0.0249 -0.0022
                                         0.0000 -0.6314  0.0031  0.0000 -0.6314
                                         0.0031  0.0000  0.3635  0.0144  0.0805
                                         0.0020  0.0020  0.0000 -0.0502
---------------- non-Lewis ----------------------------------------------------
 31. (0.00587) BD*( 1) P  1- P  2
              ( 50.00%)   0.7071* P  1 s(  6.12%)p15.20( 92.98%)d 0.15(  0.90%)
                                         0.0000  0.0000  0.2461 -0.0249 -0.0022
                                         0.0000 -0.6314  0.0031  0.0000  0.6314
                                        -0.0031  0.0000 -0.3635 -0.0144 -0.0805
                                        -0.0020  0.0020  0.0000 -0.0502
              ( 50.00%)  -0.7071* P  2 s(  6.12%)p15.20( 92.98%)d 0.15(  0.90%)
                                         0.0000  0.0000  0.2461 -0.0249 -0.0022
                                         0.0000  0.6314 -0.0031  0.0000 -0.6314
                                         0.0031  0.0000 -0.3635 -0.0144 -0.0805
                                         0.0020 -0.0020  0.0000 -0.0502
 32. (0.00587) BD*( 1) P  1- P  3
              ( 50.00%)   0.7071* P  1 s(  6.12%)p15.20( 92.98%)d 0.15(  0.90%)
                                         0.0000  0.0000  0.2461 -0.0249 -0.0022
                                         0.0000 -0.6314  0.0031  0.0000 -0.3635
```

```
                              -0.0144  0.0000  0.6314 -0.0031 -0.0020
                              -0.0805  0.0020  0.0435  0.0251
              ( 50.00%)  -0.7071* P  3 s(  6.12%)p15.20( 92.98%)d 0.15(  0.90%)
                               0.0000  0.0000  0.2461 -0.0249 -0.0022
                               0.0000  0.6314 -0.0031  0.0000 -0.3635
                              -0.0144  0.0000 -0.6314  0.0031  0.0020
                              -0.0805 -0.0020  0.0435  0.0251
 33. (0.00587) BD*( 1) P  1- P  4
              ( 50.00%)   0.7071* P  1 s(  6.12%)p15.20( 92.98%)d 0.15(  0.90%)
                               0.0000  0.0000  0.2461 -0.0249 -0.0022
                               0.0000  0.3635  0.0144  0.0000  0.6314
                              -0.0031  0.0000  0.6314 -0.0031 -0.0020
                              -0.0020  0.0805 -0.0435  0.0251
              ( 50.00%)  -0.7071* P  4 s(  6.12%)p15.20( 92.98%)d 0.15(  0.90%)
                               0.0000  0.0000  0.2461 -0.0249 -0.0022
                               0.0000  0.3635  0.0144  0.0000 -0.6314
                               0.0031  0.0000 -0.6314  0.0031  0.0020
                               0.0020  0.0805 -0.0435  0.0251
 34. (0.00587) BD*( 1) P  2- P  3
              ( 50.00%)   0.7071* P  2 s(  6.12%)p15.20( 92.98%)d 0.15(  0.90%)
                               0.0000  0.0000  0.2461 -0.0249 -0.0022
                               0.0000 -0.3635 -0.0144  0.0000 -0.6314
                               0.0031  0.0000  0.6314 -0.0031 -0.0020
                               0.0020 -0.0805 -0.0435  0.0251
              ( 50.00%)  -0.7071* P  3 s(  6.12%)p15.20( 92.98%)d 0.15(  0.90%)
                               0.0000  0.0000  0.2461 -0.0249 -0.0022
                               0.0000 -0.3635 -0.0144  0.0000  0.6314
                              -0.0031  0.0000 -0.6314  0.0031  0.0020
                              -0.0020 -0.0805 -0.0435  0.0251
 35. (0.00587) BD*( 1) P  2- P  4
              ( 50.00%)   0.7071* P  2 s(  6.12%)p15.20( 92.98%)d 0.15(  0.90%)
                               0.0000  0.0000  0.2461 -0.0249 -0.0022
                               0.0000  0.6314 -0.0031  0.0000  0.3635
                               0.0144  0.0000  0.6314 -0.0031 -0.0020
                               0.0805 -0.0020  0.0435  0.0251
              ( 50.00%)  -0.7071* P  4 s(  6.12%)p15.20( 92.98%)d 0.15(  0.90%)
                               0.0000  0.0000  0.2461 -0.0249 -0.0022
                               0.0000 -0.6314  0.0031  0.0000  0.3635
                               0.0144  0.0000 -0.6314  0.0031  0.0020
                               0.0805  0.0020  0.0435  0.0251
 36. (0.00587) BD*( 1) P  3- P  4
              ( 50.00%)   0.7071* P  3 s(  6.12%)p15.20( 92.98%)d 0.15(  0.90%)
                               0.0000  0.0000  0.2461 -0.0249 -0.0022
                               0.0000  0.6314 -0.0031  0.0000  0.6314
                              -0.0031  0.0000  0.3635  0.0144  0.0805
                              -0.0020 -0.0020  0.0000 -0.0502
              ( 50.00%)  -0.7071* P  4 s(  6.12%)p15.20( 92.98%)d 0.15(  0.90%)
                               0.0000  0.0000  0.2461 -0.0249 -0.0022
                               0.0000 -0.6314  0.0031  0.0000 -0.6314
                               0.0031  0.0000  0.3635  0.0144  0.0805
                               0.0020  0.0020  0.0000 -0.0502
 37. (0.01014) RY ( 1) P  1           s( 37.01%)p 0.44( 16.32%)d 1.26( 46.67%)
                               0.0000  0.0000  0.0207  0.6078 -0.0148
                               0.0000 -0.0429  0.2293  0.0000  0.0429
                              -0.2293  0.0000  0.0429 -0.2293  0.3944
                               0.3944 -0.3944  0.0000  0.0000
 38. (0.00377) RY ( 2) P  1           s(  0.00%)p 1.00( 19.56%)d 4.11( 80.44%)
                               0.0000  0.0000  0.0000  0.0000  0.0000
                               0.0000  0.0142 -0.3105  0.0000  0.0144
                              -0.3142  0.0000 -0.0002  0.0037 -0.0035
                               0.3024  0.2988  0.7897  0.0080
 39. (0.00377) RY ( 3) P  1           s(  0.00%)p 1.00( 19.56%)d 4.11( 80.44%)
```

```
                                       0.0000  0.0000  0.0000  0.0000  0.0000
                                       0.0000 -0.0084  0.1835  0.0000  0.0081
                                      -0.1772  0.0000 -0.0166  0.3607 -0.3471
                                       0.1705 -0.1766 -0.0080  0.7897
 40. (0.00103) RY ( 4) P  1           s( 55.25%)p 0.63( 34.76%)d 0.18(  9.99%)
                                       0.0000  0.0000 -0.0022  0.7352 -0.1093
                                       0.0000 -0.0113 -0.3402  0.0000  0.0113
                                       0.3402  0.0000  0.0113  0.3402 -0.1825
                                      -0.1825  0.1825  0.0000  0.0000
 41. (0.00048) RY ( 5) P  1           s(  0.00%)p 1.00( 13.06%)d 6.66( 86.94%)
                                       0.0000  0.0000  0.0000  0.0000  0.0000
                                       0.0000 -0.0088  0.0337  0.0000 -0.0687
                                       0.2623  0.0000  0.0598 -0.2286 -0.5815
                                       0.6672  0.0857 -0.1681 -0.2248
 42. (0.00048) RY ( 6) P  1           s(  0.00%)p 1.00( 13.06%)d 6.66( 86.94%)
                                       0.0000  0.0000  0.0000  0.0000  0.0000
                                       0.0000 -0.0742  0.2834  0.0000 -0.0295
                                       0.1125  0.0000 -0.0447  0.1709  0.4347
                                       0.2862  0.7209 -0.2248  0.1681
 43. (0.00000) RY ( 7) P  1           s(  7.95%)p 6.25( 49.72%)d 5.32( 42.33%)
 44. (0.00000) RY ( 8) P  1           s(  0.00%)p 1.00( 68.38%)d 0.46( 31.62%)
 45. (0.00000) RY ( 9) P  1           s( 99.64%)p 0.00(  0.09%)d 0.00(  0.27%)
 46. (0.00000) RY (10) P  1           s(  0.00%)p 1.00( 68.38%)d 0.46( 31.62%)
 47. (0.01014) RY ( 1) P  2           s( 37.01%)p 0.44( 16.32%)d 1.26( 46.67%)
                                       0.0000  0.0000  0.0207  0.6078 -0.0148
                                       0.0000  0.0429 -0.2293  0.0000 -0.0429
                                       0.2293  0.0000  0.0429 -0.2293  0.3944
                                      -0.3944  0.3944  0.0000  0.0000
 48. (0.00377) RY ( 2) P  2           s(  0.00%)p 1.00( 19.56%)d 4.11( 80.44%)
                                       0.0000  0.0000  0.0000  0.0000  0.0000
                                       0.0000 -0.0142  0.3105  0.0000 -0.0144
                                       0.3142  0.0000 -0.0002  0.0036 -0.0035
                                      -0.3024 -0.2988  0.7897  0.0080
 49. (0.00377) RY ( 3) P  2           s(  0.00%)p 1.00( 19.56%)d 4.11( 80.44%)
                                       0.0000  0.0000  0.0000  0.0000  0.0000
                                       0.0000  0.0084 -0.1835  0.0000 -0.0081
                                       0.1772  0.0000 -0.0166  0.3607 -0.3471
                                      -0.1705  0.1766 -0.0080  0.7897
 50. (0.00103) RY ( 4) P  2           s( 55.25%)p 0.63( 34.76%)d 0.18(  9.99%)
                                       0.0000  0.0000 -0.0022  0.7352 -0.1093
                                       0.0000  0.0113  0.3402  0.0000 -0.0113
                                      -0.3402  0.0000  0.0113  0.3402 -0.1825
                                       0.1825 -0.1825  0.0000  0.0000
 51. (0.00048) RY ( 5) P  2           s(  0.00%)p 1.00( 13.06%)d 6.66( 86.94%)
                                       0.0000  0.0000  0.0000  0.0000  0.0000
                                       0.0000 -0.0088  0.0336  0.0000 -0.0687
                                       0.2623  0.0000 -0.0598  0.2287  0.5816
                                       0.6671  0.0856  0.1680  0.2249
 52. (0.00048) RY ( 6) P  2           s(  0.00%)p 1.00( 13.06%)d 6.66( 86.94%)
                                       0.0000  0.0000  0.0000  0.0000  0.0000
                                       0.0000 -0.0742  0.2835  0.0000 -0.0295
                                       0.1126  0.0000  0.0447 -0.1709 -0.4346
                                       0.2864  0.7209  0.2249 -0.1680
 53. (0.00000) RY ( 7) P  2           s(  7.95%)p 6.25( 49.72%)d 5.32( 42.33%)
 54. (0.00000) RY ( 8) P  2           s(  0.00%)p 1.00( 68.38%)d 0.46( 31.62%)
 55. (0.00000) RY ( 9) P  2           s( 99.64%)p 0.00(  0.09%)d 0.00(  0.27%)
 56. (0.00000) RY (10) P  2           s(  0.00%)p 1.00( 68.38%)d 0.46( 31.62%)
 57. (0.01014) RY ( 1) P  3           s( 37.01%)p 0.44( 16.32%)d 1.26( 46.67%)
                                       0.0000  0.0000  0.0207  0.6078 -0.0148
                                       0.0000  0.0429 -0.2293  0.0000  0.0429
                                      -0.2293  0.0000 -0.0429  0.2293 -0.3944
                                       0.3944  0.3944  0.0000  0.0000
```

```
58. (0.00377) RY ( 2) P  3           s(  0.00%)p 1.00( 19.56%)d 4.11( 80.44%)
                                      0.0000  0.0000  0.0000  0.0000  0.0000
                                      0.0000 -0.0136  0.2970  0.0000  0.0149
                                     -0.3257  0.0000  0.0013 -0.0287  0.0276
                                      0.3135 -0.2859  0.7872  0.0628
59. (0.00377) RY ( 3) P  3           s(  0.00%)p 1.00( 19.56%)d 4.11( 80.44%)
                                      0.0000  0.0000  0.0000  0.0000  0.0000
                                      0.0000  0.0094 -0.2046  0.0000  0.0071
                                     -0.1549  0.0000  0.0165 -0.3596  0.3460
                                      0.1491  0.1969 -0.0628  0.7872
60. (0.00103) RY ( 4) P  3           s( 55.25%)p 0.63( 34.76%)d 0.18(  9.99%)
                                      0.0000  0.0000 -0.0022  0.7352 -0.1093
                                      0.0000  0.0113  0.3402  0.0000  0.0113
                                      0.3402  0.0000 -0.0113 -0.3402  0.1825
                                     -0.1825 -0.1825  0.0000  0.0000
61. (0.00048) RY ( 5) P  3           s(  0.00%)p 1.00( 13.06%)d 6.66( 86.94%)
                                      0.0000  0.0000  0.0000  0.0000  0.0000
                                      0.0000 -0.0694  0.2650  0.0000  0.0587
                                     -0.2244  0.0000 -0.0106  0.0406  0.1033
                                     -0.5706  0.6740  0.2779 -0.0400
62. (0.00048) RY ( 6) P  3           s(  0.00%)p 1.00( 13.06%)d 6.66( 86.94%)
                                      0.0000  0.0000  0.0000  0.0000  0.0000
                                      0.0000 -0.0278  0.1061  0.0000 -0.0462
                                      0.1765  0.0000 -0.0739  0.2825  0.7186
                                      0.4488  0.2698 -0.0400 -0.2779
63. (0.00000) RY ( 7) P  3           s(  7.95%)p 6.25( 49.72%)d 5.32( 42.33%)
64. (0.00000) RY ( 8) P  3           s(  0.00%)p 1.00( 68.38%)d 0.46( 31.62%)
65. (0.00000) RY ( 9) P  3           s( 99.64%)p 0.00(  0.09%)d 0.00(  0.27%)
66. (0.00000) RY (10) P  3           s(  0.00%)p 1.00( 68.38%)d 0.46( 31.62%)
67. (0.01014) RY ( 1) P  4           s( 37.01%)p 0.44( 16.32%)d 1.26( 46.67%)
                                      0.0000  0.0000  0.0207  0.6078 -0.0148
                                      0.0000 -0.0429  0.2293  0.0000 -0.0429
                                      0.2293  0.0000 -0.0429  0.2293 -0.3944
                                     -0.3944 -0.3944  0.0000  0.0000
68. (0.00377) RY ( 2) P  4           s(  0.00%)p 1.00( 19.56%)d 4.11( 80.44%)
                                      0.0000  0.0000  0.0000  0.0000  0.0000
                                      0.0000  0.0136 -0.2970  0.0000 -0.0149
                                      0.3257  0.0000  0.0013 -0.0287  0.0276
                                     -0.3135  0.2859  0.7873  0.0628
69. (0.00377) RY ( 3) P  4           s(  0.00%)p 1.00( 19.56%)d 4.11( 80.44%)
                                      0.0000  0.0000  0.0000  0.0000  0.0000
                                      0.0000 -0.0094  0.2046  0.0000 -0.0071
                                      0.1549  0.0000  0.0165 -0.3596  0.3460
                                     -0.1491 -0.1969 -0.0628  0.7873
70. (0.00103) RY ( 4) P  4           s( 55.25%)p 0.63( 34.76%)d 0.18(  9.99%)
                                      0.0000  0.0000 -0.0022  0.7352 -0.1093
                                      0.0000 -0.0113 -0.3402  0.0000 -0.0113
                                     -0.3402  0.0000 -0.0113 -0.3402  0.1825
                                      0.1825  0.1825  0.0000  0.0000
71. (0.00048) RY ( 5) P  4           s(  0.00%)p 1.00( 13.06%)d 6.66( 86.94%)
                                      0.0000  0.0000  0.0000  0.0000  0.0000
                                      0.0000 -0.0694  0.2650  0.0000  0.0587
                                     -0.2243  0.0000  0.0106 -0.0407 -0.1035
                                     -0.5706  0.6741 -0.2779  0.0400
72. (0.00048) RY ( 6) P  4           s(  0.00%)p 1.00( 13.06%)d 6.66( 86.94%)
                                      0.0000  0.0000  0.0000  0.0000  0.0000
                                      0.0000  0.0277 -0.1060  0.0000  0.0462
                                     -0.1765  0.0000 -0.0739  0.2825  0.7186
                                     -0.4489 -0.2697 -0.0400 -0.2779
73. (0.00000) RY ( 7) P  4           s(  7.95%)p 6.25( 49.72%)d 5.32( 42.33%)
74. (0.00000) RY ( 8) P  4           s(  0.00%)p 1.00( 68.38%)d 0.46( 31.62%)
75. (0.00000) RY ( 9) P  4           s( 99.64%)p 0.00(  0.09%)d 0.00(  0.27%)
```

76. (0.00000) RY (10) P 4   s(0.00%)p 1.00(68.38%)d 0.46(31.62%)

NHO DIRECTIONALITY AND BOND BENDING (deviation from line of nuclear centers at the position of maximum hybrid amplitude)

[Thresholds for printing: angular deviation > 1.0 degree]
p- or d-character > 25.0%
orbital occupancy > 0.10e

	Line of Centers		Hybrid 1			Hybrid 2		
NBO	Theta	Phi	Theta	Phi	Dev	Theta	Phi	Dev
25. BD (1) P 1- P 2	90.0	135.0	106.5	135.0	16.5	106.5	315.0	16.5
26. BD (1) P 1- P 3	45.0	180.0	47.3	202.8	16.5	132.7	337.2	16.5
27. BD (1) P 1- P 4	45.0	90.0	47.3	67.2	16.5	132.7	292.8	16.5
28. BD (1) P 2- P 3	45.0	270.0	47.3	247.2	16.5	132.7	112.8	16.5
29. BD (1) P 2- P 4	45.0	0.0	47.3	22.8	16.5	132.7	157.2	16.5
30. BD (1) P 3- P 4	90.0	45.0	73.5	45.0	16.5	73.5	225.0	16.5

SECOND ORDER PERTURBATION THEORY ANALYSIS OF FOCK MATRIX IN NBO BASIS

Threshold for printing: 0.50 kcal/mol

Donor (L) NBO	Acceptor (NL) NBO	E(2) kcal/mol	E(NL)-E(L) a.u.	F(L,NL) a.u.
within unit 1				
25. BD (1) P 1- P 2	32. BD*(1) P 1- P 3	0.60	0.43	0.014
25. BD (1) P 1- P 2	33. BD*(1) P 1- P 4	0.60	0.43	0.014
25. BD (1) P 1- P 2	34. BD*(1) P 2- P 3	0.60	0.43	0.014
25. BD (1) P 1- P 2	35. BD*(1) P 2- P 4	0.60	0.43	0.014
25. BD (1) P 1- P 2	57. RY (1) P 3	1.35	0.79	0.029
25. BD (1) P 1- P 2	59. RY (3) P 3	1.33	0.99	0.032
25. BD (1) P 1- P 2	67. RY (1) P 4	1.35	0.79	0.029
25. BD (1) P 1- P 2	69. RY (3) P 4	1.33	0.99	0.032
26. BD (1) P 1- P 3	31. BD*(1) P 1- P 2	0.60	0.43	0.014
26. BD (1) P 1- P 3	33. BD*(1) P 1- P 4	0.60	0.43	0.014
26. BD (1) P 1- P 3	34. BD*(1) P 2- P 3	0.60	0.43	0.014
26. BD (1) P 1- P 3	36. BD*(1) P 3- P 4	0.60	0.43	0.014
26. BD (1) P 1- P 3	47. RY (1) P 2	1.35	0.79	0.029
26. BD (1) P 1- P 3	48. RY (2) P 2	1.02	0.99	0.028
26. BD (1) P 1- P 3	67. RY (1) P 4	1.35	0.79	0.029
26. BD (1) P 1- P 3	68. RY (2) P 4	1.09	0.99	0.029
27. BD (1) P 1- P 4	31. BD*(1) P 1- P 2	0.60	0.43	0.014
27. BD (1) P 1- P 4	32. BD*(1) P 1- P 3	0.60	0.43	0.014
27. BD (1) P 1- P 4	35. BD*(1) P 2- P 4	0.60	0.43	0.014
27. BD (1) P 1- P 4	36. BD*(1) P 3- P 4	0.60	0.43	0.014
27. BD (1) P 1- P 4	47. RY (1) P 2	1.35	0.79	0.029
27. BD (1) P 1- P 4	48. RY (2) P 2	0.99	0.99	0.028
27. BD (1) P 1- P 4	57. RY (1) P 3	1.35	0.79	0.029
27. BD (1) P 1- P 4	58. RY (2) P 3	0.91	0.99	0.027
28. BD (1) P 2- P 3	31. BD*(1) P 1- P 2	0.60	0.43	0.014
28. BD (1) P 2- P 3	32. BD*(1) P 1- P 3	0.60	0.43	0.014
28. BD (1) P 2- P 3	35. BD*(1) P 2- P 4	0.60	0.43	0.014
28. BD (1) P 2- P 3	36. BD*(1) P 3- P 4	0.60	0.43	0.014
28. BD (1) P 2- P 3	37. RY (1) P 1	1.35	0.79	0.029
28. BD (1) P 2- P 3	38. RY (2) P 1	0.99	0.99	0.028
28. BD (1) P 2- P 3	67. RY (1) P 4	1.35	0.79	0.029
28. BD (1) P 2- P 3	68. RY (2) P 4	0.91	0.99	0.027

```
  29. BD ( 1) P  2- P  4      31. BD*( 1) P  1- P  2      0.60    0.43   0.014
  29. BD ( 1) P  2- P  4      33. BD*( 1) P  1- P  4      0.60    0.43   0.014
  29. BD ( 1) P  2- P  4      34. BD*( 1) P  2- P  3      0.60    0.43   0.014
  29. BD ( 1) P  2- P  4      36. BD*( 1) P  3- P  4      0.60    0.43   0.014
  29. BD ( 1) P  2- P  4      37. RY ( 1) P  1            1.35    0.79   0.029
  29. BD ( 1) P  2- P  4      38. RY ( 2) P  1            1.02    0.99   0.028
  29. BD ( 1) P  2- P  4      57. RY ( 1) P  3            1.35    0.79   0.029
  29. BD ( 1) P  2- P  4      58. RY ( 2) P  3            1.09    0.99   0.029
  30. BD ( 1) P  3- P  4      32. BD*( 1) P  1- P  3      0.60    0.43   0.014
  30. BD ( 1) P  3- P  4      33. BD*( 1) P  1- P  4      0.60    0.43   0.014
  30. BD ( 1) P  3- P  4      34. BD*( 1) P  2- P  3      0.60    0.43   0.014
  30. BD ( 1) P  3- P  4      35. BD*( 1) P  2- P  4      0.60    0.43   0.014
  30. BD ( 1) P  3- P  4      37. RY ( 1) P  1            1.35    0.79   0.029
  30. BD ( 1) P  3- P  4      39. RY ( 3) P  1            1.34    0.99   0.032
  30. BD ( 1) P  3- P  4      47. RY ( 1) P  2            1.35    0.79   0.029
  30. BD ( 1) P  3- P  4      49. RY ( 3) P  2            1.34    0.99   0.032


NATURAL BOND ORBITALS (Summary):

                                                     Principal Delocalizations
          NBO                 Occupancy    Energy   (geminal,vicinal,remote)
===============================================================================
Molecular unit  1  (P4)
------ Lewis --------------------------------------
   1. CR ( 1) P  1             2.00000   -75.55593
   2. CR ( 2) P  1             1.99999    -8.12344
   3. CR ( 3) P  1             1.99999    -4.73745
   4. CR ( 4) P  1             1.99999    -4.73745
   5. CR ( 5) P  1             1.99999    -4.73745
   6. CR ( 1) P  2             2.00000   -75.55593
   7. CR ( 2) P  2             1.99999    -8.12344
   8. CR ( 3) P  2             1.99999    -4.73745
   9. CR ( 4) P  2             1.99999    -4.73745
  10. CR ( 5) P  2             1.99999    -4.73745
  11. CR ( 1) P  3             2.00000   -75.55593
  12. CR ( 2) P  3             1.99999    -8.12344
  13. CR ( 3) P  3             1.99999    -4.73745
  14. CR ( 4) P  3             1.99999    -4.73745
  15. CR ( 5) P  3             1.99999    -4.73745
  16. CR ( 1) P  4             2.00000   -75.55593
  17. CR ( 2) P  4             1.99999    -8.12344
  18. CR ( 3) P  4             1.99999    -4.73745
  19. CR ( 4) P  4             1.99999    -4.73745
  20. CR ( 5) P  4             1.99999    -4.73745
  21. LP ( 1) P  1             1.99473    -0.45939
  22. LP ( 1) P  2             1.99473    -0.45939
  23. LP ( 1) P  3             1.99473    -0.45939
  24. LP ( 1) P  4             1.99473    -0.45939
  25. BD ( 1) P  1- P  2       1.98456    -0.39151  57(v),67(v),59(v),69(v)
                                                    32(g),33(g),34(g),35(g)
  26. BD ( 1) P  1- P  3       1.98456    -0.39151  47(v),67(v),68(v),48(v)
                                                    31(g),33(g),34(g),36(g)
  27. BD ( 1) P  1- P  4       1.98456    -0.39151  47(v),57(v),48(v),58(v)
                                                    31(g),32(g),35(g),36(g)
  28. BD ( 1) P  2- P  3       1.98456    -0.39151  37(v),67(v),38(v),68(v)
                                                    31(g),32(g),35(g),36(g)
  29. BD ( 1) P  2- P  4       1.98456    -0.39151  37(v),57(v),58(v),38(v)
                                                    31(g),33(g),34(g),36(g)
  30. BD ( 1) P  3- P  4       1.98456    -0.39151  37(v),47(v),39(v),49(v)
                                                    32(g),33(g),34(g),35(g)
------ non-Lewis ----------------------------------
```

```
  31. BD*( 1) P  1- P  2       0.00587     0.03595
  32. BD*( 1) P  1- P  3       0.00587     0.03595
  33. BD*( 1) P  1- P  4       0.00587     0.03595
  34. BD*( 1) P  2- P  3       0.00587     0.03595
  35. BD*( 1) P  2- P  4       0.00587     0.03595
  36. BD*( 1) P  3- P  4       0.00587     0.03595
  37. RY ( 1) P  1             0.01014     0.39729
  38. RY ( 2) P  1             0.00377     0.59486
  39. RY ( 3) P  1             0.00377     0.59486
  40. RY ( 4) P  1             0.00103     0.54715
  41. RY ( 5) P  1             0.00048     0.67794
  42. RY ( 6) P  1             0.00048     0.67794
  43. RY ( 7) P  1             0.00000     0.68133
  44. RY ( 8) P  1             0.00000     0.52204
  45. RY ( 9) P  1             0.00000     3.34423
  46. RY (10) P  1             0.00000     0.52204
  47. RY ( 1) P  2             0.01014     0.39729
  48. RY ( 2) P  2             0.00377     0.59486
  49. RY ( 3) P  2             0.00377     0.59486
  50. RY ( 4) P  2             0.00103     0.54715
  51. RY ( 5) P  2             0.00048     0.67794
  52. RY ( 6) P  2             0.00048     0.67794
  53. RY ( 7) P  2             0.00000     0.68133
  54. RY ( 8) P  2             0.00000     0.52204
  55. RY ( 9) P  2             0.00000     3.34423
  56. RY (10) P  2             0.00000     0.52204
  57. RY ( 1) P  3             0.01014     0.39729
  58. RY ( 2) P  3             0.00377     0.59486
  59. RY ( 3) P  3             0.00377     0.59486
  60. RY ( 4) P  3             0.00103     0.54715
  61. RY ( 5) P  3             0.00048     0.67794
  62. RY ( 6) P  3             0.00048     0.67794
  63. RY ( 7) P  3             0.00000     0.68133
  64. RY ( 8) P  3             0.00000     0.52204
  65. RY ( 9) P  3             0.00000     3.34423
  66. RY (10) P  3             0.00000     0.52204
  67. RY ( 1) P  4             0.01014     0.39729
  68. RY ( 2) P  4             0.00377     0.59486
  69. RY ( 3) P  4             0.00377     0.59486
  70. RY ( 4) P  4             0.00103     0.54715
  71. RY ( 5) P  4             0.00048     0.67794
  72. RY ( 6) P  4             0.00048     0.67794
  73. RY ( 7) P  4             0.00000     0.68133
  74. RY ( 8) P  4             0.00000     0.52204
  75. RY ( 9) P  4             0.00000     3.34423
  76. RY (10) P  4             0.00000     0.52204
          -------------------------------
                 Total Lewis   59.88606  ( 99.8101%)
           Valence non-Lewis    0.03525  (  0.0587%)
           Rydberg non-Lewis    0.07869  (  0.1312%)
          -------------------------------
               Total unit  1   60.00000  (100.0000%)
              Charge unit  1    0.00000

$CHOOSE
  LONE 1 1 2 1 3 1 4 1 END
  BOND S 1 2 S 1 3 S 1 4 S 2 3 S 2 4 S 3 4 END
$END


NATURAL RESONANCE THEORY ANALYSIS:
```

```
Maximum reference structures : 20
Maximum resonance structures : 5000
Memory requirements : 86586872 words of 149782540 available

55 candidate reference structure(s) calculated by SR LEWIS
Initial loops searched 163 bonding pattern(s); 1 was retained
Delocalization list threshold set to 1.00 kcal/mol for reference 1
Reference   1:  rho*=0.11394, f(w)=0.00000 converged after   0 iterations

                                               fractional accuracy f(w)
                 non-Lewis             -------------------------------------
 Ref     Wgt      density      d(0)       all NBOs     val+core     valence
----------------------------------------------------------------------------
  1    1.00000    0.11394    0.00548     0.00000      0.00000      0.00000


TOPO matrix for the leading resonance structure:

    Atom  1   2   3   4
    ---- --- --- --- ---
  1.  P   1   1   1   1
  2.  P   1   1   1   1
  3.  P   1   1   1   1
  4.  P   1   1   1   1

        Resonance
   RS   Weight(%)                  Added(Removed)
---------------------------------------------------------------------------
   1*    100.00
---------------------------------------------------------------------------
         100.00   * Total *                [* = reference structure]


Natural Bond Order:  (total/covalent/ionic)

    Atom       1      2      3      4
    ----   ------ ------ ------ ------
  1.  P  t 1.0000 1.0000 1.0000 1.0000
         c   ---  1.0000 1.0000 1.0000
         i   ---  0.0000 0.0000 0.0000

  2.  P  t 1.0000 1.0000 1.0000 1.0000
         c 1.0000   ---  1.0000 1.0000
         i 0.0000   ---  0.0000 0.0000

  3.  P  t 1.0000 1.0000 1.0000 1.0000
         c 1.0000 1.0000   ---  1.0000
         i 0.0000 0.0000   ---  0.0000

  4.  P  t 1.0000 1.0000 1.0000 1.0000
         c 1.0000 1.0000 1.0000   ---
         i 0.0000 0.0000 0.0000   ---


Natural Atomic Valencies:

                     Co-    Electro-
    Atom  Valency  Valency  Valency
    ----  -------  -------  -------
  1.  P    3.0000   3.0000   0.0000
  2.  P    3.0000   3.0000   0.0000
  3.  P    3.0000   3.0000   0.0000
```

```
  4.  P    3.0000   3.0000   0.0000


$NRTSTR
  STR        ! Wgt =100.00%
    LONE 1 1 2 1 3 1 4 1 END
    BOND S 1 2 S 1 3 S 1 4 S 2 3 S 2 4 S 3 4 END
  END
$END

NBO analysis completed in 0.34 CPU seconds (1 wall second)
Maximum scratch memory used by NBO was 86919867 words (663.15 MB)
..... done with NBO analysis .....
```
